# Author Correction: Triggering the 2022 eruption of Mauna Loa

**DOI:** 10.1038/s41467-025-56706-z

**Published:** 2025-02-05

**Authors:** Kendra J. Lynn, Drew T. Downs, Frank A. Trusdell, Penny E. Wieser, Berenise Rangel, Baylee McDade, Alicia J. Hotovec-Ellis, Ninfa Bennington, Kyle R. Anderson, Dawn C. S. Ruth, Charlotte L. DeVitre, Andria P. Ellis, Patricia A. Nadeau, Laura Clor, Peter Kelly, Peter J. Dotray, Jefferson C. Chang

**Affiliations:** 1 U.S. Geological Survey, Hawaiian Volcano Observatory, Hilo, HI USA; 2 Department of Earth and Planetary Science, University of California, Berkeley, Berkeley, CA USA; 3 U.S. Geological Survey, California Volcano Observatory, Moffett Field, CA USA; 4 U.S. Geological Survey, Cascades Volcano Observatory, Vancouver, WA USA

**Keywords:** Natural hazards, Solid Earth sciences

Correction to: *Nature Communications* 10.1038/s41467-024-52881-7, published online 12 November 2024

In the version of the article initially published, there was an error in the EarthChem Library doi listed in the Data availability section and ref. 76, which has now been amended to read 10.60520/IEDA/113447 in the HTML and PDF versions of the article.

