# Supplementary material for: Triggering the 2022 eruption of Mauna Loa
Source: Nat Commun. 2024 Nov 12;15:9451. doi: 10.1038/s41467-024-52881-7 (PMC11557896; doi:10.1038/s41467-024-52881-7)

Supplementary Material For:

Triggering the 2022 eruption of Mauna Loa

Kendra J. Lynn[1*], Drew T. Downs[1], Frank A. Trusdell[1], Penny E. Wieser[2], Berenise Rangel[2], Baylee McDade[1], Alicia Hotovec-Ellis[3], Ninfa Bennington[1], Kyle R. Anderson[3], Dawn C.S. Ruth[3], Charlotte DeVitre[2], Andria P. Ellis[1], Patricia A. Nadeau[1], Laura Clor[4], Peter Kelly[4], Peter J. Dotray[1], Jefferson C. Chang[1]

* Corresponding author: klynn@usgs.gov

[1] U.S. Geological Survey
Hawaiian Volcano Observatory
1266 Kamehameha Avenue, Suite A8
Hilo, HI 96720, USA

[2] University of California Berkeley
Department of Earth and Planetary Science
307 McCone Hall
Berkeley, CA 94720-4767, USA

[3] U.S. Geological Survey
California Volcano Observatory
Building 19, 350 N. Akron Road
Moffett Field, CA 94035, USA

[4] U.S. Geological Survey
Cascades Volcano Observatory
1300 SE Cardinal Court
Vancouver, WA 98683, USA

Eruption Overview

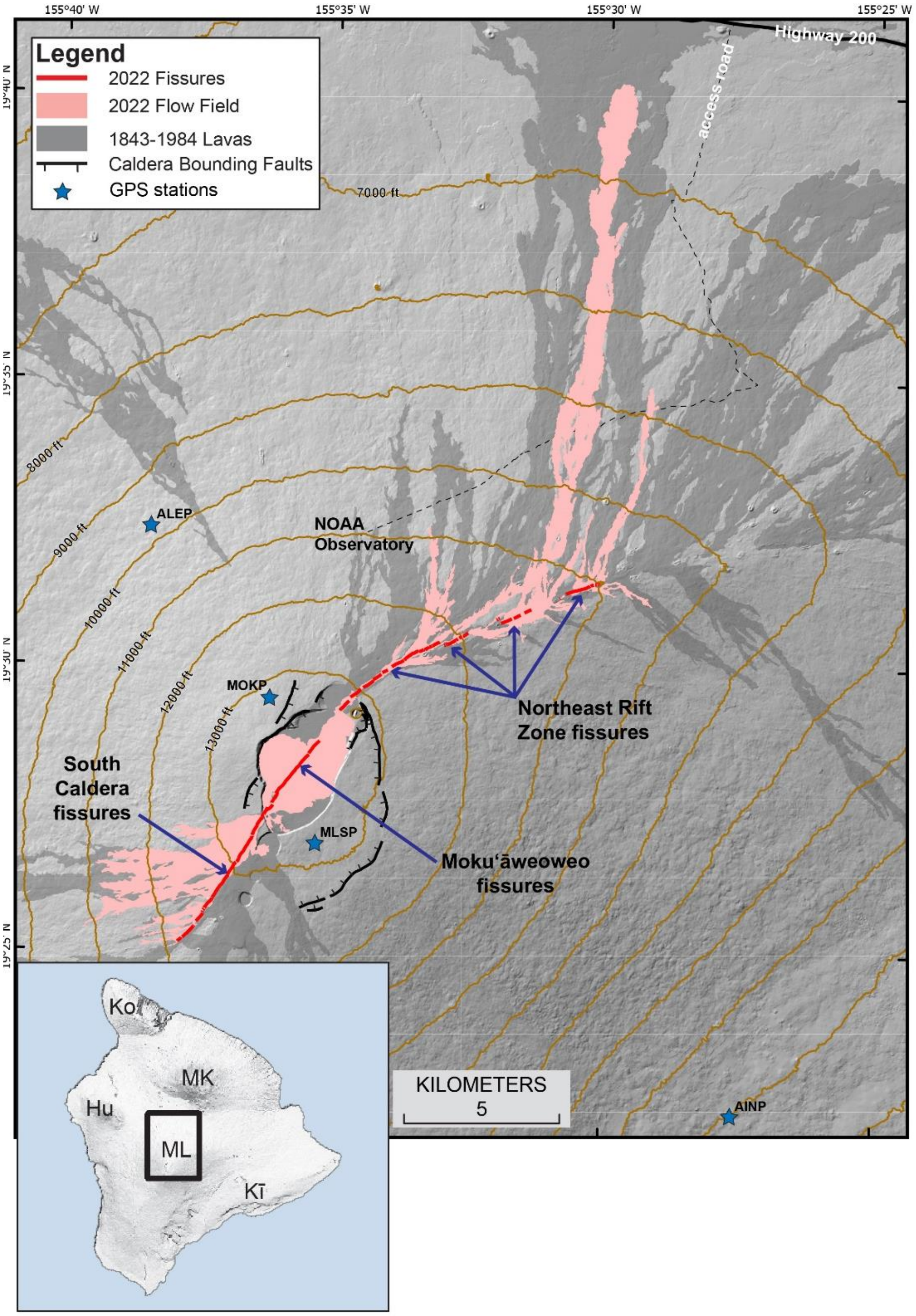

Fig. S1. Extended view of Mauna Loa 2022 eruption and GPS station locations. Map of Mauna Loa summit and upper Northeast Rift Zone regions (Island of Hawai'i on inset map) with 2022 lava flow field[1] (pink), all lava flows since 1843 (dark gray)[2-3], 1,000 ft (~305 m) contours (brown lines), and GPS stations. Basemap generated from a 2005 NOAA digital terrain model[4] and a 1983 USGS digital elevation model[5]. Caldera bounding faults are denoted as black lines with teeth pointing to the downthrown side and 2022 eruptive fissures with red lines. The 2022 flows cross the NOAA Observatory access road (dashed black line) and came within ~3 km of Highway 200 (Daniel K. Inouye Highway, "Saddle Road").

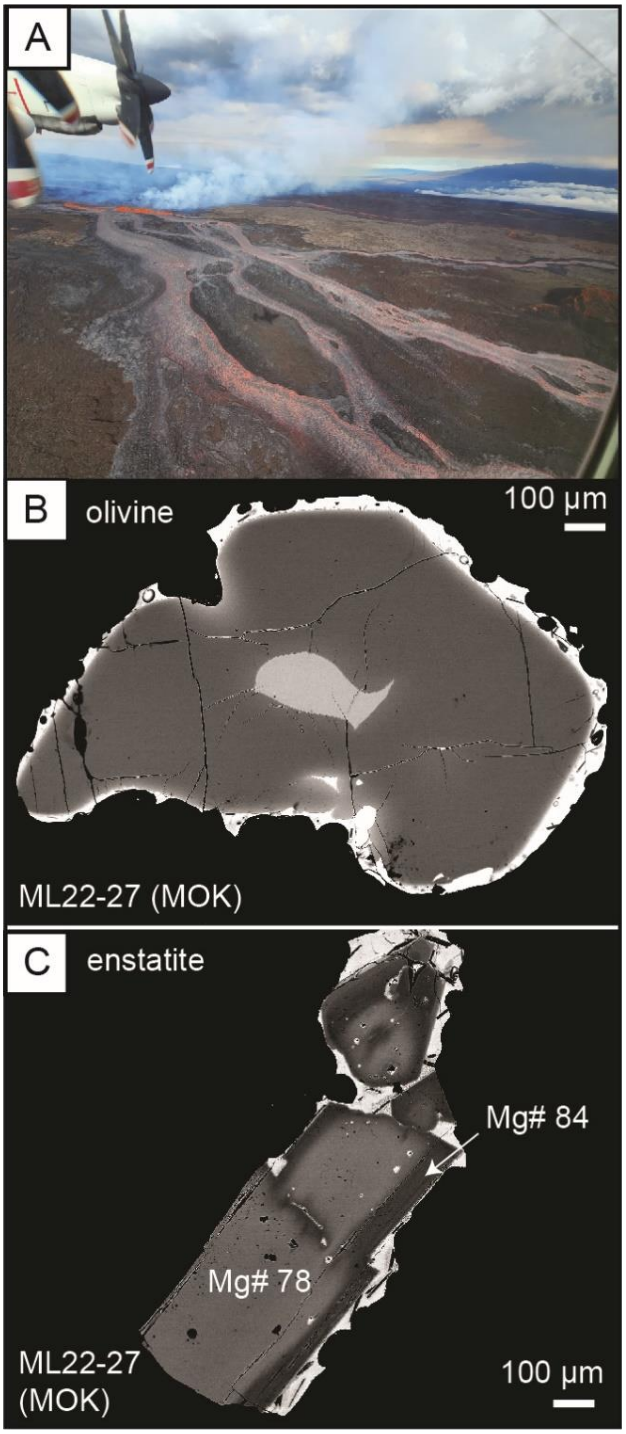

Fig. S2. Images of eruption and mineral cargo. (A) Photograph of the 2022 Northeast Rift Zone lava channels and flows, view toward the northwest. USGS Photo, public domain. **(B)** Backscattered electron (BSE) image of a typical olivine phenocryst from a 2022 tephra sample (ML22-27, collected in Mokuʻāweoweo). **(C)** BSE image of a rare enstatite phenocryst from sample ML22-27, displaying reverse zoning with a Mg# 78 core to Mg# 84 overgrowth rim.

Previous mineral studies on Mauna Loa

Prior to this 2022 eruption study, there has been very little published Mauna Loa mineral chemistry data from which to make interpretations about magma storage and transport. Although Tucker et al.[6] presented a Mauna Loa melt inclusion study, Wieser et al.[7] subsequently showed that because vapor bubbles were not measured in the Tucker et al. melt inclusions, the data could not be used to retrieve accurate barometry calculations. Thus, during the run-up to the 2022 eruption there were no published mineral chemistry, barometry, or diffusion studies for Mauna Loa eruptions from 1843 CE to present that could be reliably used for interpretations about magma storage and transport. Some mineral zoning patterns were described in [8] for the 1868 eruption and some olivine compositions were collected by Riker for Mauna Loa eruptions in the past 200 years[9-10]. Couperthwaite et al.[11] presented a diffusion study for the 2.1 ka Moinui lava flow field, and [12] presented a diffusion study of the 240 yr B.P. Hapaimamo eruption. In 2023 (after the 2022 eruption), a mineral zoning and diffusion study was published for the 1950 eruption[13].

Extended Methods

Mineral Sample Details

Four tephra samples were processed in large volumes (>1 m^3) for mineral separation and analysis: ML22-27 (Moku'āweoweo sample), ML22-87 (Northeast Rift Zone), and ML22-88 and -89 (South Caldera; Fig. 1). Samples 27, 88, and 89 were erupted and air quenched in the opening ~6 hours of the eruption, overnight from 27-28 November 2022. Sample 87 from the Northeast Rift Zone was collected after the eruption had ended and represents a bulk sample of tephra from the main vent that was active through December 10, 2022. Additional sample metadata, ED-XRF whole rock chemistry, and grain size and shape data for samples 27 and 87 can be found in [14].

Table S1. Sample information.

Sample Number	Latitude	Longitude	Date Formed	Eruptive Source
ML22-27	19.46383	-155.60018	11/27/2022	Moku'āweoweo
ML22-87	19.51449	-155.53148	12/10/2022	NERZ
ML22-88	19.44689	-155.60941	11/27/2022	South Caldera
ML22-89	19.44273	-155.61183	11/27/2022	South Caldera

Note(s): Latitude and longitude are in the WGS84 reference system. NERZ = Northeast Rift Zone.

Electron probe micro-analysis (EPMA)

Olivine and enstatite core-to-rim traverses and basaltic glasses were measured using a five-spectrometer JEOL Hyperprobe JXA-8530F+ at U.S. Geological Survey (USGS) lab in Menlo Park, California (Any use of trade, firm, or product names is for descriptive purposes only and does not imply endorsement by the U.S. Government). Olivine and enstatite were analyzed using a 20 kV accelerating voltage and a 1 µm beam with a 40 nA current. Counting times for olivine analyses were 20 s for Si, Mg and Fe, 40 s for Ca and Mn, and 60 s for Ni on the peak, with half the peak counting times on each side of the peak for backgrounds. San Carlos olivine (USNM 111312/444[15]) was measured regularly throughout the analyses to monitor for instrumental drift. Standards were San Carlos olivine for Si, Fe, and Mg, a synthetic nickel-oxide for Ni, Mn_2O_3 for Mn, and Wollastonite for Ca. Two-sigma relative precision for olivine, based on repeated analyses of San Carlos olivine, are 0.66 wt. % for SiO_2, 0.80 wt. % for MgO, 0.08 wt. % for FeO, and 0.01 wt. % for MnO, NiO, and CaO (Supplementary Data). Two-sigma relative

precision of Fo is 0.16 mol%. Enstatite standards were San Carlos olivine for Si, Fe, and Mg, Mn_2O_3 for Mn, Wollastonite for Ca, Kakanui Pyrope Garnet (USNM 143968[15]) for Al and Ti, Tiburon Albite for Na, and Cr_2O_3 for Cr. Two-sigma relative precision for pyroxene, based on repeated analyses of Clinopyroxene PSU (B-35A), are 0.24 wt. % for SiO_2, 0.23 wt. % for MgO, 0.04 wt. % for FeO, 0.16 wt. % for CaO, 0.007 wt. % for Al_2O_3, and 0.003 wt. % for MnO, Na_2O, and Cr_2O_3 (Supplementary Data).

Fluid inclusion olivine hosts were also measured using the USGS Menlo Park microprobe with the same conditions and peak counting times as the olivine core-to-rim traverses. San Carlos olivine (USNM 111312/444[15]) was measured regularly throughout the analyses to monitor for instrumental drift. Standards were Synthetic Fayalite for Si and Fe, San Carlos olivine for Mg, a synthetic nickel-oxide for Ni, Mn_2O_3 for Mn, and Wollastonite for Ca. Two-sigma relative precision, based on repeated analyses of San Carlos olivine, are 0.38 wt. % for SiO_2, 0.67 wt. % for MgO, 0.07 wt. % for FeO, 0.01 wt. % for NiO, 0.005 wt. % MnO, and 0.003 wt% for CaO (Supplementary Data). Two-sigma relative precision of Fo is 0.17 mol%.

Basaltic glasses were analyzed using a 15 kV accelerating voltage and a 10 µm beam with a 10-20 nA current. Counting times for glass analyses were 20 s for Si, Ca, Fe, Al, and Mg, 10 s for Na and K, 40 s for Ti, P, and Mn, and 45 s for S, Cl, and F on the peak with half the peak counting times on each side of the peak for backgrounds. VG2 Basaltic Glass (USNM 111240/52[15]) was measured regularly throughout the analyses to monitor for instrumental drift. Standards were VG2 Basaltic Glass (USNM 111240/52[15]) for Si, Al, and Mg, Tiburon Albite for Na, Crystal Bay Bytownite for Ca, Orthoclase OR-1A for K, Kakanui Pyrope Garnet (USNM 143968[15]) for Fe, TiO_2 for Ti, Wilburforce Apatite USGS-M105731 for P, Mn_2O_3 for Mn, Barite for S, Sodalite for Cl, and MgF_2 for F. Two-sigma relative precision, based on repeated analyses of VG2 Basaltic Glass, are 1.13 wt. % for SiO_2, 0.06 wt. % for TiO_2, 0.28wt. % for Al_2O_3, 0.26 wt. % for FeO, 0.02 wt.% for MnO 0.08 wt. % for MgO, 0.40 wt. % for CaO, 0.12 wt. % for Na_2O, 0.02 wt.% for K_2O and P_2O_5, 0.09 wt. % for SO_3, 0.004 wt. % for Cl, and 0.03 wt. % for F (Supplementary Data).

Electron backscatter diffraction (EBSD)

Olivine and enstatite orientations, necessary for addressing diffusion anisotropy, were determined using a TESCAN Vega3 SEM with Oxford Symmetry electron backscatter detector

(EBSD) at the USGS lab in Menlo Park, California (mounts ORI-1, ORI-2, ORI-3), and a FEI Quanta 450 Field Emission SEM with Oxford Symmetry EBSD at the USGS lab in Denver, Colorado (mount ORI-4). Measurements were taken using a 70° sample tilt, 15–20 kV accelerating voltage, and working distance between 17.5 and 20 mm. Area maps of olivine macrocrysts in the region where core-to-rim traverses were taken achieved mean angular deviation values of <1°. Orientations were plotted in lower equal-area (Schmidt) hemisphere stereonet projections (trend and plunge of axis positions available in Supplementary Data), and α, β, and γ, the angles between the traverse and the *a*-, *b*-, and *c*-axes of the olivine macrocrysts, respectively, were calculated using the *Stereonet* software[16].

Earthquake Catalog Descriptions

All catalogs were queried for earthquakes at all depths and magnitudes located in a box from 19.35ºN to 19.60ºN latitude and 155.52ºW to 155.75ºW longitude. The line bisecting the southwest and northeast corners of this box separates the earthquake epicenters into "NW Flank" (northwestern triangle) and "Summit" (southeastern triangle) for comparing the occurrence of seismicity in these two epicentral locations. See "Comparison of Earthquake Catalogues" discussion section below.

HVO/ComCat. The earthquakes in this catalog are from routine earthquake location efforts done by seismic analysts at the U.S. Geological Survey Hawaiian Volcano Observatory (HVO). Earthquake locations and magnitudes reviewed by these analysts are then forwarded to the Advanced National Seismic Systems Comprehensive Catalog (ANSS ComCat) hosted by the USGS Earthquake Hazards Program[17]. This catalog spans 1970 to present day and was last queried for this study on January 3, 2024.

Matoza. The earthquakes in this catalog are taken from the HVO catalog for the years 1986 to 2018 across the entire island and then systematically relocated using waveform cross-correlation with a 1-D velocity model. As a result of their original locations and depths being sourced from the HVO catalog, the depths in this catalog are, on average, the same as the HVO catalog but with less hypocentral spread. This catalog is available as a supplement of [18].

Wilding. This catalog is completely independent of the HVO catalog, and instead applies an automated, deep learning workflow to detect and locate earthquakes. Earthquake locations are

further refined with a similar cross-correlation approach to Matoza et al. (2020) but with a different 1-D velocity model and incorporation of station elevations in the travel-time calculations. It spans the period following the 2018 Kīlauea eruption to early 2022. No magnitude information is provided with this catalog. The catalog is available as a supplement of [19].

REDPy. Continuous waveform data from a subset of seismic stations were used to automatically create a more temporally complete, organized catalog using the open software REDPy[20]. This catalog spans the years 2012 to 2024 and groups triggered earthquakes by waveform similarity into "families" that share similar hypocenters and mechanisms. This catalog does not contain new location information and is instead associated with the HVO/ComCat catalog by event time to derive approximate locations from shared events. The outputs and technical details to reproduce it are included in [21].

Results

Analysis of ~80 air-quenched summit samples and air- or water-quenched molten samples collected almost daily from the Northeast Rift Zone were characterized by energy dispersive X-ray fluorescence in near-real-time to inform the eruption response[14]. All lava flow and tephra samples along ~19 km of fissures (Fig. S1) and 13 days of eruption time-series had uniform major- and minor-element compositions[14]. Olivine cores are primarily >Fo_{87}, and all are normally zoned toward $Fo_{78–82}$ rims (Fig. S3A, B). Core-to-rim Fe-Mg zoning was modeled (Fig. S3C) for 59 olivine crystals and two rare reversely zoned enstatite macrocrysts. The enstatite crystals have cores in equilibrium with the host melt (Supplementary Data, Fig. S4) and indicate timescales similar to the olivine dataset.

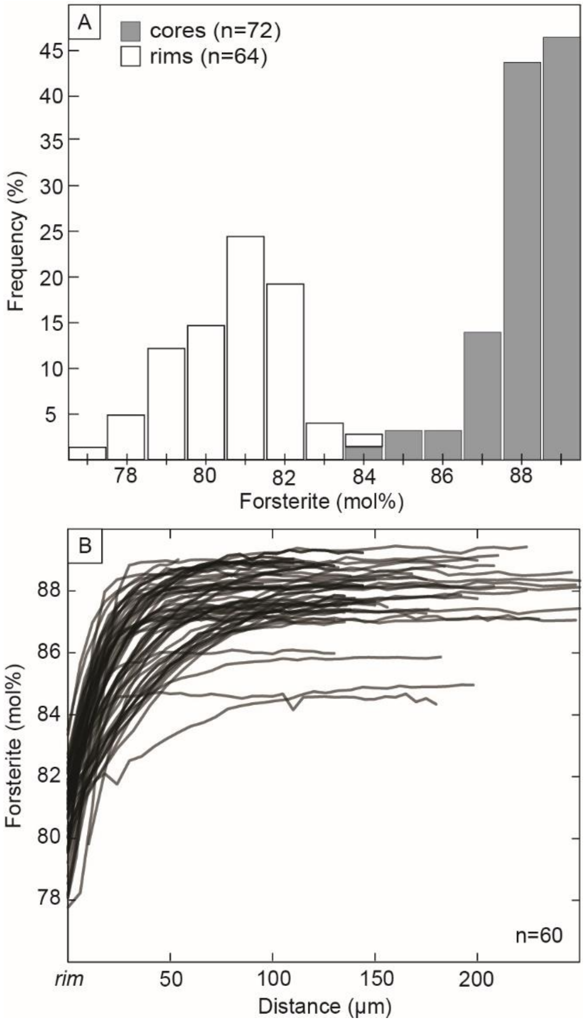

Fig. S3. Overview of olivine chemistry. (A) Histogram of olivine core (gray) and rim (white) compositions (Supplementary Data). Bins represent 1 mol. % (e.g., $Fo_{86.5}$–$Fo_{87.5}$). **(B)** Olivine rim (left side of figure) to core (right side) zoning profiles. All Mauna Loa 2022 olivine phenocrysts are normally zoned ($Fo_{core} > Fo_{rim}$).

Discussion

Mineral re-equilibration

Although none of the 2022 olivine or enstatite macrocrysts record intrusion in the upper reservoir (1.5-2 km depth) prior to 2022 (based on diffusion timescales), any intrusions to this level[26-29]) prior to 2022 might have been entirely homogenized and the macrocryst cargo erased (no low-Fo olivine were found; Fig. S3). Complete re-equilibration of Hawaiian olivine can occur rapidly, over timescales of a few months to years, depending on crystal size and the olivine and surrounding melt compositions[31-31]. Thus, the question of how efficiently recharge intruded the upper reservoir (number of intrusions, how quicky intrusions are homogenized) over the past years to decades remains to be explored.

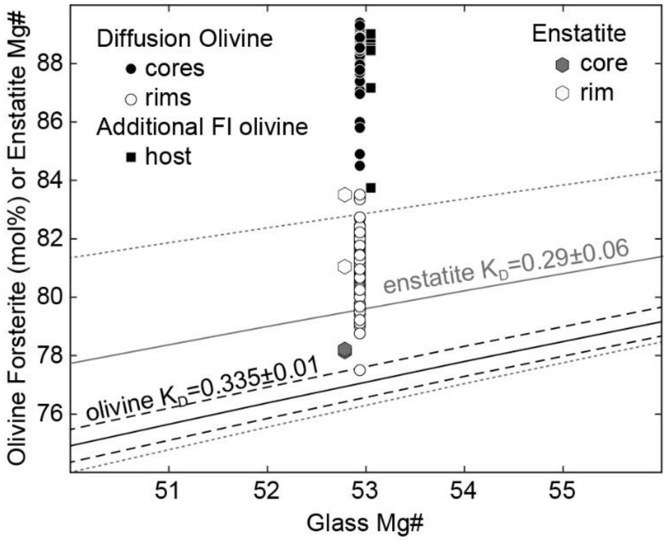

Fig. S4. Mineral-melt equilibrium. Glass Mg-number (Mg#=Mg/(Mg+Fe^{2+})×100 plotted against Mauna Loa 2022 olivine core (black circles) and rim (white circles) compositions from diffusion modeling crystals, host olivine for fluid inclusions (black squares), and enstatite core (gray hexagon) and rim (white hexagon) compositions. Data are slightly offset from each other for clarity. The black dashed lines mark the shallow pressure (1 atm) olivine equilibrium field (Fe-Mg K_D = 0.335 ± 0.01)[22]. The gray dashed lines mark the orthopyroxene equilibrium field (Fe-Mg K_D = 0.29 ± 0.06)[23]. Mg# is calculated assuming $Fe^{3+}/\Sigma Fe$ = 0.16 based on recent μ-XANES analyses of Mauna Loa olivine-hosted melt inclusions[24], similar to Kīlauea[25]. An Mg# of 0.53 was used to represent the highest MgO glass measured from 2022 samples (Supplementary Data). All other glass MgO (and calculated Mg#'s) are lower, and thus mineral compositions would be further out of equilibrium.

Alternating Summit and northwest flank earthquake swarms

Pre-eruptive seismicity can be divided into groups based on hypocentral locations as occurring beneath the northwest flank (NWF) or summit (Sum; Fig. 3 in main text). NWF earthquakes occur above the dashed line in Fig. S5 and summit earthquakes below. NWF earthquakes also extend to greater depths (down to 8 km) compared to shallower summit earthquakes (Fig. S6).

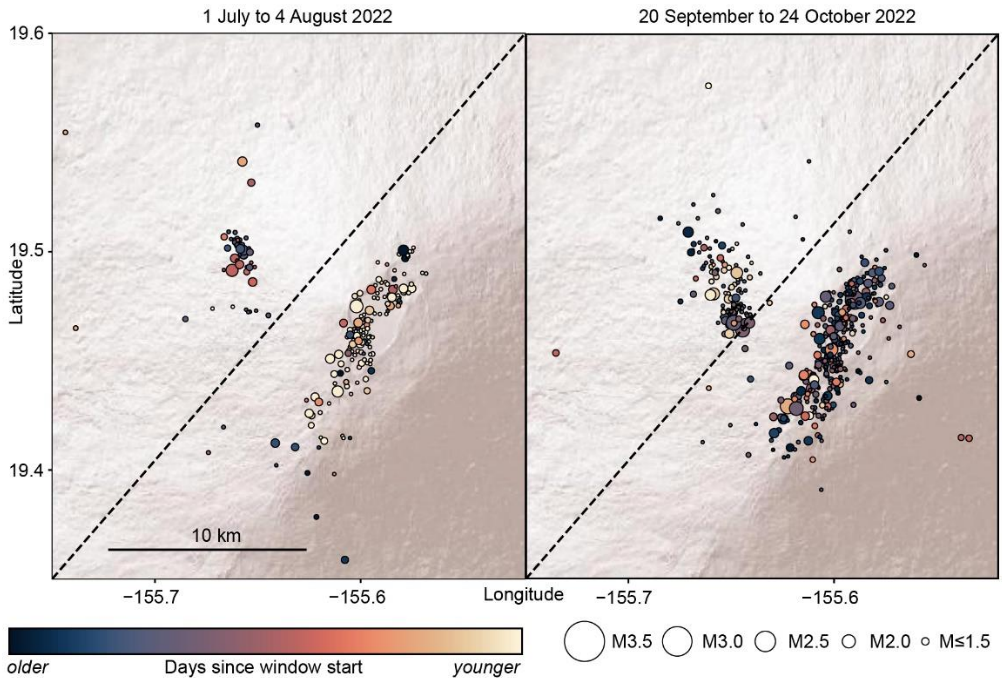

Fig. S5. Northwest Flank versus Summit earthquake hypocenters. Earthquake hypocenters at Mauna Loa's summit comparing periods of alternating northwest flank and summit (1 July to 4 August 2022) versus period of synchronous activity from 20 September to the onset of eruption on 27 November 2022. Dashed line represents northwest flank versus summit designation. Earthquakes are color coded by relative time within each figure, with cooler colors representing older events and warmer colors representing younger events. Symbol size corresponds to earthquake magnitude. Data are from [17].

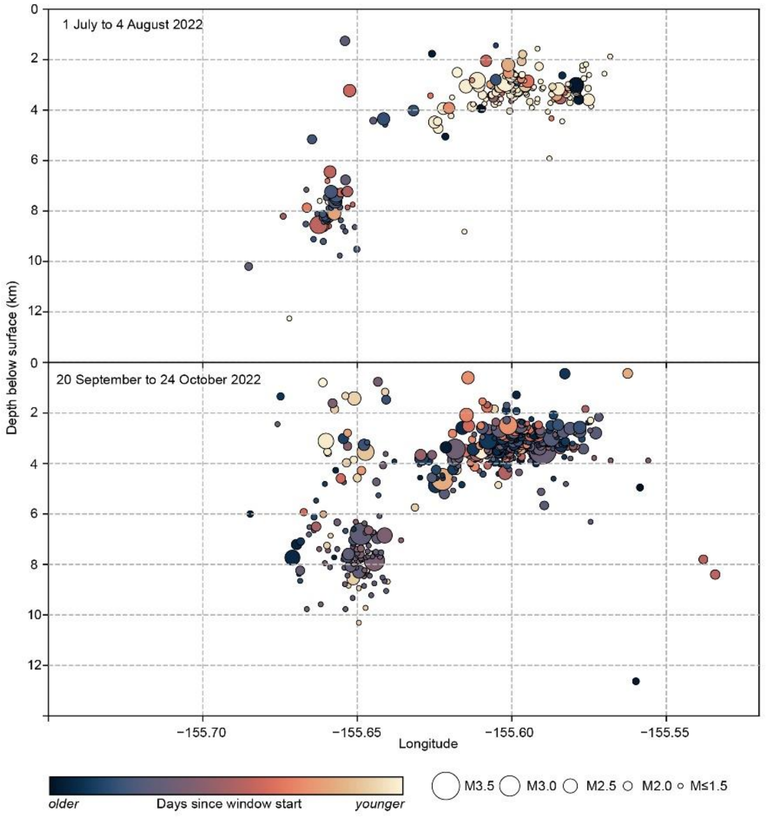

Fig. S6. Companion figure to Fig. S5 (previous). Earthquake hypocenters at Mauna Loa's summit plotted as a function of depth comparing periods of alternating northwest flank and summit (top panel; 1 July to 4 August 2022) versus period of synchronous activity from 20 September to the onset of eruption on 27 November 2022 (bottom panel). Northwest flank earthquakes are between -155.68 and -155.64 degrees, whereas earthquakes under the summit caldera are located at > -155.64 degrees. Earthquakes are color coded by relative time within each figure, with cooler colors representing older events and warmer colors representing younger events. Symbol size corresponds to earthquake magnitude. Data are from [17].

Older histories of recharge and intrusion

Some 2022 olivine crystals have subtle reverse zoning in their high-Fo interiors that reflect a history of recharge and intrusion that pre-dates the final zoning of the rims to lower Fo (Fig. S7). These interior reverse zonation patterns are subtle and not reliable for modeling re-equilibration timescales, but they are outside of analytical error and reflect an older history of recharge and intrusion.

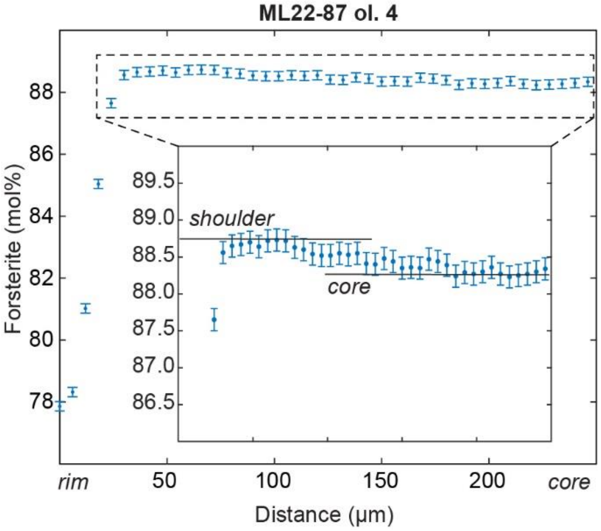

Fig. S7. Evidence for older recharge histories. Example of a Mauna Loa 2022 olivine (ML22-87, Northeast Rift Zone) with subtle reverse zoning just outside of analytical error in the high-Fo interior, consistent with a history of dissolution-recrystallization in a reservoir deeper than where the low-Fo rim zoning occurred. Fo uncertainty of ±0.18 mol% from Supplementary Data.

Fluid inclusion re-equilibration

Fluid inclusions reflect entrapment conditions only if they satisfy "Roedder's rules"[32]. That is, 1) a single homogenous fluid phase must have been trapped, 2) the fluid inclusion must retain its volume after entrapment, and 3) nothing must be added or removed after entrapment[32-33]. As the host crystal ascends in the magma plumbing system, pressure gradients will arise between entrapped inclusions and the host exterior. These gradients result in volumetric re-equilibration of fluid inclusions via brittle deformation (decrepitation) or plastic deformation of the crystal host (stretching)[33-40]. The result is that fluid inclusions generally reflect only minimum entrapment pressures, particularly when they were originally entrapped at high pressure (>10 km depth). Higher initial internal pressures, and larger inclusions, result in faster and more significant re-equilibration at various levels of ascent.

To assess the reliability of fluid inclusions as magmatic storage tracers, we modeled fluid-inclusion re-equilibration using the Python tool RelaxiFI (v0.0.5)[41]. We model three different scenarios (Fig. S8 a-c): 1) Fluid-inclusions with radii of 1 and 20 μm are captured in olivine at 4 km depth (1300˚ C, 99 MPa), then moved to the upper reservoir (1175˚ C, 23 MPa) where they stall for 2 months prior to eruption and quenching (Fig. S8a); 2) Fluid inclusions are captured in olivine in the upper reservoir (~1.5 km, 1175˚ C, 23 MPa), then erupted and slowly quenched over 0-7 days (Fig. S8b); 3) Fluid inclusions are captured in olivine at 8 km depth

(1350˚ C, 216 MPa), then moved to the intermediate reservoir (~4 km, 1300˚ C, 99 MPa) and stall for 0-10 years (Fig. S8c). Scenarios 1 and 2 do not produce any significant change in the pressure of the fluid inclusions (the change is smaller than the uncertainty of the method, and less than 2% in both cases); therefore, the effect of re-equilibration on fluid inclusions trapped in either the intermediate or upper reservoirs is minimal, and both reservoirs should easily be distinguishable in the distribution of fluid inclusions. Scenario 3 produces a significant change in the inclusion pressures at long timescales (up to ~45%). This is expected, as the pressure differential is larger for inclusions trapped at higher pressures and stored at high temperatures and lower pressures – here 4 km. We note that although this pressure change is large, it is likely to be overestimated by the model, which is very sensitive to storage temperature, inclusion size and distance from edges or cracks. Smaller inclusions (typical of this dataset with radii <10 µm), far from crystal edges and cracks tend to preserve pressure better than others.

In addition, it is common for fluid inclusions at other localities (i.e., Fogo in Cape Verde[42-44], Cumbre Vieja, Canary Islands[45]) to record much greater storage depths (30, 25, 18, 10 km) than those at Mauna Loa, despite undergoing re-equilibration. This indicates that although re-equilibration is an important process for fluid-inclusions captured at high pressures and stored at high temperatures, distributions corresponding to different storage regions can still be distinguished, and it is therefore unlikely that they are completely overprinted. In such a case, and if a deeper Mauna Loa reservoir (for example 8 km) had contributed to the fluid-inclusion distribution reported here, we would expect an asymmetric trailing distribution, with more fluid inclusions recording depths between 4-8 km. As this is not the case, we consider that the fluid inclusions presented here are highly unlikely to have been captured in deeper reservoirs.

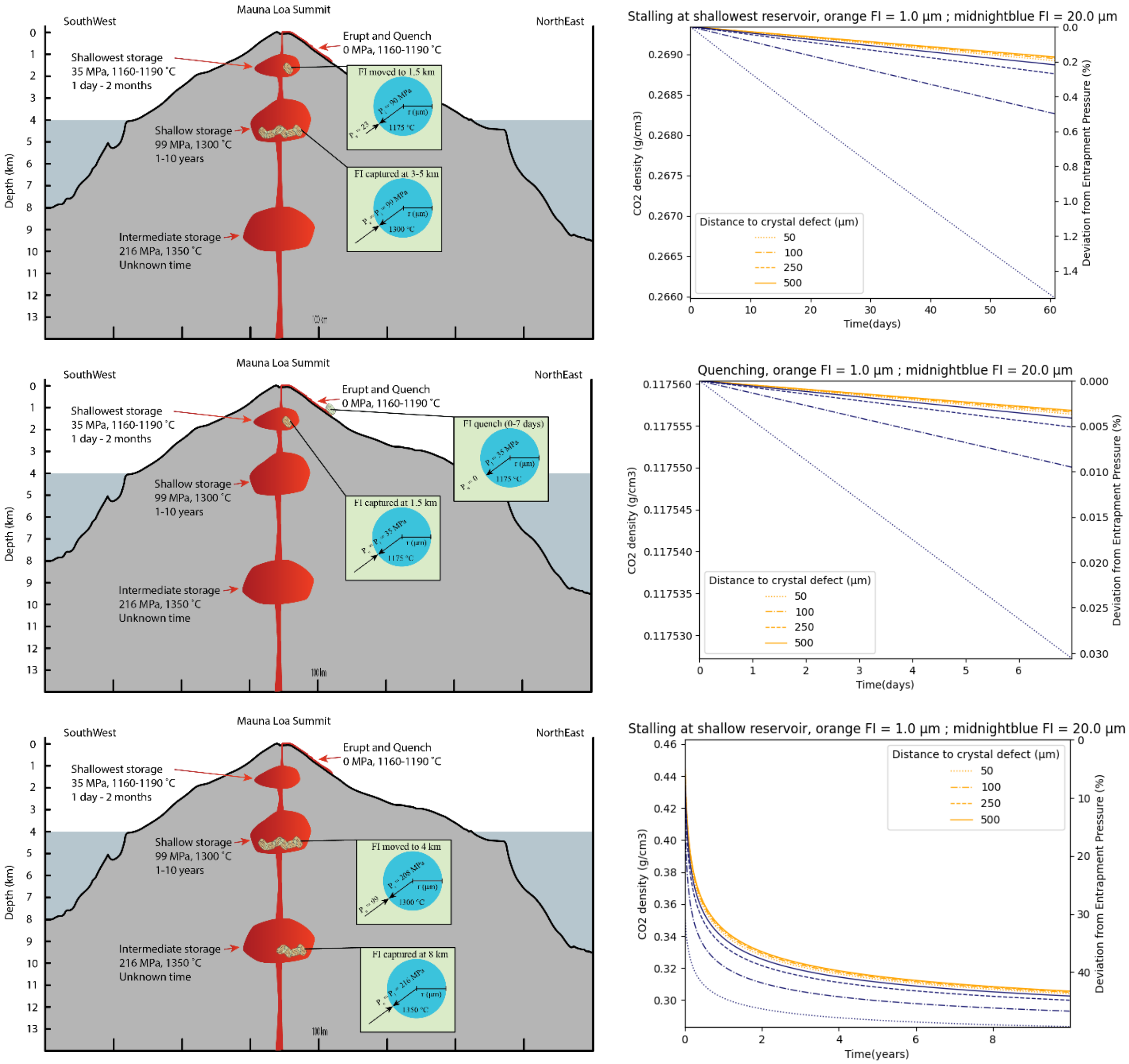

Fig. S8. Mauna Loa fluid inclusion (FI) re-equilibration models. a; top row) FIs are trapped at 4 km, the moved to upper reservoir in September 2022 where they stalled for 2 months (does not include quenching but quenching has an even smaller effect than that modeled here). The change in FI barometry is smaller than the uncertainty of the method. **b; middle row)** FIs captured at 1.5 km, erupted and quenched in 0-7 days. The change in FI barometry is smaller than the uncertainty of the method. **c; bottom row)** FIs captured at 8 km, stalled at 4 km for 0-10 years. Appreciable re-equilibration of FIs occurs, but is not seen in the natural dataset.

Comparison of Earthquake Catalogues

We elect to use the HVO/Comcat earthquake catalogue in the main text for comparison with the olivine barometry but there are also relocated earthquake catalogues from [18] and [19] (Fig. S9). There are significant differences between the relocated catalogues and the HVO catalogue. The HVO catalogue shows diffuse seismicity between 1 and 5 km, centered at 3 km below the

surface. Relative relocations of these earthquakes[18-19] condense the depth range, but the absolute depths of these earthquakes are uncertain (e.g., due to uncertainties in the velocity model in addition to timing uncertainties, but also that depth is necessarily the least constrained free parameter because seismometers must exist on the surface or close to it).

Matoza's[18] catalog condenses the earthquakes to a similar depth centroid beneath the summit because the locations prior to relocation are at those depths (i.e., from the HVO catalog[17]). This catalogue also does not represent the NW flank earthquakes well. Wilding's[19] catalog is independent of the HVO catalog[17] and systematically locates the summit seismicity ~3 km deeper, whereas the flank seismicity is at the same depths, and this is confirmed for earthquakes shared between the two catalogs. We prefer the HVO catalogue[17] because both NW flank and summit earthquakes are well represented, it spans the entire time period of interest, and the Wilding[19] catalogue was designed to focus on seismicity at much greater depths than considered here.

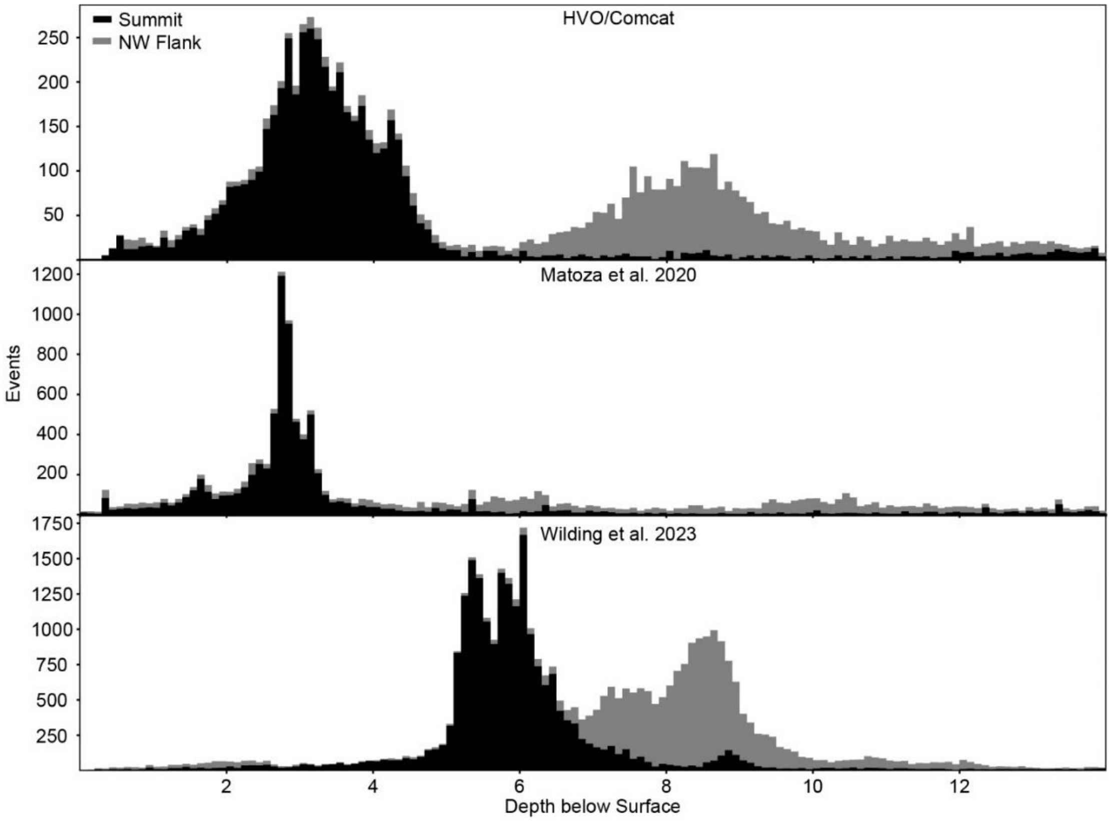

Fig. S9. Comparison of HVO/Comcat[17] (also shown in Fig. 4 in the main text), [18] (relocated), and [19] (relocated) earthquake catalogues for Mauna Loa. Data are all events occurring between the latitudes of 19.35ºN and 19.6ºN, and longitudes of 155.75ºW and 155.52°W. We define a line connecting the northeast and southwest corners of the location query box defined earlier, and earthquakes below (i.e., to the southeast of) this line are defined as "Summit" and above/northwest are defined as "Northwest Flank" earthquakes.

Olivine Fe-Mg diffusion coefficients

Recently, [46] suggested current olivine Fe-Mg diffusion coefficients may be ~10× too slow. If Mauna Loa 2022 olivine timescales are reduced by a factor of 10×, then most timescales would record magma movement <1 week prior to eruption, when there are no correlations to geophysical monitoring datasets (Fig. S9). This would imply that the entire intrusion that led to the eruption was aseismic and did not generate discernable changes in deformation signals. This seems geologically unreasonable, and we thus prefer the classically used diffusion coefficient presented in [47].

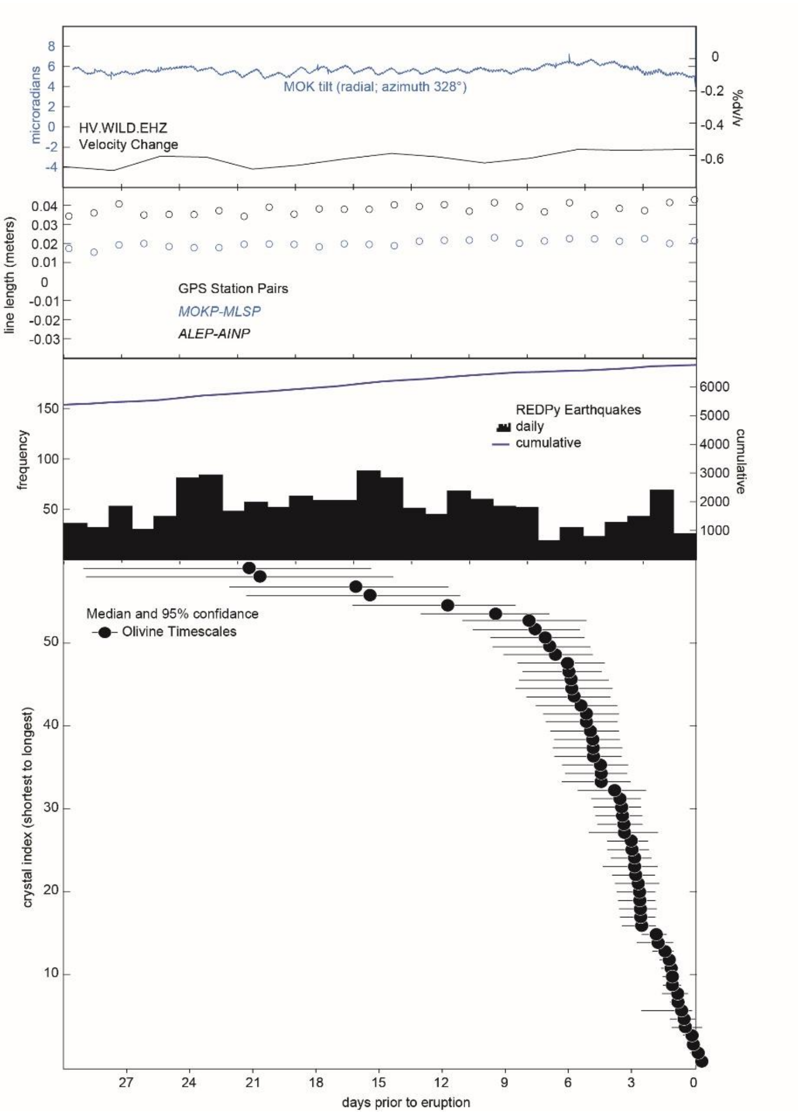

Fig. S10. Olivine diffusion timescales recalculated using [46].

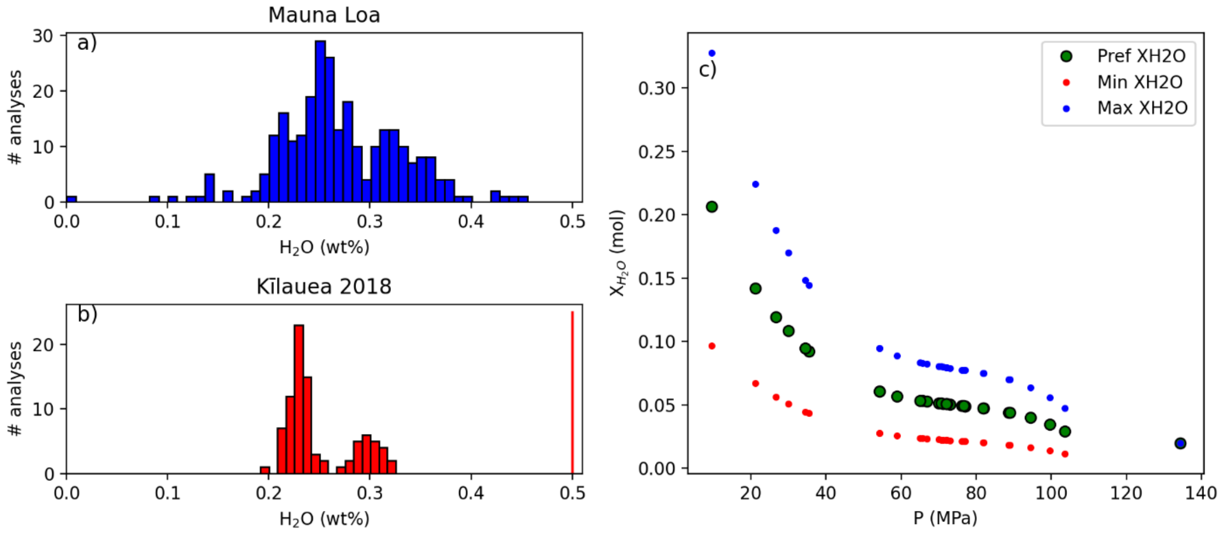

Fig. S11. Comparison of melt inclusion H_2O contents. (A) Mauna Loa[48] (), and **(B)** the melt inclusions from the 2018 eruption of Kīlauea used to construct the XH2O-P trends in [41]. **(C)** XH_2O values calculated at the point of vapor saturation. The red dots show XH_2O values calculated from measured melt inclusion H_2O contents (inferred to have undergone H^+ loss), the blue dots for a fixed melt water content of 0.5 wt% (a reasonable upper limit at both Mauna Loa and Kīlauea). The green dots show the average of these two calculations – we propagate the uncertainty spanning the red to blue dots when calculating pressures.

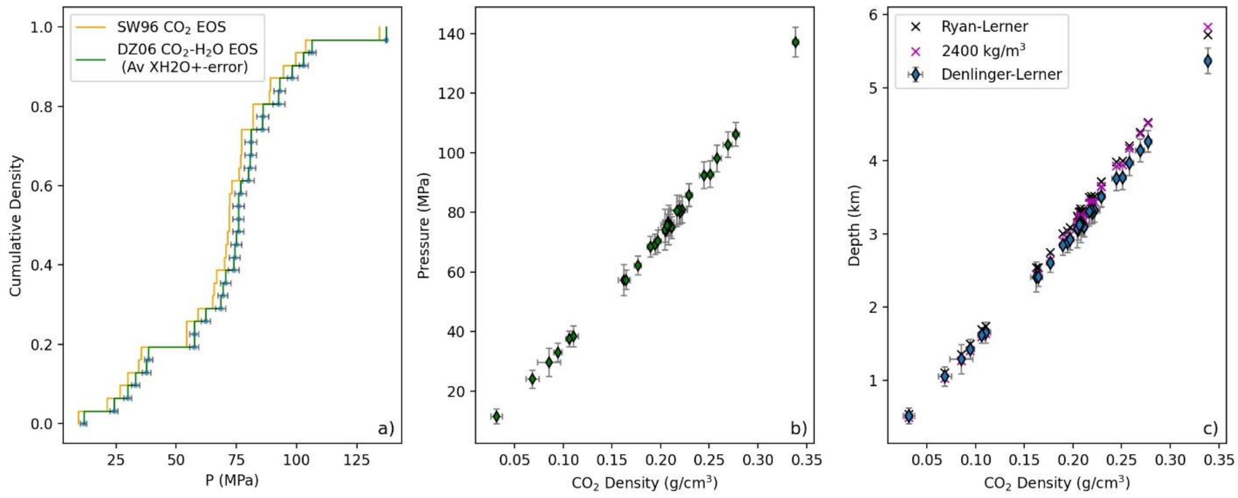

Fig. S12. Visualization of the effect of XH_2O and other sources of uncertainty on calculated pressures and depths. a) Cumulative density function showing pressures calculated using the pure CO_2 EOS[49] vs. the mixed CO_2-H_2O EOS[50] (XH2O from the green datap in Fig. S10). The error bar shows the uncertainty in pressure from using the min and max XH_2O values indicated from melt inclusion data. **b)** Propagated uncertainties in CO_2 density (from peak fitting, drift correction, and densimeter fitting) vs. uncertainty in the calculated pressure (from CO_2 density, uncertainty in entrapment temperature, and XH_2O). Pressures and errors were translated into depth space using the density-depth model [51] and [52] and assuming a constant density of 2400 kg/m^3 are shown for comparison (error bars will be of similar magnitude).

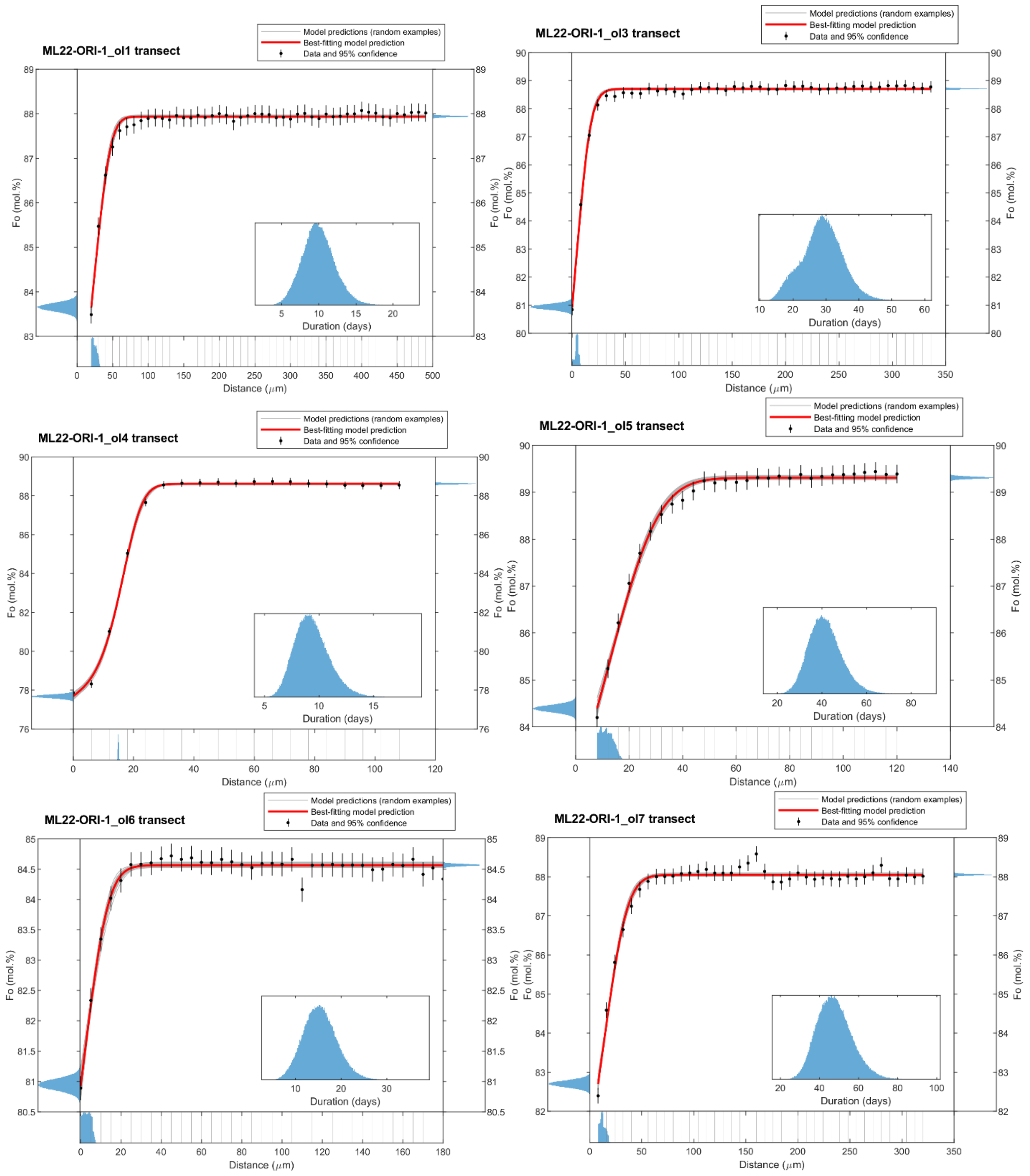
ML22-ORI-1_ol1 transect
ML22-ORI-1_ol3 transect
ML22-ORI-1_ol4 transect
ML22-ORI-1_ol5 transect
ML22-ORI-1_ol6 transect
ML22-ORI-1_ol7 transect
Model predictions (random examples)
Best-fitting model prediction
Data and 95% confidence
Fo (mol.%)
Distance (μm)
Duration (days)

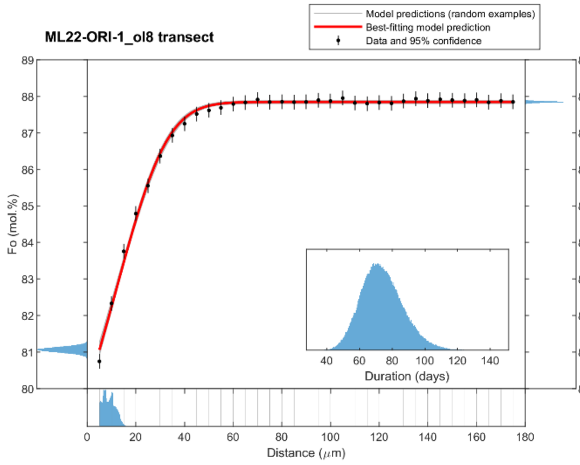
ML22-ORI-1_ol8 transect
Model predictions (random examples)
Best-fitting model prediction
Data and 95% confidence
Fo (mol.%)
Duration (days)
Distance (μm)

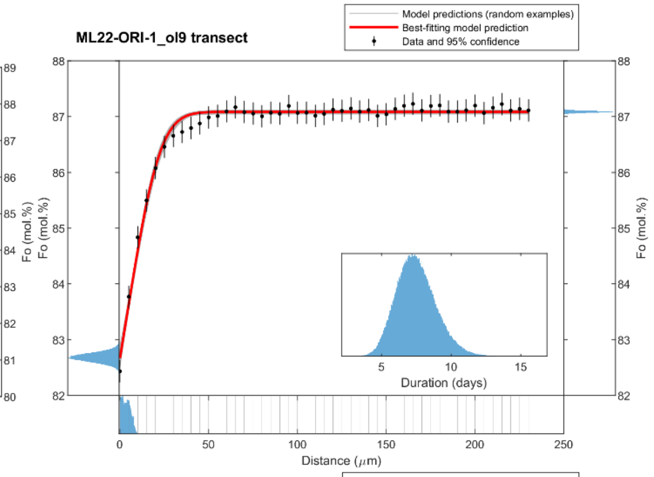
ML22-ORI-1_ol9 transect
Model predictions (random examples)
Best-fitting model prediction
Data and 95% confidence
Fo (mol.%)
Duration (days)
Distance (μm)

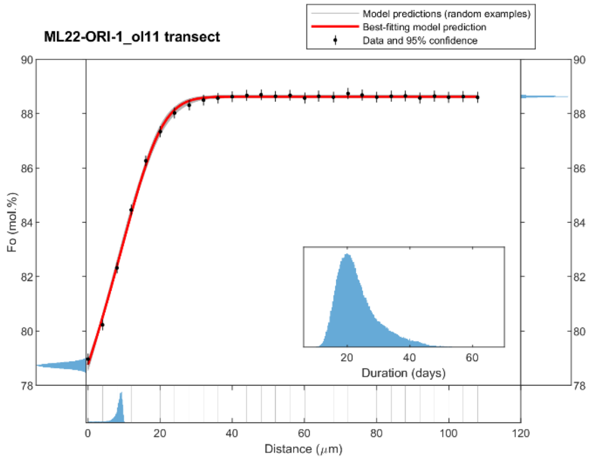
ML22-ORI-1_ol11 transect
Model predictions (random examples)
Best-fitting model prediction
Data and 95% confidence
Fo (mol.%)
Duration (days)
Distance (μm)

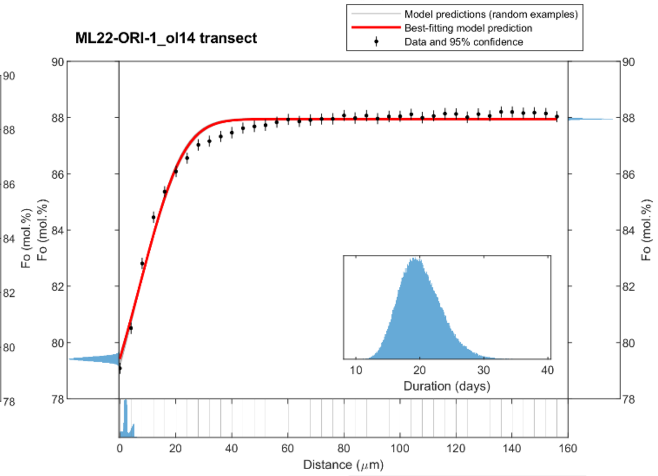
ML22-ORI-1_ol14 transect
Model predictions (random examples)
Best-fitting model prediction
Data and 95% confidence
Fo (mol.%)
Duration (days)
Distance (μm)

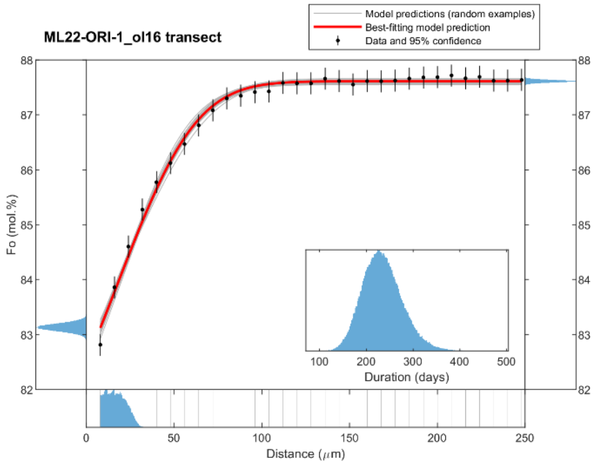
ML22-ORI-1_ol16 transect
Model predictions (random examples)
Best-fitting model prediction
Data and 95% confidence
Fo (mol.%)
Duration (days)
Distance (μm)

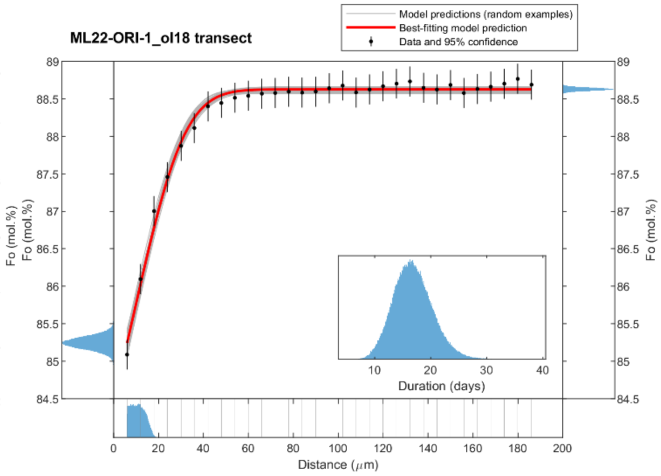
ML22-ORI-1_ol18 transect
Model predictions (random examples)
Best-fitting model prediction
Data and 95% confidence
Fo (mol.%)
Duration (days)
Distance (μm)

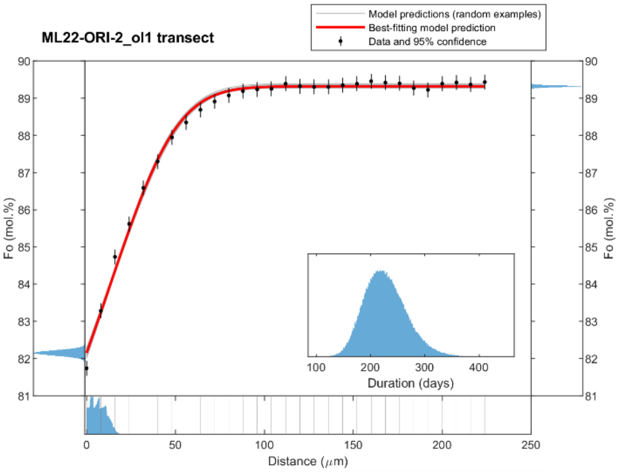
ML22-ORI-2_ol1 transect
Model predictions (random examples)
Best-fitting model prediction
Data and 95% confidence
Fo (mol.%)
Duration (days)
Distance (μm)

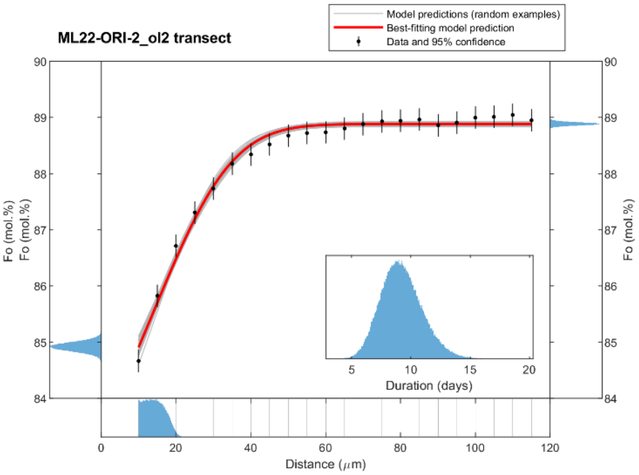
ML22-ORI-2_ol2 transect
Model predictions (random examples)
Best-fitting model prediction
Data and 95% confidence
Fo (mol.%)
Duration (days)
Distance (μm)

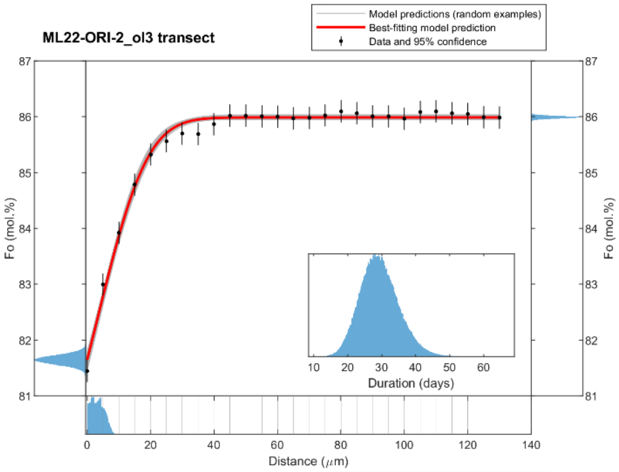
ML22-ORI-2_ol3 transect
Model predictions (random examples)
Best-fitting model prediction
Data and 95% confidence
Fo (mol.%)
Duration (days)
Distance (μm)

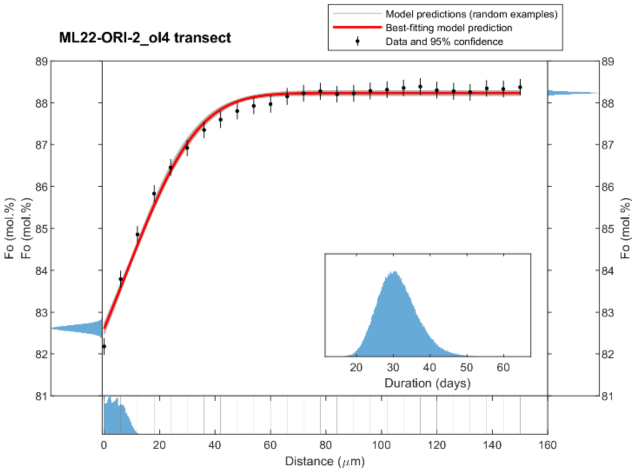
ML22-ORI-2_ol4 transect
Model predictions (random examples)
Best-fitting model prediction
Data and 95% confidence
Fo (mol.%)
Duration (days)
Distance (μm)

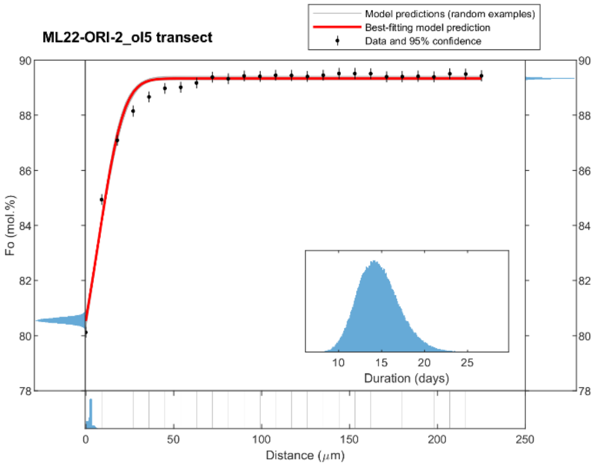
ML22-ORI-2_ol5 transect
Model predictions (random examples)
Best-fitting model prediction
Data and 95% confidence
Fo (mol.%)
Duration (days)
Distance (μm)

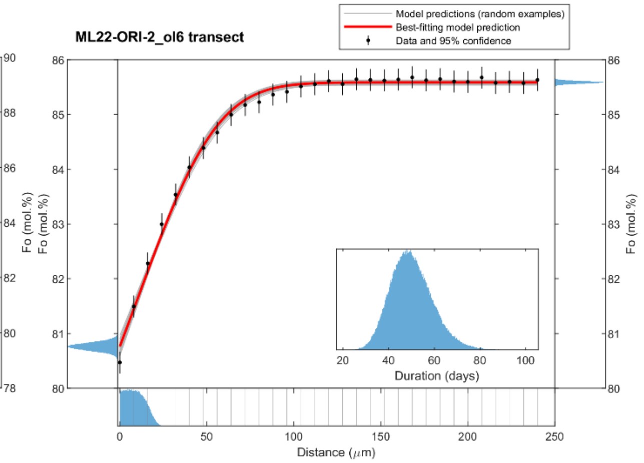
ML22-ORI-2_ol6 transect
Model predictions (random examples)
Best-fitting model prediction
Data and 95% confidence
Fo (mol.%)
Duration (days)
Distance (μm)

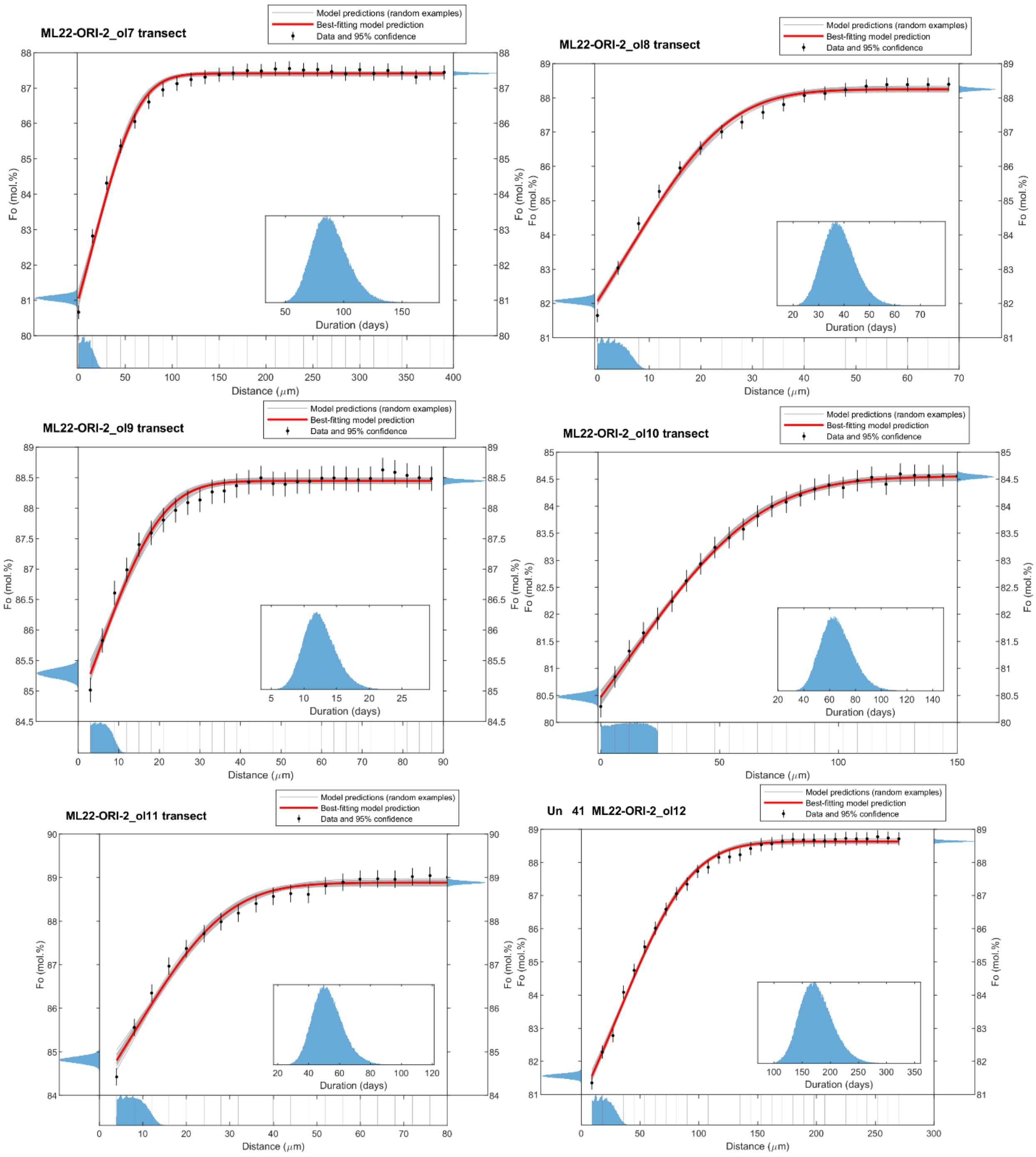

ML22-ORI-2_ol7 transect
ML22-ORI-2_ol8 transect
ML22-ORI-2_ol9 transect
ML22-ORI-2_ol10 transect
ML22-ORI-2_ol11 transect
Un 41 ML22-ORI-2_ol12
Model predictions (random examples)
Best-fitting model prediction
Data and 95% confidence
Fo (mol.%)
Duration (days)
Distance (μm)

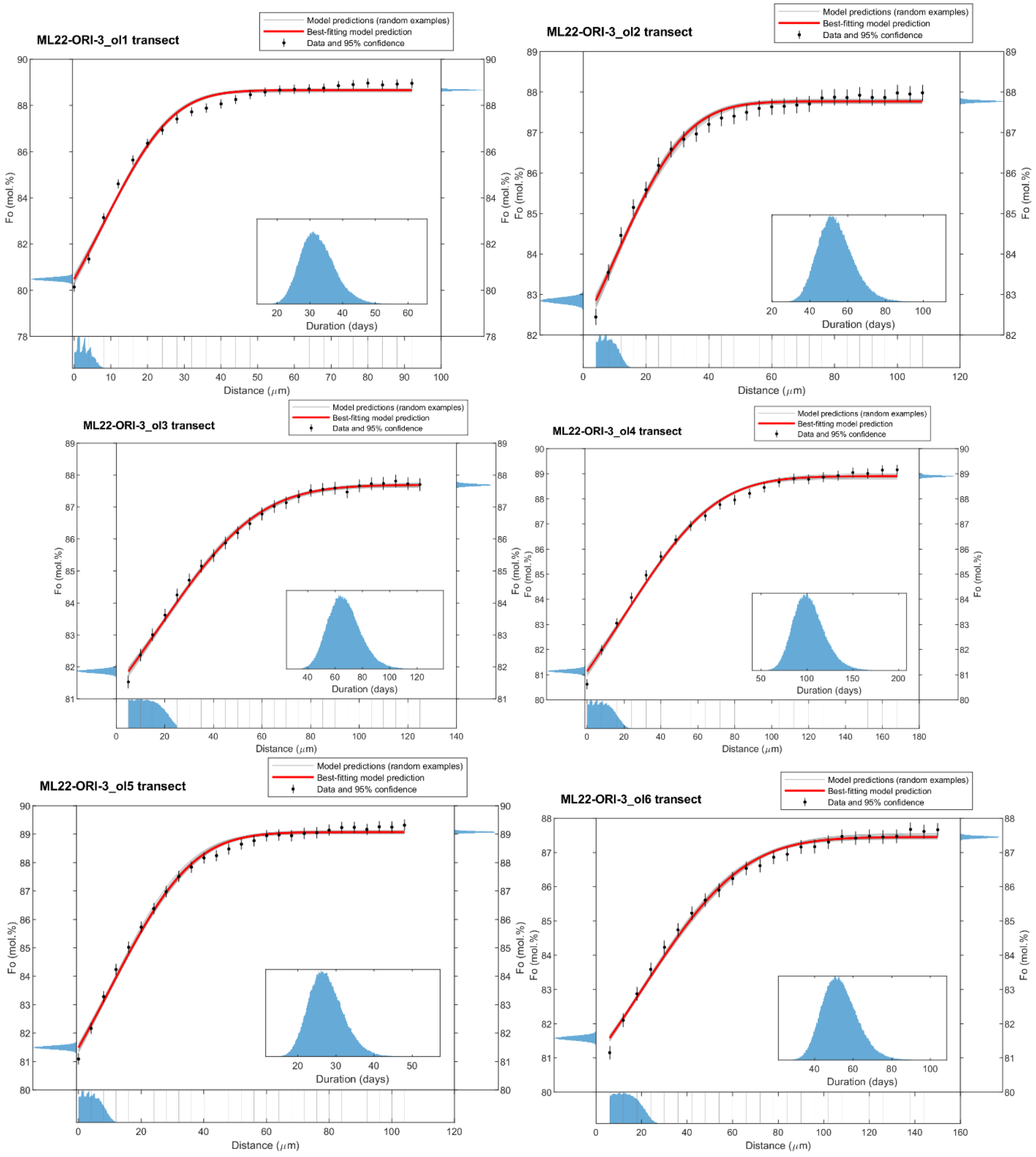
ML22-ORI-3_ol1 transect
ML22-ORI-3_ol2 transect
ML22-ORI-3_ol3 transect
ML22-ORI-3_ol4 transect
ML22-ORI-3_ol5 transect
ML22-ORI-3_ol6 transect
Model predictions (random examples)
Best-fitting model prediction
Data and 95% confidence
Fo (mol.%)
Duration (days)
Distance (μm)

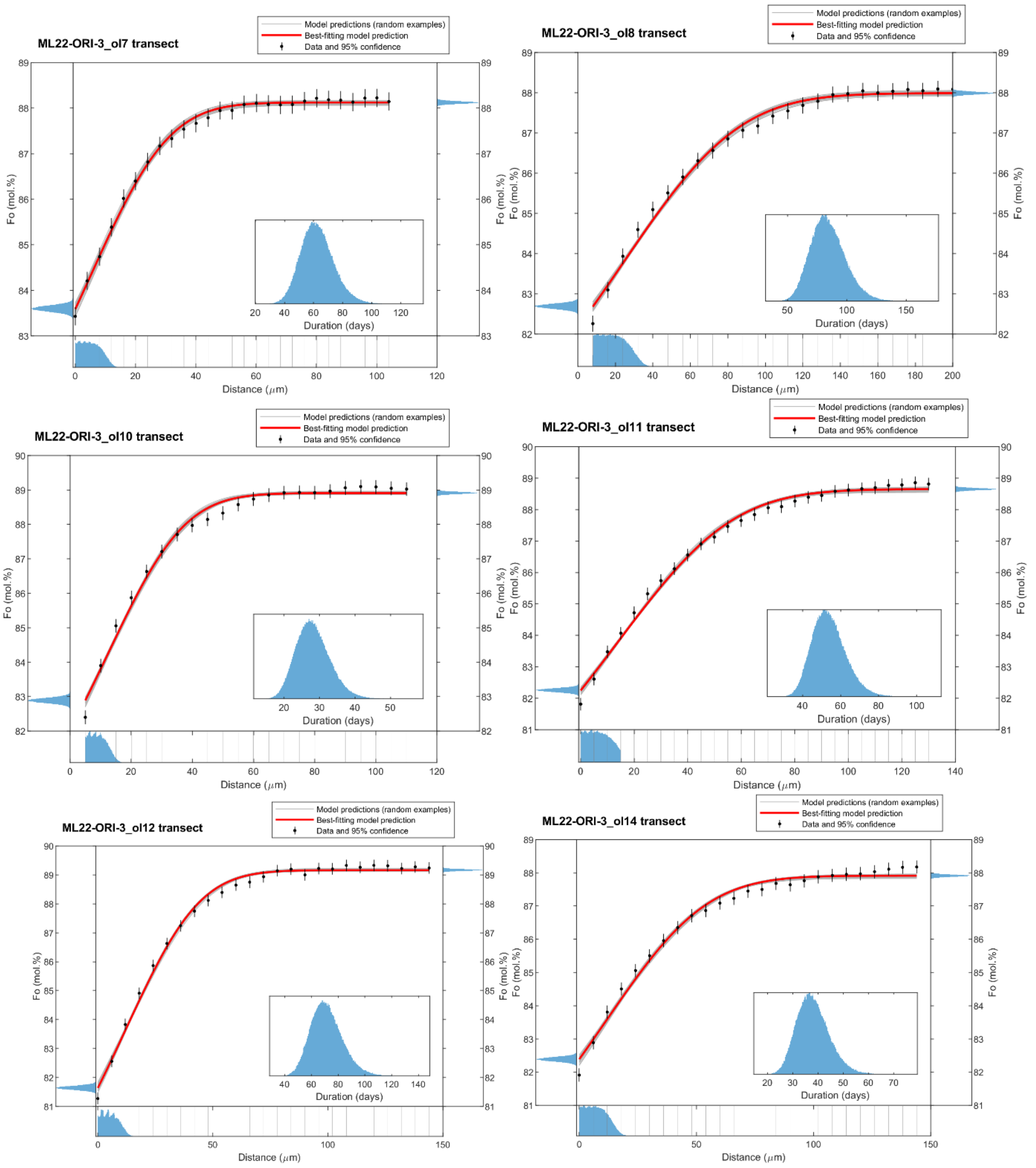
ML22-ORI-3_ol7 transect
Model predictions (random examples)
Best-fitting model prediction
Data and 95% confidence
Fo (mol.%)
Duration (days)
Distance (μm)
ML22-ORI-3_ol8 transect
Model predictions (random examples)
Best-fitting model prediction
Data and 95% confidence
Fo (mol.%)
Duration (days)
Distance (μm)
ML22-ORI-3_ol10 transect
Model predictions (random examples)
Best-fitting model prediction
Data and 95% confidence
Fo (mol.%)
Duration (days)
Distance (μm)
ML22-ORI-3_ol11 transect
Model predictions (random examples)
Best-fitting model prediction
Data and 95% confidence
Fo (mol.%)
Duration (days)
Distance (μm)
ML22-ORI-3_ol12 transect
Model predictions (random examples)
Best-fitting model prediction
Data and 95% confidence
Fo (mol.%)
Duration (days)
Distance (μm)
ML22-ORI-3_ol14 transect
Model predictions (random examples)
Best-fitting model prediction
Data and 95% confidence
Fo (mol.%)
Duration (days)
Distance (μm)

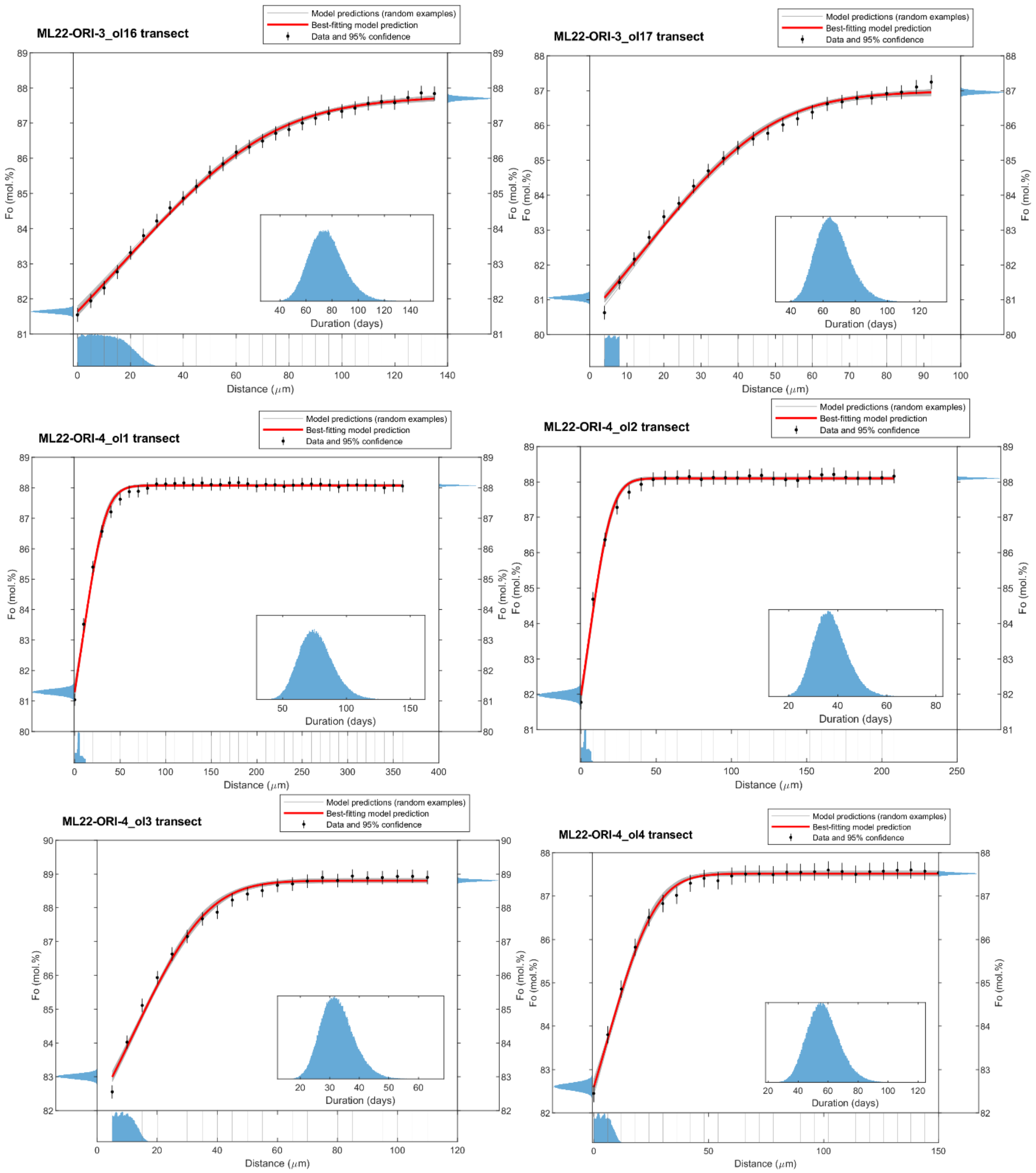
ML22-ORI-3_ol16 transect
ML22-ORI-3_ol17 transect
ML22-ORI-4_ol1 transect
ML22-ORI-4_ol2 transect
ML22-ORI-4_ol3 transect
ML22-ORI-4_ol4 transect
Model predictions (random examples)
Best-fitting model prediction
Data and 95% confidence
Fo (mol.%)
Duration (days)
Distance (μm)

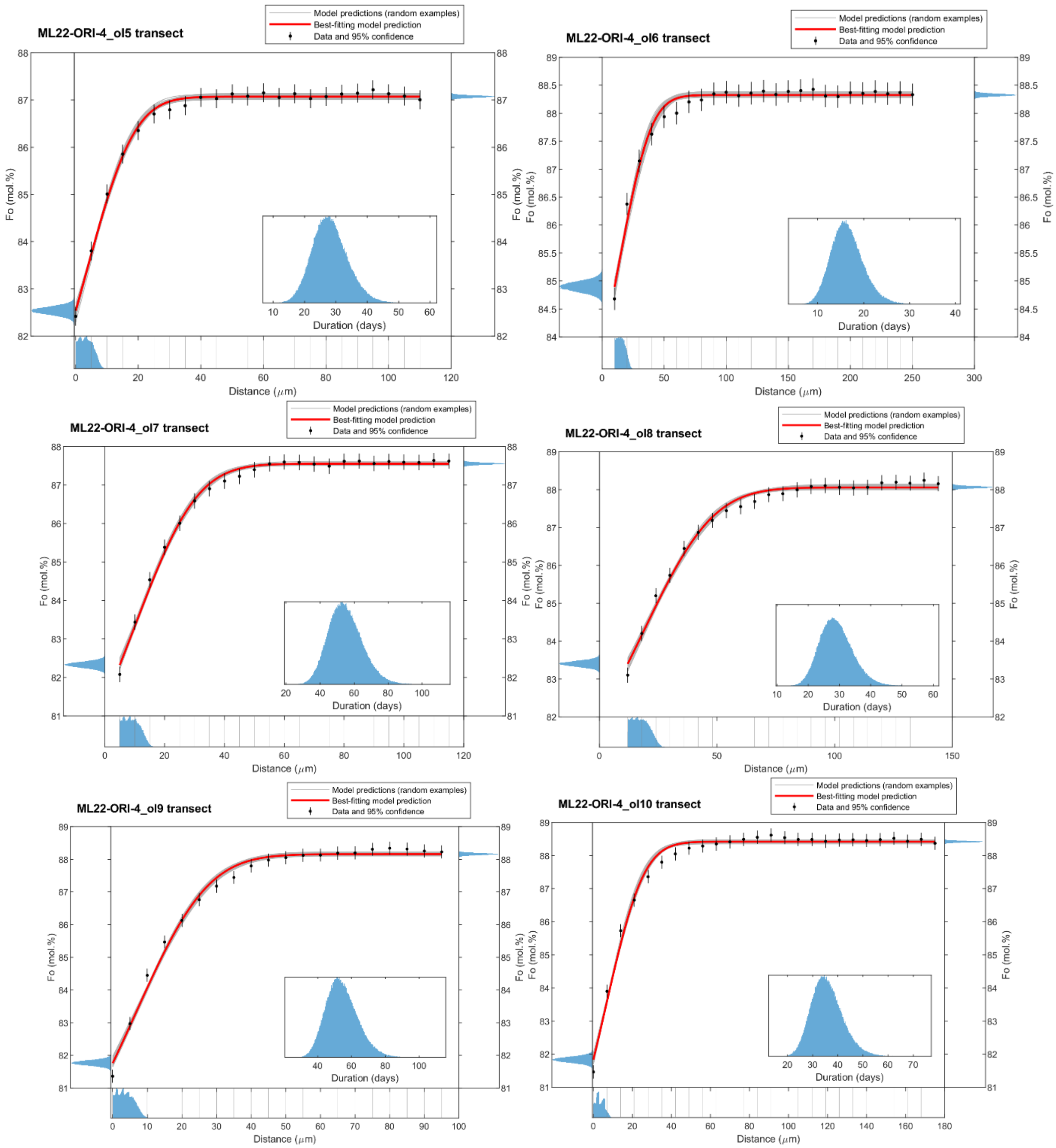
ML22-ORI-4_ol5 transect
ML22-ORI-4_ol6 transect
ML22-ORI-4_ol7 transect
ML22-ORI-4_ol8 transect
ML22-ORI-4_ol9 transect
ML22-ORI-4_ol10 transect
Model predictions (random examples)
Best-fitting model prediction
Data and 95% confidence
Fo (mol.%)
Duration (days)
Distance (μm)

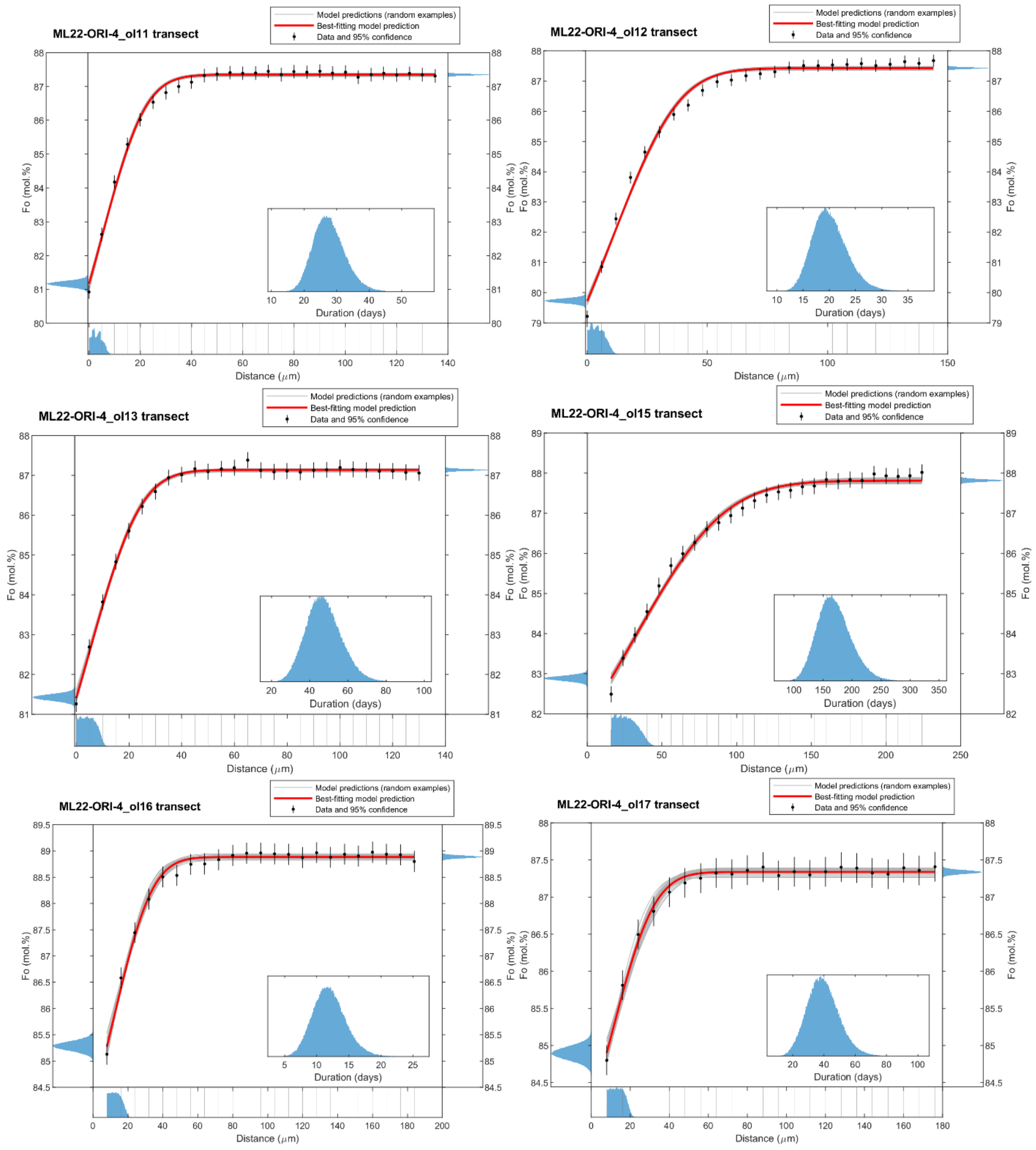
ML22-ORI-4_ol11 transect
ML22-ORI-4_ol12 transect
ML22-ORI-4_ol13 transect
ML22-ORI-4_ol15 transect
ML22-ORI-4_ol16 transect
ML22-ORI-4_ol17 transect
Model predictions (random examples)
Best-fitting model prediction
Data and 95% confidence
Fo (mol.%)
Duration (days)
Distance (μm)

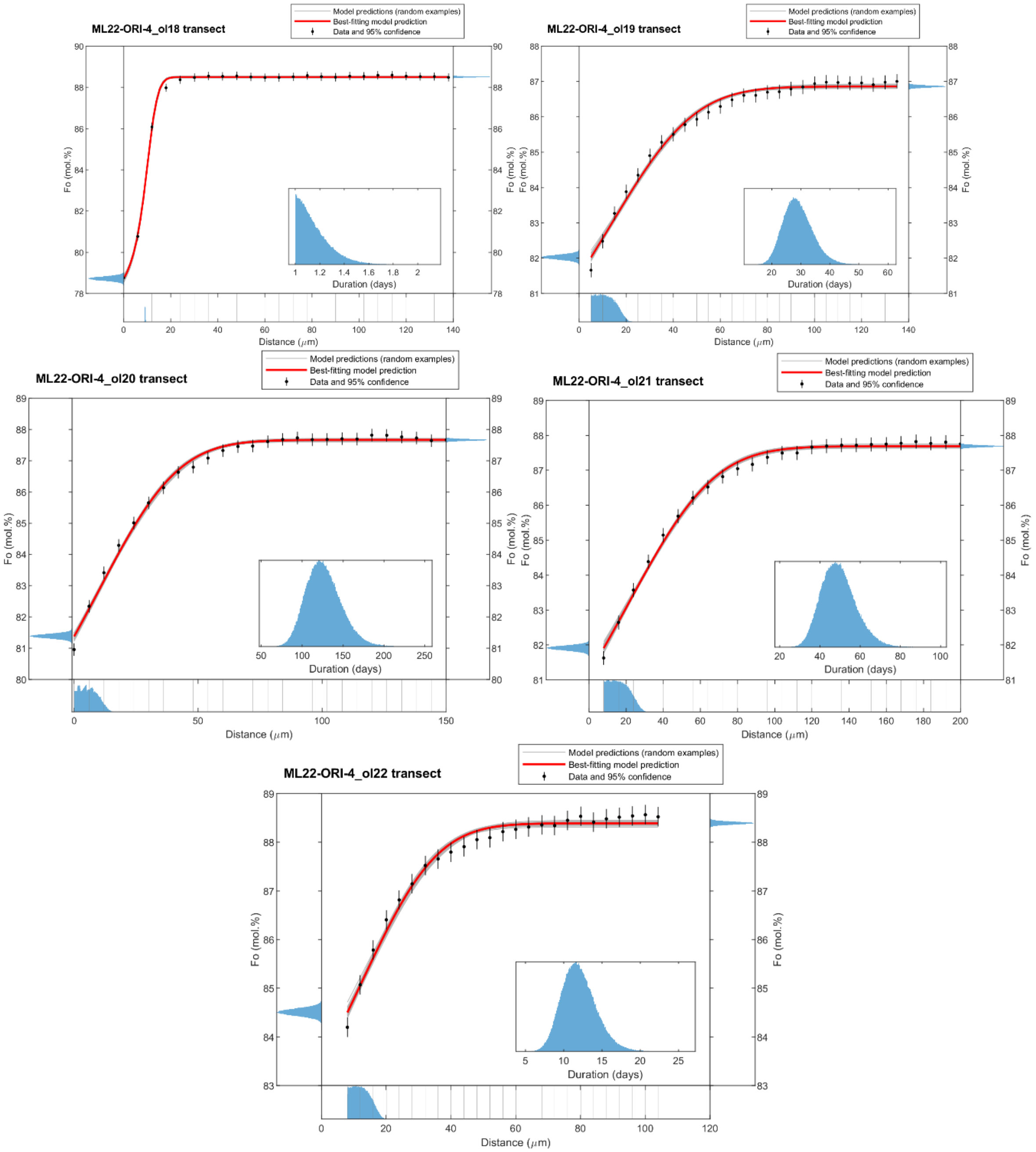

Figs. S12-S70. Olivine diffusion models. Model outputs for each olivine diffusion profile with timescale and initial and boundary condition distributions.

Movies S1 and S2. High-resolution X-ray computed tomography video of Mauna Loa 2022 south caldera olivine phenocrysts (sample ML22-88; Table S1) showing the rounded morphology at the crystal-melt interface and inclusions of Cr-spinel (red) melt (yellow), and fluid inclusions (blue).

References

1. Zoeller, M.H., Patrick, M.R., Bard, J.A., Trusdell, F.A., Parcheta, C.E., Wessels, R.L., Carbo, C.L., Mosbrucker, A.R., and Robinson, J.E. Geospatial database of the 2022 summit and Northeast Rift Zone eruption of Mauna Loa volcano, Hawaiʻi: *U.S. Geol. Surv. data release* (2024). doi: 10.5066/P1KES7F4
2. Sherrod, D.R., Sinton, J.M., Watkins, S.E., Brunt, K.M. Geologic map of the State of Hawaiʻi: *U.S. Geol. Surv. Sci. Inv. Map* **3143**, pamphlet 72 p., 5 sheets, scales 1:100,000 and 1:250,000 (2021). doi: 10.3133/sim3143
3. Sherrod, D.R., Robinson, J.E., Sinton, J.M., Watkins, S.E., Brunt, K.M. Geologic map database to accompany geologic map of the State of Hawaii: *U.S. Geol. Surv. data release* (2021). doi: 10.5066/P9YWXT41
4. Office for Coastal Management. 2005 Hawaii IfSAR Digital Terrain Model (DTM) from 2010-06-15 to 2010-08-15. NOAA National Centers for Environmental Information, (2024). https://www.fisheries.noaa.gov/inport/item/48377.
5. U.S. Geological Survey. USGS 1/3 Arc Second n20w156 20230522: U.S. Geological Survey. USGS 1/3 Arc Second n20w156 20230522 - ScienceBase-Catalog (1983).
6. Tucker, J.M., Hauri, E.H., Pietruszka, A.J., Garcia, M.O., Marske, J.P., Trusdell, F.A. A high carbon content of the Hawaiian mantle from olivine-hosted melt inclusions. *Geochim. Cosmochim. Acta* **254**, 156-172. doi: 10.1016/j.gca.2019.04.001
7. Wieser, P.E., Lamadrid, H., Maclennan, J., Edmonds, M., Matthews, S., Iacovino, K., Jenner, F.E., Gansecki, C., Trusdell, F., Lee, R.L., Ilyinskaya, E. Reconstructing Magma Storage Depths for the 2018 Kīlauean Eruption From Melt Inclusion CO2 Contents: The Importance of Vapor Bubbles. *Geochem. Geophys. Geosyst.* **22(2),** e2020GC009364 (2021). doi: 10.1029/2020GC009364
8. Schleicher, J.M. Crystal-scale control on magmatic mush mobilization and mixing. Ph.D. thesis, University of Washington, 110 p (2017).
9. Riker, J.M. The 1859 eruption of Mauna Loa volcano, Hawaiʻi: Controls on the development of long lava channels. M.S. thesis, University of Oregon, 131 p (2005).
10. Riker, J.M. Petrographic variation in Mauna Loa lavas: Implications for petrogenetic and magmatic processes. B.S. thesis, University of Oregon, 188 p (2003).

11. Couperthwaite, F.K., Morgan, D.J., Harvey, J., Kahl, M. Pre-eruptive timescales from the historical Hapaimamo eruption of Mauna Loa, Hawai‘i. J. *Volcanol. Geotherm. Res.* **432**, 107690 (2022). doi: 10.1016/j.jvolgeores.2022.107690
12. Couperthwaite, F.K., Thordardson, T., Morgan, D.J., Harvey, J., Wilson, M. Diffusion timesacles of magmatic processes in the Moinui lava eruption at Mauna Loa, Hawai‘i, as inferred from bimodal olivine populations. *J. Petrol.* **61**, egaa058 (2020). doi: 10.1093/petrology/egaa058
13. Kahl, M., Morgan, D.J., Thornber, C., Walshaw, R., Lynn, K.J., Trusdell, F.A. Dynamics of magma mixing and magma mobilization beneath Mauna Loa – insights from the 1950 AD Southwest Rift Zone eruption. *Bull. Volcanol.* **85**, 75 (2023). doi:10.1007/s00445-023-01680-x
14. Downs, D.T. et al. Sample details and near-real-time ED-XRF and grain size data collected during the November-December 2022 eruption of Mauna Loa volcano, Island of Hawai‘i. *USGS Data Rel.* (2023), doi: 10.5066/P9NA7GU3
15. Jarosewich, E., Nelen, J.A., Norberg, J.A. Reference samples for electron microprobe analysis. *Geos. News.* **4,** 43–47, (1980). doi:10.1111/ j.1751-908X.1980.tb00273.x
16. Cardozo, N., Allmendinger, R.W. Spherical projections with OSXStereonet: *Comp. Geosci.* **51**, 193–205 (2013). doi: 10.1016/j.cageo.2012.07.021
17. U.S. Geological Survey, Earthquake Hazards Program. Advanced National Seismic System (ANSS) Comprehensive Catalog of Earthquake Events and Products: Various, (2017). doi: 10.5066/F7MS3QZH
18. Matoza, R.S., Okubo, P.G., Shearer, P.M. Comprehensive high-precision relocation of seismicity on the island of Hawai‘i 1986-2018. *Earth Space Sci.* **7**, e2020EA001253 (2020). doi: 10.1029/2020EA001253
19. Wilding, J.D., Zhu, W., Ross, Z.E., Jackson, J.M. The magmatic web beneath Hawai‘i. *Science* **379(6631),** 462-468 (2022). doi: 10.1126/science.ade5755
20. Hotovec-Ellis, A.J. REDPy - Repeating Earthquake Detector in Python (Version 1.1.3). *USGS Software Release* (2024a) doi: 10.5066/P13SRZTC.
21. Hotovec-Ellis, A.J. REDPy Catalogs for Mauna Loa (2012-2024): *USGS data release* (2024b) doi: 10.5066/P1RAI6EP

22. Shea, T., Matzen, A.K., Mourey, A.J. Experimental study of Fe-Mg partitioning and zoning during rapid growth of olivine in Hawaiian tholeiites. *Cont. Mineral. Petrol.* **177,** 114 (2022). doi:10.1007/s00410-022-01969-8
23. Putirka, K.D. Thermometer and barometers for volcanic systems. *Rev. Mineral. Geochem.* **69**, 61-120 (2008). https://doi.org/10.2138/rmg.2008.69.3
24. Saper, L.M. et al. Experimental constraints on Fe and S redox equilibria and kinetics in basaltic melt inclusions. *Geochim. Cosmochim. Acta.* ***381**, 75-96* (2024). doi: 10.1016/j.gca.2024.07.018
25. Helz, R.T., Cottrell, E., Brounce, M.N., Kelley, K.A. Olivine-melt relationships and syneruptive redox variations in the 1959 eruption of Kīlauea Volcano as revealed by XANES. *J. Volcanol. Geotherm. Res.* **333–334**, 1–14 (2017). doi: 10.1016/j.jvolgeores.2016.12.006
26. Cervelli, P.F., Miklius, A. The shallow magmatic system of Kīlauea Volcano. *USGS Prof. Pap.* **1676**, 149-164.
27. Poland, M.P., Miklius, A., Montgomery-Brown, E.K. Magma supply, storage, and transport at shield-stage Hawaiian volcanoes. In: Characteristics of Hawaiian Volcanoes; Poland, Takahashi, Landowski, eds. *USGS Prof. Pap.* **1801**, 179-234 (2014).
28. Amelung, F., Yun, S-H., Walter, T.R., Segall, P., Kim, S-W. Stress control of deep rift intrusion at Mauna Loa Volcano, Hawaiʻi. *Science* **316**, 1026-1030 (2007). doi: 10.1126/science.1140035
29. Varugu, B., Amelung, F. Southward growth of Mauna Loa's dike-like magma body driven by topographic stress. *Scientific Reports* **11**, 9816 (2021). doi: 10.1038/s41598-021-89203-6
30. Lynn, K.J., Shea, T., Garcia, M.O. Nickel variability in Hawaiian olivine: Evaluating the relative contributions from mantle and crustal processes. *Am. Mineral.* **102**, 507-518. doi: 10.2138/am-2017-5763
31. Mourey, A.J., Shea, T., Hammer, J.E. Preservation of magma recharge signatures in Kīlauea olivine during protracted storage. Journal of Geophysical Research: Solid Earth, 128, e2022JB025523 (2023). doi: 10.1029/2022JB025523
32. Roedder, E. Fluid Inclusions. *Min. Soc. Am. Rev. in Min.* **12**, 646 pp (1984).

33. Hansteen, T.H., Klügel, A. Fluid inclusion thermobarometry as a tracer for magmatic processes. *Rev. Mineral. Geochem.* **69(1),** 143-177 (2008). doi: 10.2138/jrmg.2008.69.5
34. Kirby, S.H., Green, H.W. Dunite xenoliths from Hualalai volcano: evidence for mantle diapiric flow beneath the island of Hawaii. *Amer. J. Sci.* **A280,** 550–575 (1980).
35. Wanamaker, B.J., Evans, B. Mechanical re-equilibration of fluid-inclusions in San Carlos olivine by power-law creep. *Contrib. Mineral. Petrol.* **102**, 102-111 (1989).
36. Viti, C., Frezzotti, M-L. Re-equilibration of glass and CO_2 inclusions in xenolithic olivine: A TEM study. *Am. Mineral.* **85(10),** 1390-1396 (2000).
37. Yamamoto, J., Otsuka, K., Ohfuji, H., Ishibashi, H., Hirano, N., Kagi, H. Retentivity of CO_2 in fluid inclusions in mantle minerals. *Euro. J. Mineral.* **23(5),** 805–815 (2011). doi: 10.1127/0935-1221/2011/0023-2150
38. Yamamoto, J., Kagi, H., Kawakami, Y., Hirano, N., Nakamura, M. Paleo-Moho depth determined from the pressure of CO_2 fluid inclusions: Raman spectroscopic barometry of mantle- and crust-derived rocks. *Earth Planet. Sci. Lett.* **253(3-4),** 369-377 (2007). doi: 10.016/j.epsl.2006.10.038
39. Yamamoto, J., Kagi, H., Kaneoka, I., Lai, Y., Prikhod'ko, V.S., Arai, S. Fossil pressures of fluid inclusions in mantle xenoliths exhibiting rheology of mantle minerals: implications for the geobarometry of mantle minerals using micro-Raman spectroscopy. *Earth Planet. Sci. Lett.* **198(3-4),** 511-519 (2002). doi: 10.016/S0012-821X(02)00528-9
40. Bodnar, R.J. Reequilibration of fluid inclusions. In I. Samson, A. Anderson, D. Marshall, eds. Fluid Inclusions: Analysis and Interpretation. *Mineral. Assoc. Canada, Short Course* **32**, 213-230 (2003).
41. DeVitre, C.L., Wieser, P.E. Reliability of Raman analyses of CO2-rich fluid inclusions as a geobarometer at Kīlauea. *Geochem. Persp. Lett.* **29**, 1-8 (2024).
42. Lo Forte, F.M., Aiuppa, A., Rotolo, S.G., Zanon, V. Temporal evolution of the Fogo Volcano magma storage system (Cape Verde Archipelago): a fluid inclusions perspective. *J. Volcanol. Geotherm. Res.* **433**, 107730 (2023). doi: 10.1016/j.jvolgeores.2022.107730
43. Hildner, E., Klügel, A., Hansteen, T.H. Barometry of lavas from the 1951 eruption of Fogo, Cape Verde Islands: Implications for historic and prehistoric magma plumbing systems. *J. Volcanol. Geotherm. Res.* **217-218**, 73-90 (2012). doi: 10.1016/j.volgeores.2011.12.014

44. Hildner, E., Klügel, A., Hauff, F. Magma storage and ascent during the 1995 eruption of Fogo, Cape Verde Archipelago. *Cont. Mineral. Petrol.* **162**, 751-772 (2011). doi: 10.1007/s00410-011-0623-6
45. Dayton, K., Gazel, E., Wieser, P., Troll, V.R., Carracedo, J.C., La Madrid, H., Roman, D.C., Ward, J., Aulinas, M., Geiger, H., Deegan, F.M., Gisbert, G., Perez-Torrado, F.J. Deep magma storage during the 2021 La Palma eruption. *Sci. Adv.* **9(6),** eade7641 (2022). doi: 10.1126/sciadv.ade7641
46. Shea, T., Ruth, D., Jollands, M., Ohtaki, K., Ishii, H., Bradley, J. The presence of silicate melt may enhance rates of cation diffusion in olivine. *Earth Planet. Sci. Lett.* **621**, 118370, (2023). doi: 10.1016/j.epsl.2023.118370
47. Chakraborty, S. Diffusion Coefficients in Olivine, Wadsleyite and Ringwoodite. *Rev. Mineral. Geochem.* **72(1),** 603–639 (2010). doi: 10.2138/rmg.2010.72.13
48. Marske, J.P., Hauri, E.H. Major- and trace-element compositions of 915 melt inclusions and host olivines from Hawaiian shield volcanoes. Interdisciplinary Earth Data Alliance (IEDA) (2019). doi: 10.1594/IEDA/111193
49. Span, R., Wagner, W. A New Equation of State for Carbon Dioxide covering the Fluid Region from the Triple-point Temperature to 1100 K at Pressures up to 800 MPa. *J. Phys. Chem. Ref. Data* **25(6)**, 1509–1596 (1996).
50. Duan, Z., Zhang, Z. Equation of state of the H2O, CO2, and H2O–CO2 systems up to 10 GPa and 2573.15 K: Molecular dynamics simulations with ab initio potential surface. *Geochim. Cosmochim. Acta* **70(9),** 2311-2324 (2006).
51. Lerner, A.H., Sublett Jr, D.M., Wallace, P.J., Cauley, C., Bodnar, R.J. Insights into magma storage depths and eruption controls at Kīlauea Volcano during explosive and effusive periods of the past 500 years based on melt and fluid inclusions. *Earth Planet. Sci. Lett.* **628**, 118579 (2024).
52. Lerner, A.H., Wallace, P.J., Shea, T., Mourey, A.J., Kelly, P.J., Nadeau, P.A., Elias, T., Kern, C., Clor, L.E., Gansecki, C., Lee, R.L., Moore, L.R., Werner, C.A. The petrologic and degassing behavior of sulfur and other magmatic volatiles from the 2018 eruption of Kīlauea, Hawaiʻi: melt concentrations, magma storage depths, and magma recycling. *Bull. Volcanol.* **83**, 43 (2021). https://doi.org/10.1007/s00445-021-01459-y

Bibliography for Diffusion Studies at Volcanoes with Eruptions 1500-2024 CE

The following reference list represents examples of diffusion studies applied at historically active volcanoes around the globe (n=56; Fig. 1 main text). This is not meant to be a comprehensive list of all diffusion studies published for each of these volcanoes, but instead represents the rationale for the calculation that 9% of active volcanoes with an eruption between 1500-2024 CE have some type of diffusion chronometry study applied to any of their eruptions. At the end of the list we separate out the 19 studies that have any type of monitoring comparison, from which we determined that only four volcanoes (Kīlauea, Mount Ruapehu, Mount Etna, and Piton de la Fournaise) have multiparametric syntheses. See below for more details.

Diffusion Study, no Monitoring Comparison

Albert, H., Trua, T., Fonseca, J., Marani, M.P., Gamberi, F., Spiess, R., Marzoli, A. Time scales of open-system processes in a complex and heterogeneous mush-dominated plumbing system. *Geology* **50(8),** 869-873 (2022). doi: 10.1130/G49934.1

Albert, H., Costa, F., Marti, J. Timing of magmatic processes and unrest associated with mafic historical monogenetic eruptions in Tenerife Island. *J. Petrol.* **56**, 1945–1965 (2015).

Audétat, A., Schmitt, A.K., Njul, R., Saalfeld, M., Borisova, A., Lu, Y. New constraints on Ti diffusion in quartz and the priming of silicic volcanic eruptions. *Nature Comm.* **14**, 4277 (2023). doi: s41467-023-39912-5

Barth, A., Newcombe, M., Plank, T., Gonnermann, H., Hajimirza, S., Soto, G.J., Saballos, A., Hauri, E. Magma decompression rate correlates with explosivity at basaltic volcanoes - Constraints from water diffusion in olivine. *J. Volcanol. Geotherm. Res.* **387**, 106664 (2019). doi: 10.1016/j.jvolgeores.2019.106664

Berthod, C., Médard, E., Bachèlery, P., Gurioli, L., Di Muro, A., Peltier, A., Kommorowski, J-C., Benbakkar, M., Devidal, J-L., Langlade, J., Besson, P., Boudon, G., Rose-Koga, E., Deplus, C., Le Friant, A., Bickert, M., Nowak, S., Thinon, I., Burckel, P., Hidalgo, S., Kaliwoda, M., Jorry, S.J., Fouquet, Y., Feuillet, N. The 2018-ongoing Mayotte submarine eruption: Magma migration imaged by petrological monitoring. *Earth Planet. Sci. Lett.* **571**, 117085 (2021). doi: 10.1016/j.epsl.2021.117085

Borzi, A.M., Giuffrida, M., Zuccarello, F., Palano, M., Viccaro, M. The Christmas 2018 Eruption at Mount Etna: Enlightening How the Volcano Factory Works Through a Multiparametric

Inspection. *Geochem. Geophys. Geosys.* **21**, 2020GC009226 (2020), doi: 10.1029/2020GC009226

Cannata, A., Spedalieri, G., Behncke, B., Cannavò, F., De Grazia, G., Gambino, S., Gresta, S., Gurrieri, S., Liuzzo, M., Planao, M. Pressurization and depressurization phases inside the plumbing system of Mount Etna volcano: Evidence from a multiparametric approach. *J. Geophys. Res. Solid Earth* **120**, 5965–5982 (2015).

Caracciolo, A., Kahl, M., Bali, E., Gudfinnsson, G.H., Halldorsson, S.A., Hartley, M.E. Timescales of crystal mush mobilization in the Baroarbunga-Veioivotn volcanic system based on olivine diffusion chronometry. *Am. Mineral.* **106**, 1083-1096 (2021). doi: 10.2138/am-2021-7670

Chertkoff, D.G., Gardner, J.E. Nature and timing of magma interactions before, during, and after the caldera-forming eruption of Volcán Ceboruco, Mexico. *Contrib. Mineral. Petrol.* **146(6),** 715-735 (2004). doi: 10.1007/s00410-003-0530-6

Conway, C.E., Chamberlain, K.J., Harigane, Y., Morgan, D.J., Wilson, C.J.N. Rapid assembly of high-Mg andesites and dacites by magma mixing at a continental arc stratovolcano. *Geology* **48**, 1033-1037 (2020). doi: 10.1130/G47614.1

Coombs, M.L., Eichelberger, J.C., Rutherford, M.J. Magma storage and mixing conditions for the 1953–1974 eruptions of Southwest Trident Volcano, Katmai National Park, Alaska. *Contrib. Mineral. Petrol.* **140**, 99-118 (2000).

Cooper, K.M., Kent, A.J.R. Rapid remobilization of magmatic crystals kept in cold storage. *Nature* **506**, 480-483 (2014).

Costa, F., Andreastuti, S., de Maisonneuve, C.B., Pallister, J.S. Petrological insights into the storage conditions, and magmatic processes that yielded the centennial 2010 Merapi explosive eruption. *J. Volcanol. Geotherm. Res.* **261**, 209-235 (2013). doi: 10.1016/j.jvolgeores.2012.12.025

Couperthwaite, F.K., Morgan, D.J., Harvey, J., Kahl, M. Pre-eruptive timescales from the historical Hapaimamo eruption of Mauna Loa, Hawaiʻi. J. *Volcanol. Geotherm. Res.* **432**, 107690 (2022). doi: 10.1016/j.jvolgeores.2022.107690

Couperthwaite, F.K., Thordardson, T., Morgan, D.J., Harvey, J., Wilson, M. Diffusion timesacles of magmatic processes in the Moinui lava eruption at Mauna Loa, Hawaiʻi, as inferred from bimodal olivine populations. *J. Petrol.* **61**, egaa058 (2020). doi: 10.1093/petrology/egaa058

Davidson, J., Tepley, F. III., Palacz, Z., Meffan-Main, S. Magma recharge, contamination and residence times revealed by in situ laser ablation isotopic analysis of feldspar in volcanic rocks. *Earth Planet Sci. Lett.* **184**, 427-442 (2001).

de Leon, A.C., Schmitt, A.K. Reconciling Li and O diffusion in zircon with protracted magmatic crystal residence. *Contrib. Mineral. Petrol.* **174**, 28 (2019). doi: 10.1007/s00410-019-1564-8

Devine, J.D., Rutherford, M.J., Norton, G.E., Young, S.R. Magma storage region processes inferred from geochemistry of Fe–Ti oxides in andesitic magma, Soufriere Hills Volcano, Montserrat, WI. *J. Petrol.* **44(8),** 1375-1400 (2003). doi: 10.1093/petro logy/44.8.1375

Di Stefano, F., Mollo, S., Ubide, T., Petrone, C.M., Caulfield, J., Scarlato, P., Nazzari, M., Andronico, D., Del Bello, E. Mush cannibalism and disruption recorded by clinopyroxene phenocrysts at Stromboli volcano: New insights from recent 2003-2017 activity. *Lithos* **360**, 105440 (2020). doi: 10.1016/j.lithos.2020.105440

Druitt, T.H., Costa, F., Deloule, E., Dungan, M., Scaillet, B. Decadal to monthly timescales of magma transfer and reservoir growth at a caldera volcano. *Nature* **482**, 77-80 (2012). doi: 10.1038/ nature10706

Edmonds, M., Kohn, S.C., Hauri, E.H., Humphreys, M.C.S., Cassidy, M. Extensive, water-rich magma reservoir beneath southern Montserrat. *Lithos* **252**, 216-233 (2016). doi: 10.1016/j.lithos.2016.02.026

Fabbro, G.N., Druitt, T.H., Costa, F. Storage and eruption of silicic magma across the transition from dominantly effusive to caldera-forming states at an arc volcano (Santorini, Greece). *J. Petrol.* **58**, 2429-2464 (2017).

Ferguson, D.J., Gonnermann, H.M., Ruprecht, P., Plank, T., Hauri, E.H., Houghton, B.F., Swanson, D.A. Magma decompression rates during explosive eruptions of Kilauea volcano, Hawaii, recorded by melt embayments. *Bull. Volcanol.* **78**, 71 (2016). doi: 10.1007/s00445-016-1064-x.

Ferriss, E., Plank, T., Newcombe, M., Walker, D., Hauri, E. Rates of dehydration of olivines from San Carlos and Kilauea Iki. *Geochim. Cosmochim. Acta* **242**, 165-190, (2018). doi 10.1016/j.gca.2018.08.050

Finney, B., Turner, S., Hawkesworth, C., Larsen, J., Nye, C., George, R., Bindeman, I., Eichelberger, J. Magmatic differentiation at an island-arc caldera: Okmok volcano, Aleutian Islands, Alaska. *J. Petrol.* **49**, 857-884 (2008) doi: 10.1093/petrology/egn008

Flaherty, T., Druitt, T.H., Tuffen, H., Higgins, M.D., Costa, F., Cadoux, A. Multiple timescale constraints for high-flux magma chamber assembly prior to the Late Bronze Age eruption of Santorini (Greece). *Contrib. Mineral. Petrol.* **173**, 75 (2018). doi: 10.1007/s00410-018-1490-1

Giuffrida, M., Viccaro, M., Ottolini, L. Ultrafast syn-eruptive degassing and ascent trigger high-energy basic eruptions. *Sci. Reports* **8**, 147 (2018) doi: 10.1038/s41598-017-18580-8

Gordeychik, B., Churikova, T., Kronz, A., Sundermeyer, C., Simakin, A., Wörner, G. Growth of, and diffusion in, olivine in ultra-fast ascending basalt magmas from Shiveluch volcano. *Sci. Reports* **8**, 11775 (2018). doi: s41598-018-30133-1

Hartley, M.E., Bali, E., Maclennan, J., Neave, D.A., Halldorsson, S.A. Melt inclusion constraints on petrogenesis of the 2014-2015 Holuhraun eruption, Iceland. *Contrib. Mineral. Petrol.* **173**, (2018). doi: 10.1007/s00410-017-1435-0

Hartley, M.E., Shorttle, O., Maclennan, J., Moussallam, Y., Edmonds, M. Olivine-hosted melt inclusions as an archive of redox heterogeneity in magmatic systems. *Earth Planet Sci. Lett.* **479**, 192-205, (2017). doi: 10.1016/j.epsl.2017.09.029

Hartley, M.E., Morgan, D.J., Maclennan, J., Edmonds, M., Thordarson, T. Tracking timescales of short-term precursors to large basaltic fissure eruptions through Fe–Mg diffusion in olivine. *Earth Planet Sci. Lett.* **439**, 58-70 (2016)

Hollyday, A.E., Leiter, S.H., Walowski, K.J. Pre-eruptive storage, evolution, and ascent timescales of a high-Mg basaltic andesite in the southern cascade arc. *Contrib. Mineral. Petrol.* **175**, 88 (2020). doi: 10.1007/s00410-020-01730-z

Humphreys, M.C.S., Menand, T., Blundy, J.D., Klimm, K. Magma ascent rates in explosive eruptions: Constraints from H2O diffusion in melt inclusions. *Earth Planet Sci. Lett.* **270,** 25-40 (2008).

Iovine, R.S., Fedele, L., Mazzeo, F.C., Arienzo, I., Cavallo, A., Worner, G., Orsi, G., Civetta, L., D'Antonio, M. Timescales of magmatic processes prior to the similar to 4.7 ka Agnano-Monte Spina eruption (Campi Flegrei caldera, Southern Italy) based on diffusion chronometry from sanidine phenocrysts. *Bull. Volcanol.* **79**, 18 (2017). doi: 10.1007/s00445-017-1101-4

Johnson, E.R., Wallace, P.J., Cashman, K.V., Granados, H.D., Kent, A.J.R. Magmatic volatile contents and degassing-induced crystallization at Volcán Jorullo, Mexico: Implications for

melt evolution and the plumbing systems of monogenetic volcanoes. *Earth Planet. Sci. Lett.* **269**, 478-487. doi: 10.1016/j.epsl.2008.03.004

Kahl, M., Morgan, D.J., Thornber, C., Walshaw, R., Lynn, K.J., Trusdell, F.A. Dynamics of magma mixing and magma mobilization beneath Mauna Loa – insights from the 1950 AD Southwest Rift Zone eruption. *Bull. Volcanol.* **85**, 75 (2023). doi:10.1007/s00445-023-01680-x

Kahl, M., Viccaro, M., Ubide, T., Morgan, D.J., Dingwell, D.B. A branched magma feeder system during the 1669 eruption of Mt Etna: Evidence from a time-integrated study of zoned olivine phenocryst populations. *J. Petrol.* **58,** 443–472 (2017).

Kahl, M., Chakraborty, S., Pompilio, M., Costa, F. Constraints on the nature and evolution of the magma plumbing system of Mt. Etna Volcano (1991–2008) from a combined thermodynamic and kinetic modeling of the compositional record of minerals. *J. Petrol.* **56**, 2015-2068 (2015).

Kahl, M., Chakraborty, S., Costa, F., Pompilio, M., Liuzzo, M., Viccaro, M. Compositionally zoned crystals and real-time degassing data reveal changes in magma transfer dynamics during the 2006 summit eruptive episodes of Mt. Etna. *Bull. Volcanol.* **75**, 1-14 (2013).

Klügel, A. Reactions between mantle xenoliths and host magma beneath La Palma (Canary Islands): Constraints on magma ascent rates and crustal reservoirs. *Contrib. Mineral. Petrol.* **131**, 237-257 (1998). doi: 10.1007/s004100050391

Larrea, P., Albert, H., Ubide, T., Costa, F., Colas, V., Widom, E., Siebe, C. From explosive vent opening to effusive outpouring: Mineral constraints on magma dynamics and timescales at Paricutin Monogenetic Volcano. *J. Petrol.* **62**, egaa112 (2021). doi: 10.1093/petrology/egaa112

Li, W.R., Costa, F., Nagashima, K. Apatite crystals reveal melt volatile budgets and magma storage depths at Merapi Volcano, Indonesia. *J. Petrol.* **62**, egaa100 (2021). doi: 10.1093/petrology/egaa100

Lloyd, A.S., Ferriss, E., Ruprecht, P., Hauri, E.H., Jicha, B.R., Plank, T. An assessment of clinopyroxene as a recorder of magmatic water and magma ascent rate. *J. Petrol.* **57**, 1865-1885 (2016). doi: 10.1093/petrology/egw058

Longpré, M.A., Klügel, A., Diehl, A., Stix, J. Mixing in mantle magma reservoirs prior to and during the 2011–2012 eruption at El Hierro, Canary Islands. *Geology* **42**, 315-318 (2014a).

Longpré, M.A., Stix, J., Costa, F., Espinoza, E., Munoz, A. Magmatic processes and associated timescales leading to the January 1835 eruption of Cosiguina Volcano, Nicaragua. *J. Petrol.* **55**, 1173-1201 (2014b). doi: 10.1093/petrology/egu022

Lynn, K.J., Helz, R.T. Magma storage and transport timescales for the 1959 Kīlauea Iki eruption and implications for diffusion chronometry studies using time-series samples versus tephra deposits. *Bull. Volcanol.* **85**, 3 (2023). doi: 10.1007/s00445-022-01618-9 [earthquakes, tilt]

Mangler, M.F., Petrone, C.M., Prytulak, J. Magma recharge patterns control eruption styles and magnitudes at Popocatépetl volcano (Mexico). *Geology* **50(3),** 366-370. doi: 10.1130/G49365.1

Martin, V.M., Morgan, D.J., Jerram, D.A., Caddick, M.J., Prior, D.J., and Davidson, J.P. Bang! Month-scale eruption triggering at santorini volcano. *Science* **321**, 1178 (2008).

McCarthy, A., Chelle-Michou, C., Blundy, J.D., Vonlanthen, P., Meibom, A., Escrig, S. Taking the pulse of volcanic eruptions using plagioclase glomerocrysts. *Earth Planet. Sci. Lett.* **552**, 116596 (2020). doi: 10.1016/j.epsl.2020.116596

Metcalfe, A., Moune, S., Komorowski, J.C., Kilgour, G., Jessop, D.E., Moretti, R., Legendre, Y. Magmatic processes at La Soufriere de Guadeloupe: Insights from crystal studies and diffusion timescales for eruption snset. *Frontiers Earth Sci.* **9**, 617294 (2021). doi: 10.3389/feart.2021.617294

Moore, A., Coogan, L.A., Costa, F., Perfit, M.R. Primitive melt replenishment and crystal-mush disaggregation in the weeks preceding the 2005–2006 eruption 9°50′ N, EPR. *Earth Planet. Sci. Lett.* **403**, 15-26 (2014).

Morgado, E., Morgan, D.J., Castruccio, A., Ebmeier, S.K., Parada, M.A., Brahm, R., Harvey, J., Gutierrez, F., Walshaw, R. Old magma and a new, intrusive trigger: using diffusion chronometry to understand the rapid-onset Calbuco eruption, April 2015 (Southern Chile). *Contrib. Mineral. Petrol.* **174**, 61 (2019). doi: 10.1007/s00410-019-1596-0

Morgan, D.J., Blake, S., Rogers, N.W., De Vivo, B., Rolandi, G., Macdonald, R., Hawkesworth, C.J. Timescales of crystal residence and magma chamber volume from modelling of diffusion profiles in phenocrysts: Vesuvius 1944. *Earth Planet. Sci. Lett.* **222(3-4),** 933-946 (2004).

Morgan, D.J., Blake, S., Rogers, N.M., De Vivo, B., Rolandi, G., Davidson, J.P. Magma chamber recharge at Vesuvius in the century prior to the eruption of A.D. 79. *Geology* **34**, 845-848 (2006). doi: 10.1130/G2260 4.1

Moussallam, Y., Médard, E., Georgeais, G., Rose-Koga, E.F., Koga, K.T., Pelletier, B., Shreve, T.L., Grandin, R., Boichu, M., Tari, D., Peters, N. How to turn off a lava lake? A petrological investigation of the 2018 intra-caldera and submarine eruptions of Ambrym volcano. *Bull. Volcanol.* **83**, 36 (2021). doi: 10.1007/s00445-021-01455-2

Moussallam, Y., Rose-Koga, E.F., Koga, K.T., Medard, E., Bani, P., Devidal, J.L., Tari, D. Fast ascent rate during the 2017-2018 Plinian eruption of Ambae (Aoba) volcano: a petrological investigation. *Contrib. Mineral. Petrol.* **174**, 90 (2019). doi: 10.1007/s00410-019-1625-z

Moshrefzadeh, J., Izbekov, P., Loewen, M., Larsen, J., Regan, S. Dating individual phenocrysts from the 2016-2017 eruption of Bogoslof volcano provides constraints on timescales of magmatic processes. *J. Volcanol. Geotherm. Res.* **435**, 107741. doi: 10.1016/j.volgeores.2022.107741

Mutch, E. J. F., Maclennan, J., Shorttle, O., Rudge, J. F., & Neave, D. A. DFENS: Diffusion chronometry using finite elements and nested sampling. *Geochem. Geophys. Geosys.* **22**, e2020GC009303 (2021). doi: 10.1029/2020GC009303

Myers, M.L., Druitt, T.H., Schiavi, F., Gurioli, L., Flaherty, T. Evolution of magma decompression and discharge during a Plinian event (Late Bronze-Age eruption, Santorini) from multiple eruption-intensity proxies. *Bull. Volcanol.* **83**, 18 (2021). doi: 10.1007/s00445-021-01438-3

Nakagawa, M., Matsumoto, A., Kobayashi, K., Wada, K. Comparative petrological studies of 1962 and 1988-1989 eruptions of Tokachidake Volcano, Japan: A case study for understanding the relationship between eruption style and magma processes. *J. Disaster. Res.* **14**, 766-779 (2019). doi: 10.20965/jdr.2019.p0766

Nakamura, M. Continuous mixing of crystal mush and replenished magma in the ongoing Unzen eruption. *Geology* **23(9)**, 807-810 (1995). doi: 10.1130/0091-7613(1995)023%3c0807:CMOCMA%3e2.3.CO;2

Newcombe, M.E., Plank, T., Barth, A., Asimow, P.D., Hauri, E. Water-in-olivine magma ascent chronometry: Every crystal is a clock. *J. Volcanol. Geotherm. Res.* **398**, 106872 (2020). doi: 10.1016/j.jvolgeores.2020.106872

Newcombe, M.E., Fabbrizio, A., Zhang, Y., Ma, C., Le Voyer, M., Guan, Y., Eiler, J.M., Saal, A.E., Stolper, E.M. Chemical zonation in olivine-hosted melt inclusions. *Contrib. Mineral. Petrol.* **168**, 1–26 (2014).

Nikkola, P., Bali, E., Kahl, M., van der Meer, Q.H.A., Ramo, O.T., Gudfinnsson, G.H., Thordarson, T. Mid-crustal storage and crystallization of Eyjafjallajokull ankaramites, South Iceland. *Jokull* **69**, 83-102 (2019). doi: 10.33799/jokull2019.69.083

Nicotra, E., Giuffrida, M., Viccaro, M., Donato, P., D'Oriano, C., Paonita, A., De Rosa, R. Timescales of pre-eruptive magmatic processes at Vulcano (Aeolian Islands, Italy) during the last 1000 years. *Lithos* **316**, 347-365 (2018) doi: 10.1016/j.lithos.2018.07.028

Nishi, Y., Ban, M., Takebe, M., Alvarez-Valero, A.M., Oikawa, T., Yamasaki, S. Structure of the shallow magma chamber of the active volcano Mt. Zao, NE Japan: Implications for its eruptive time scales. *J. Volcanol. Geotherm. Res.* **371**, 137-161 (2019). doi: 10.1016/j.jvolgeores.2019.01.003

Oeser, M., Dohmen, R., Horn, I., Schuth, S., Weyer, S. Processes and time scales of magmatic evolution as revealed by Fe–Mg chemical and isotopic zoning in natural olivines. *Geochim. Cosmochim. Acta* **154**, 130-150 (2015).

Pankhurst, M.J., Morgan, D.J., Thordarson, T., Loughlin, S.C. Magmatic crystal records in time, space, and process, causatively linked with volcanic unrest. *Earth Planet. Sci. Lett.* **493**, 231-241 (2018). doi: 10.1016/j.epsl.2018.04.025

Petrone, C.M., Bugatti, G., Braschi, E., Tommasini, S. Pre-eruptive magmatic processes re-timed using a non-isothermal approach to magma chamber dynamics. *Nature Comm.* **7**, 12946 (2016) doi: 10.1038/ncomms12946

Petrone, C.M., Braschi, E., Francalanci, L., Casalini, M., Tommasini, S. Rapid mixing and short storage timescale in the magma dynamics of a steady-state volcano. *Earth Planet. Sci. Lett.* **492**, 206-221 (2018). doi: 10.1016/j.epsl.2018.03.055

Pichavant, M., Poussineau, S., Lesne, P., Solaro, C., Bourdier, J.L. Experimental parametrization of magma mixing: Application to the AD 1530 eruption of La Soufriere, Guadeloupe (Lesser Antilles). *J. Petrol.* **59**, 257-281 (2018).

Rasmussen, D.J., Plank, T.A., Roman, D.C., Power, J.A., Bodnar, R.J., Hauri, E.H. When does eruption run-up begin? Multidisciplinary insight from the 1999 eruption of Shishaldin volcano. *Earth Planet. Sci. Lett.* **486**, 1-14 (2018). doi: 10.1016/j.epsl.2018.01.001

Ruprecht, P., Plank, T. Feeding andesitic eruptions with a high-speed connection from the mantle. *Nature* **500**, 68–72 (2013).

Ruth, D.C.S., Costa, F. A petrological and conceptual model of Mayon volcano (Philippines) as an example of an open-vent volcano. *Bull. Volcanol.* **83**, 62 (2021). doi: 10.1007/s00445-021-01486-9

Sato, H., Holtz, F., Botcharnikov, R.E., Nakada, S. Intermittent generation of mafic enclaves in the 1991-1995 dacite of Unzen Volcano recorded in mineral chemistry. *Contrib. Mineral. Petrol.* **172,** 22 (2017). doi: 10.1007/s00410-017-1335-3

Shane, P., Costa, F., Cronin, S., Stirling, C., Reid, M. Priming and eruption of andesite magmas at Taranaki volcano recorded in plagioclase crystals. *Bull. Volcanol.* **85,** 47 (2023). doi: 10.1007/s00445-023-01661-0

Shea, T., Lynn, K.J., Garcia, M.O. Cracking the olivine zoning code: Distinguishing between crystal growth and diffusion. *Geology* **43**, 935-938 (2015a).

Singer, B., Costa, F., Herrin, J.S., Hildreth, W., Fierstein, J. The timing of compositionally zoned magma reservoirs and mafic 'priming' weeks before the 1912 Novarupta-Katmai rhyolite eruption. *Earth Planet. Sci. Lett.* **451**, 125-137 (2016).

Sio, C.K., Dauphas, N. Thermal and crystallization histories of magmatic bodies by Monte Carlo inversion of Mg–Fe isotopic profiles in olivine. *Geology* **45(1),** 67-70 (2017) doi: 10.1130/G38056.1

Sio, C.K., Dauphas, N., Teng, F-Z., Chaussidon, M., Helz, R.T., Roskosz, M. Discerning crystal growth from diffusion profiles in zoned olivine by in-situ Mg-Fe isotopic analyses. *Geochim. Cosmochim. Acta* **123**, 302-321 (2013).

Suzuki, Y., Yasuda, A., Hokanishi, N., Kaneko, T., Nakada, S., Fujii, T. Syneruptive deep magma transfer and shallow magma remobilization during the 2011 eruption of Shinmoedake, Japan-Constraints from melt inclusions and phase equilibria experiments. *J. Volcanol. Geotherm. Res.* **257**, 184-204 (2013). doi: 10.1016/j.jvolgeores.2013.03.017

Tepley, F.J. III., Davidson, J.P., Tilling, R.I., Arth, J.G. Magma mixing, recharge and eruption histories recorded in plagioclase phenocrysts from El Chichón volcano, Mexico. *J. Petrol.* **41,** 1397-1411 (2000).

Utami, S.B., Costa, F., Humaida, H. The role of magma ascent rates and viscosity in explosive and dome eruptions (Kelud volcano, Indonesia). *Bull. Volcanol.* **86**, 12 (2024). doi: 10.1007/s00445-023-01698-1

Viccaro, M., Giuffrida, M., Zuccarello, F., Scandura, M., Palano, M., Gresta, S. Violent paroxysmal activity drives self-feeding magma replenishment at Mt. Etna. *Sci. Reports* **9**, 6717 (2019). doi: 10.1038/s41598-019-43211-9

Viccaro, M., Giuffrida, M., Nicotra, E., Cristofolini, R. Timescales of magma storage and migration recorded by olivine crystals in basalts of the March–April 2010 eruption at Eyjafjallajökull volcano, Iceland. *Am. Mineral.* **101**, 222–230 (2016a).

Viccaro, M., Barca, D., Bohrson, W.A., D'Oriano, C., Giuffrida, M., Nicotra, E., Pitcher, B.W. Crystal residence times from trace element zoning in plagioclase reveal changes in magma transfer dynamics at Mt. Etna during the last 400 years. *Lithos* **248**, 309-323 (2016c). doi: 10.1016/j.lithos.2016.02.004

Zellmer, G.F., Blake, S., Vance, D., Hawkesworth, C., Turner, S. Plagioclase residence times at two island arc volcanoes (Kameni Islands, Santorini, and Soufriere, St. Vincent) determined by Sr diffusion systematics. Contrib. Mineral. Petrol. **136**, 345-357 (1999).

Diffusion Studies with Monitoring Comparisons (<4 Data Types)

We organize the following list by country and volcano/volcanic center. The type(s) of monitoring data used in comparisons are noted in brackets [ ] at the end of the citation. A data type is defined as an original dataset or an additional calculated or modeled dataset that is derived from the original. For example, comparison with frequency of earthquakes would be one data type, and additional comparison to hypocentral depths would be an additional data type. We define multidisciplinary comparisons as those that directly compare diffusion results with equivalent time series of monitoring datasets. Descriptions, broad overviews of pre-eruptive chronologies, and major pre-eruptive event chronology highlights (e.g., Lynn et al. 2023 for 1959 Kīlauea Iki eruption) are not considered as multidisciplinary syntheses, as they are not as robust as direct comparisons of datasets. Data types identified in the following studies include:

1) Seismic
    a. earthquake frequency
    b. earthquake depth

   c. earthquake location
   d. earthquake size or magnitude
   e. tremor amplitude
   f. tremor position
   g. tremor event frequency
   h. cumulative seismic moment or accumulated energy
   i. seismic velocity
2) Geodetic
   a. tilt
   b. GPS displacement
   c. GPS line length
   d. geodetic velocity
   e. InSAR
3) Chemistry
   a. gas chemistry
   b. water lake chemistry

Chile (Llaima)

Ruth, D.C.S., Costa, F., de Maisonneuve, C.B., Franco, L., Cortes, J.A., Calder, E.S. Crystal and melt inclusion timescales reveal the evolution of magma migration before eruption. *Nature Comm.* **9**, 2657 (2018). doi: 10.1038/s41467-018-05086-8 [RSAM]

France (Piton de la Fournaise)

Albert, H., Costa, E., Di Muro, A., Herrin, J., Metrich, N., Deloule, E. Magma interactions, crystal mush formation, timescales, and unrest during caldera collapse and lateral eruption at ocean island basaltic volcanoes (Piton de la Fournaise, La Reunion) *Earth Planet. Sci. Lett.* **515**, 187-199 (2019), doi: 10.1016/j.epsl.2019.02.035 [tilt]

Italy (Mtount Etna, Stromboli)

Kahl, M., Chakraborty, S., Costa, F., Pompilio, M. Dynamic plumbing system beneath volcanoes revealed by kinetic modeling and the connection to monitoring data: An example from Mt. Etna. *Earth Planet Sci. Lett.* **308**, 11-22 (2011). [gas geochemistry, focal depths of seismic events, tilt]

Petrone, C.M., Mollo, S., Gertisser, R., Buret, Y., Scarlato, P., Del Bello, E., Andronico, D., Ellis, B., Pontesilli, A., De Astis, G., Giacomoni, P.P., Coltorti, M., Reagan, M. Magma recharge and mush rejuvenation drive paroxysmal activity at Stromboli volcano. *Nature Comm.* **13,** 7717 (2022). doi: 10.1038/s41467-022-34505-z [gas geochemistry, VLP size]

Iceland (Fagradalsfjall, Eyjafjallajökull)

Kahl, M., Mutch, E.J.F., Maclennan, J., Morgan, D.J., Couperthwaite, F., Bali, E., Thordarson, T., Guðfinnsson, G.T., Walshaw, R., Buisman, I., Buhre, S., van der Meer, Q.H.A., Caracciolo, A., Marshall, E.W., Rasmussen, M.B., Gallagher, C.R., Moreland, W.M., Höskuldsson, A., Askew, R.A. Deep mamga mobilization years before the 2021 CE Fagradalsfjall eruption, Iceland. *Geology* **51**, 184-188 (2023). doi: 10.1130/G50340.1 [earthquake frequency, cumulative seismic energy release]

Pankhurst, M.J., Morgan, D.J., Thordarson, T., Loughlin, S.C. Magmatic crystal records in time, space, and process, causatively linked with volcanic unrest. Earth Planet. Sci. Lett. 493, 231-241 (2018). doi: 10.1016/j.epsl.2018.04.025 [earthquake frequency, GPS displacement]

Japan (Shinmoedake)

Tomiya, A., Miyagi, I., Saito, G., Geshi, N. Short time scales of magma-mixing processes prior to the 2011 eruption of Shinmoedake volcano, Kirishima volcanic group, Japan. *Bull. Volcanol.* **75(10),** 750 (2013). doi: 10.1007/s00445-013-0750-1 [GPS displacement, tilt]

Russia (Bezymianny, Kizimen)

Davydova, V.O., Shcherbakov, V.D., Plechov, P.Y. The timescales of magma mixing in the plumbing system of Bezymianny Volcano (Kamchatka): Insights from diffusion chronometry. *Moscow Univ. Geol. Bull.* **73**, 444-450 (2018). doi: 10.3103/S0145875218050058 [earthquake frequency]

Ostorero, L., Balcone-Boissard, H., Boudon, G., Shapiro, N.M., Belousov, A., Belousova, M., Auer, A., Senyukov, S.L., Droznina, S.Y. Correlated petrology and seismicity indicate rapid magma accumulation prior to eruption of Kizimen volcano, Kamchatka. *Nature Comm. Earth Env.* **3**, 290 (2022). doi: 10.1038/s43247-022-00622-3 [InSAR, earthquake depths, cumulative seismic moment]

Spain (El Hierro)

Marti, J., Castro, A., Rodriguez, C., Costa, F., Carrasquilla, S., Pedreira, R., Bolos, X. Correlation of magma evolution and geophysical monitoring during the 2011–2012 El Hierro

(Canary Islands) Submarine Eruption. *J. Petrol.* **54**, 1349–1373 (2013). [depth of hypocenters, frequency of earthquakes, accumulated energy]

United States (Kīlauea, Shishaldin Volcano, Mount St. Helens)

Mourey, A.J., Shea, T., Costa, F., Shiro, B., Longman, R.J. Years of magma intrusion primed Kīlauea Volcano (Hawai‘i) for the 2018 eruption: evidence from olivine diffusion chronometry and monitoring data. *Bull. Volcanol.* **85**, 18 (2023). doi: 10.1007/s00445-023-01633-4 [earthquake frequency, GPS displacement]

Rae, A.S.P., Edmonds, M., Maclennan, J., Morgan, D., Houghton, B., Hartley, M.E., Sides, I. Time scales of magma transport and mixing at Kilauea Volcano, Hawai’i. *Geology* **44**, 463-466 (2016). [earthquake depths, tilt]

Saunders, K., Blundy, J., Dohmen, R., Cashman, K. Linking petrology and seismology at an active volcano. *Science* **336**, 1023-1027 (2012). [gas geochemistry, earthquake depth]

Diffusion Studies with Monitoring Comparisons (≥4 Data Types)

The following list is organized by volcano. The types of monitoring data used in comparisons are noted in brackets [ ] at the end of the citation and are defined as described above.

Kīlauea

Lynn, K.J., Nadeau, P.A., Ruth, D.C.S., Chang, J., Dotray, P.J. & Johanson, I.A. Olivine diffusion constrains months-scale magma transport within Kīlauea volcano’s summit reservoir system prior to the 2020 eruption. *Bull. Volcanol.* **86**, 31 (2024). doi: 10.1007/s00445-024-01714-y [tilt, GPS line lengths, gas geochemistry, earthquake frequency, earthquake location]

Mauna Loa (this study)

Lynn, K.J., Downs, D.T., Trusdell, F.A., Wieser, P.E., Rangel, B., McDade, B., Hotovec-Ellis, A.J., Bennington, N., Anderson, K.R., Ruth, D.C.S., DeVitre, C., Ellis, A.P., Nadeau, P.A., Clor, L., Kelly, P., Dotray, P.J., Chang, J.C. Triggering an eruption at Mauna Loa, Earth’s largest volcano. *Nat. Comm.* . [earthquake frequency, earthquake location, GPS line length, seismic velocity change, tilt, gas geochemistry]

Mount Etna

Giuffrida, M., Scandura, M., Costa, G., Zuccarello, F., Sciotto, M., Cannata, A., Viccaro, M. Tracking the summit activity of Mt. Etna volcano between July 2019 and January 2020 by

integrating petrological and geophysical data. *J. Volcanol. Geotherm. Res.* **418**, 107350 (2021). doi: 10.1016/j.jvolgeores.2021.107350 [tremor source position, tremor amplitude, tremor event frequency, infrasound]

Viccaro, M., Zuccarello, F., Cannata, A., Palano, M., Gresta, S. How a complex basaltic volcanic system works: Constraints from integrating seismic, geodetic, and petrological data at Mount Etna volcano during the July-August 2014 eruption. *J. Geophys. Res. Solid Earth* **121**, 5659-5678, 6717 (2016b). doi: 10.1002/2016JB013164 [GPS line lengths, geodetic velocities, tremor amplitude, tremor source position]

Piton de la Fournaise

Sundermeyer, C., Di Muro, A., Gordeychik, B., Wörner, G. Timescales of magmatic processes during the eruptive cycle 2014-2015 at Piton de la Fournaise, La Réunion, obtained from Mg-Fe diffusion modeling in olivine. *Contrib. Mineral. Petrol.* **175**, 1 (2020). doi: 10.1007/s00410-019-1642-y [earthquake frequency, earthquake depth, gas geochemistry, GPS displacement]

Mount Ruapehu

Kilgour, G.N., Saunders, K.E., Blundy, J.D., Cashman, K.V., Scott, B.J., Miller, C.A. Timescales of magmatic processes at Ruapehu volcano from diffusion chronometry and their comparison to monitoring data. *J. Volcanol. Geotherm. Res.* **288**, 62-75 (2014). [water lake chemistry, earthquake frequency, earthquake depth, earthquake magnitude, gas geochemistry]

The following slides show images of fluid inclusions which were individually mounted in crystalbond, and then prepared within an epoxy mount for EPMA. The first set of images are designed to show the approximate position of the FI in crystals. The slide with a single image is a zoomed in image taken on the Raman of the FI – with dimensions of the image given.

ML22_1 – Good diad, SO2

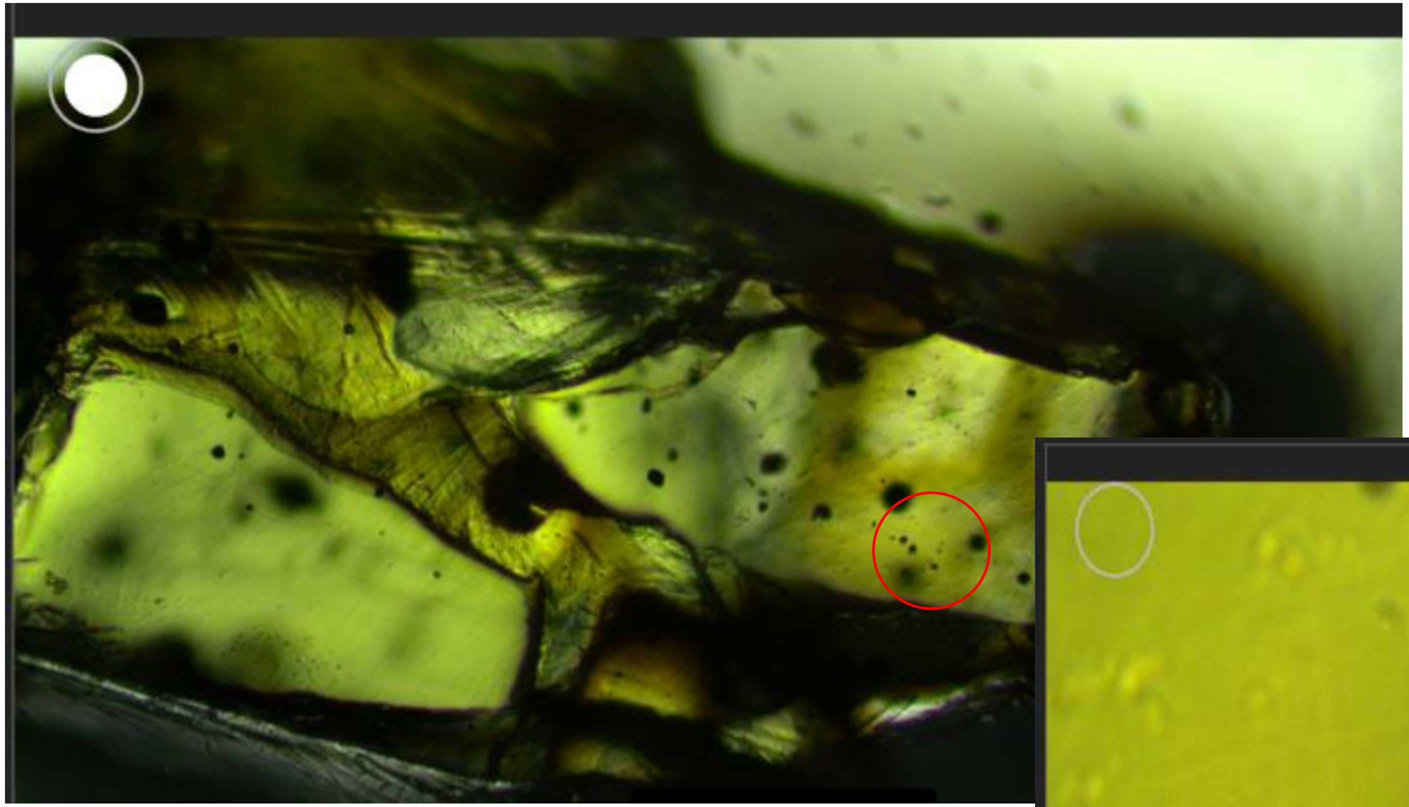

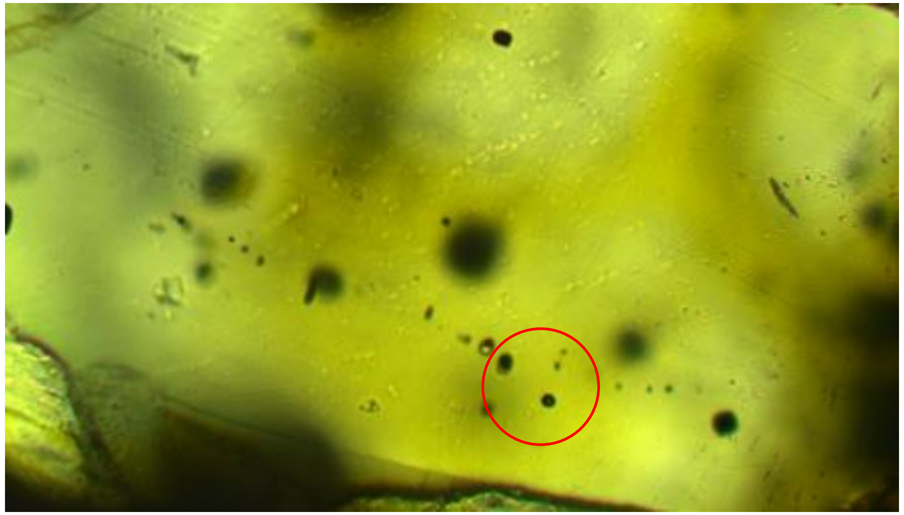

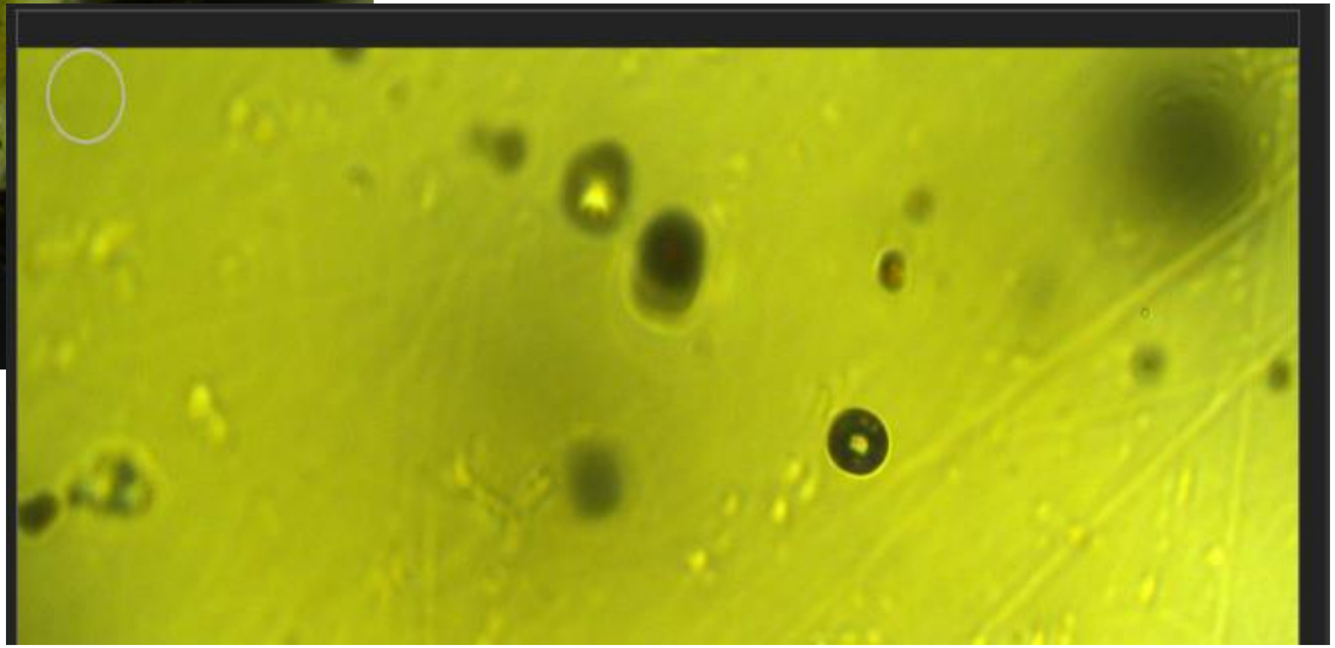

ML22_1

Mag	Width (µm)	Height (µm)
50	223.221	139.513

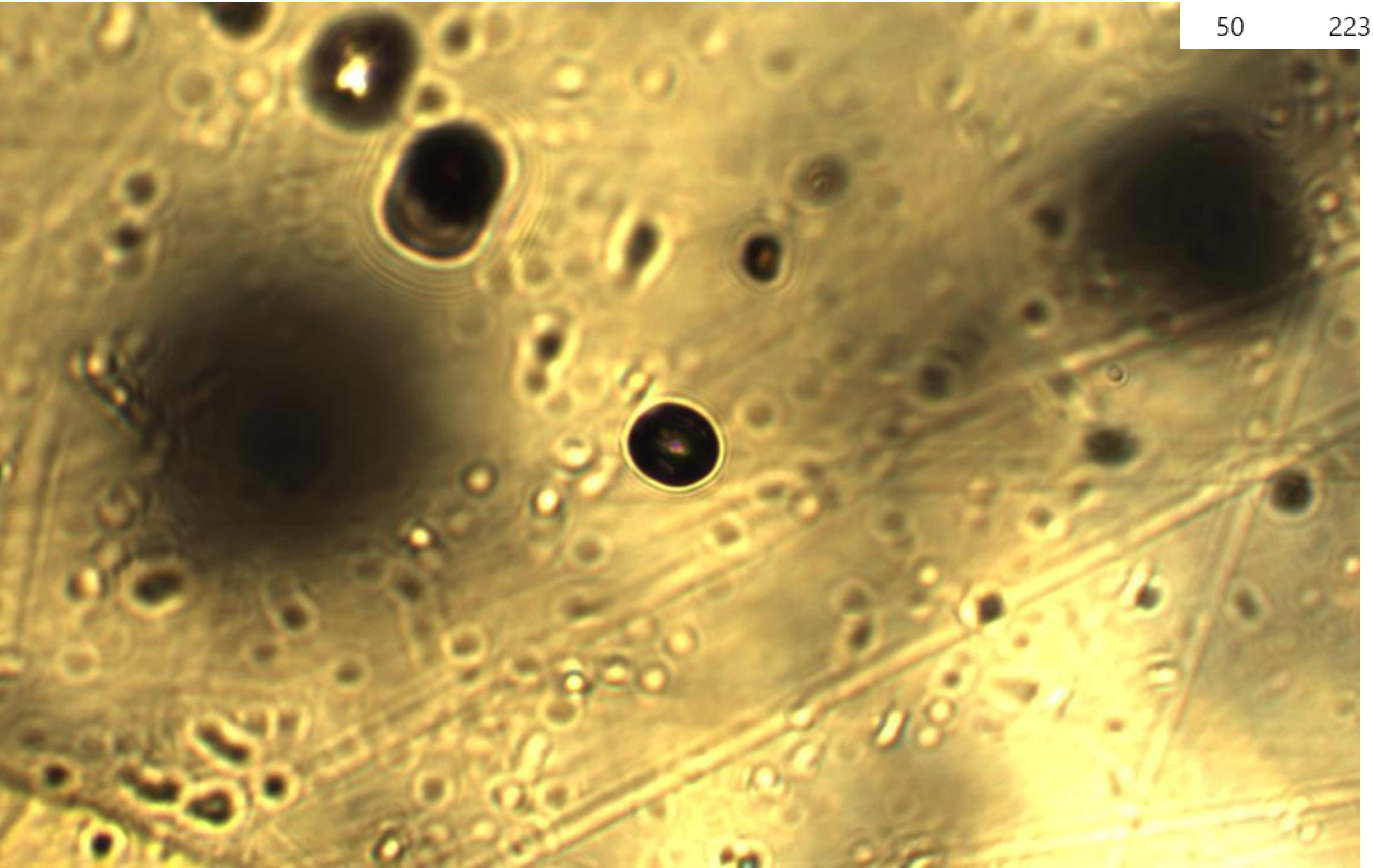

ML22_2_FIA

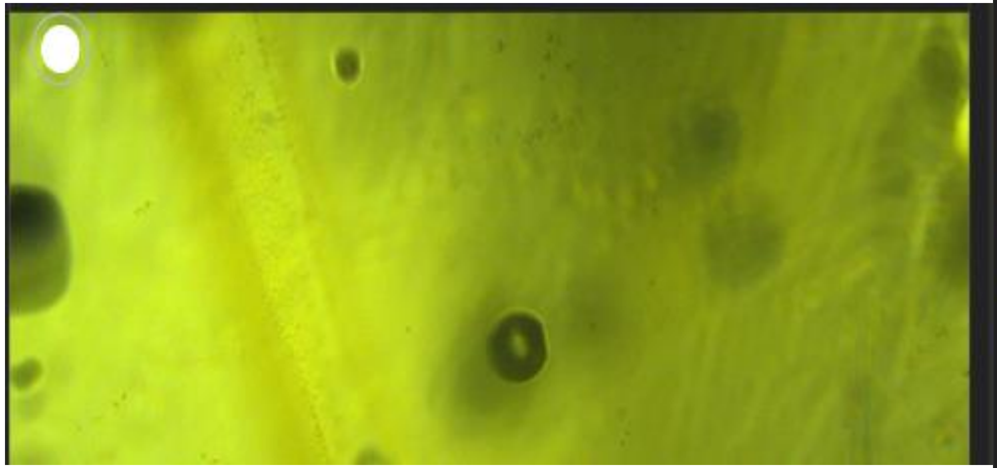

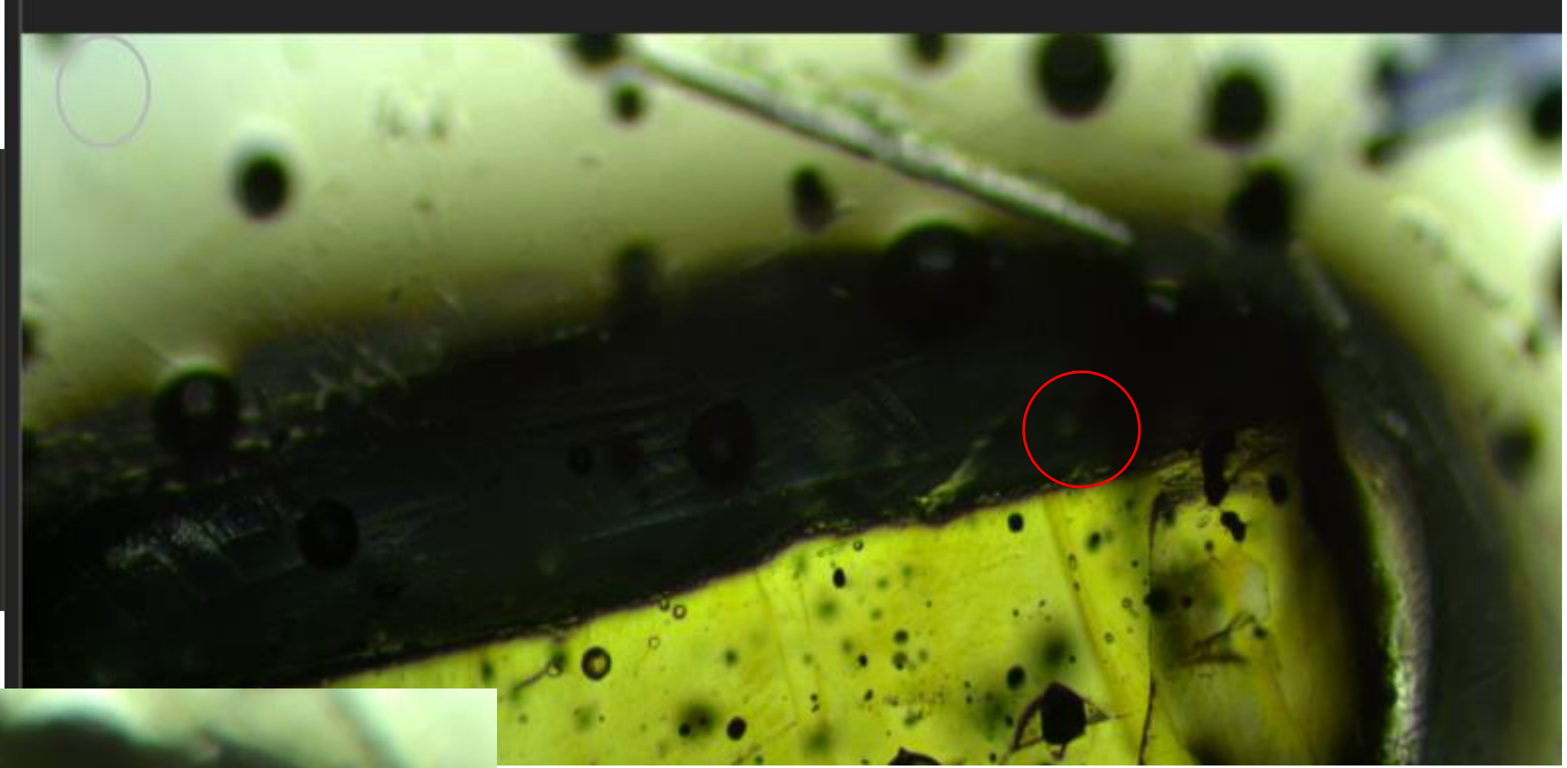

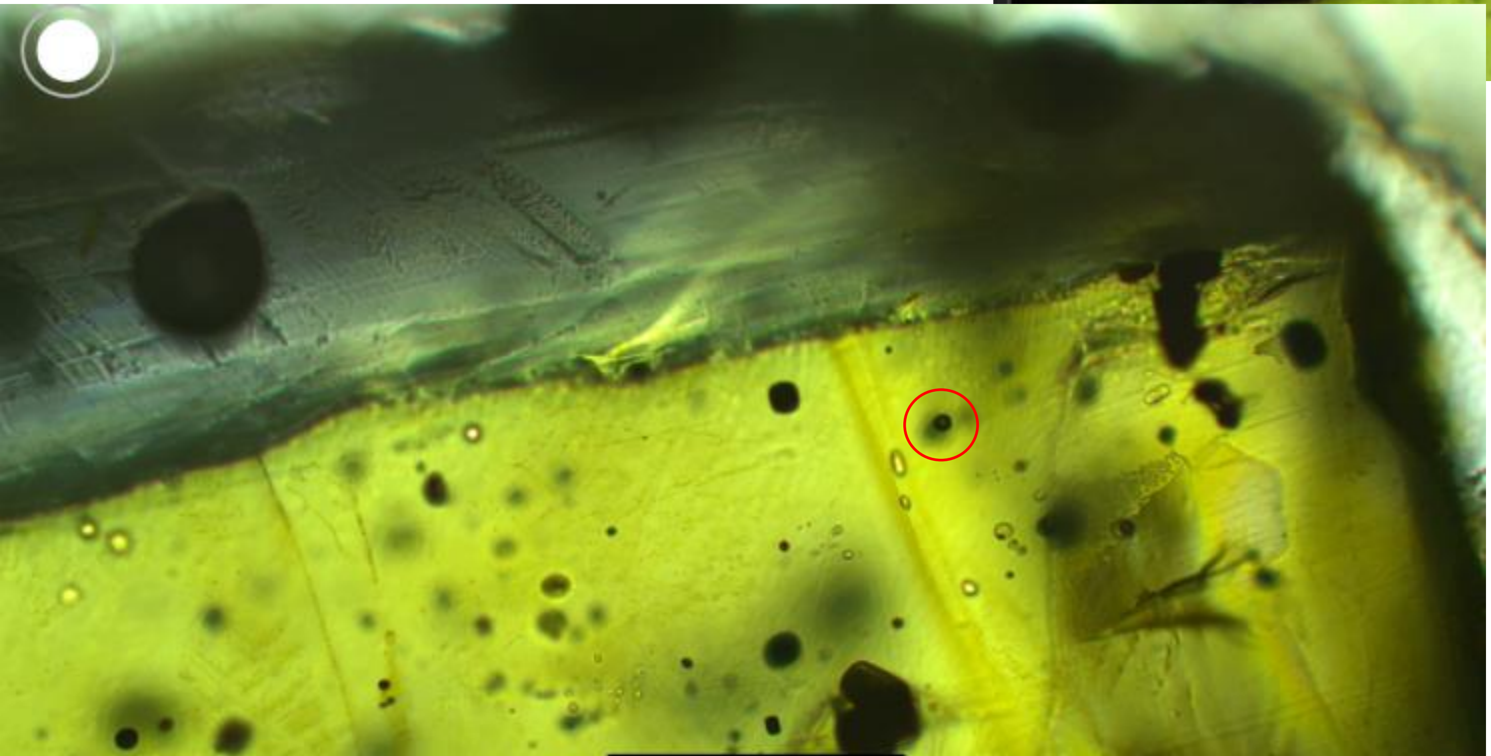

ML22_2_FIA

Mag	Width (µm)	Height (µm)
[illegible]	[illegible]1	139.513

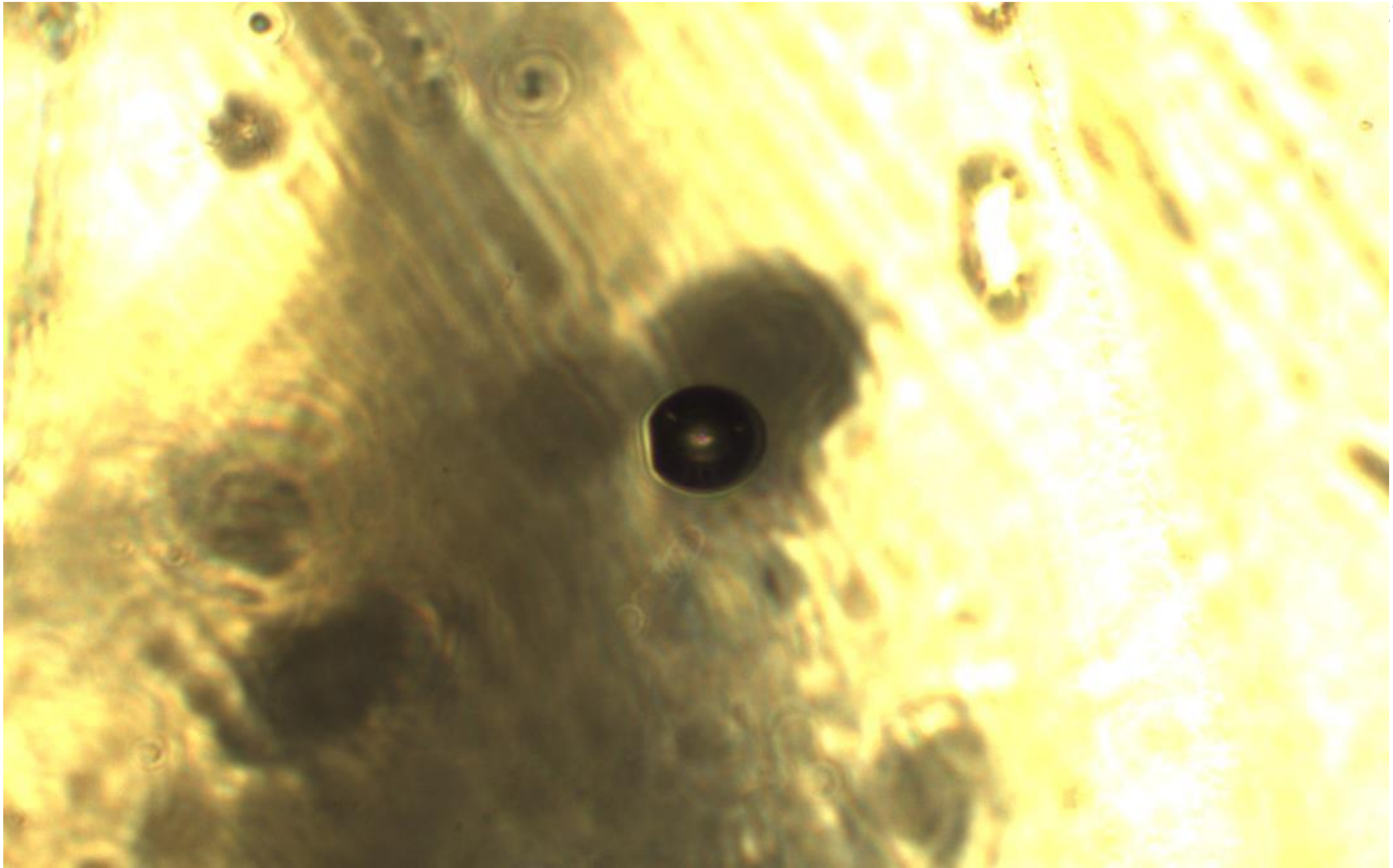

ML22_2_FIB

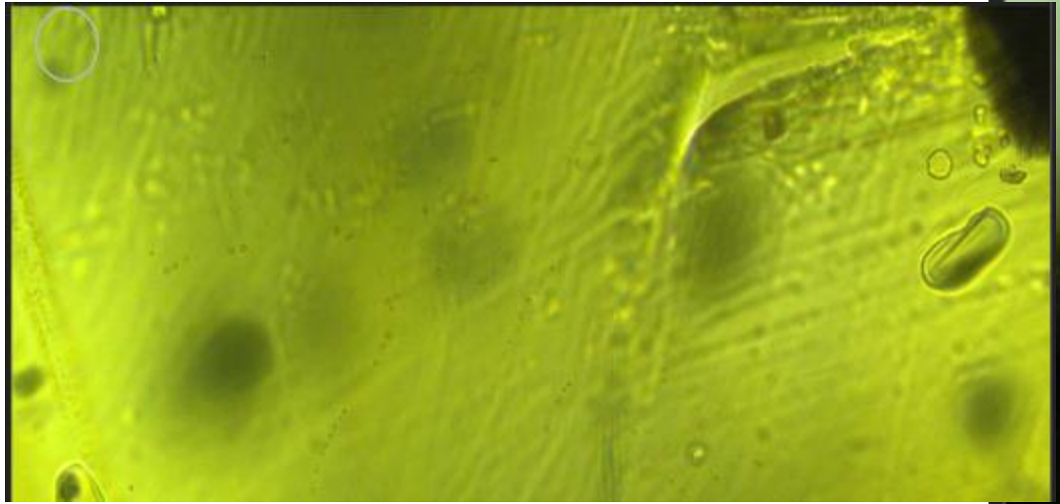

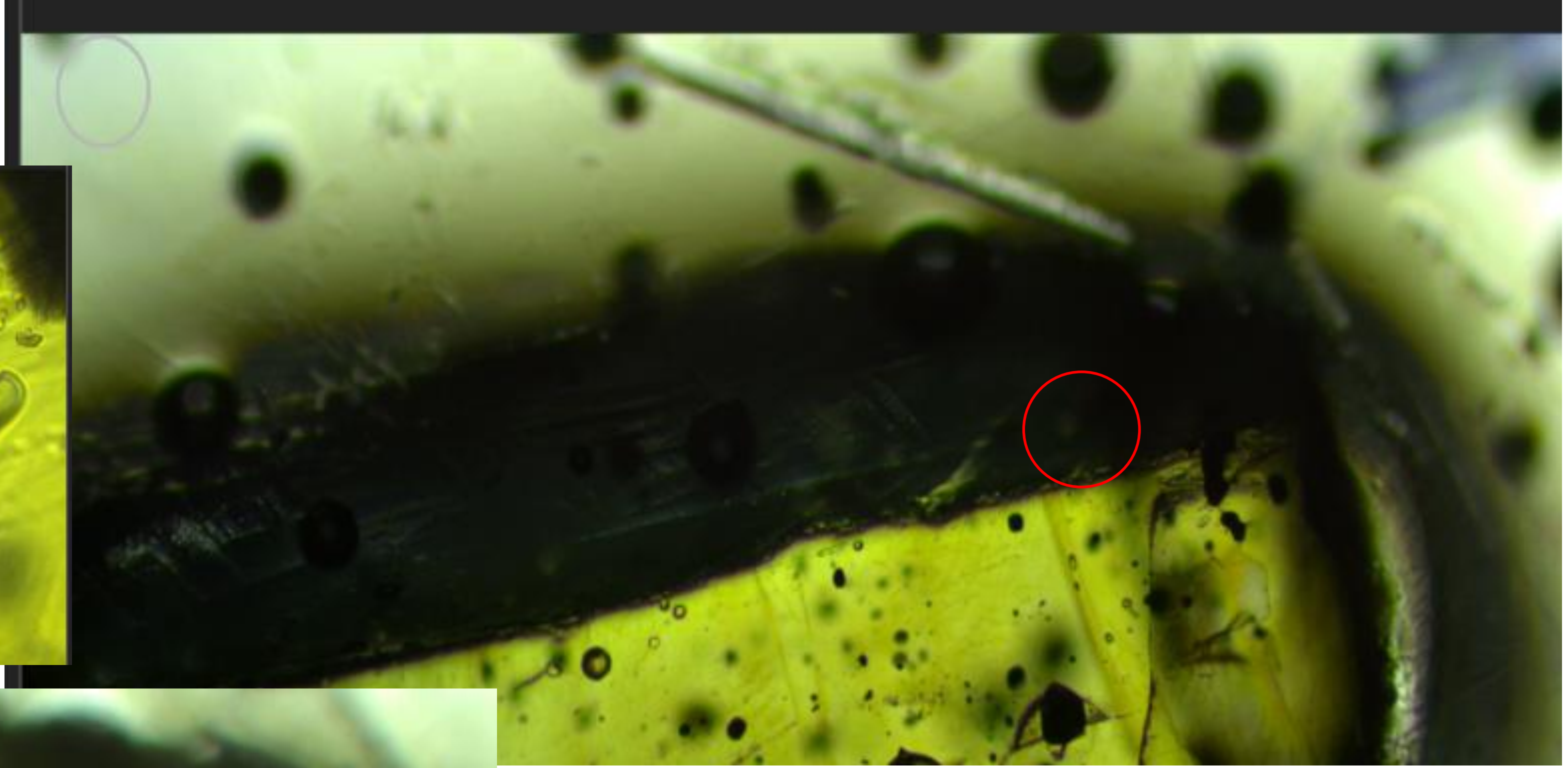

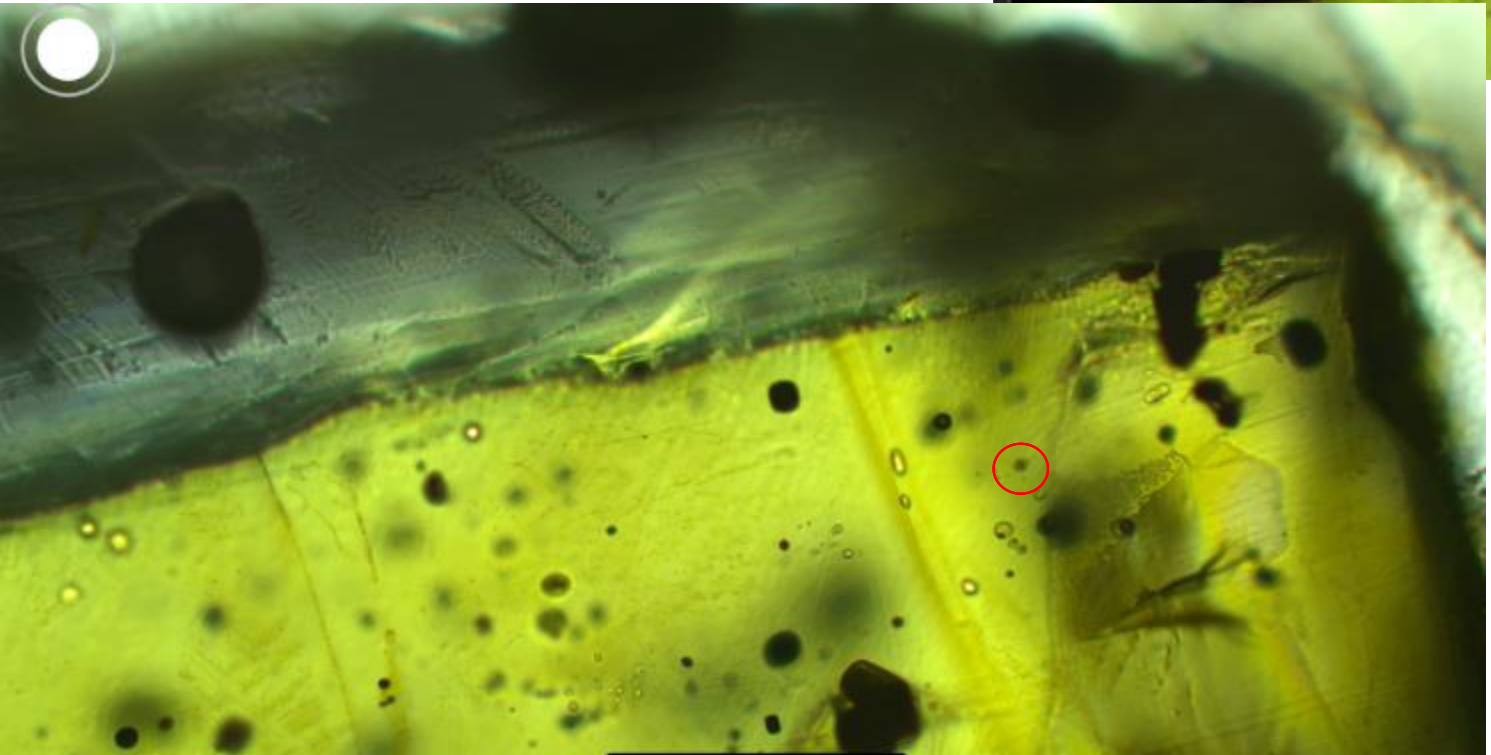

ML22_2_FIB

Mag	Width (µm)	Height (µm)
50	223.221	139.513

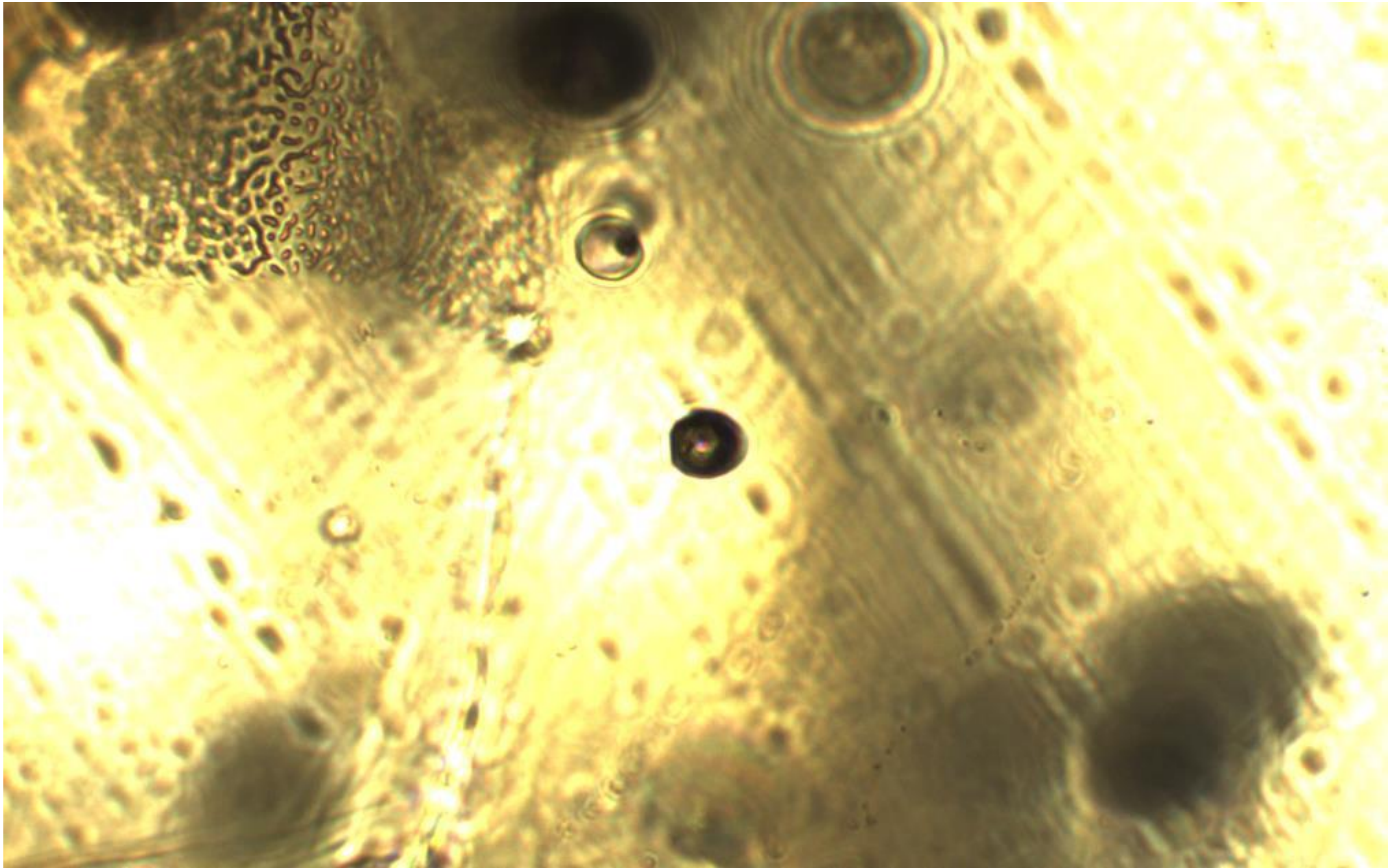

L22_2_FID

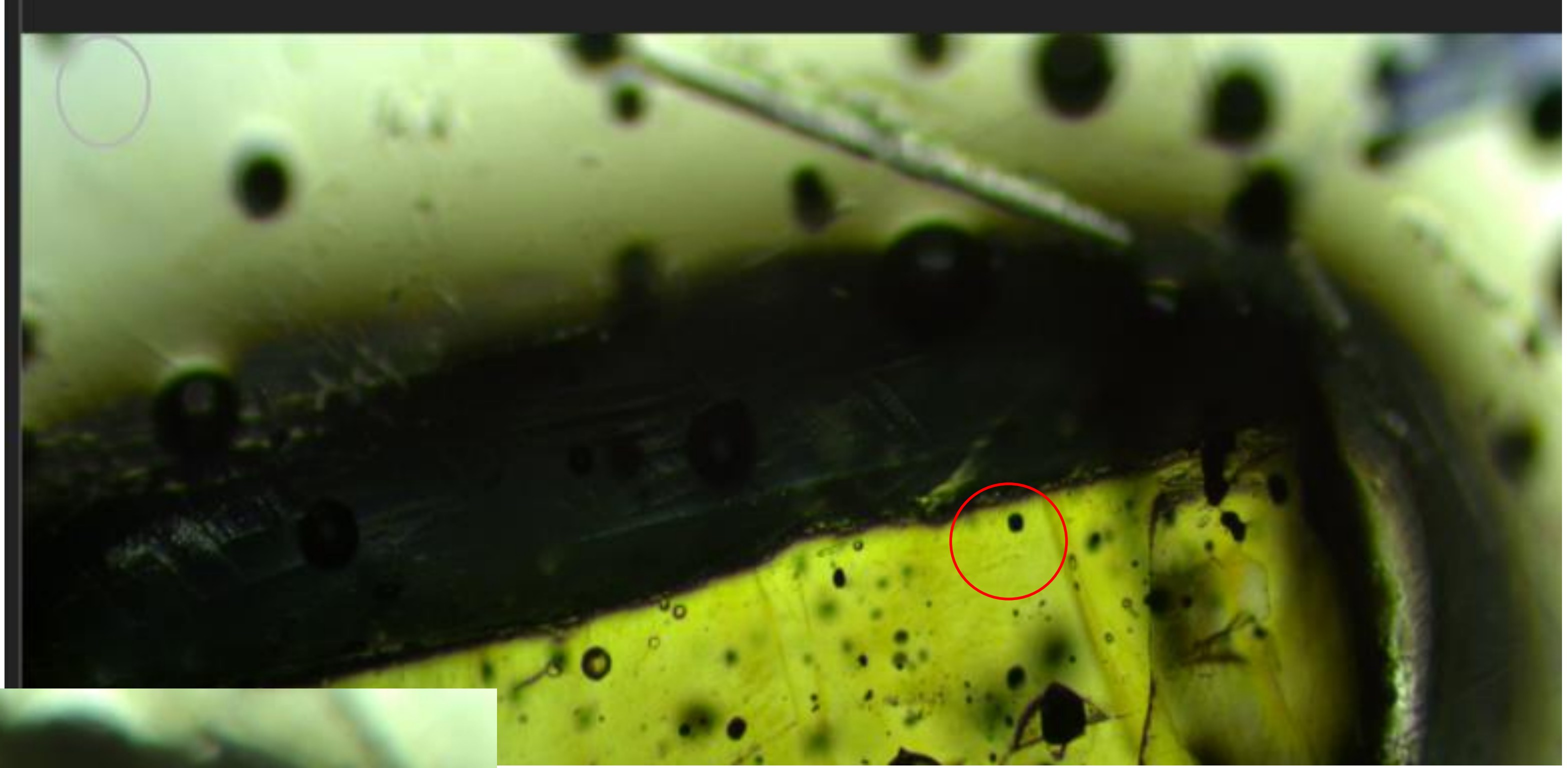

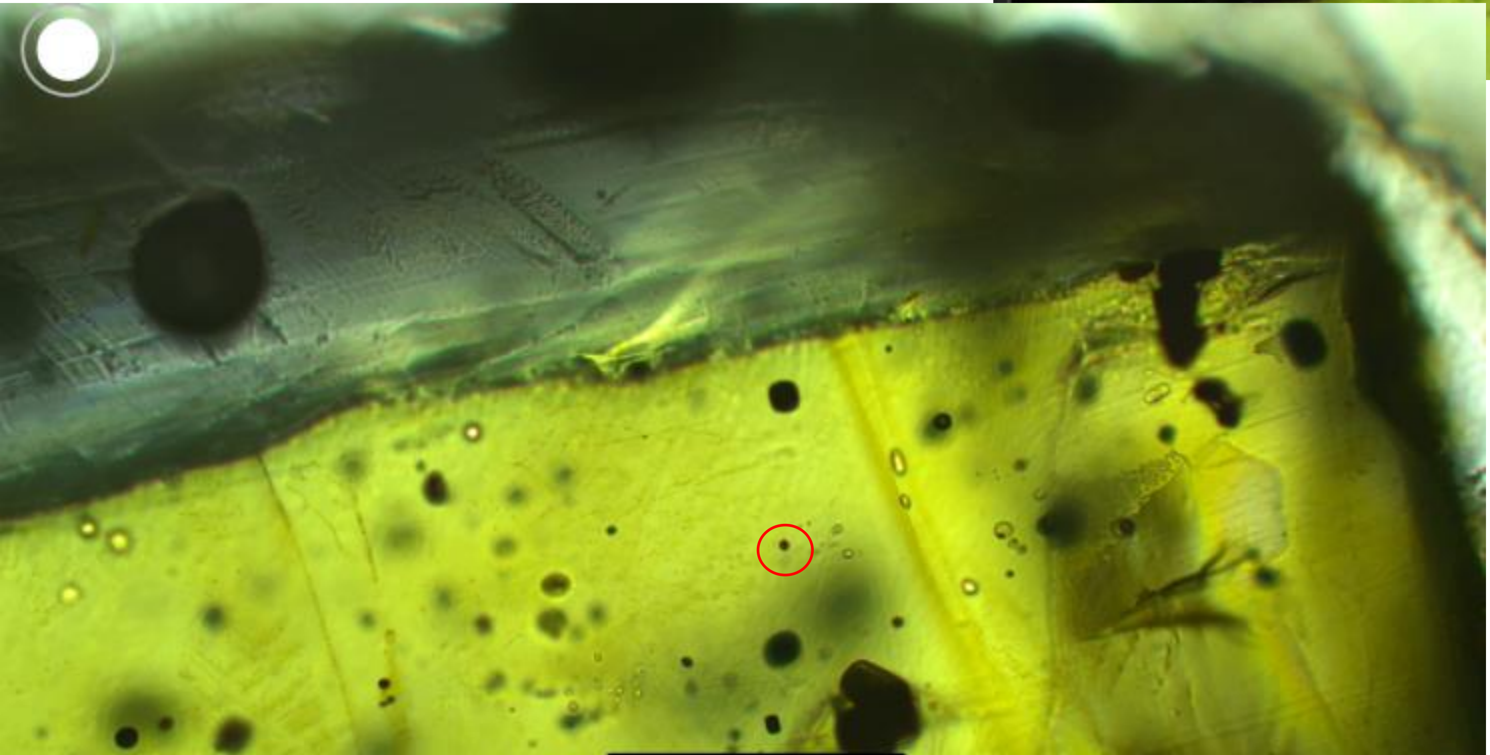

ML22_2_FID

Mag	Width (µm)	Height (µm)
50	223.221	139.513

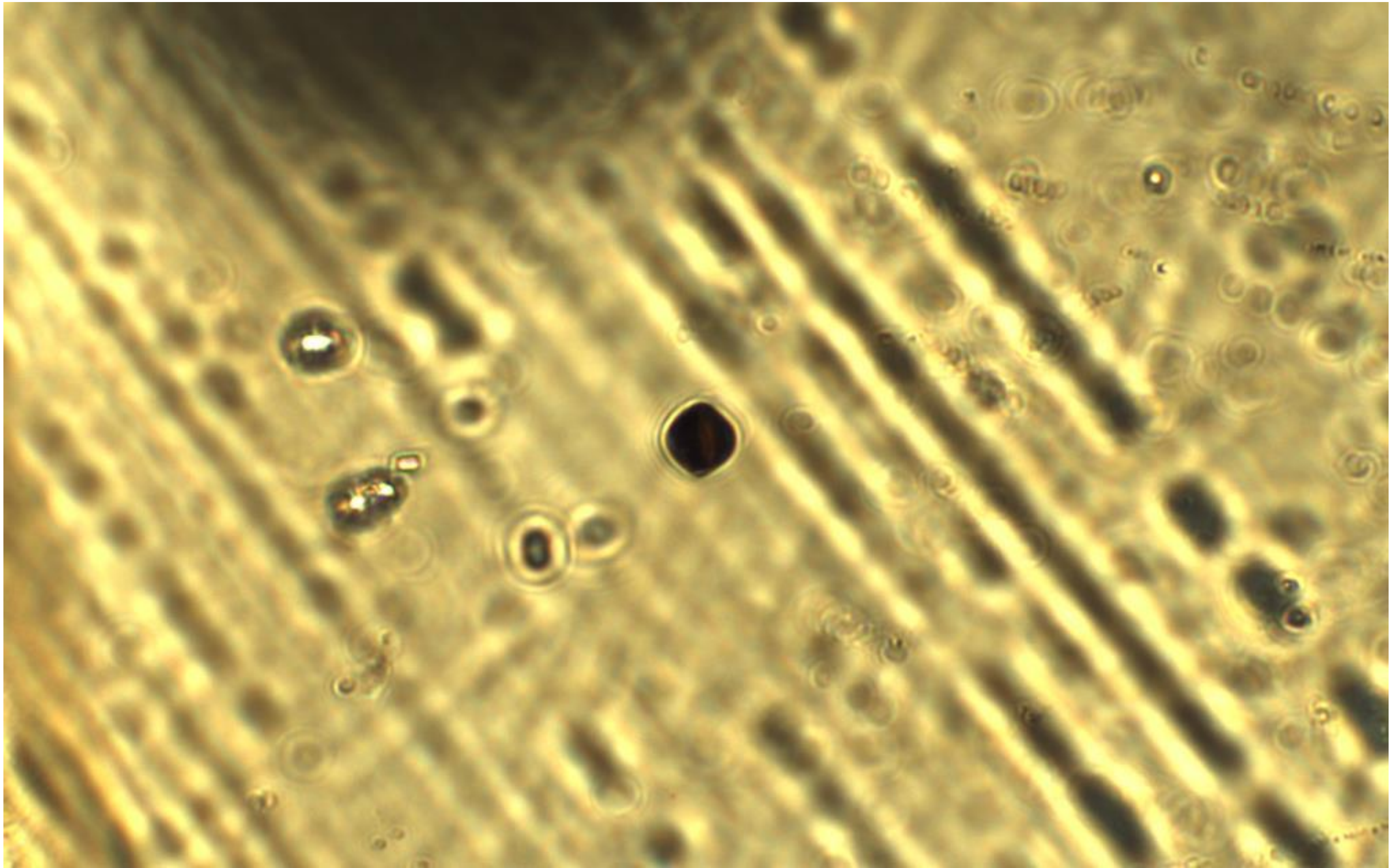

ML22_13

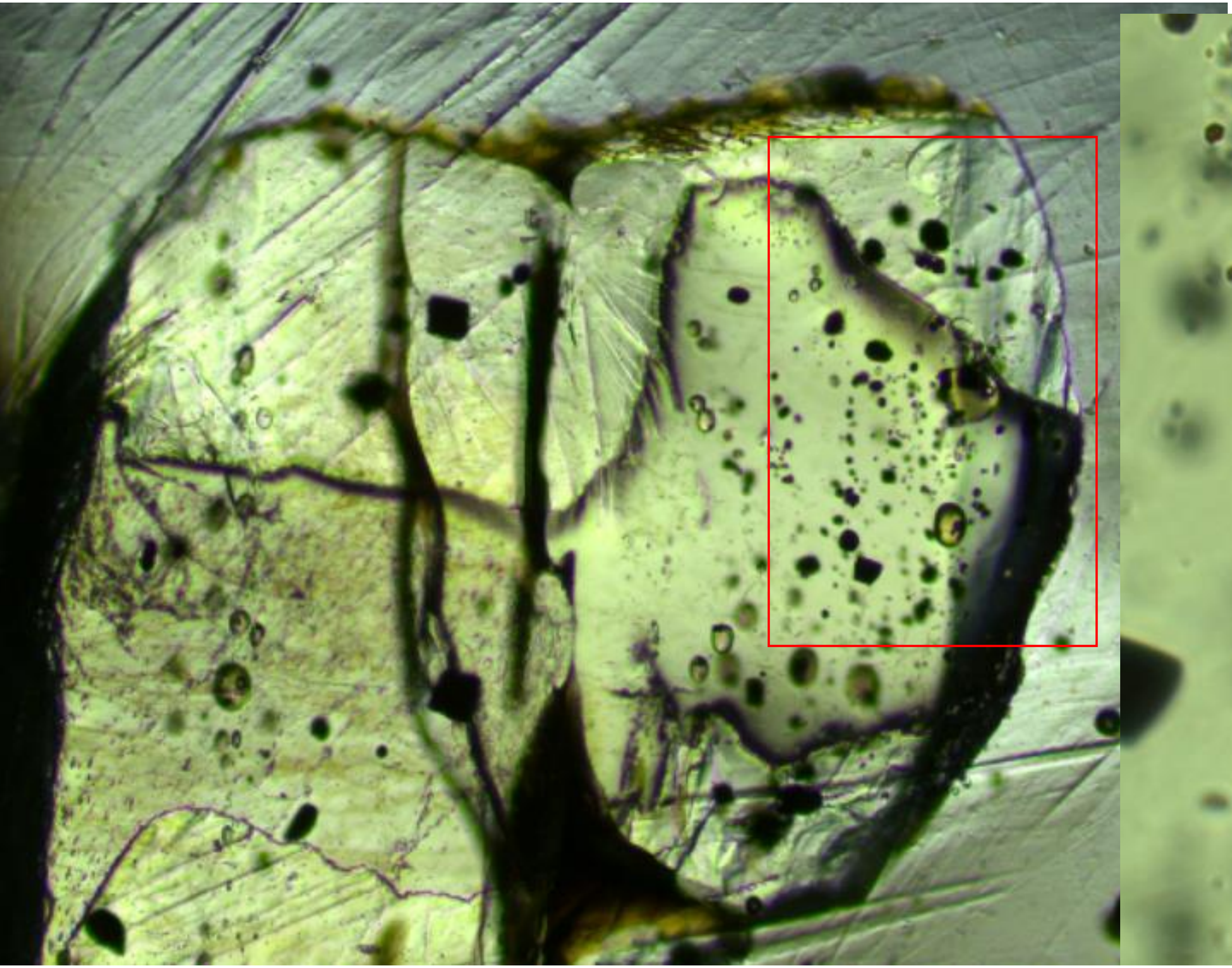

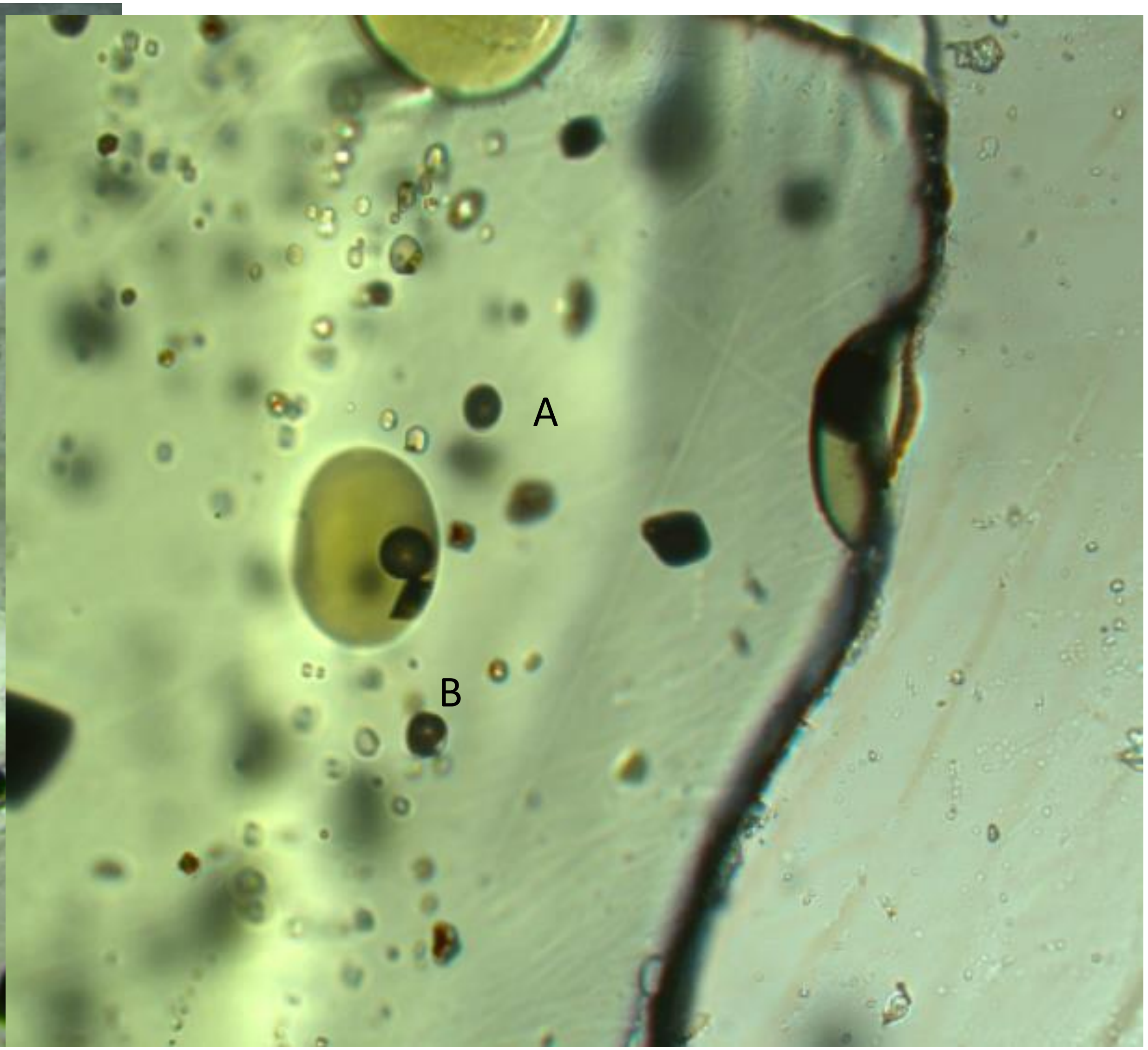

C

ML22_13_FIA

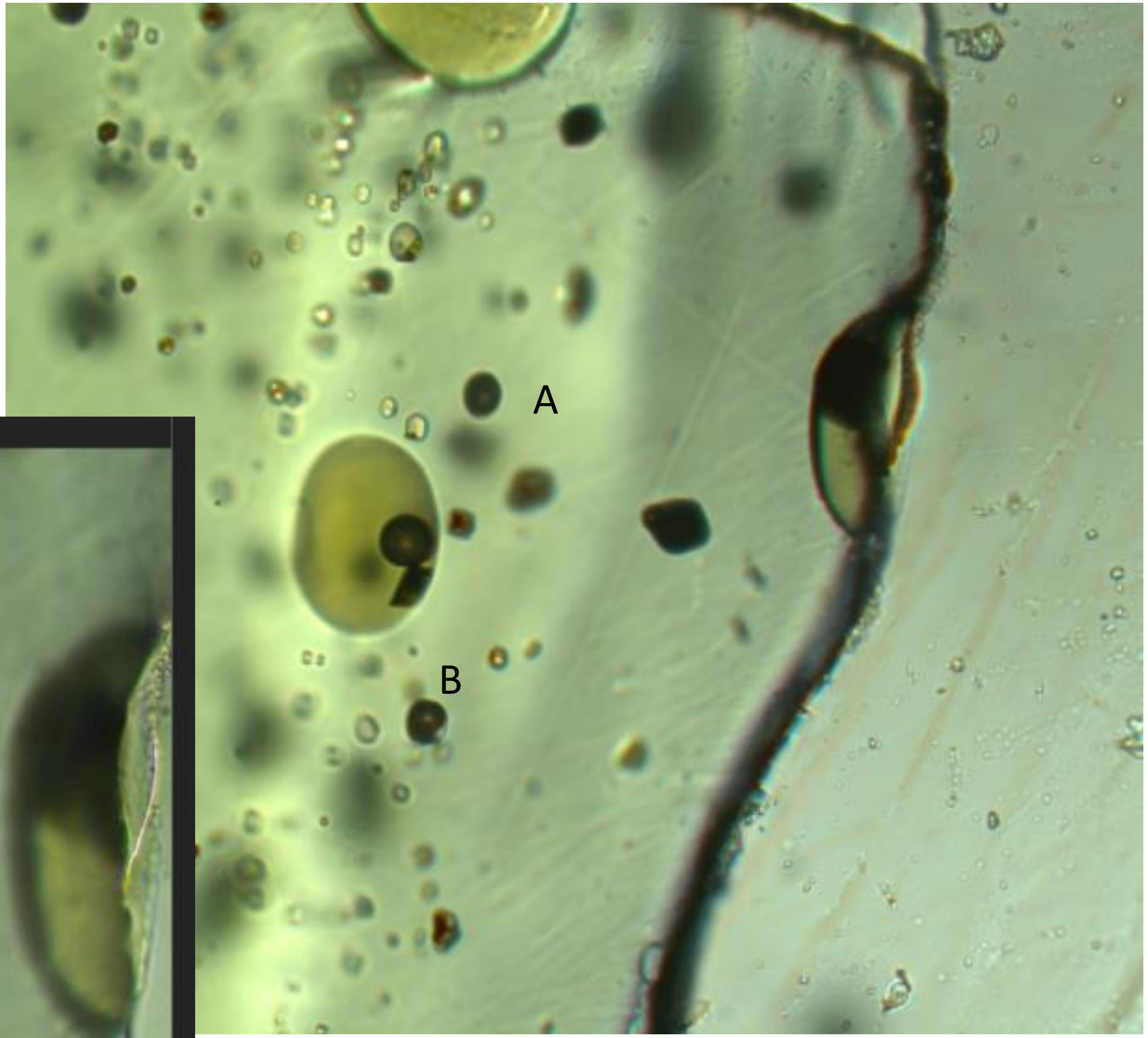

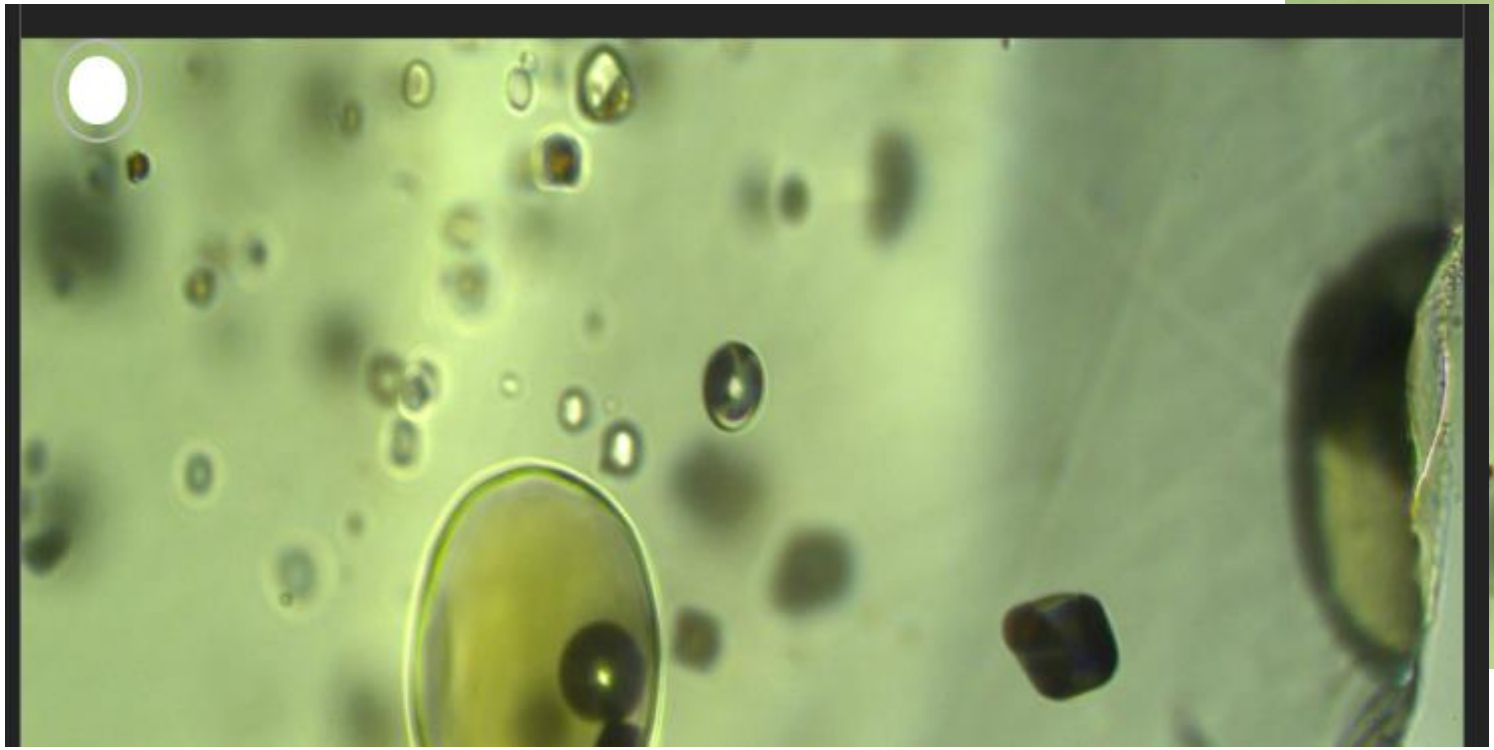

C

ML22_13_FIA

Mag	Width (µm)	Height (µm)
50	223.221	139.513

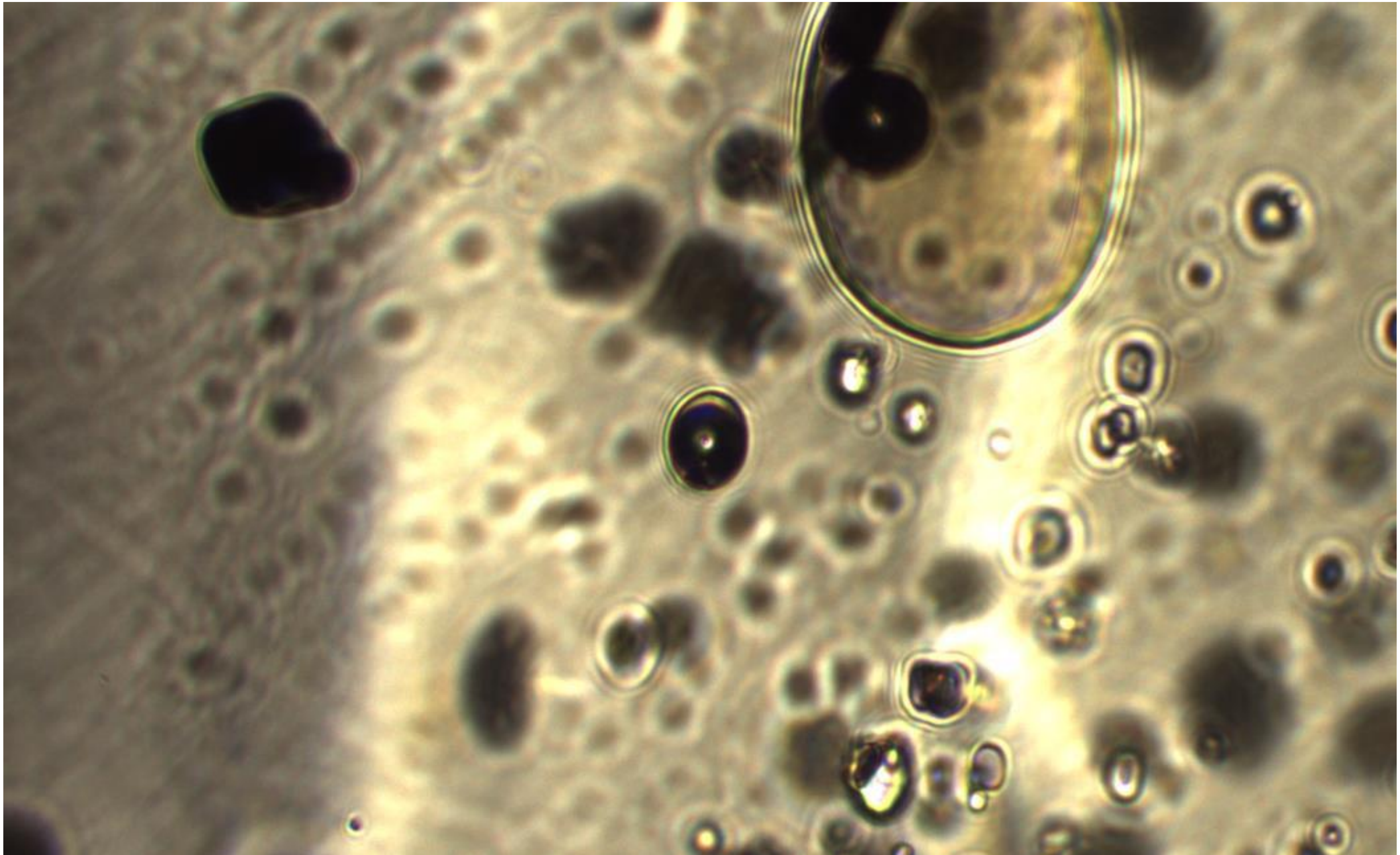

ML22_13_FIB

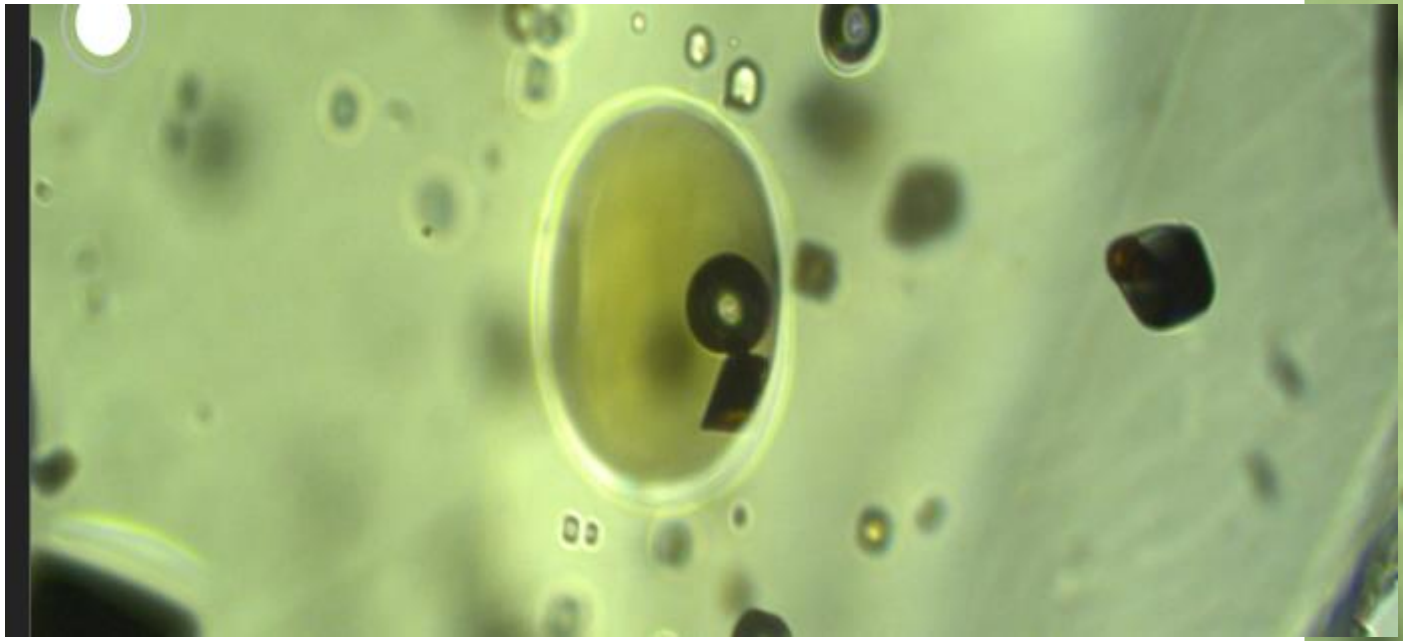

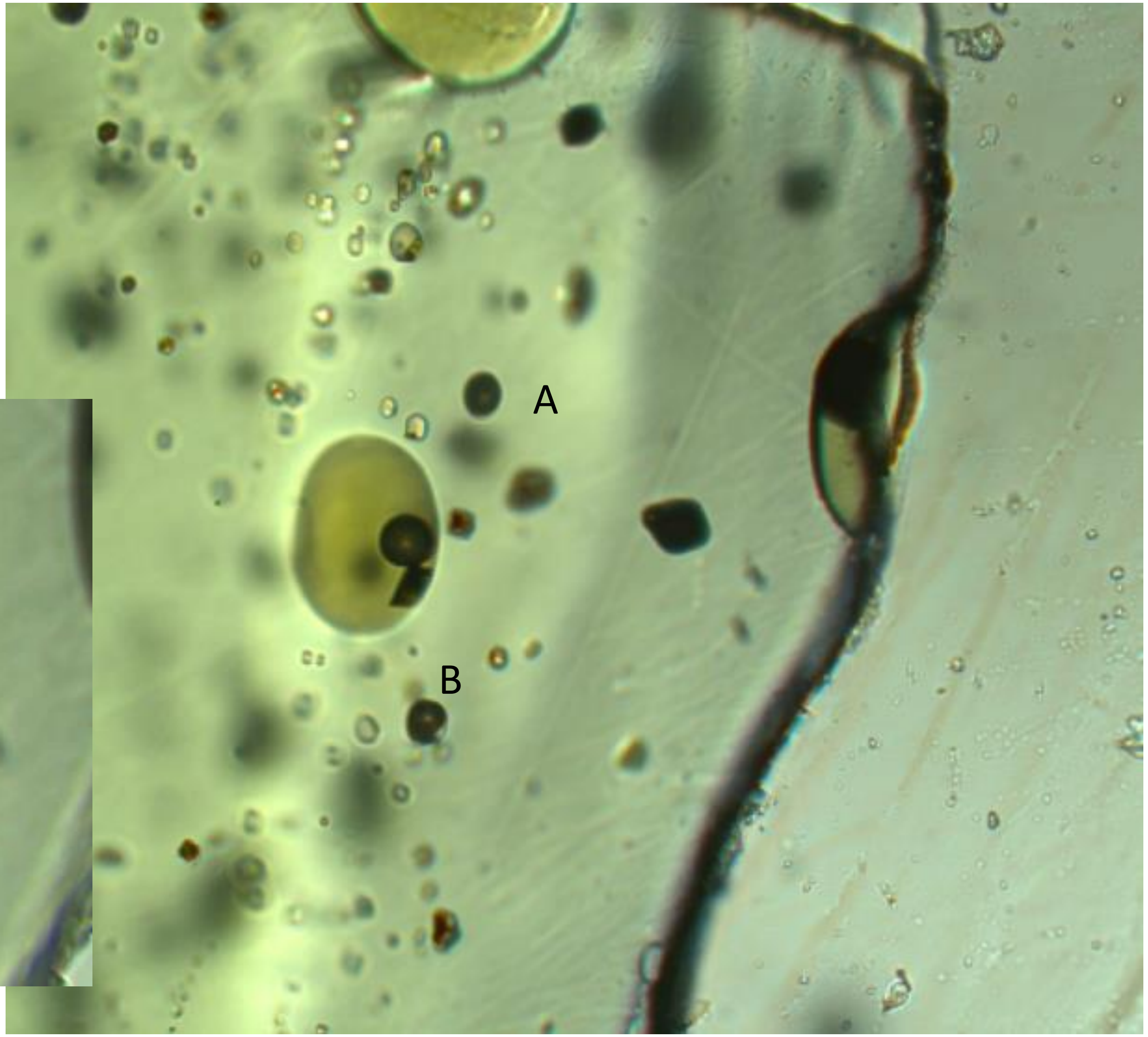

C

ML22_13_FIB

Mag	Width (µm)	Height (µm)
50	223.221	139.513

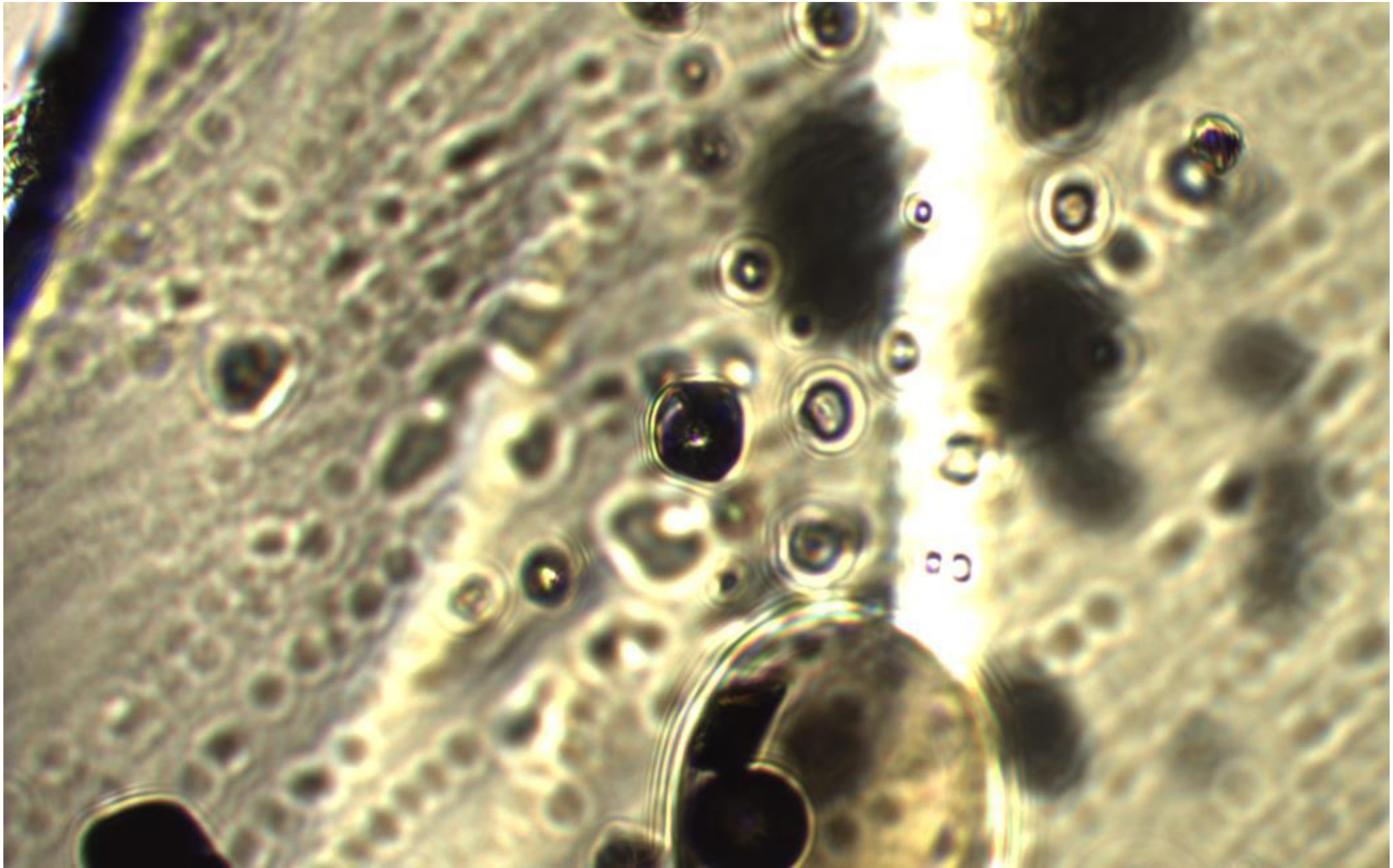

ML22_16_level1

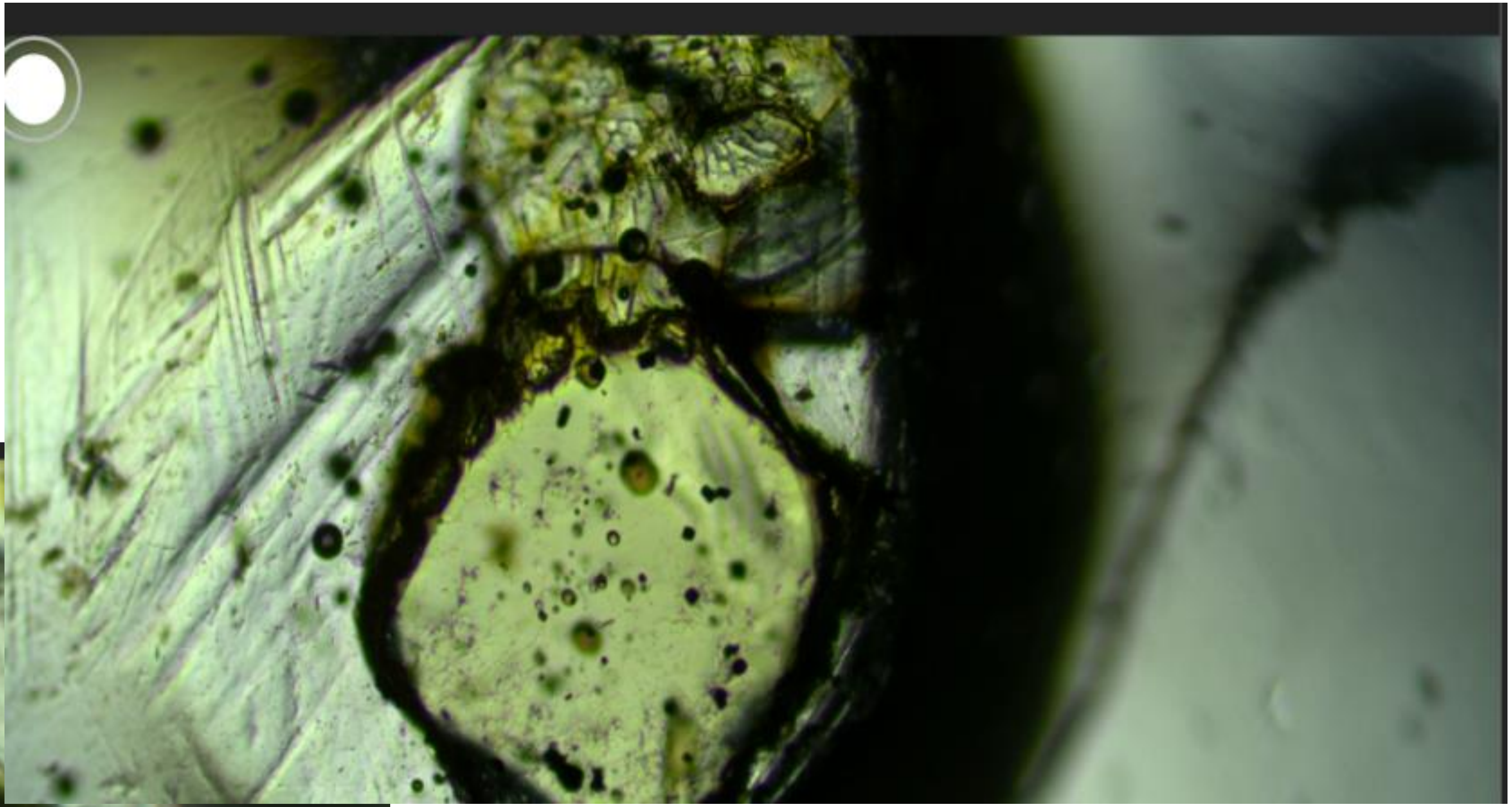

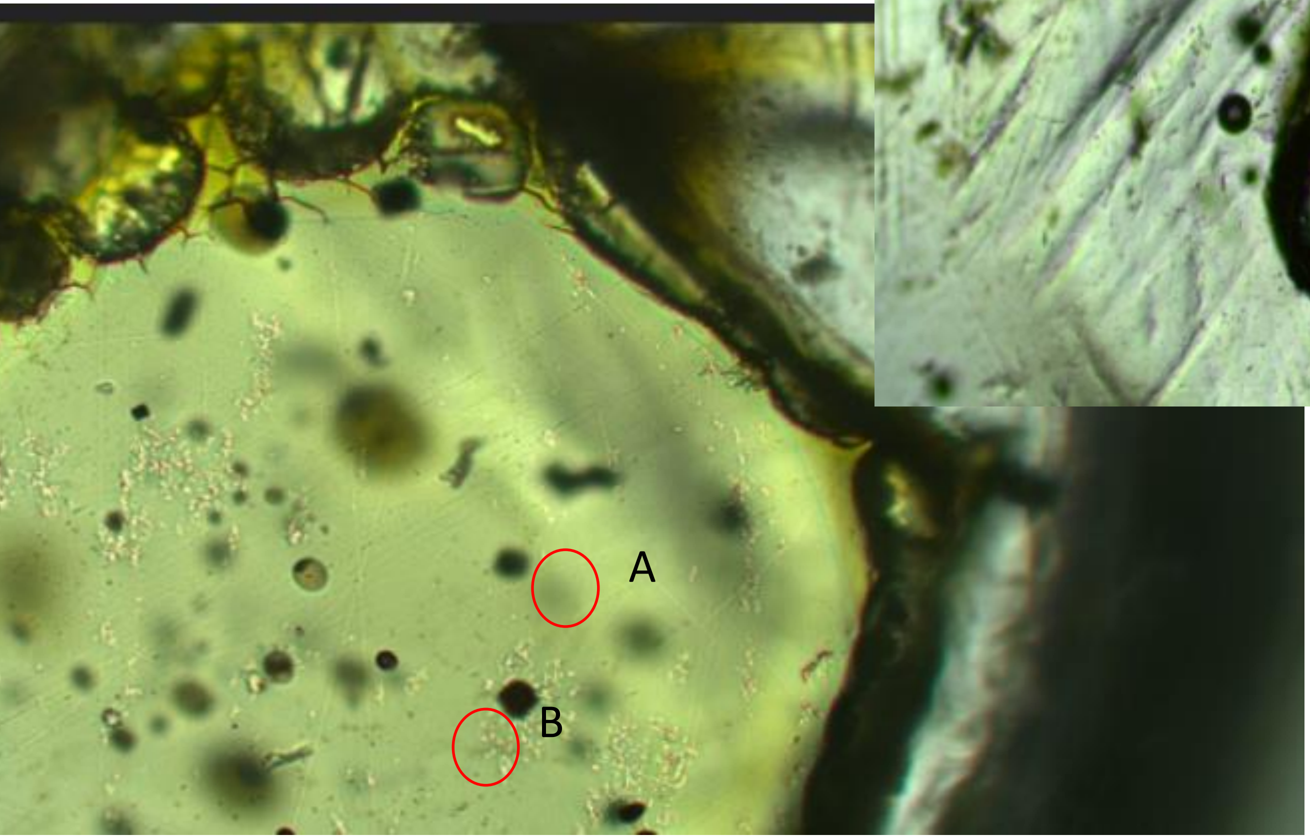

ML22_16_level1_FIA

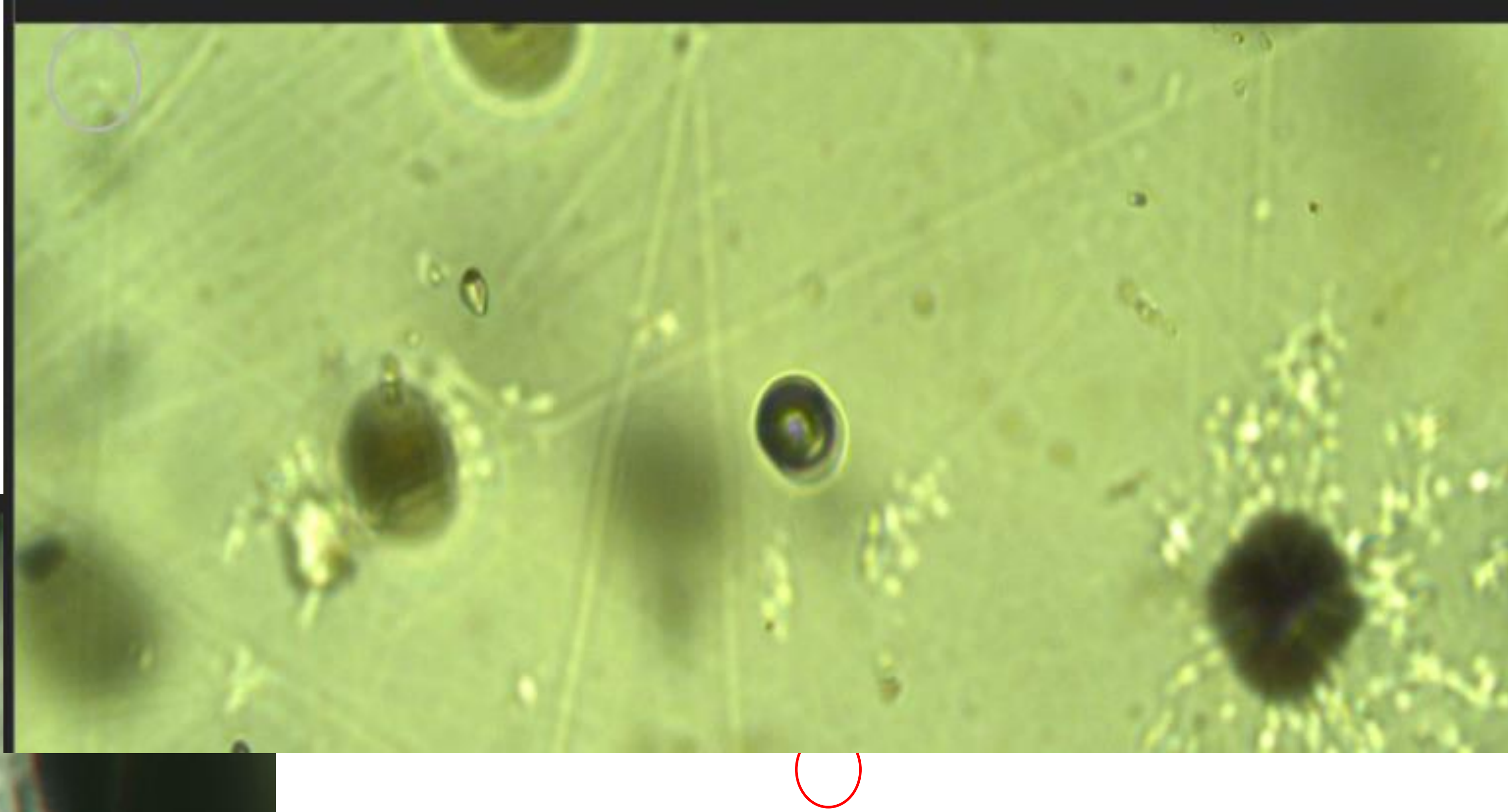

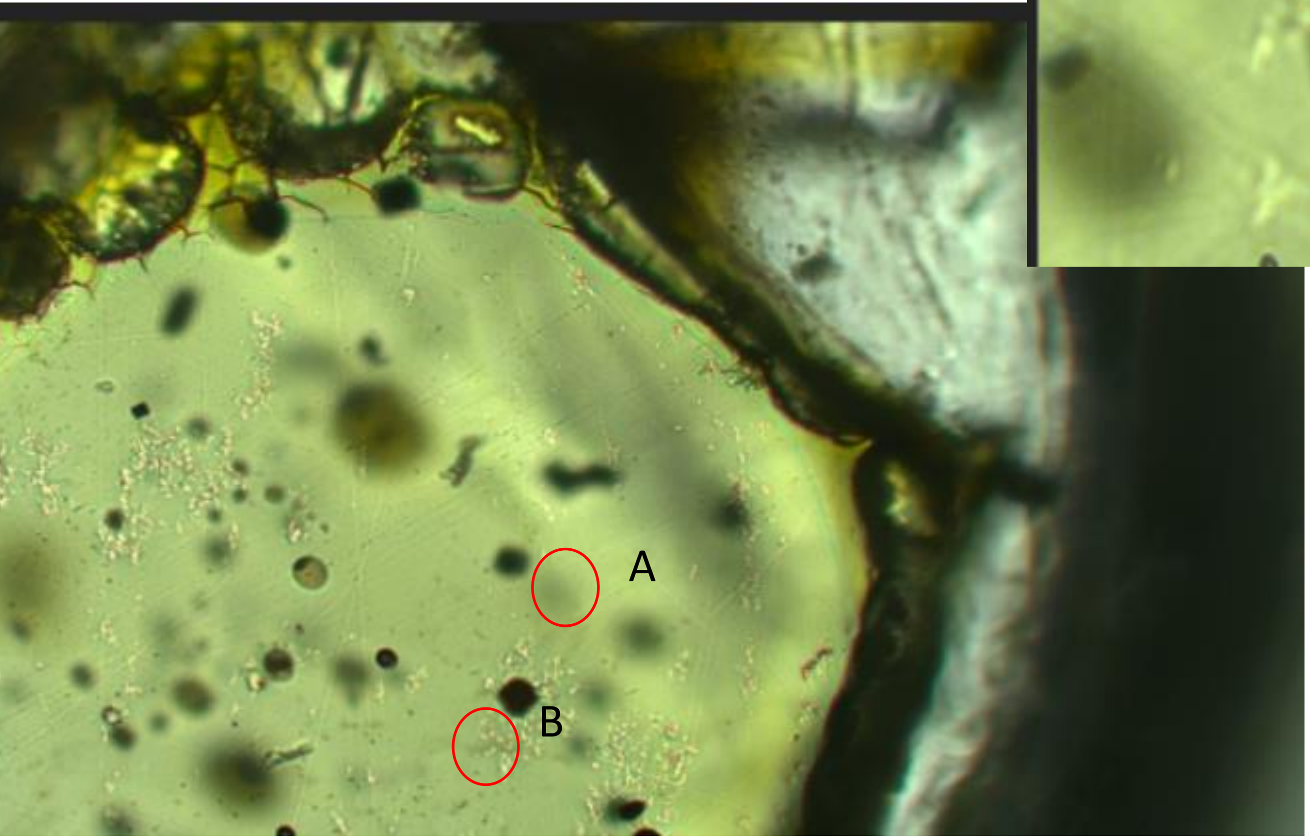

ML22_16_FIA

Mag	Width (µm)	Height (µm)
50	223.221	139.513

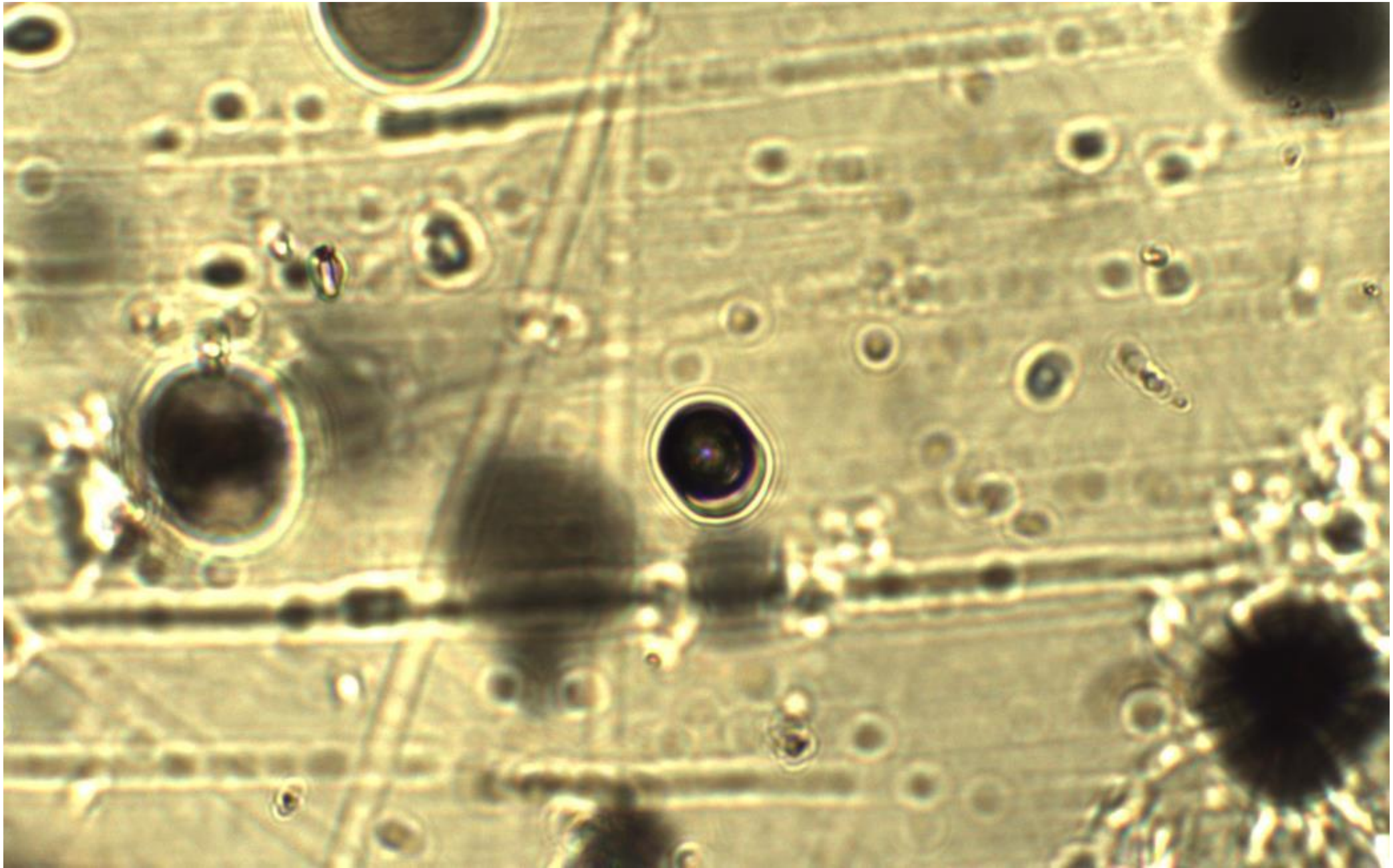

ML22_16_level1_FIB

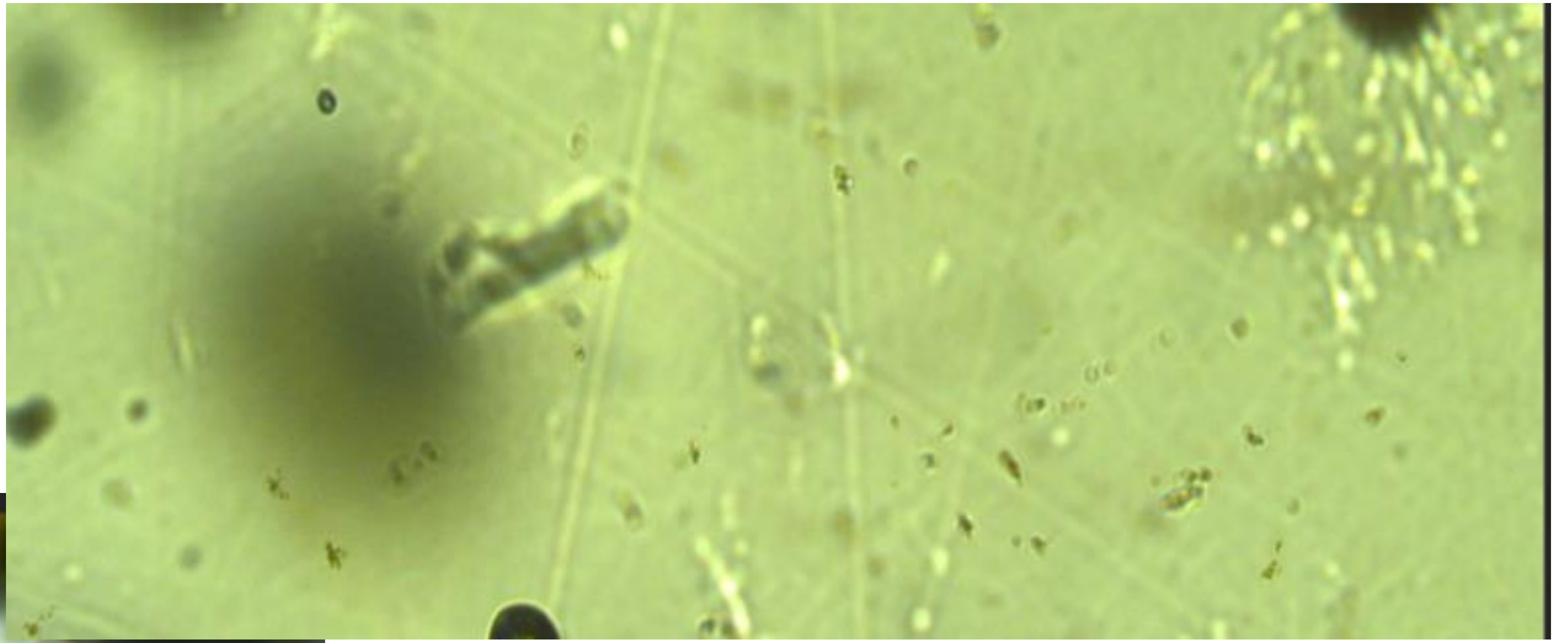

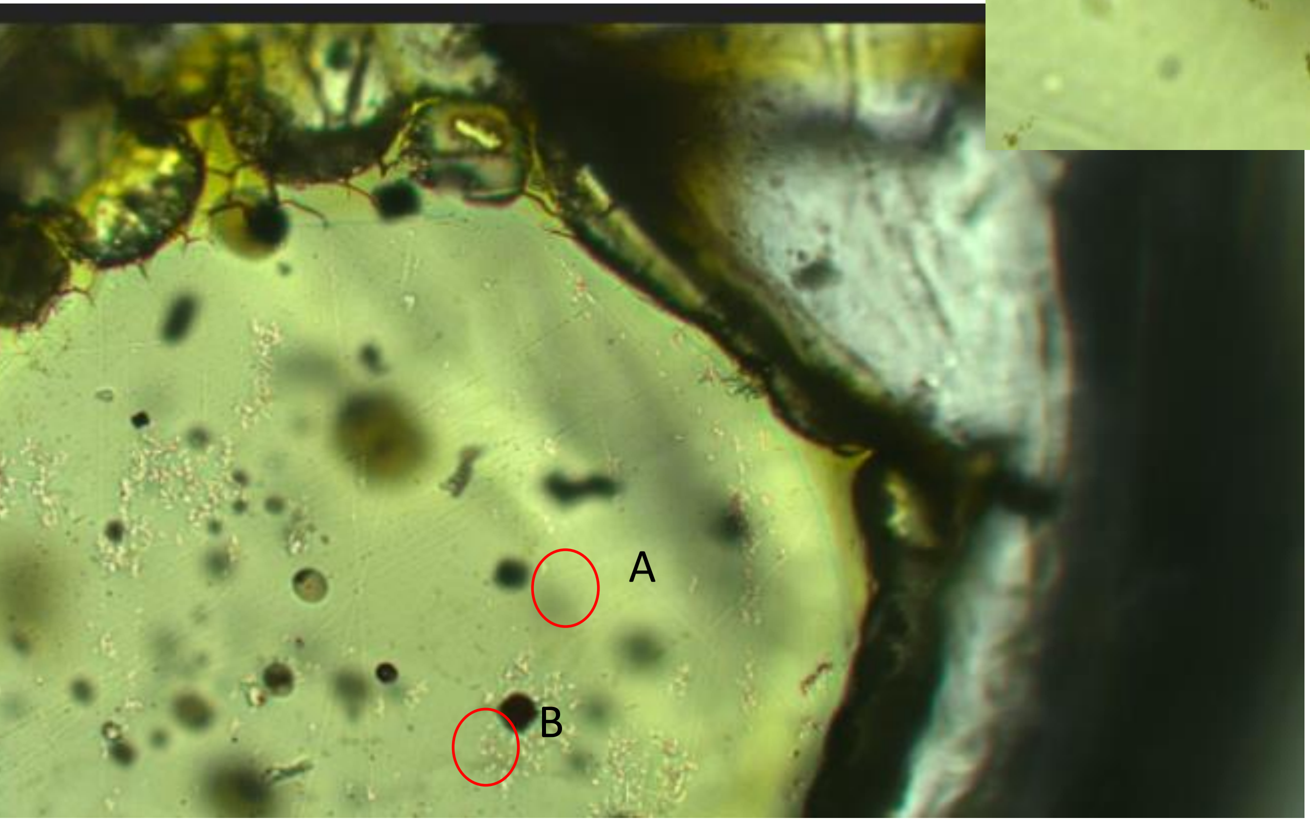

ML22_16_FIB

Mag	Width (µm)	Height (µm)
50	223.221	139.513

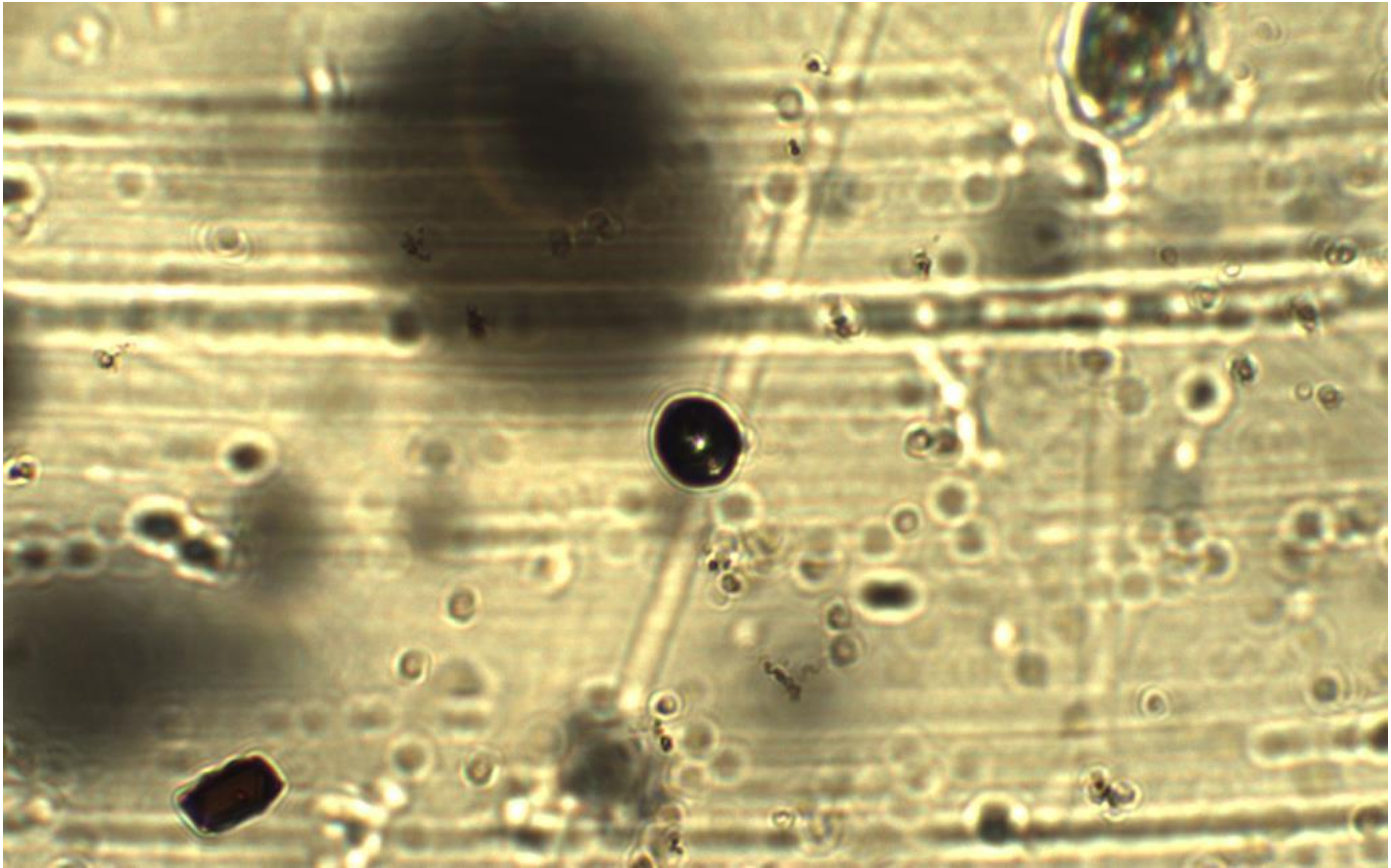

ML22_16_level2_FIC - deeper

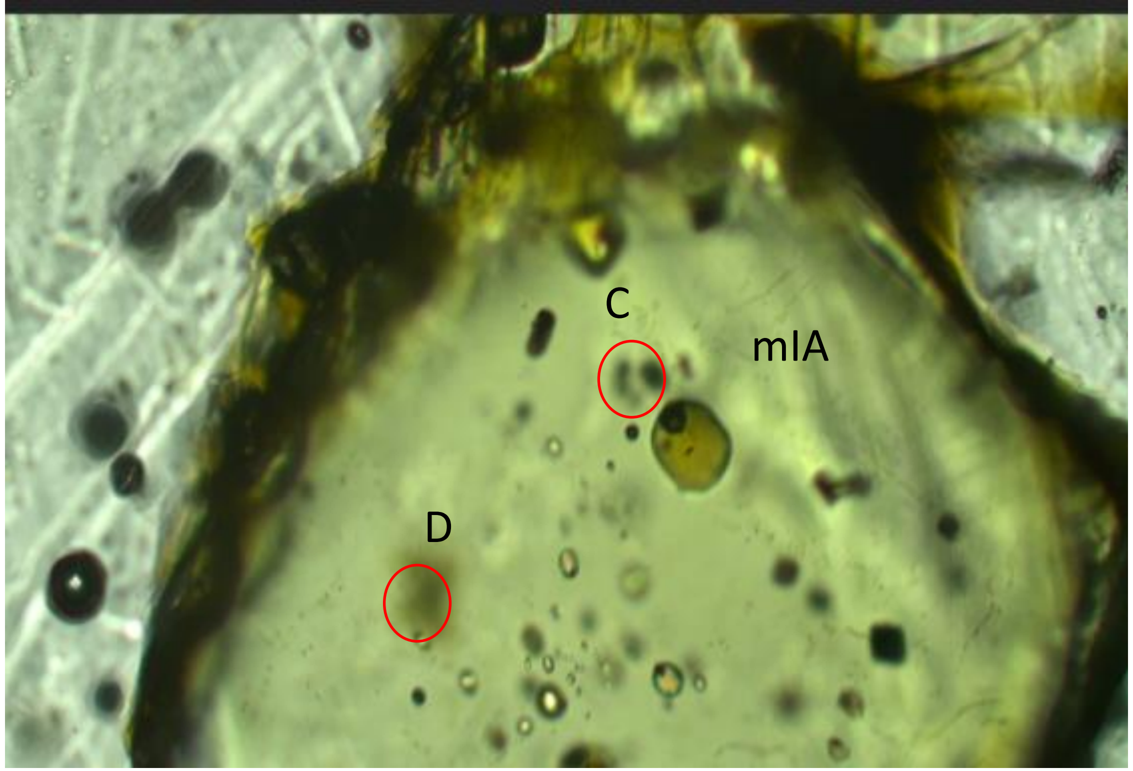

ML22_16_FIC

Mag	Width (µm)	Height (µm)
50	223.221	139.513

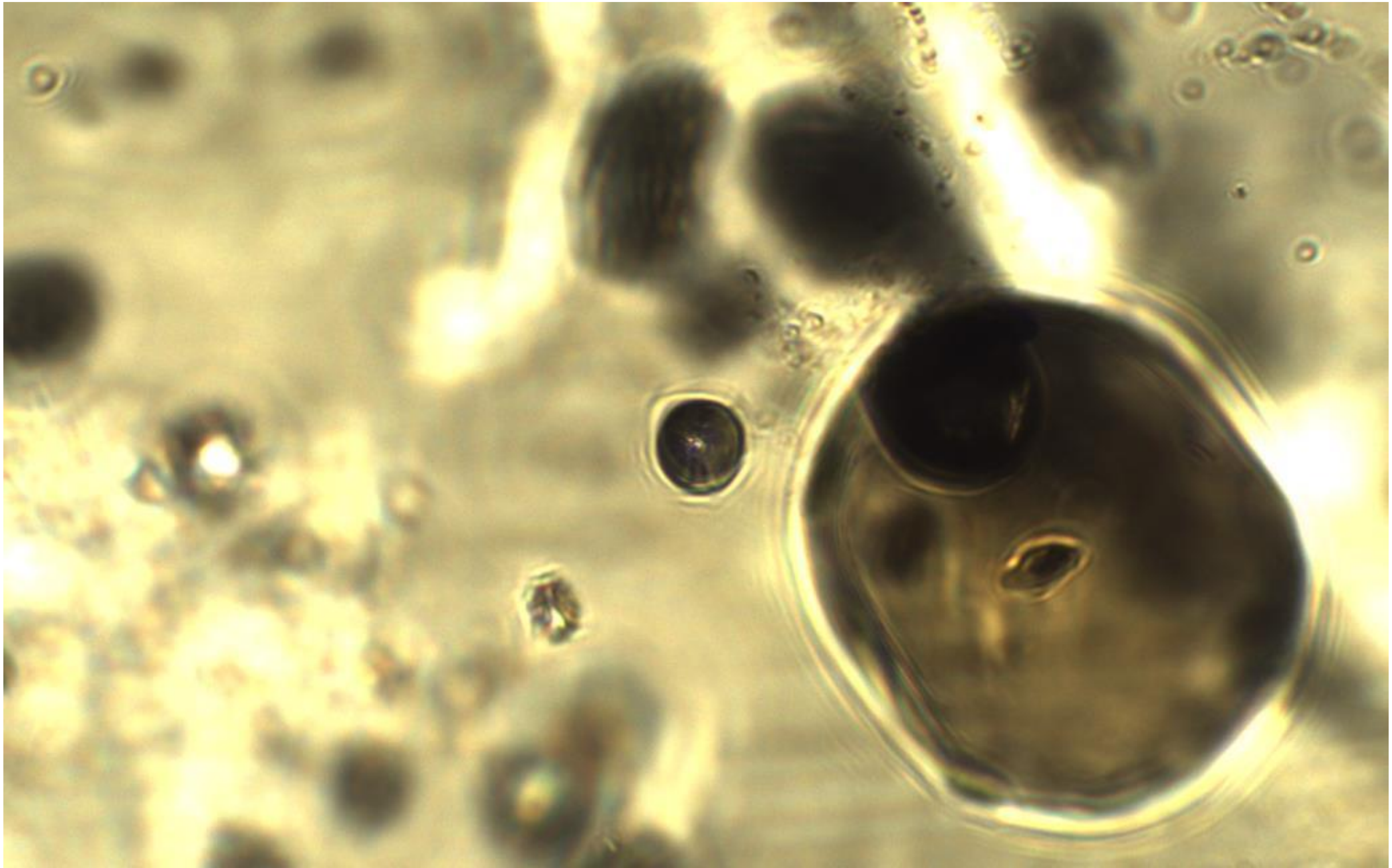

The following slides show the location of EPMA points used to characterize a Fo content for each fluid inclusion (to then get temperature)

ML22_1_FIA

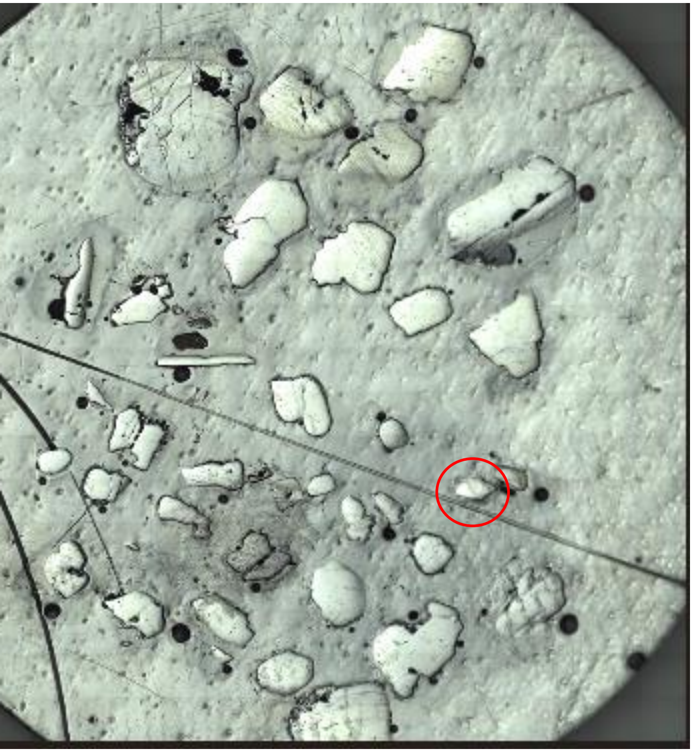

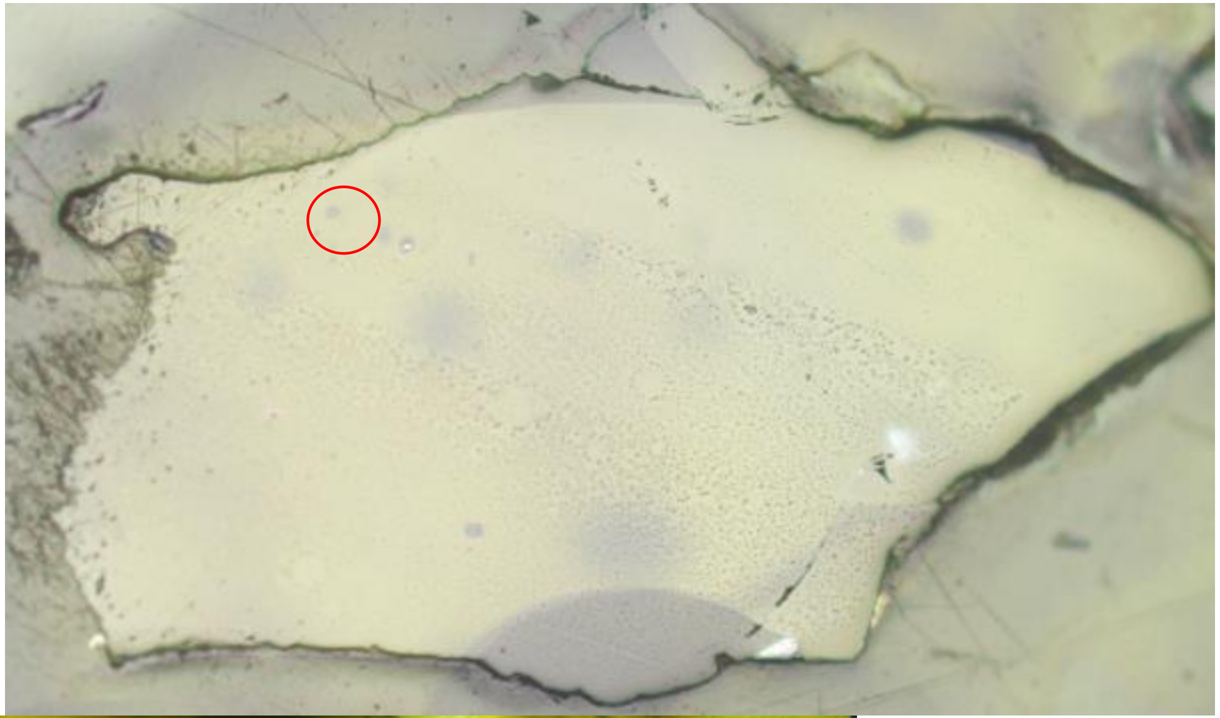

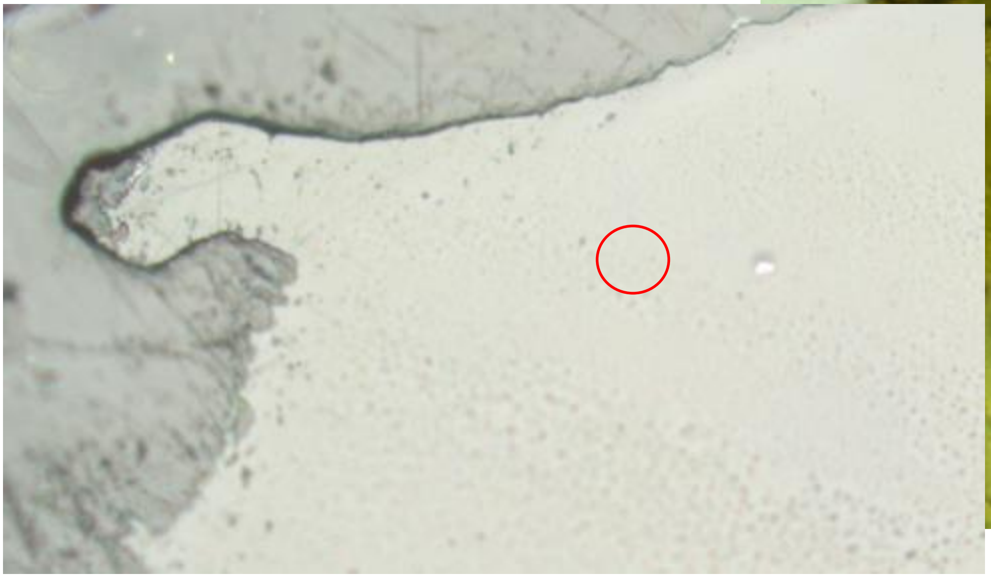

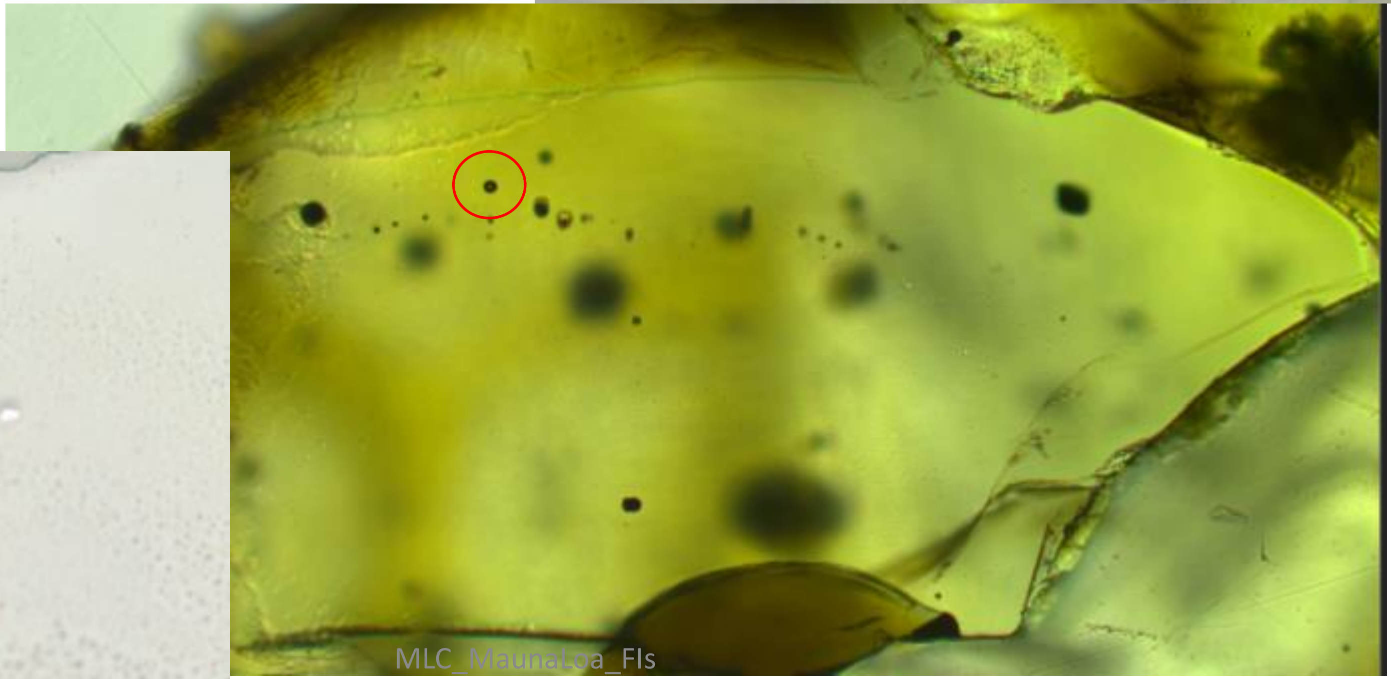

ML22_16_FIA

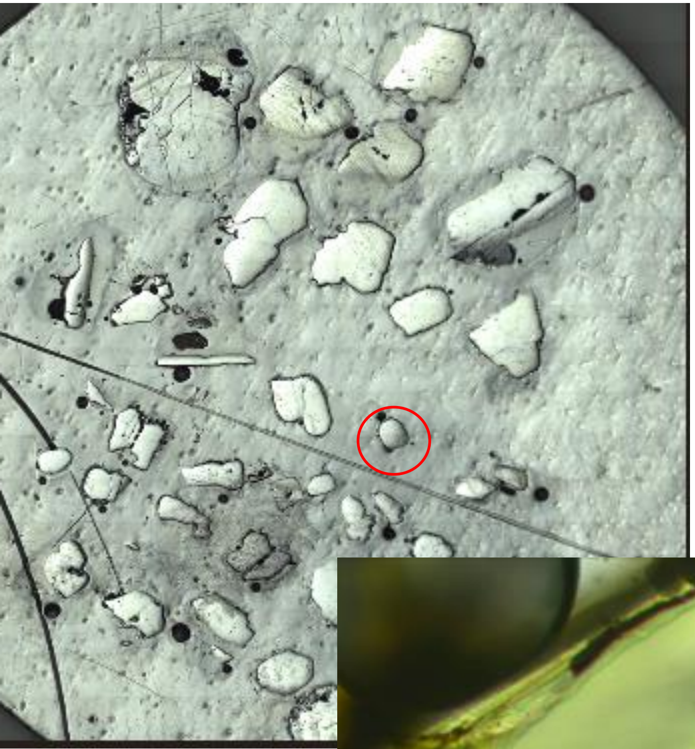

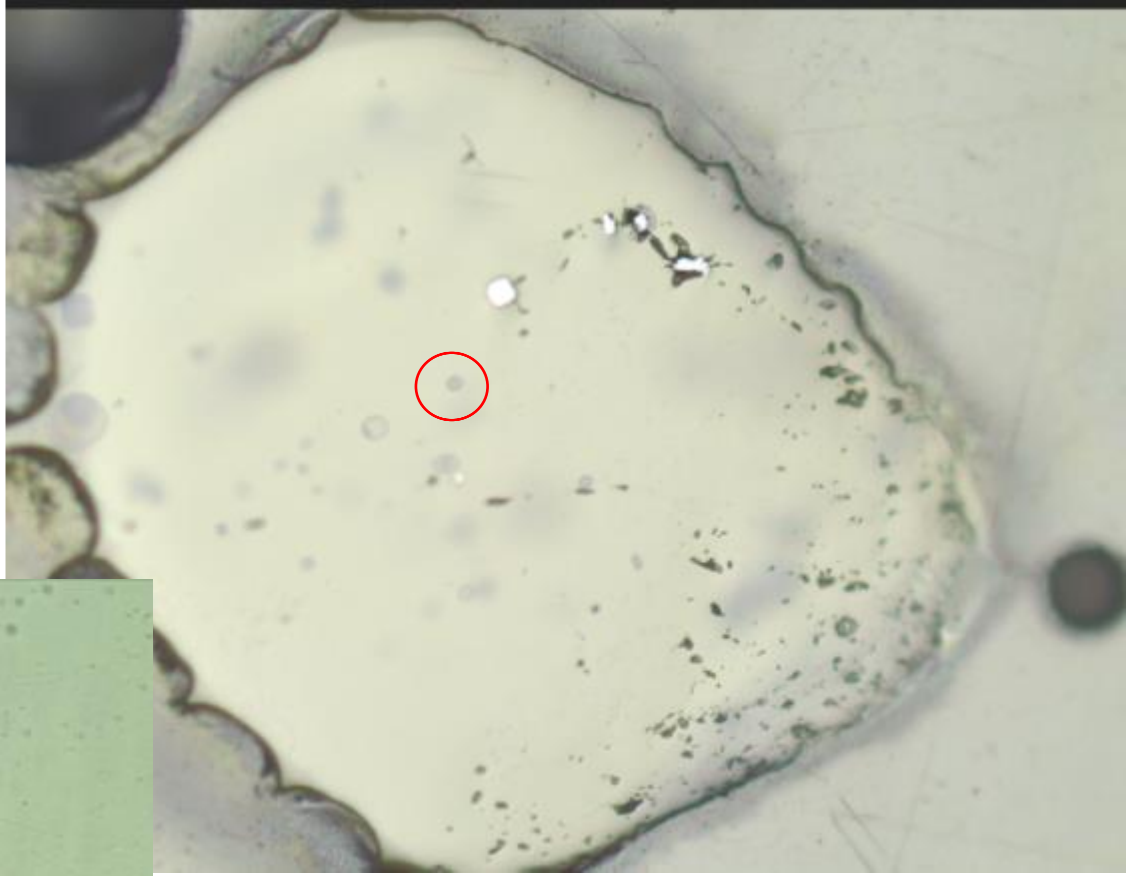

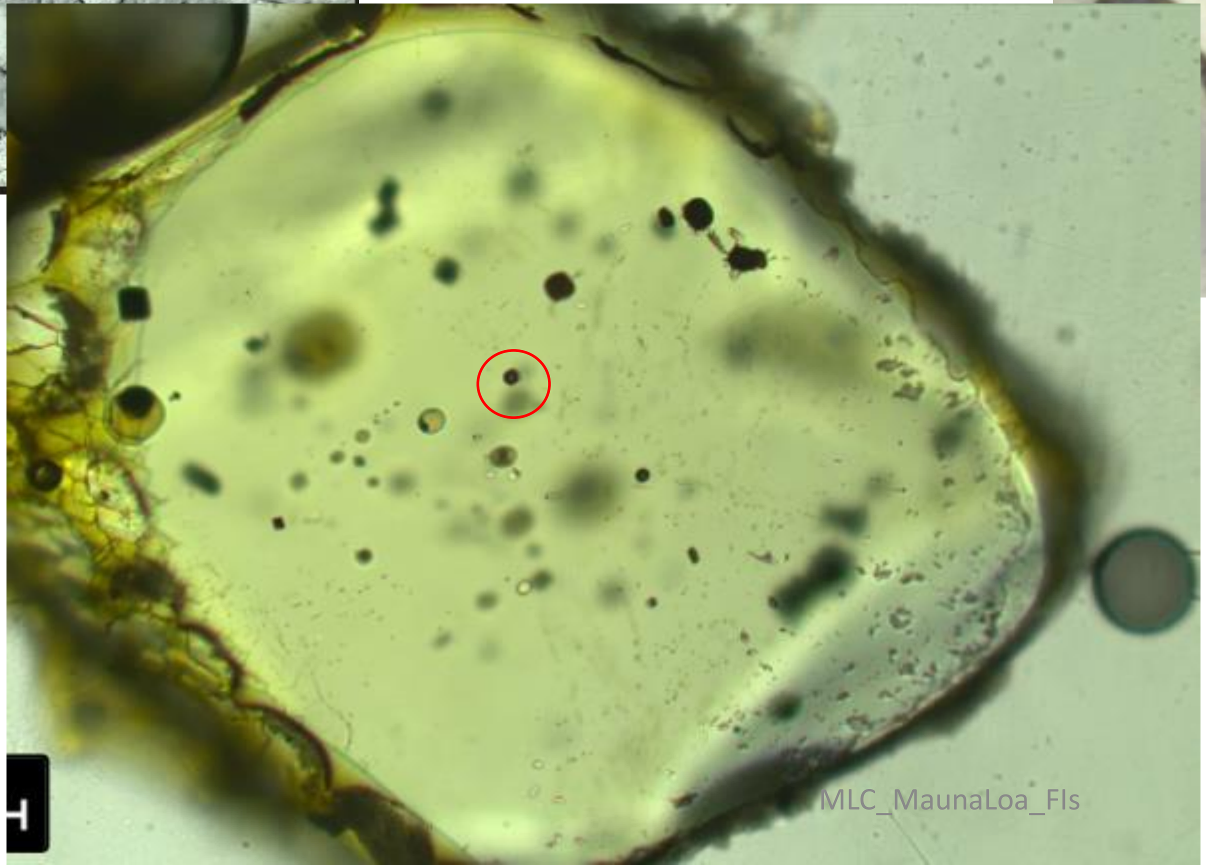

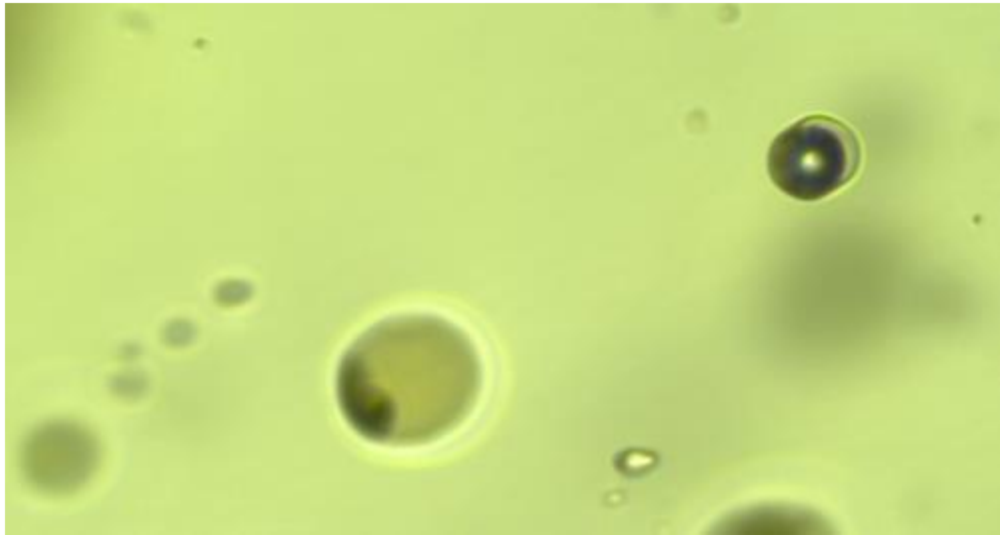

ML22_16_FIB

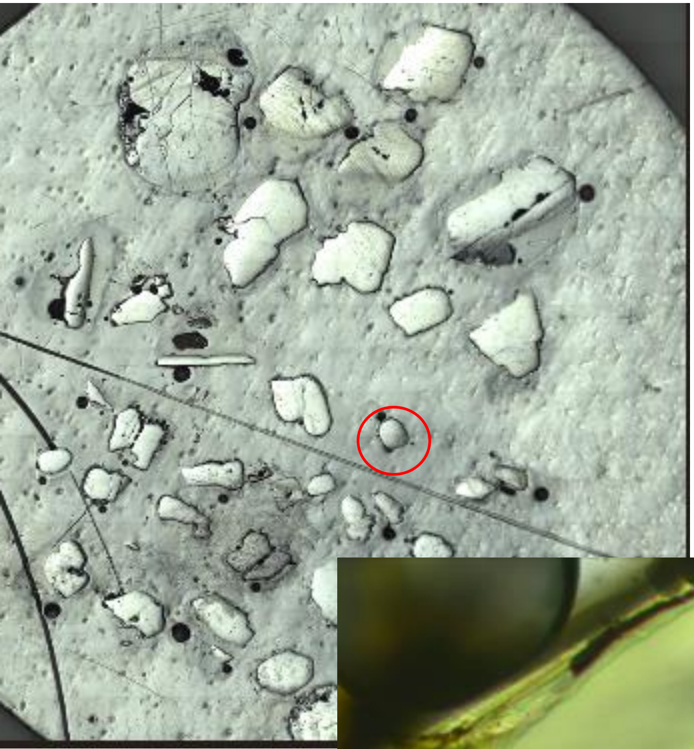

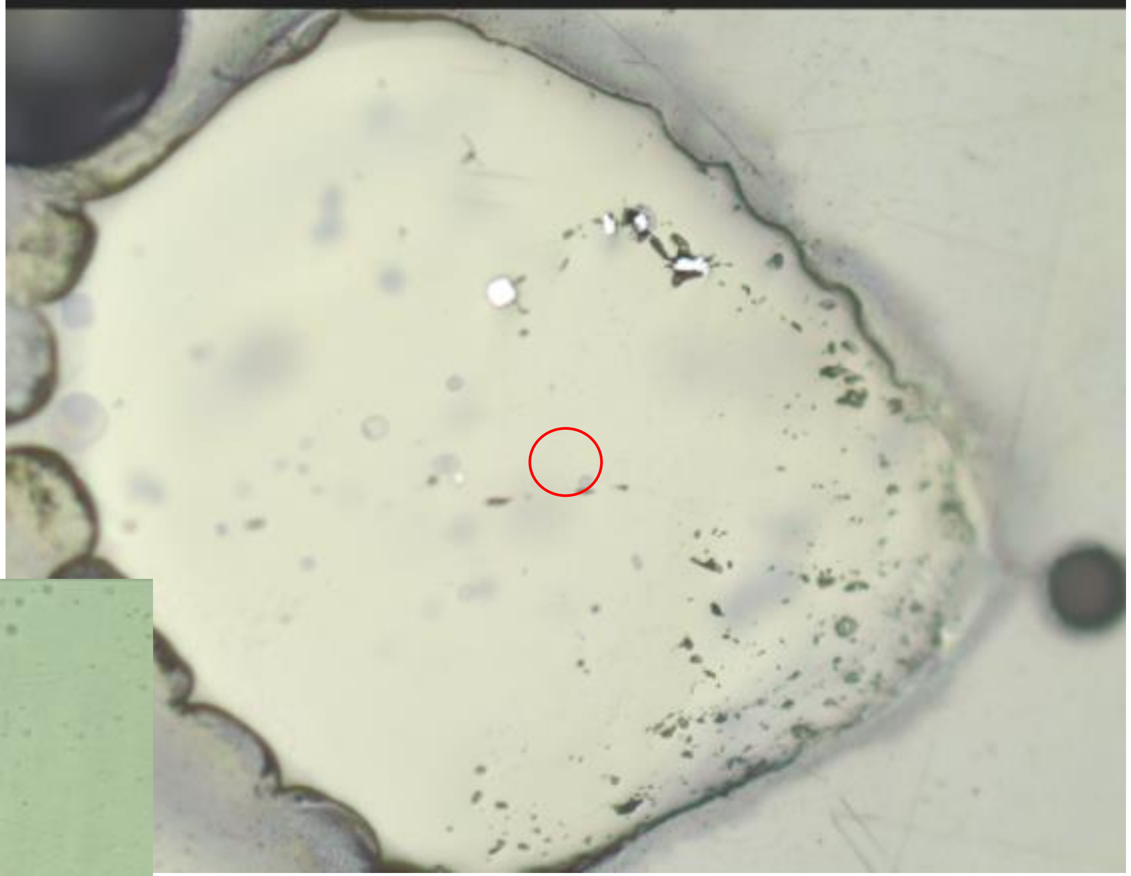

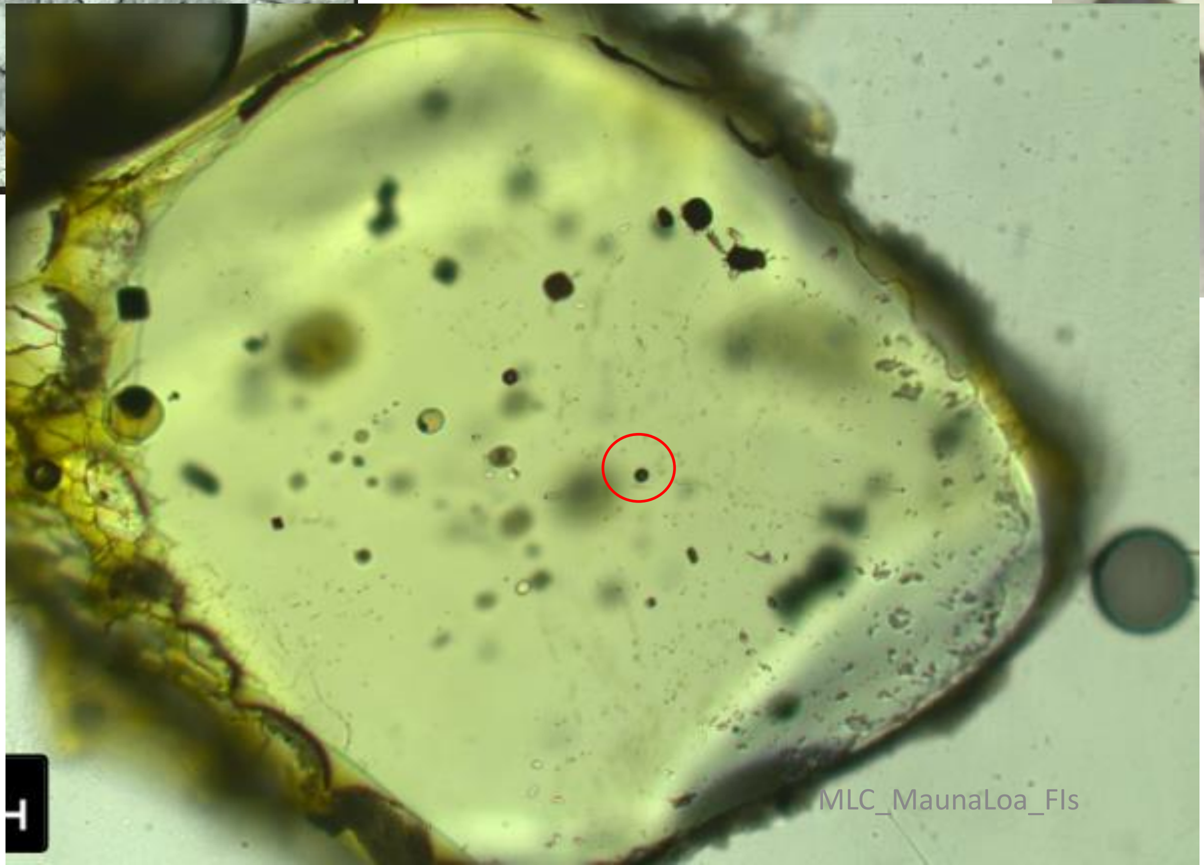

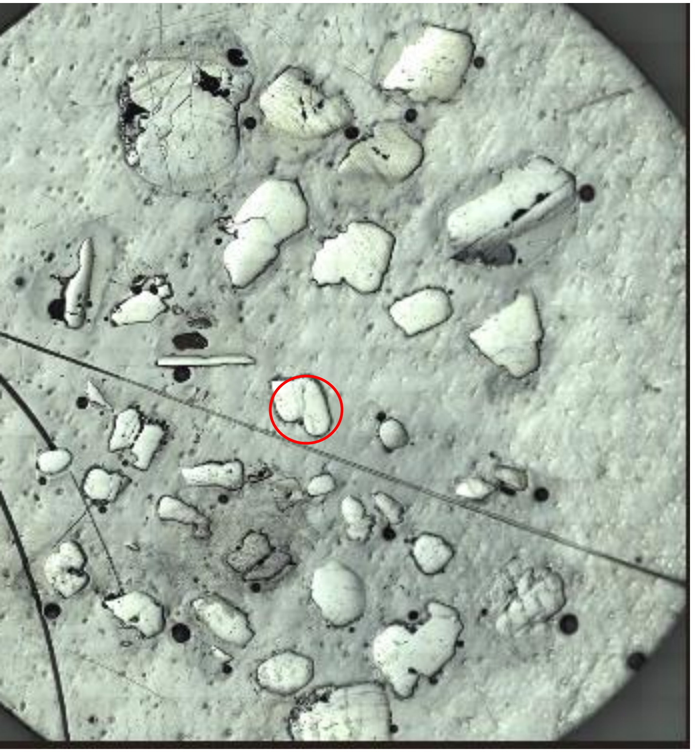

ML22_13
Mounted upsideown. Take a few Fo to check for homogeneity

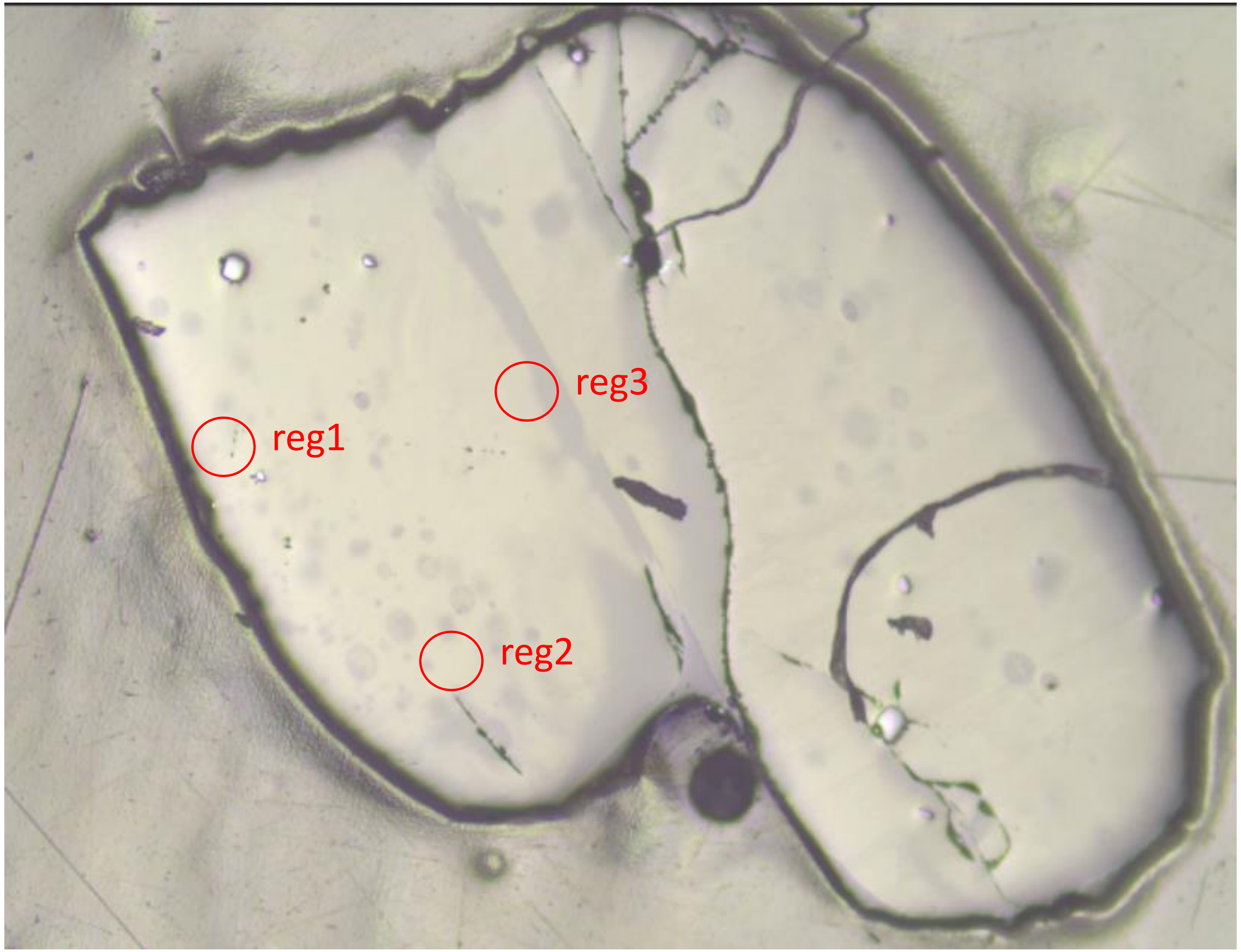

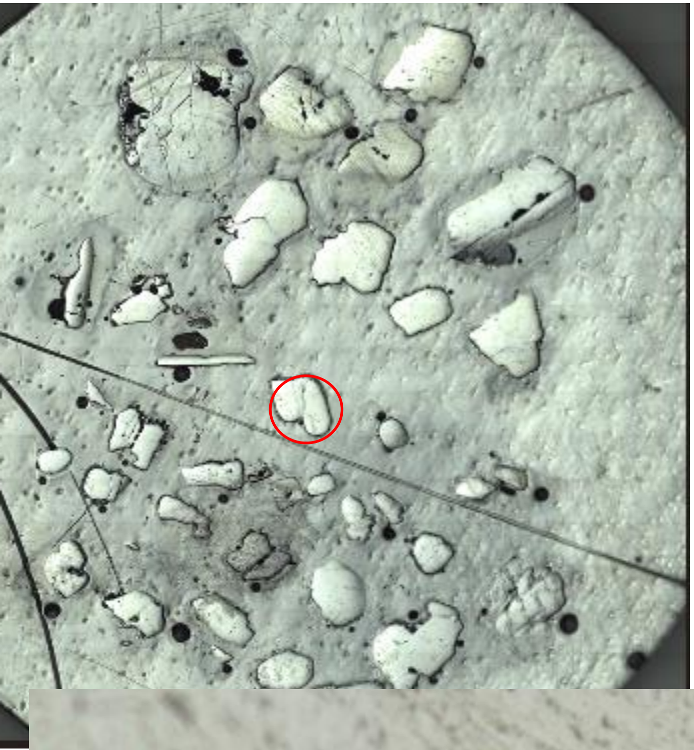

ML22_7_reg1
For PW
A and B

Is also another nice one underneath.

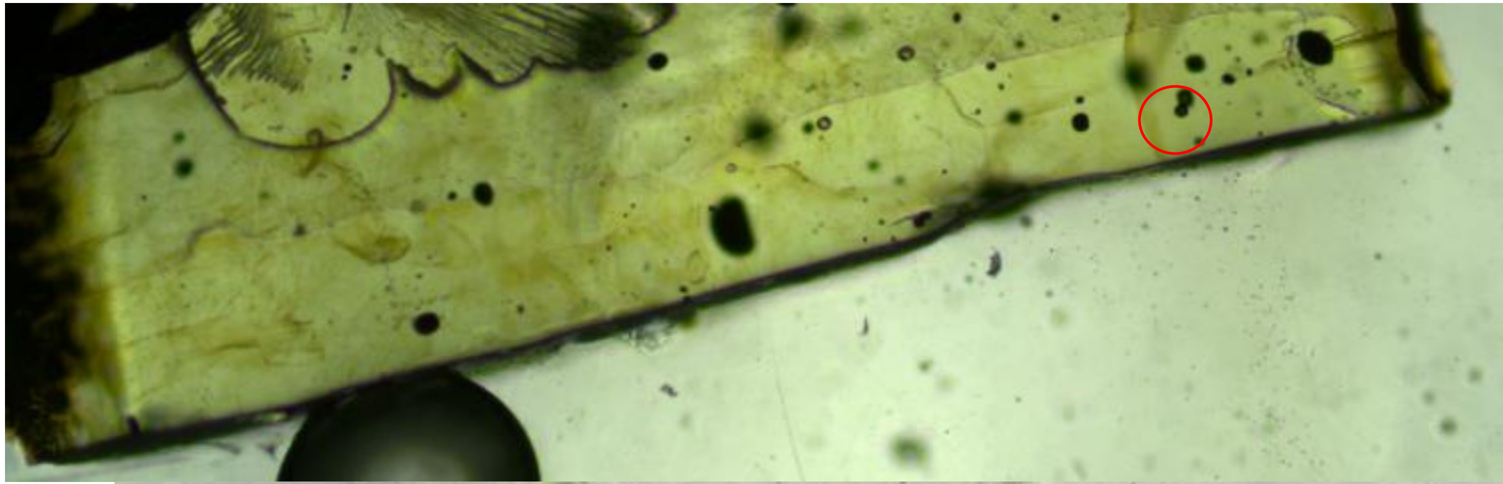

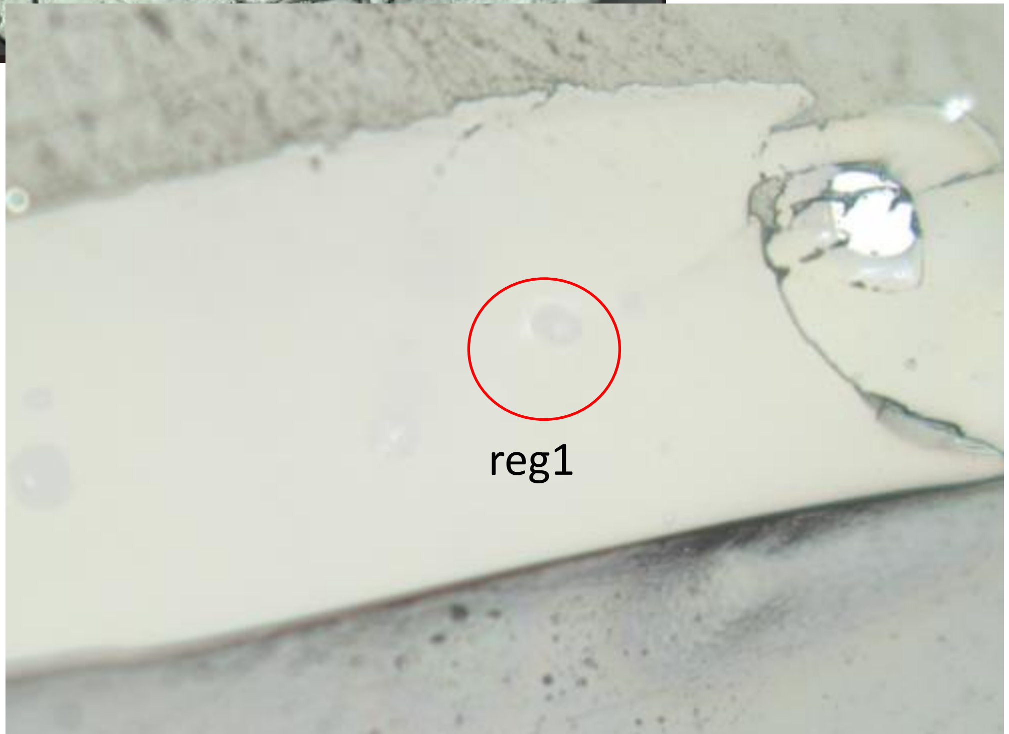

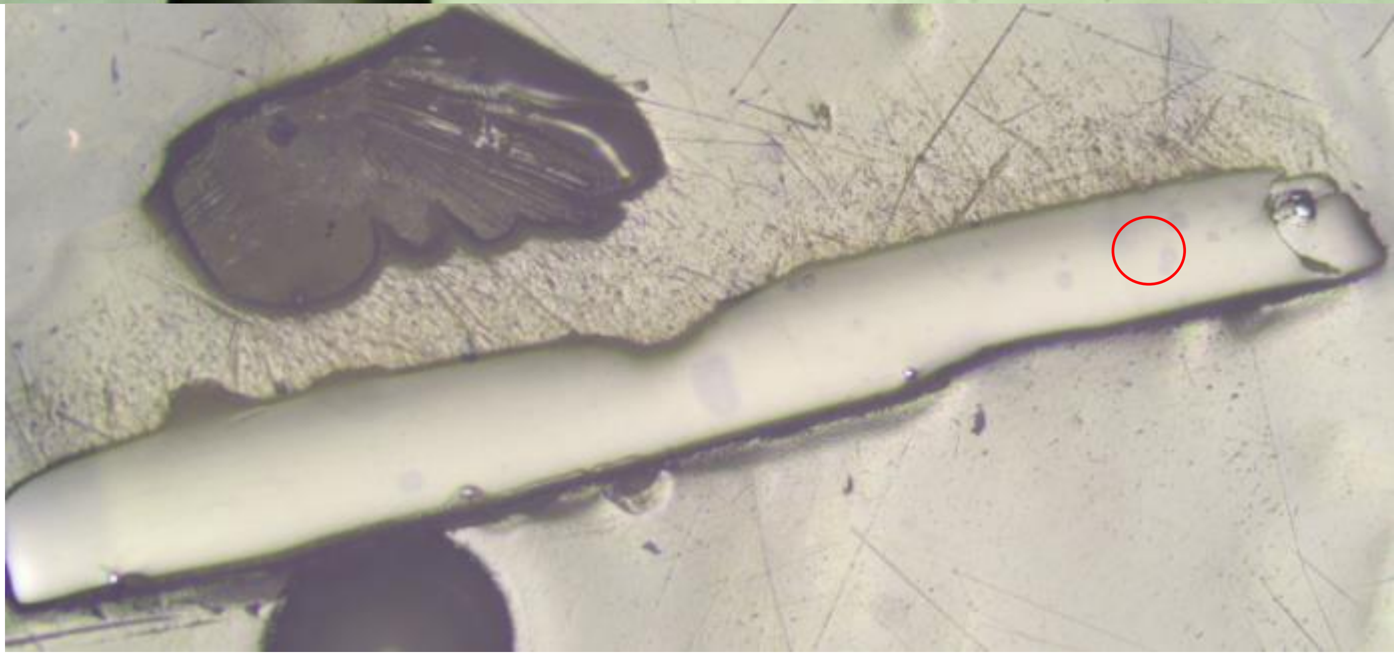

ML22_15_FIA

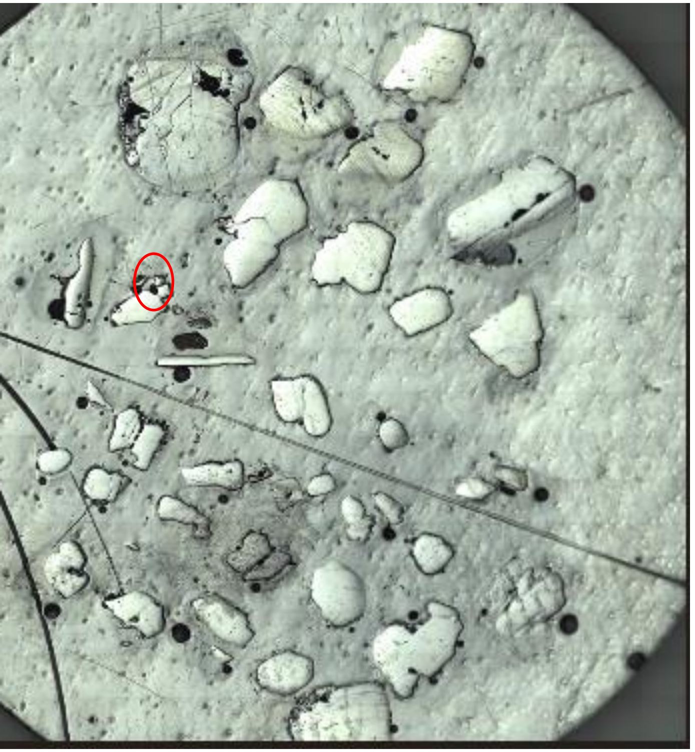

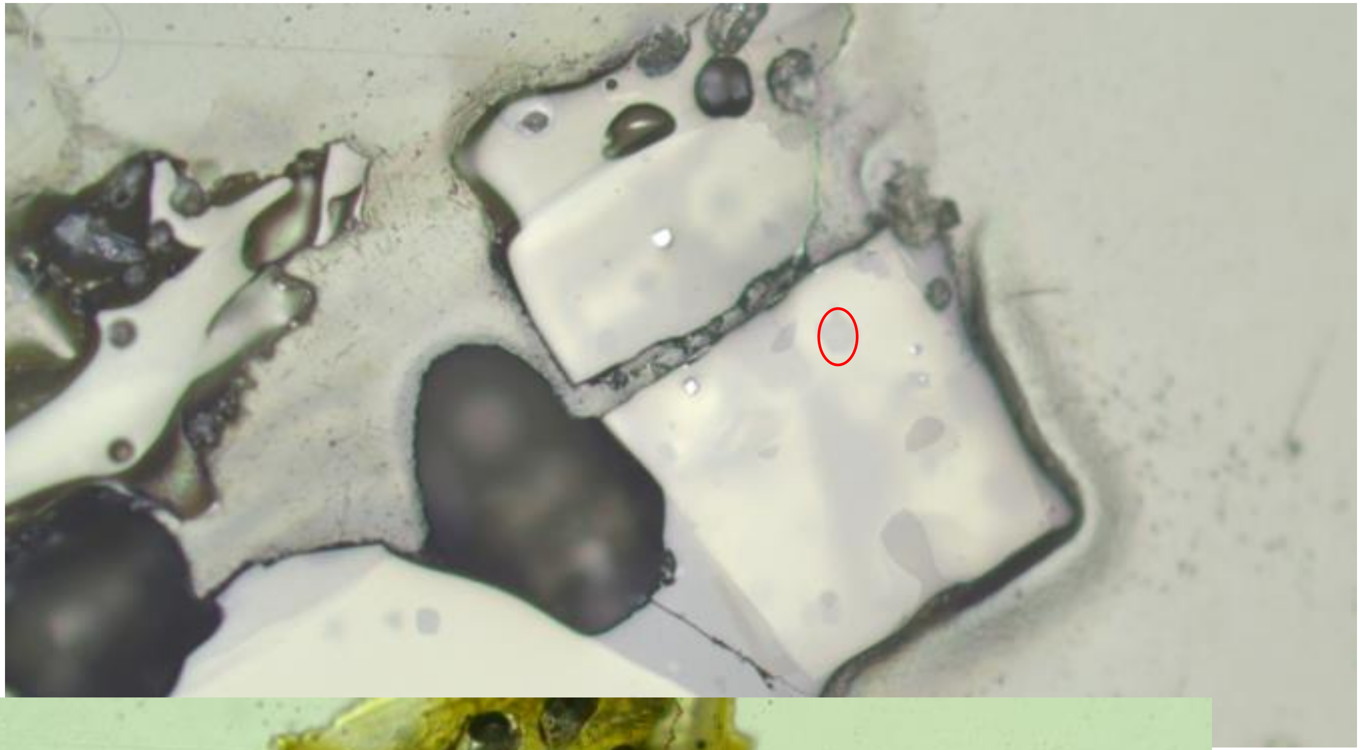

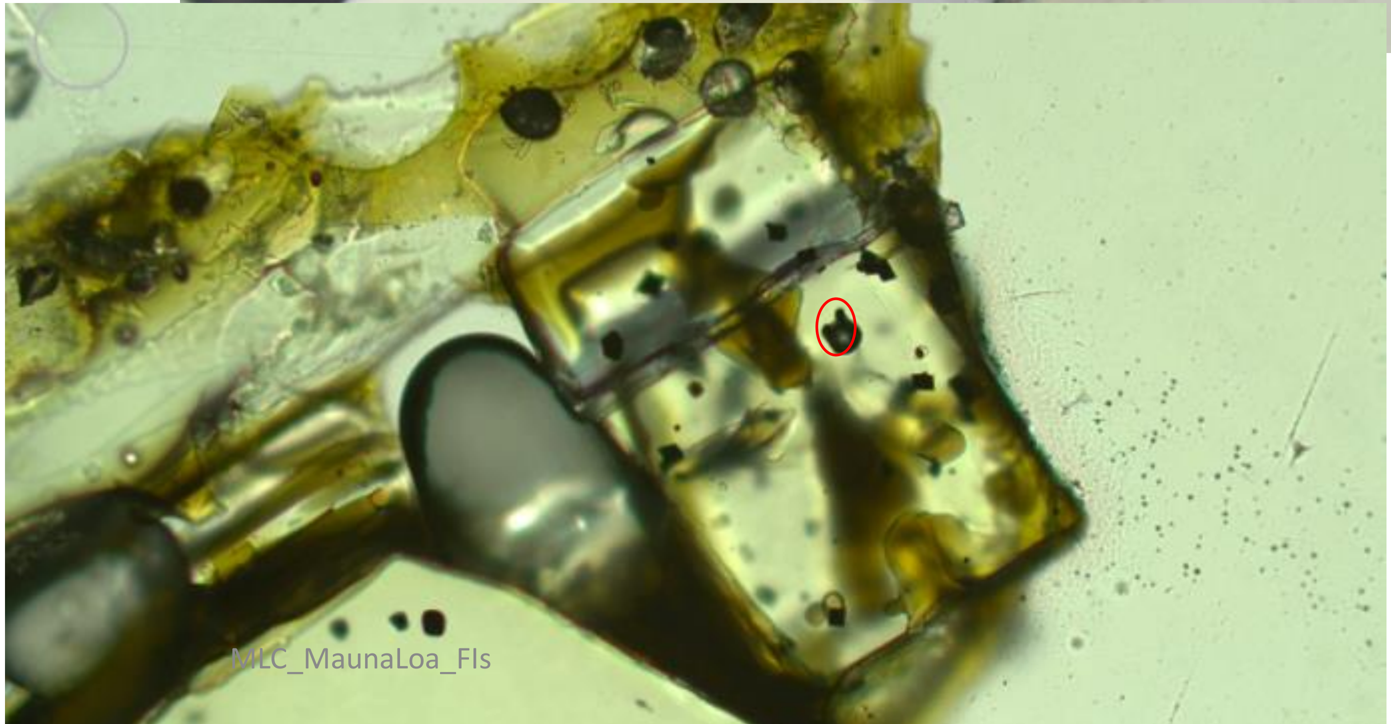

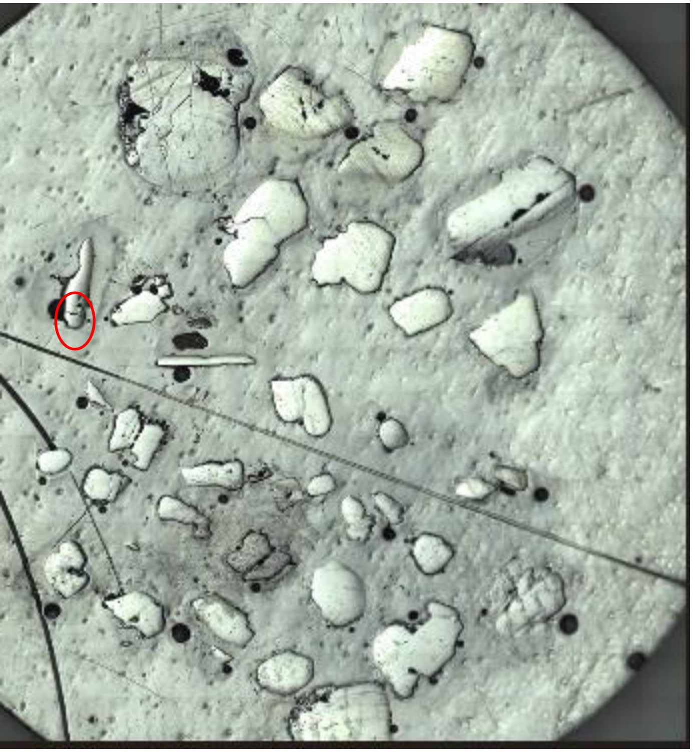

ML22_2_reg1,
reg2
PW:FIA and
FIB not
exposed. For A
average r1-r2,
B use R2

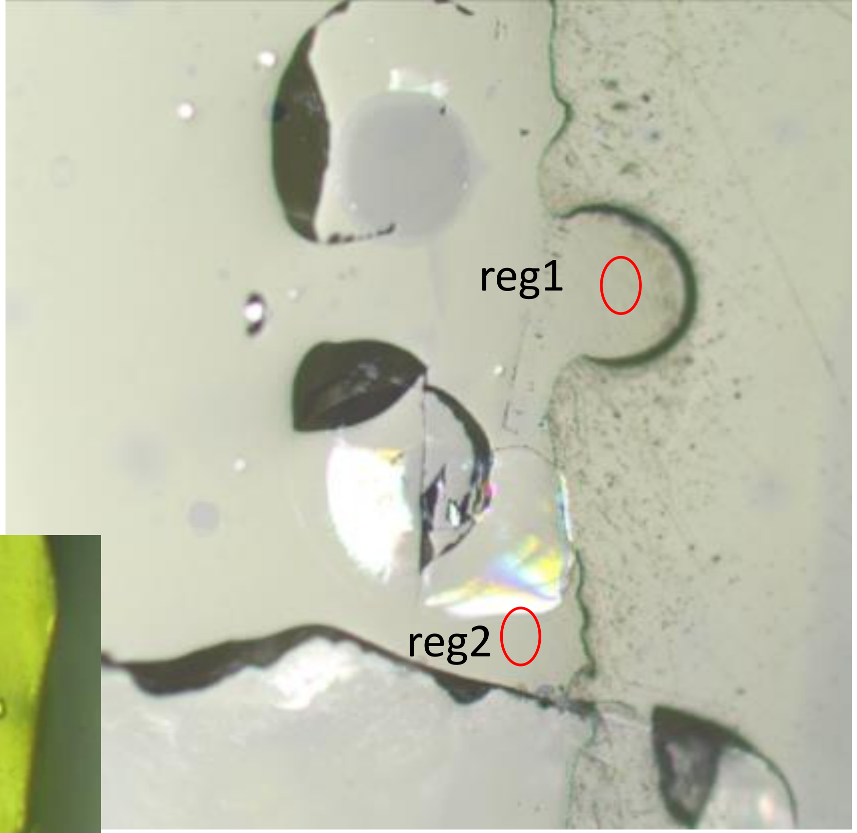

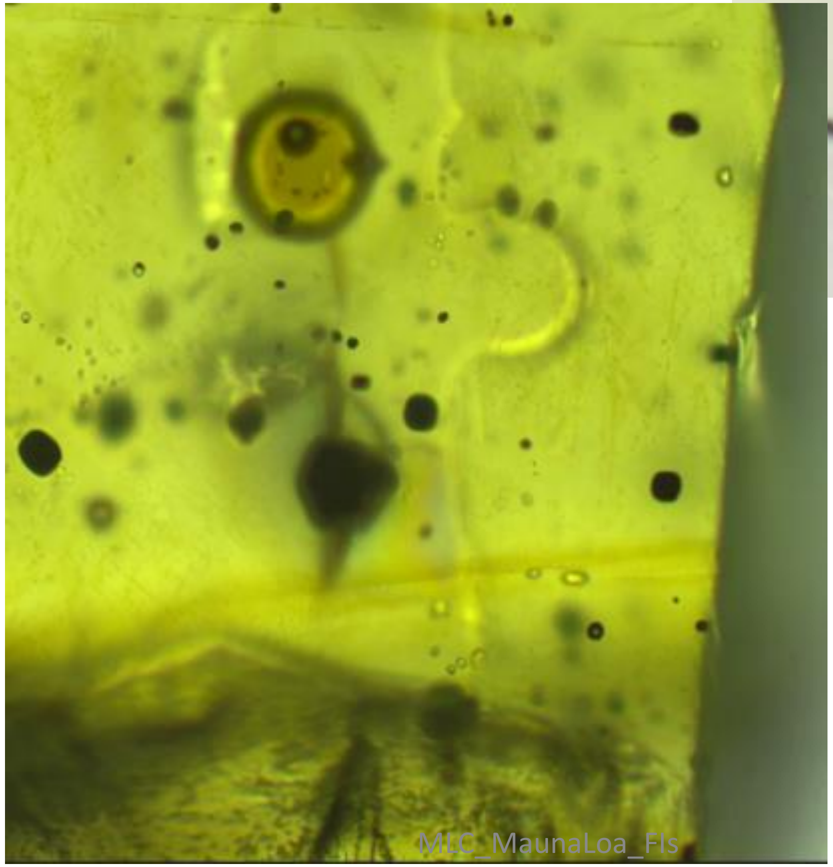

The following slides show images of fluid inclusions which were found within epoxy mounts used for diffusion chronometry.

ML_ori_1 = mount name

ML_ori_1_ol1 = ol 1 in this mount

ML_ori_1_ol1_A = FIA in this olivine in this mount.

ML_ORI_1

ML_ORI_1_ol1

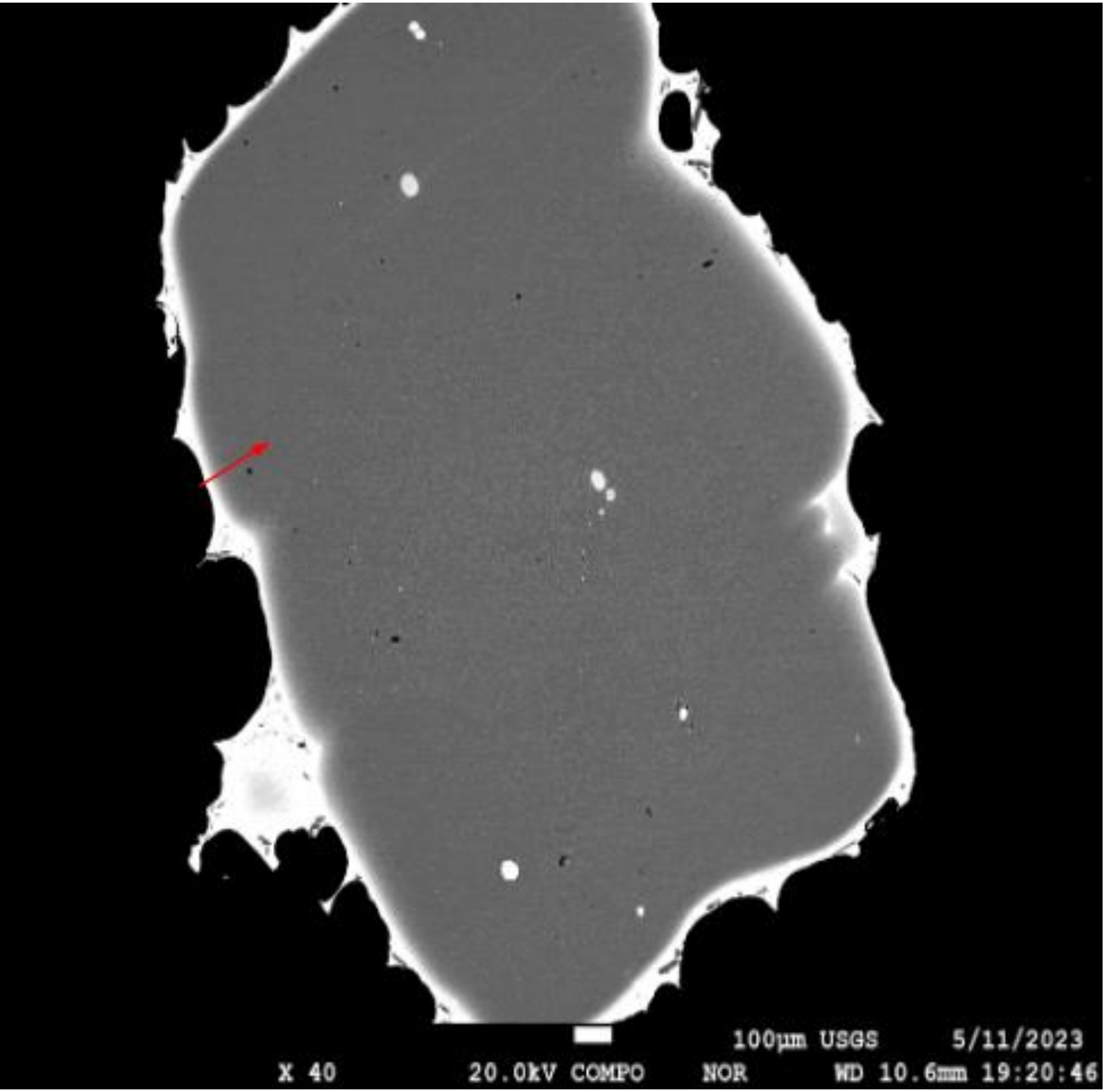

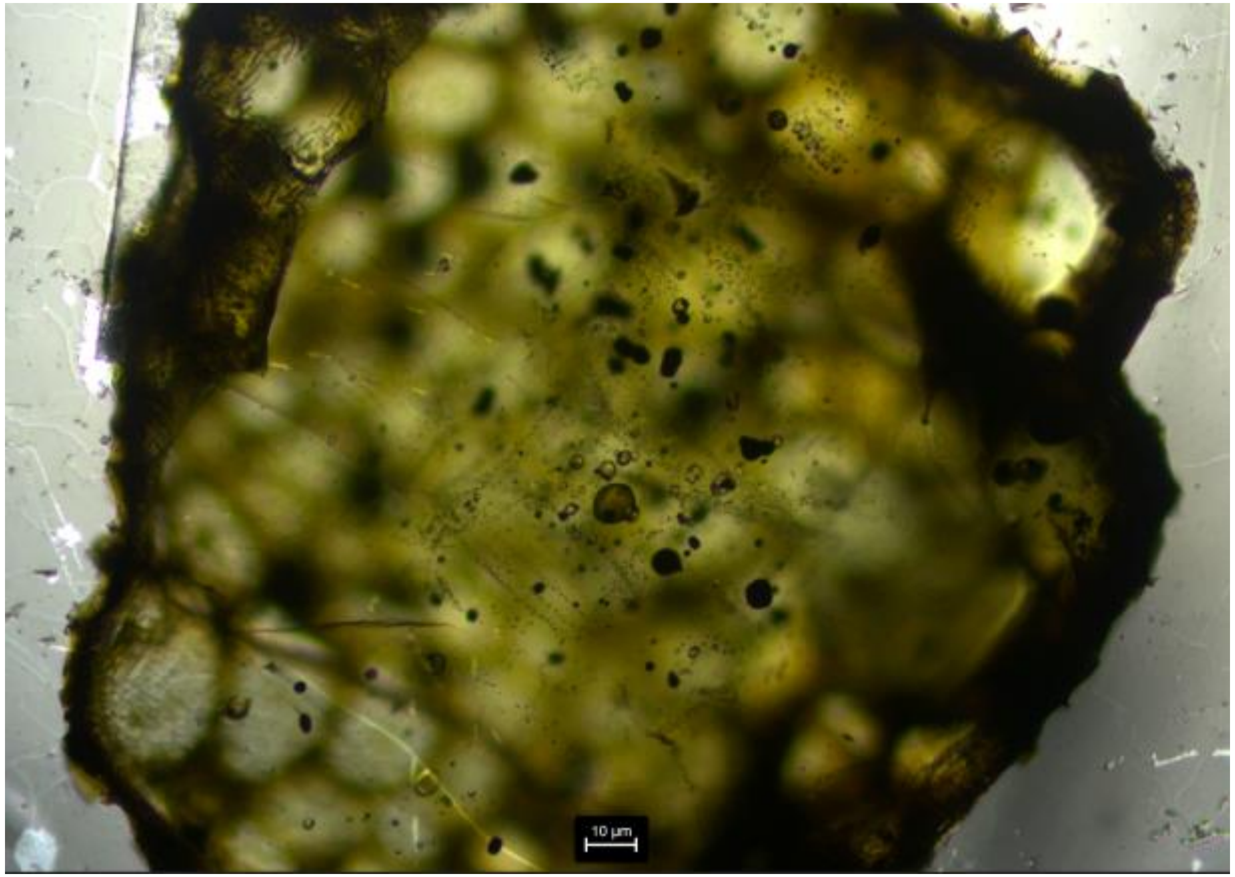

ML_ORI_1_ol1_A

Density: 0.176326962

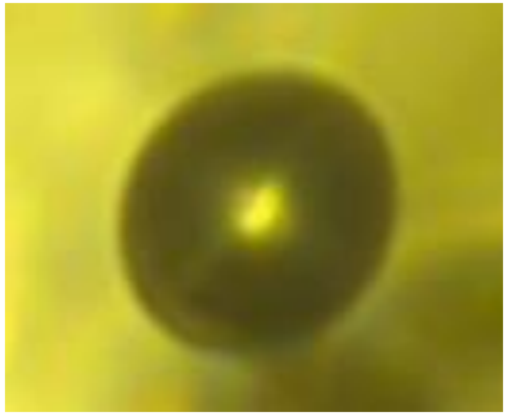

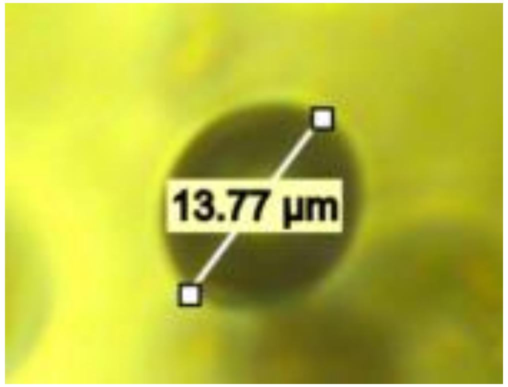

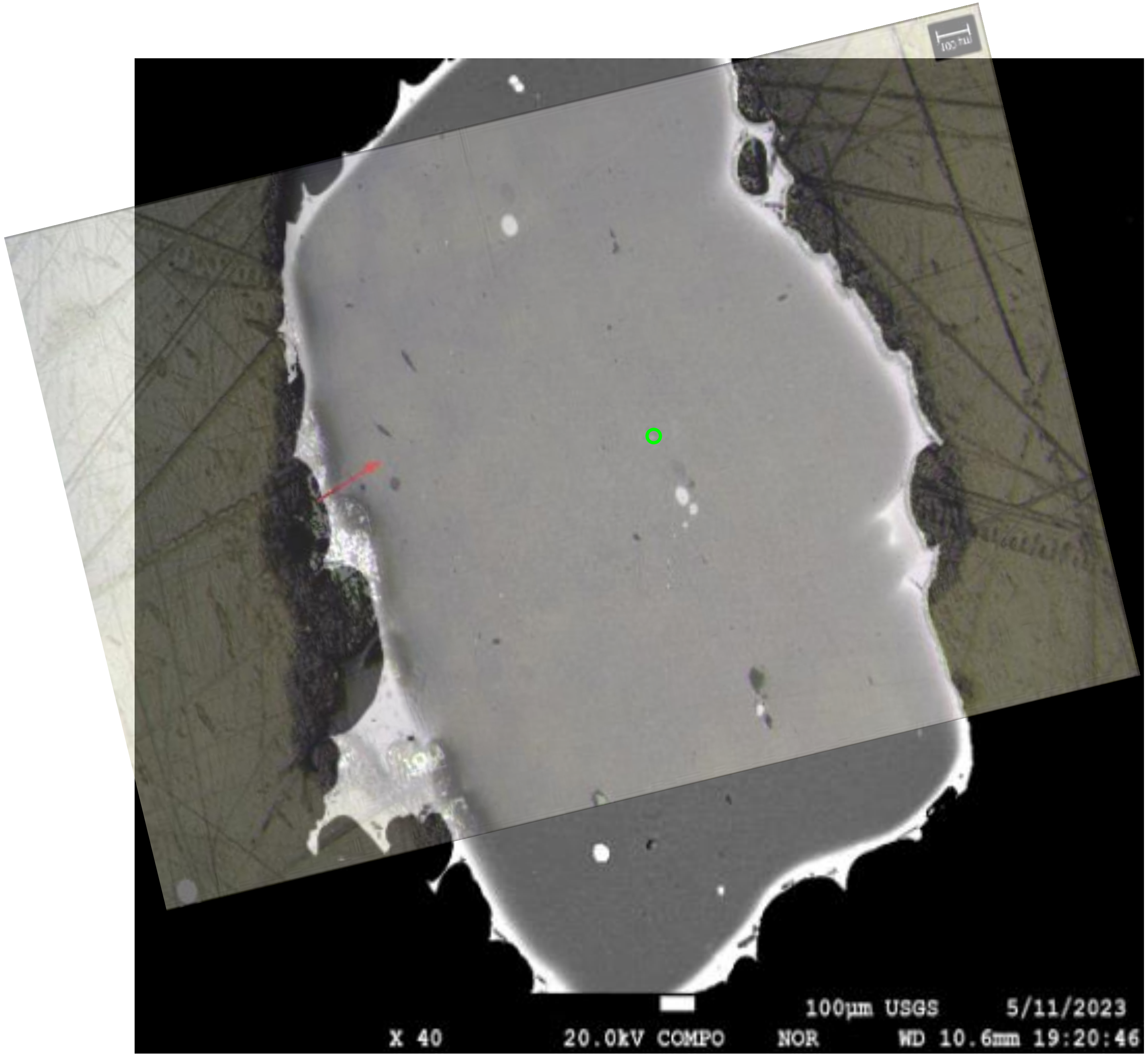

ML_ORI_1_ol1_C

Density: 0.222040931

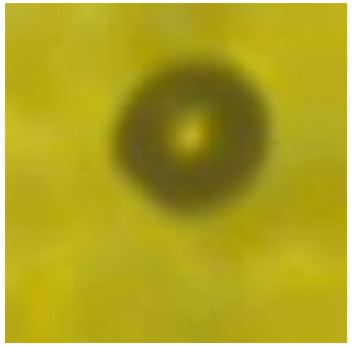

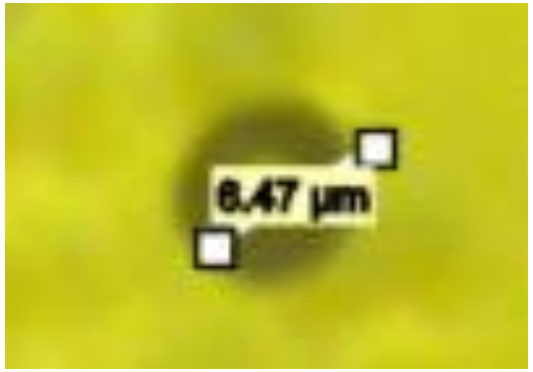

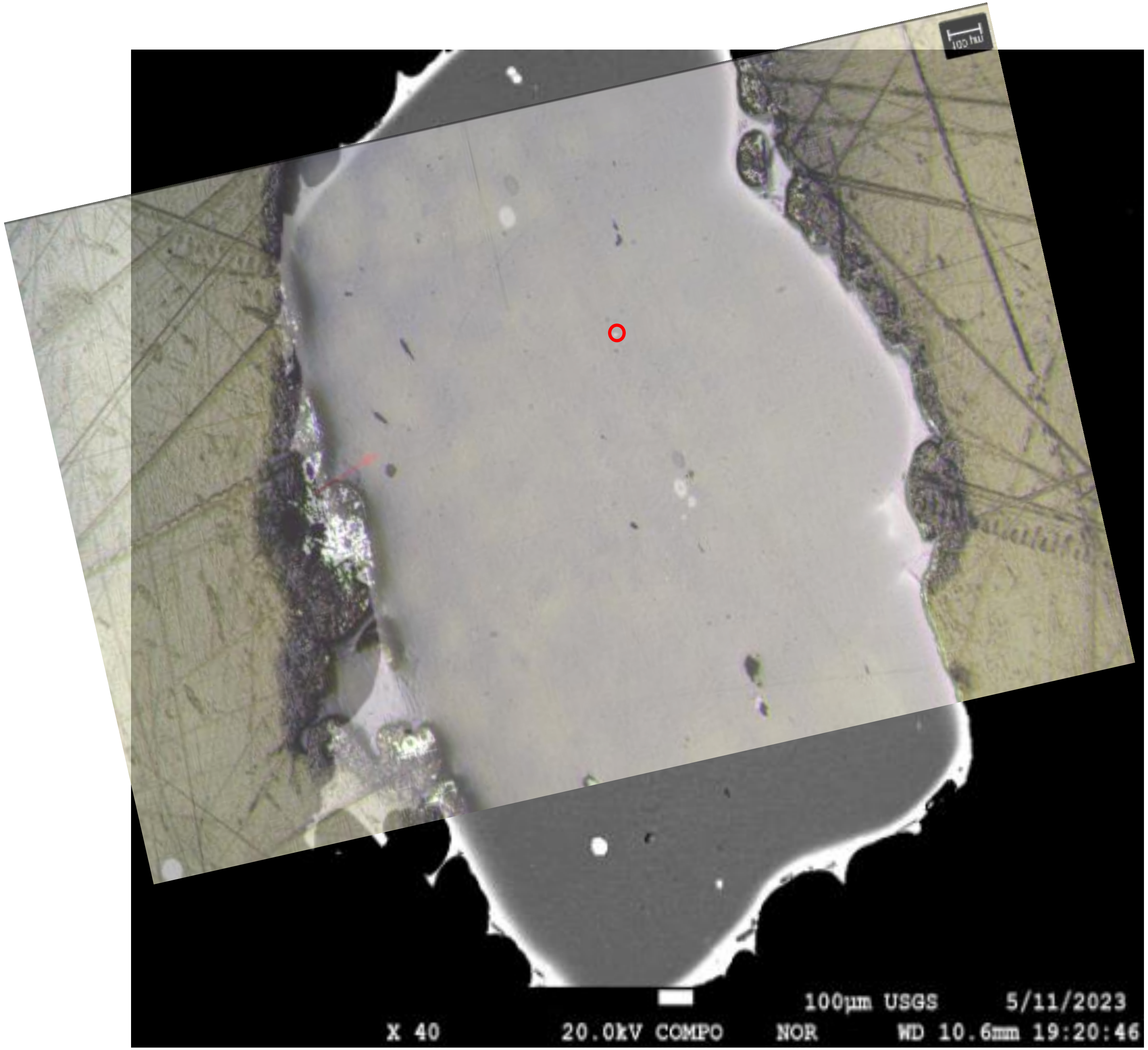

ML_ORI_1_ol1_D

Density: 0.204954806

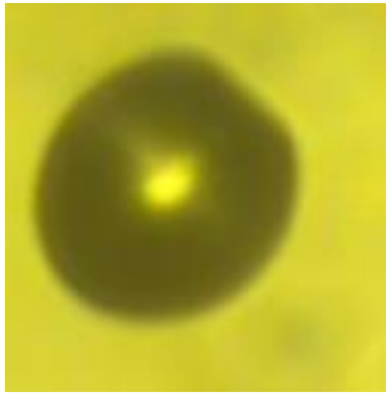

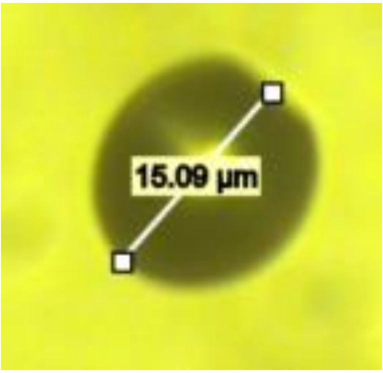

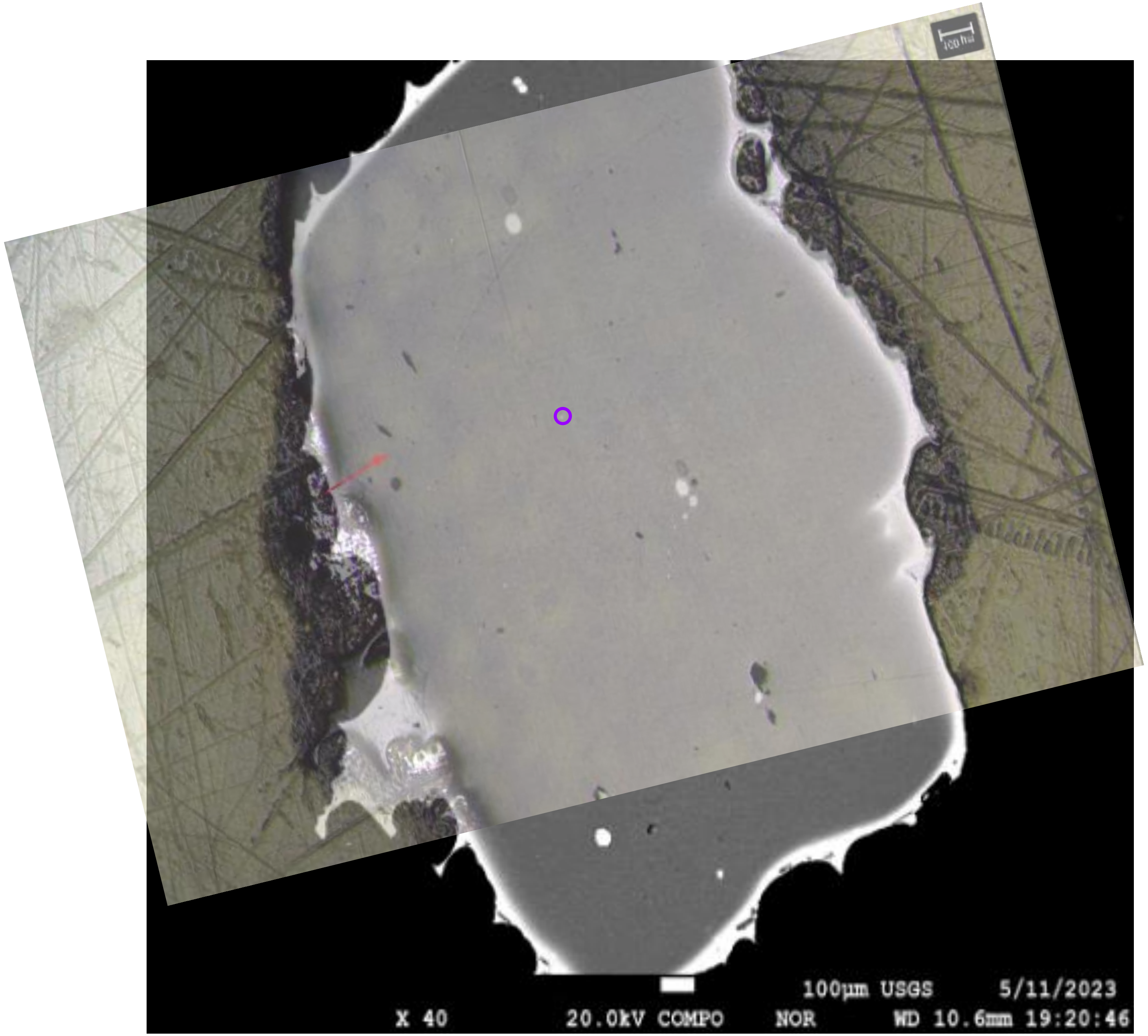

ML_ORI_1_ol1_E

Density: 0.209726671

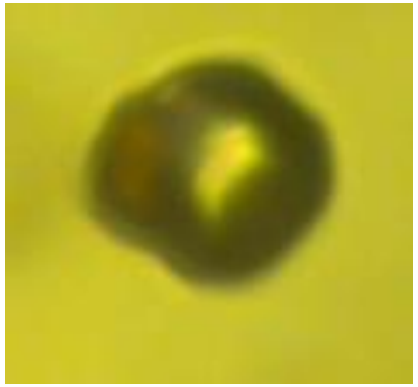

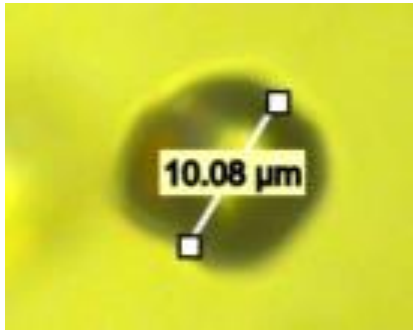

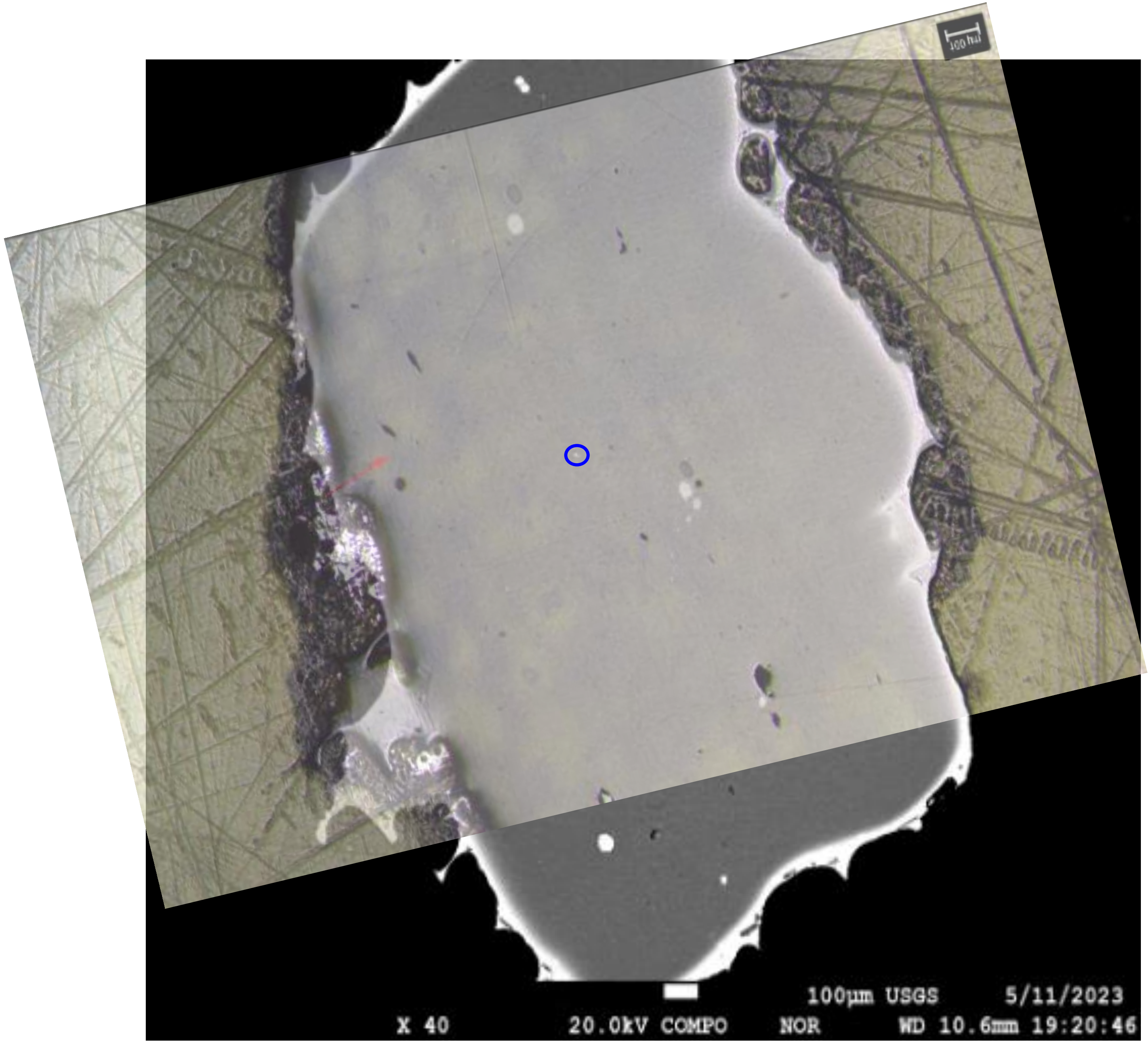

ML_ORI_1_ol1_F

Density: 0.19406259

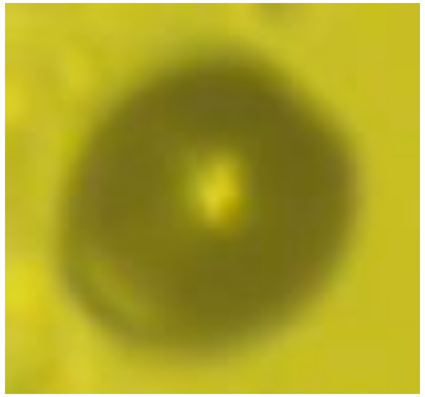

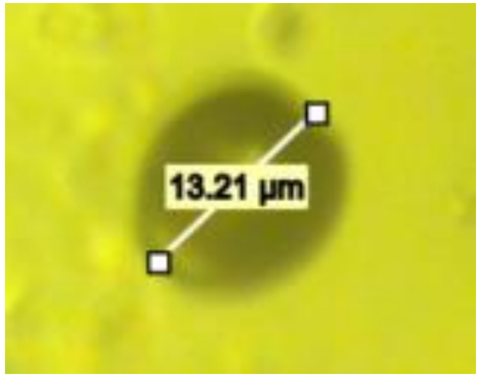

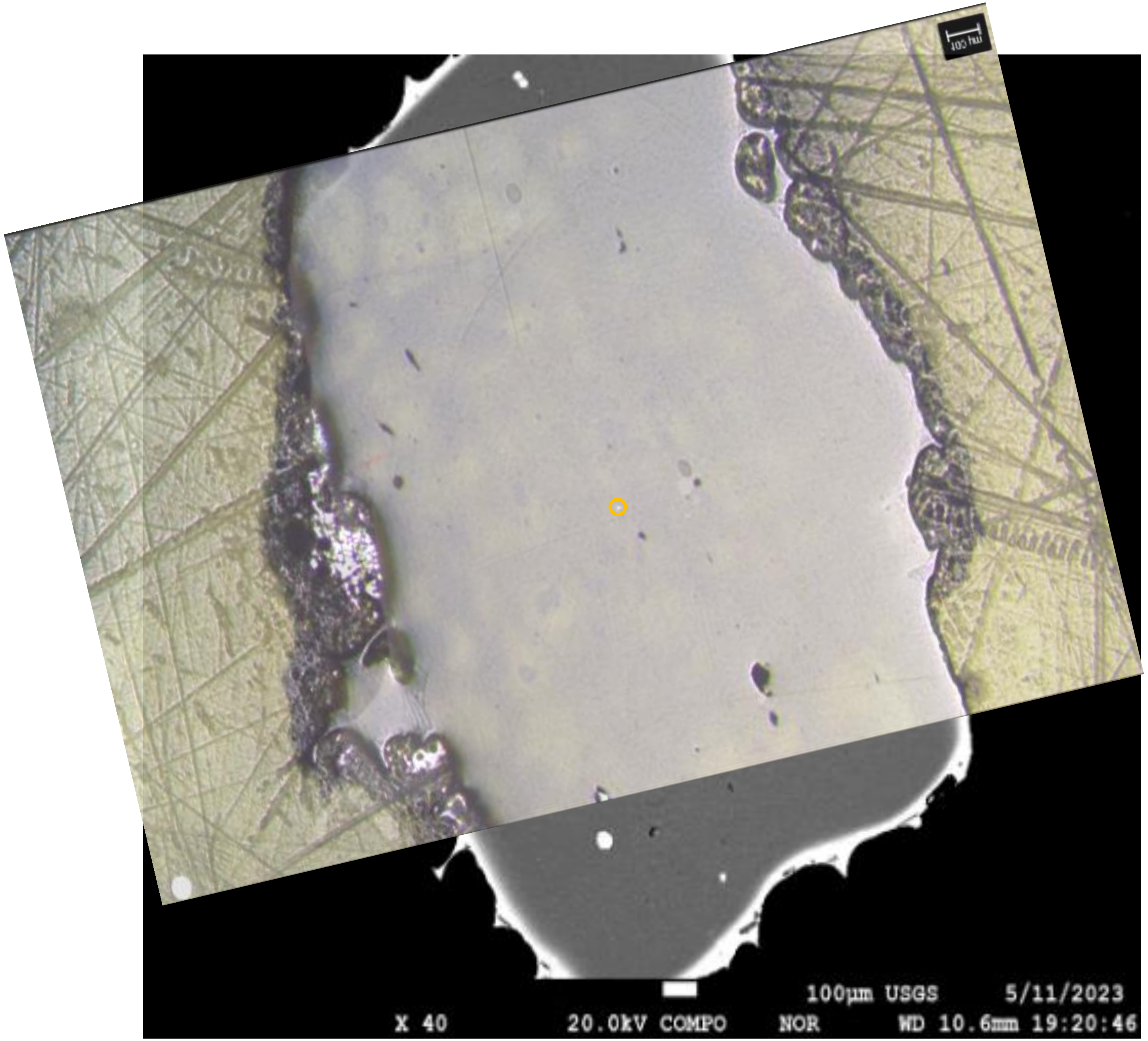

ML_ORI_1_ol1_G

Density: 0.219817149

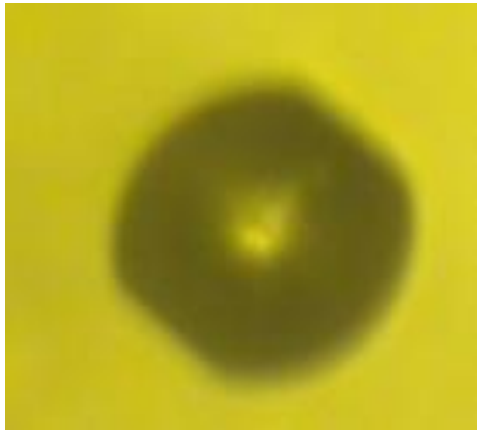

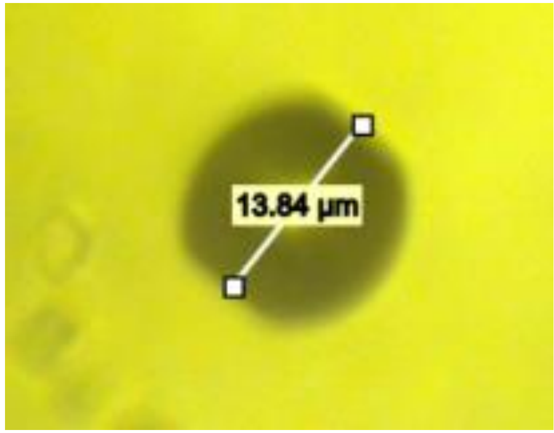

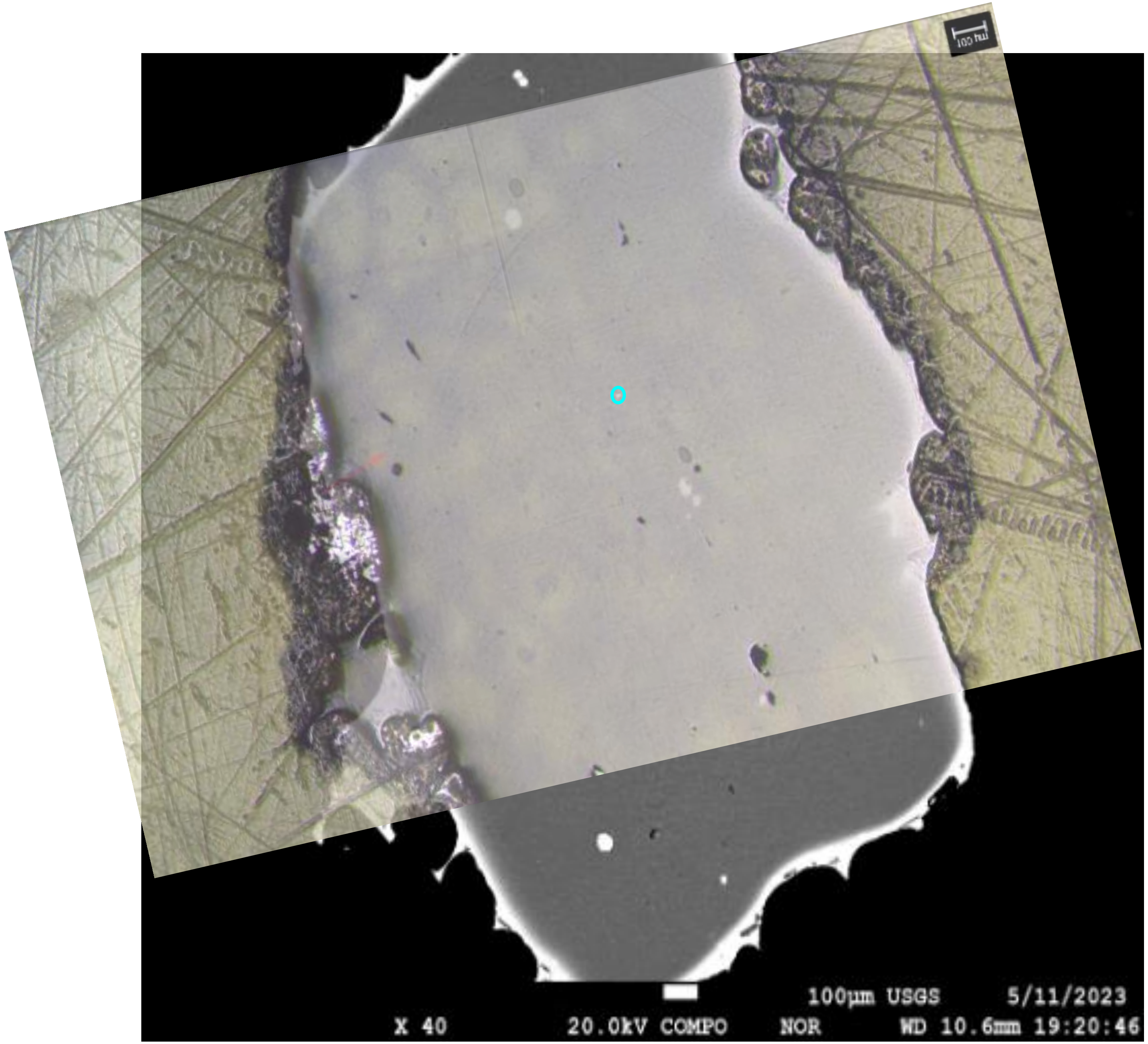

ML_ORI_1_ol1_H

Density: 0.196792487

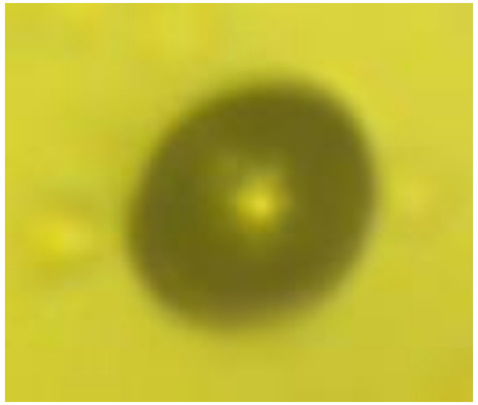

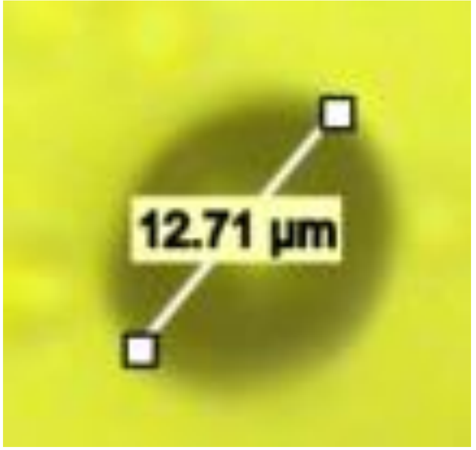

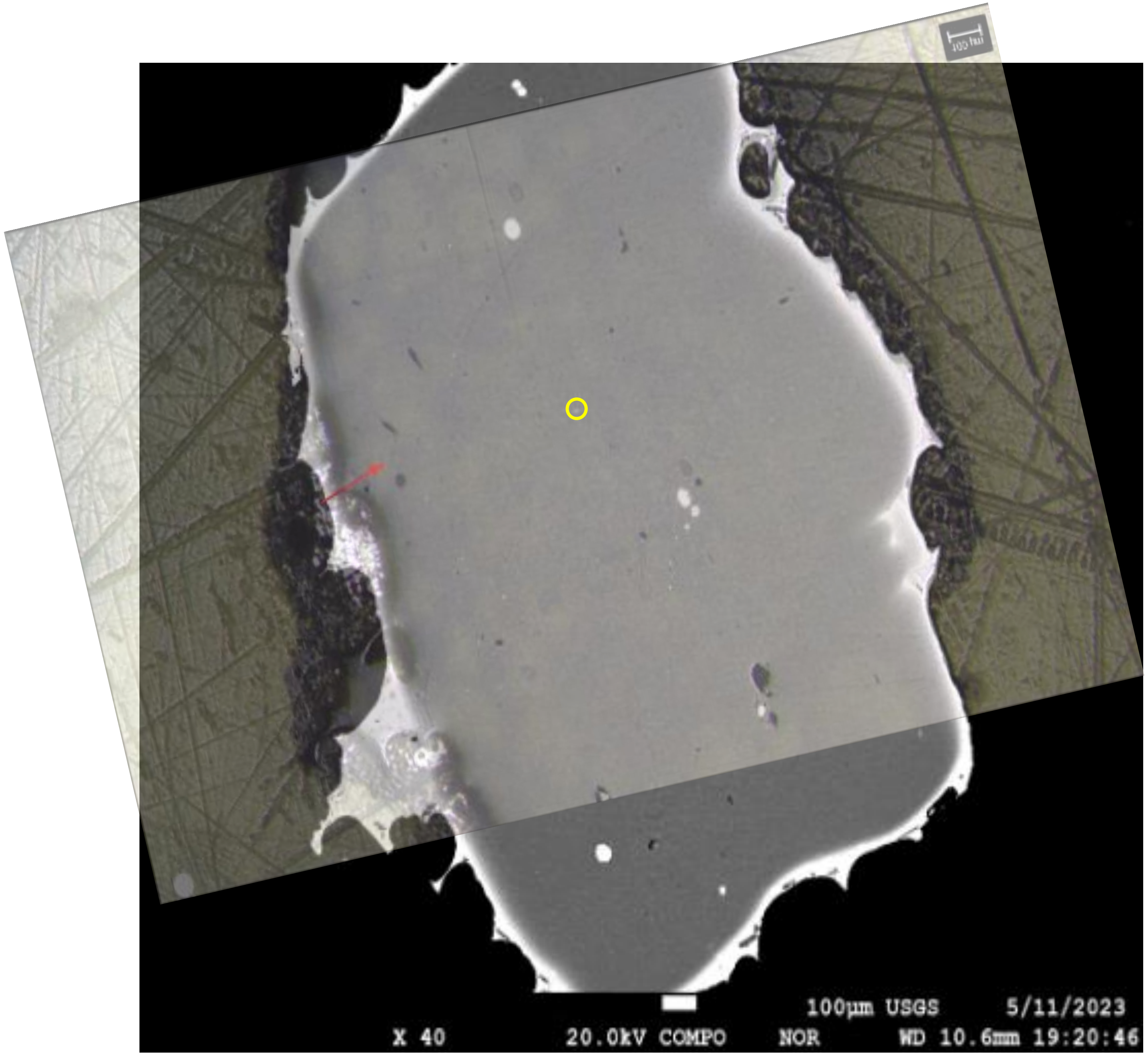

ML_ORI_1_ol1_C

Density: 0.222040931

ML_ORI_1_ol1_G

Density: 0.219817149

ML_ORI_1_ol1_E

Density: 0.209726671

ML_ORI_1_ol1_D

Density: 0.204954806

ML_ORI_1_ol1_H

Density: 0.196792487

ML_ORI_1_ol1_F

Density: 0.19406259

ML_ORI_1_ol1_A

Density: 0.176326962

Key:

Key:
Highest Density
Lowest Density

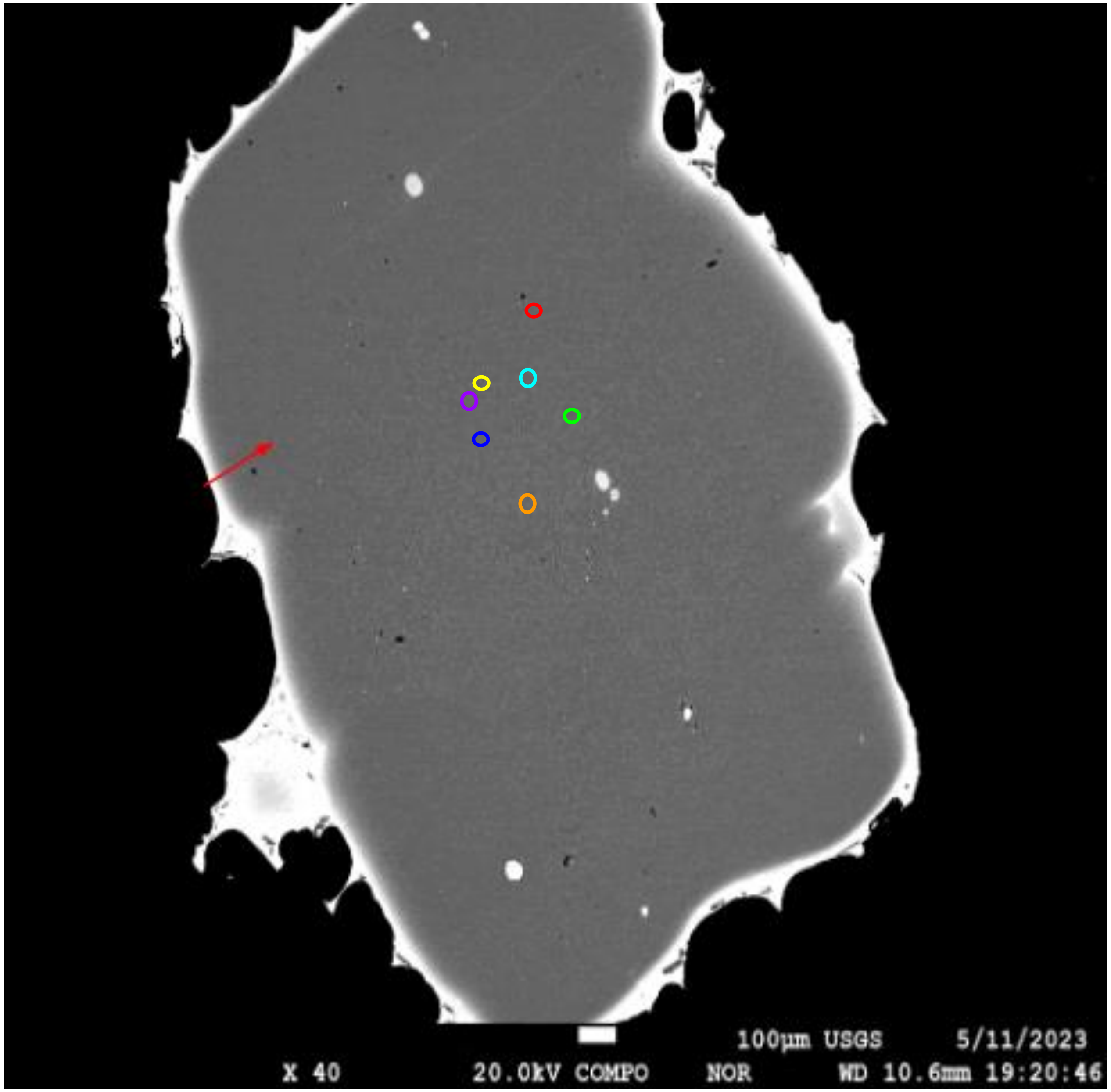

ML_ORI_1_ol4

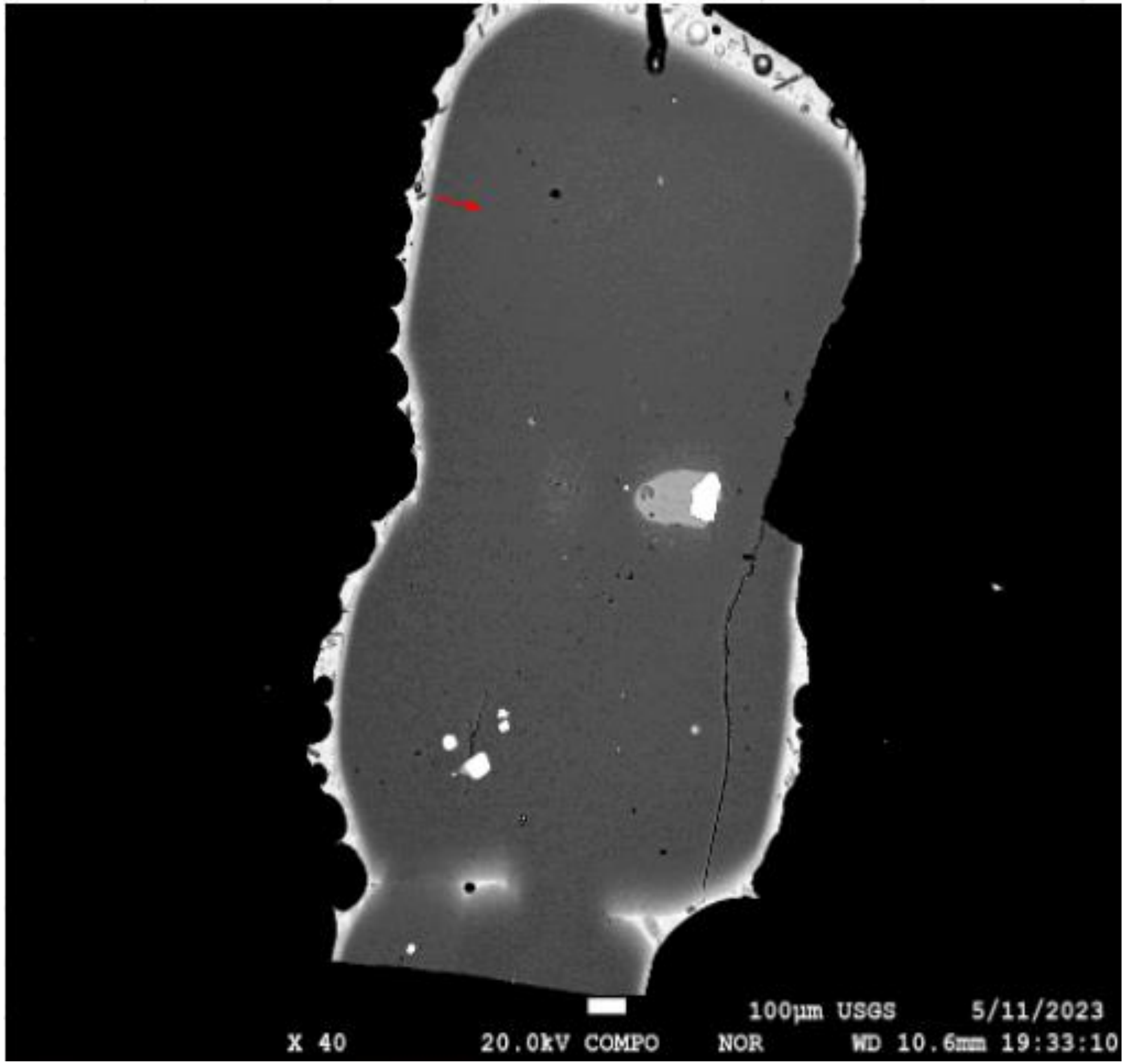

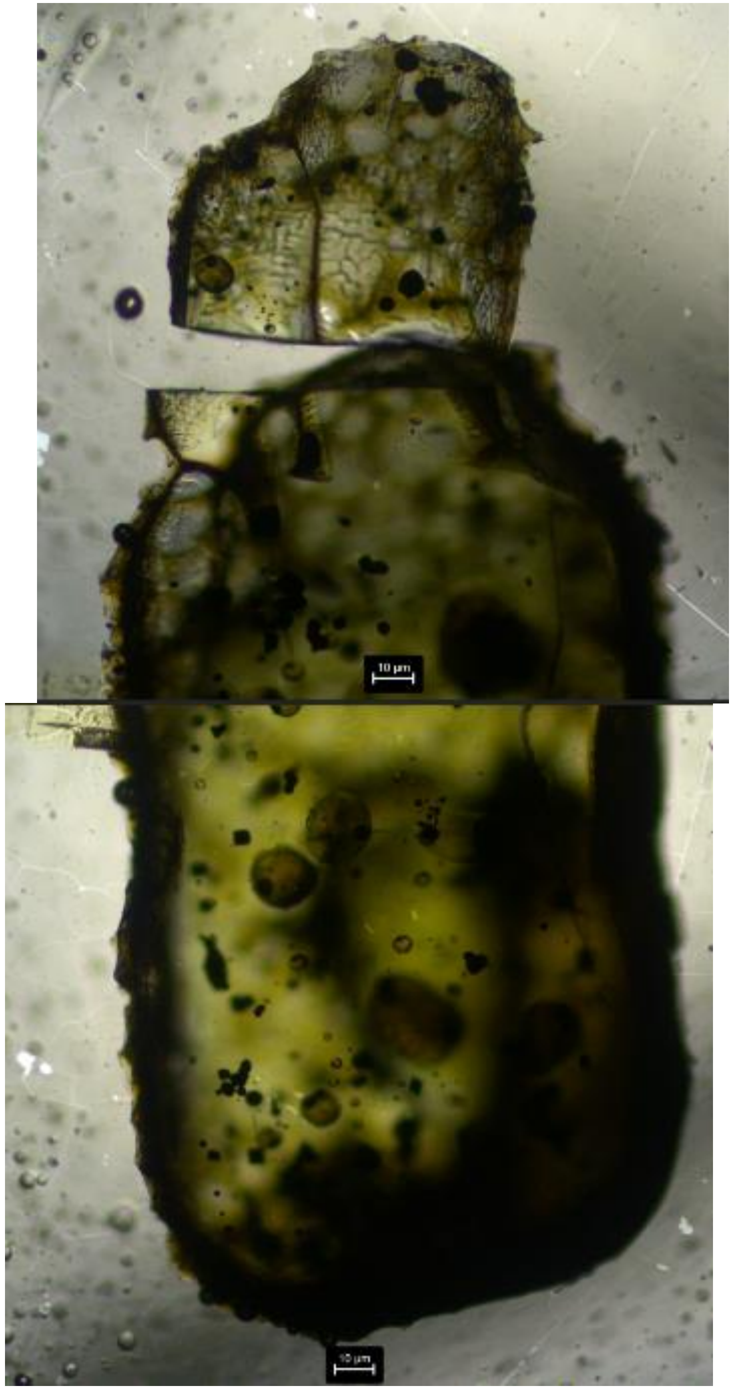

ML_ORI_1_ol4_E

Density: 0.085049984

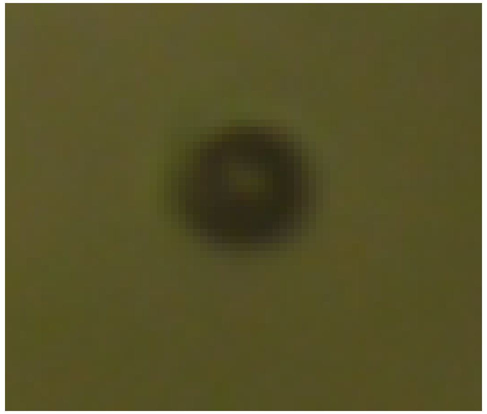

3.03µm

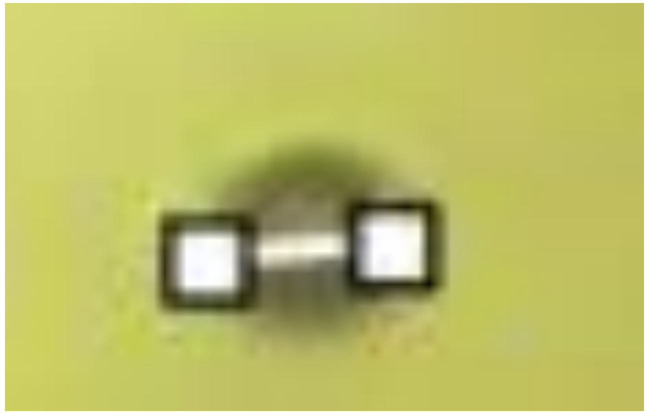

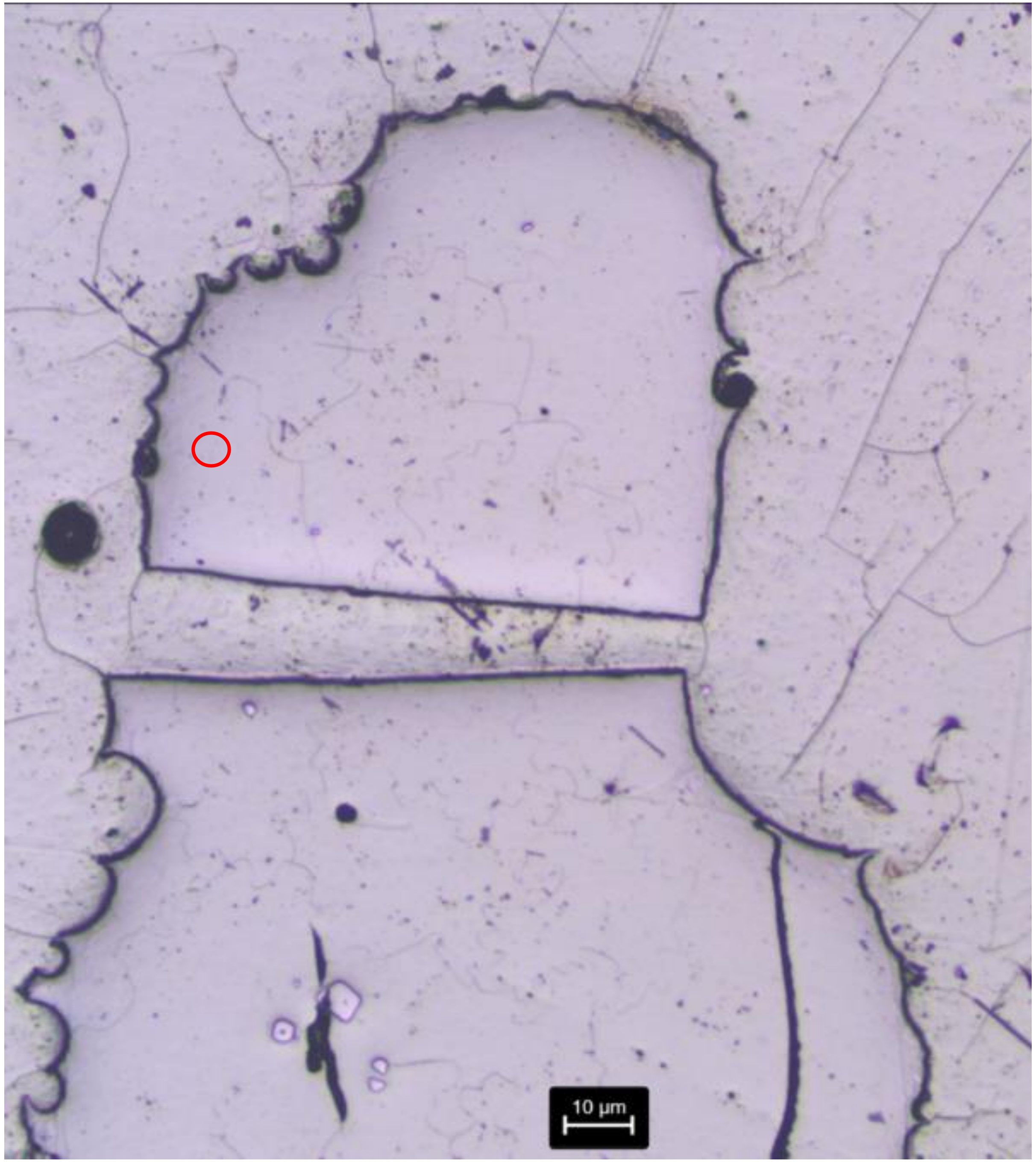

ML_ORI_1_ol4_F

Density: 0.068214784

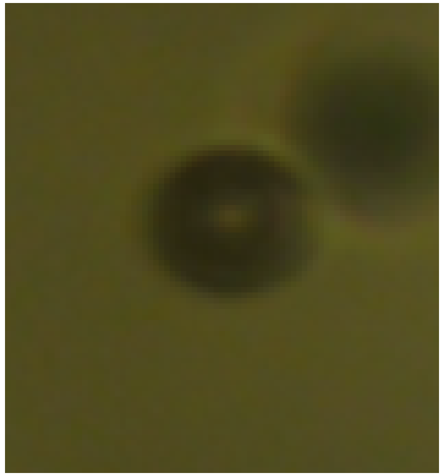

5.97μm

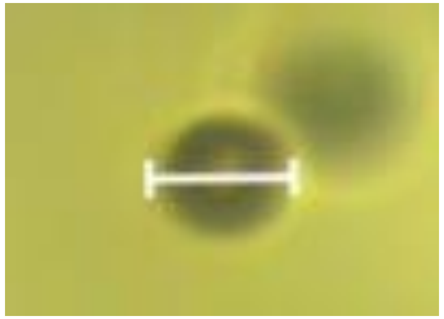

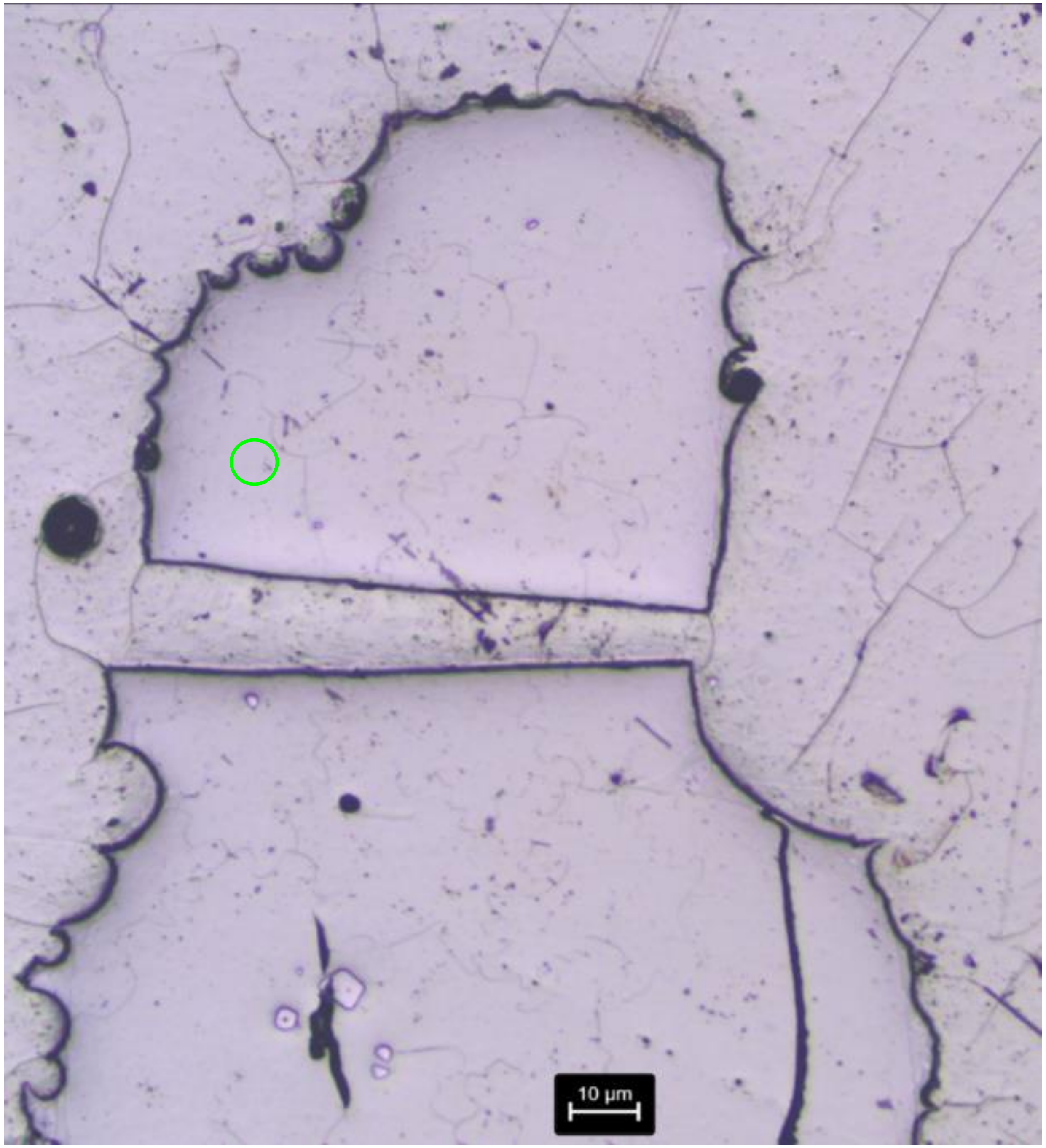

ML_ORI_1_ol4_E

Density: 0.085049984

ML_ORI_1_ol4_F

Density: 0.068214784

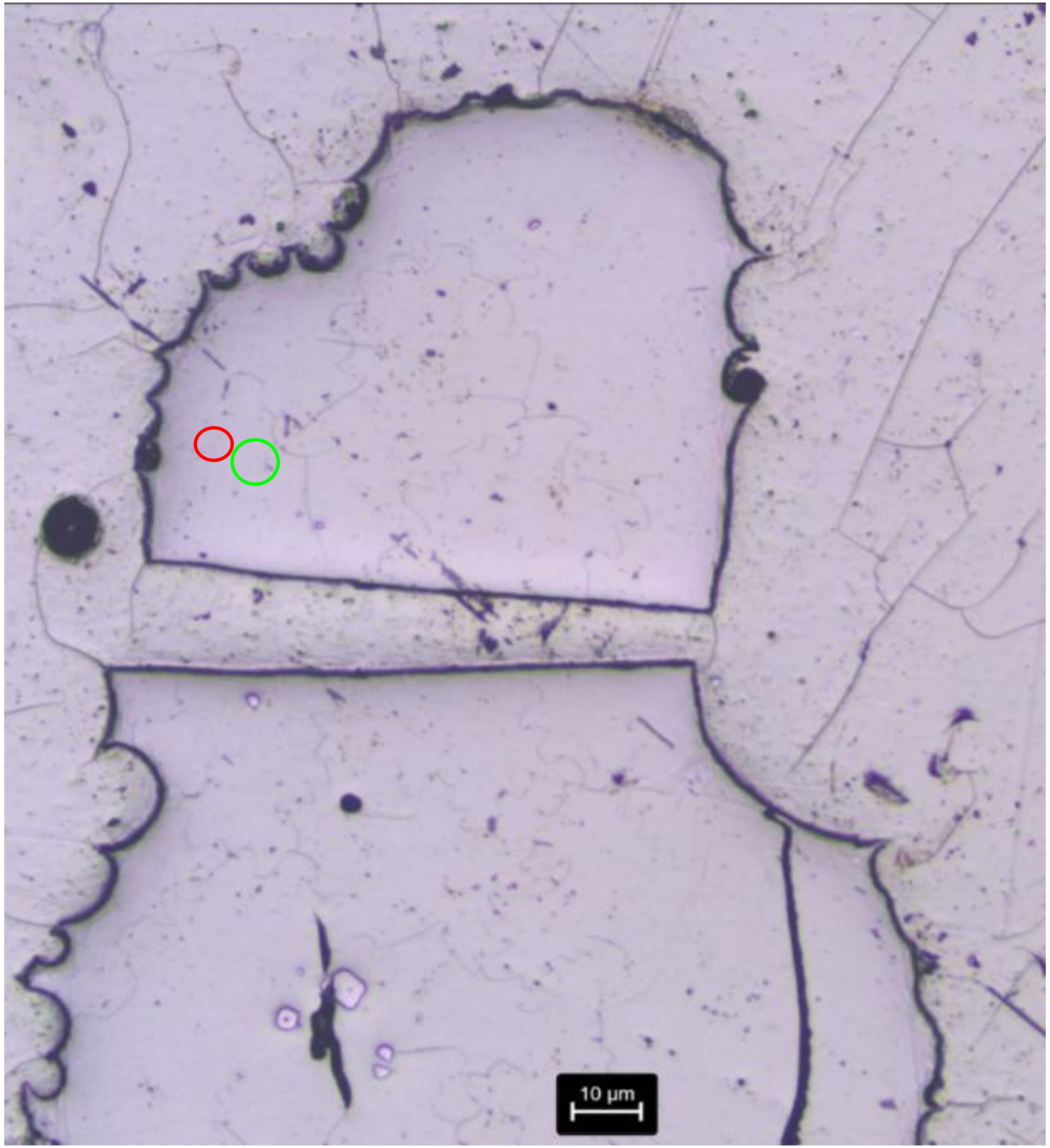

ML_ORI_1_ol5

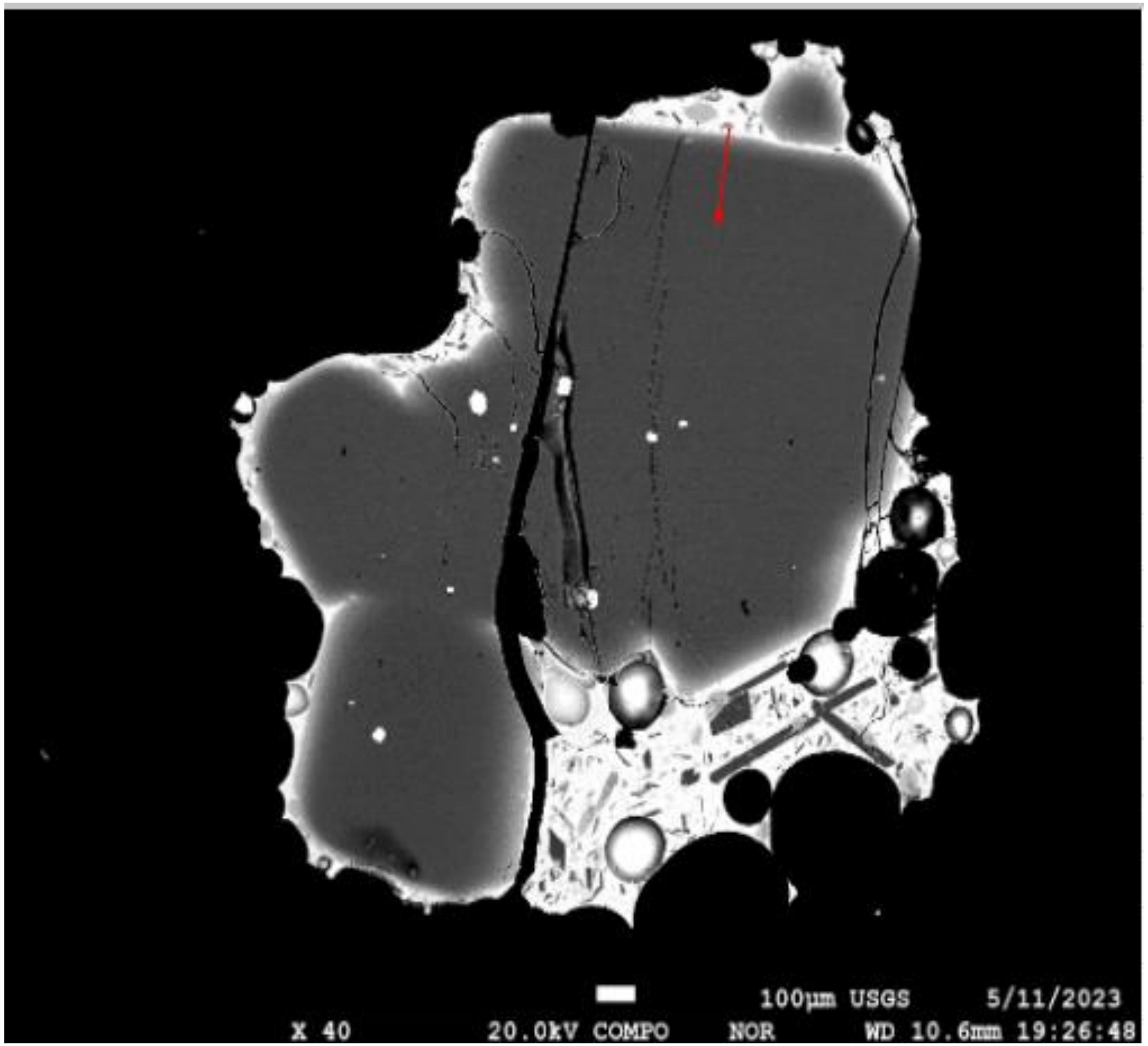

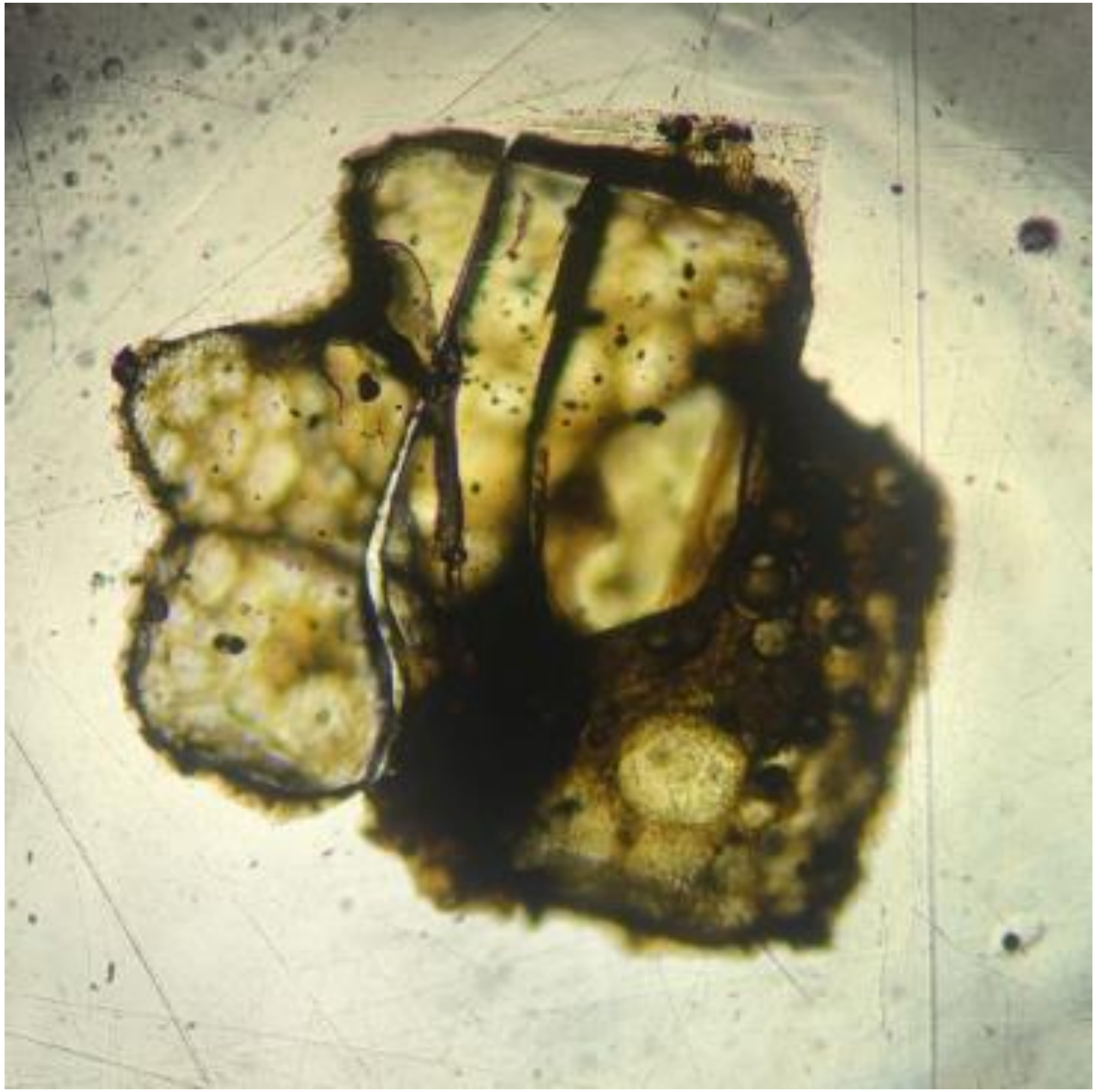

ML_ORI_1_ol5

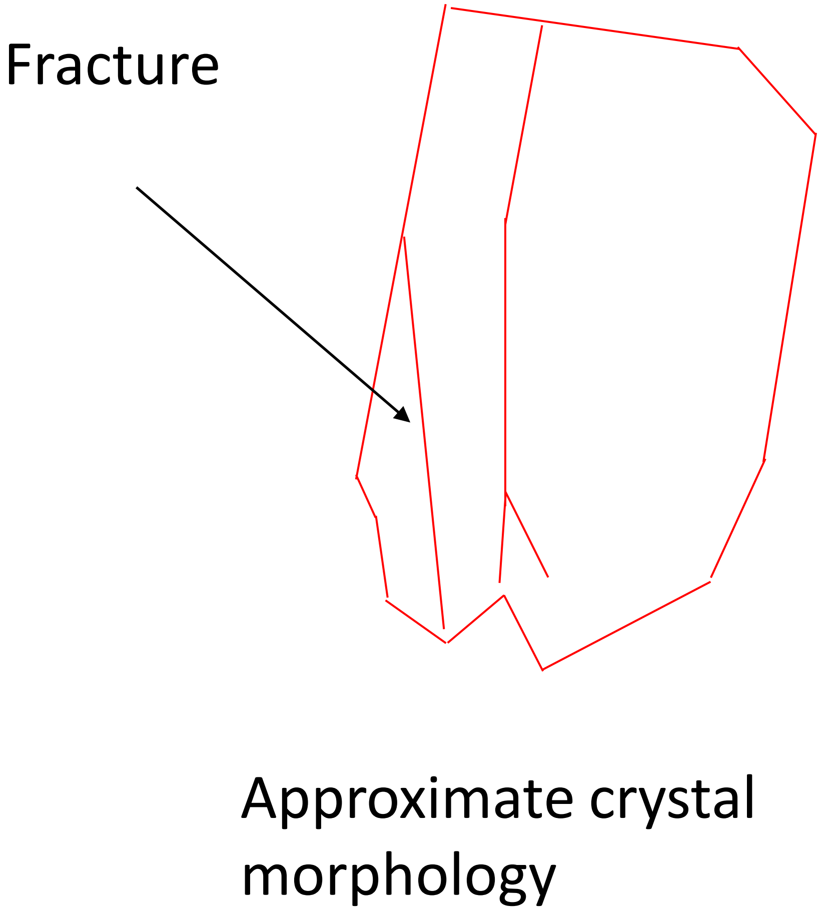

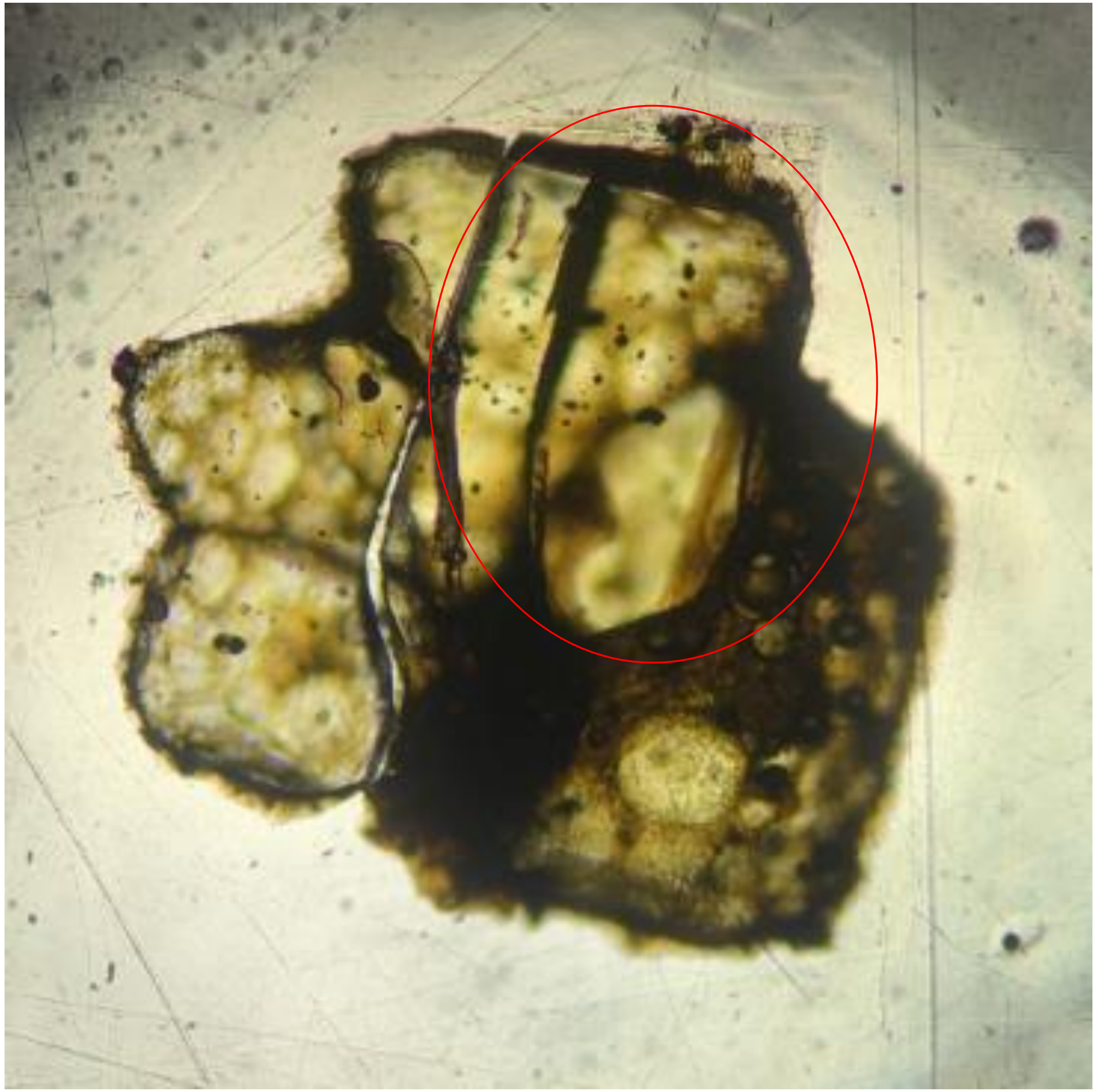

ML_ORI_1_ol5_B

Density: 0.244401115

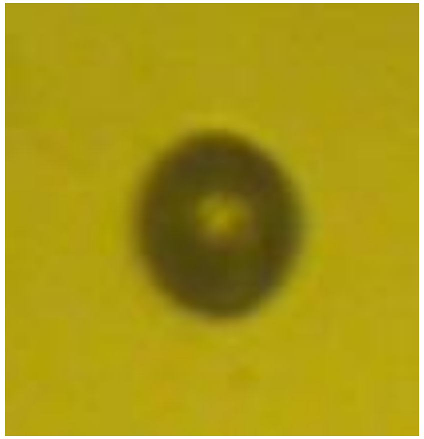

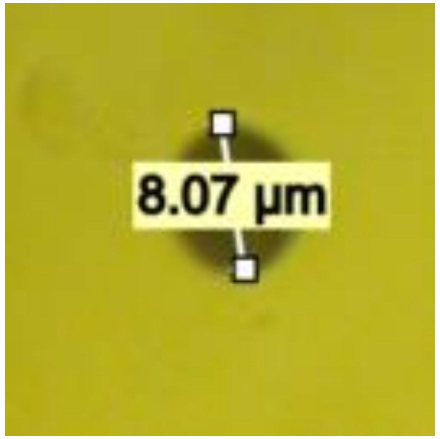

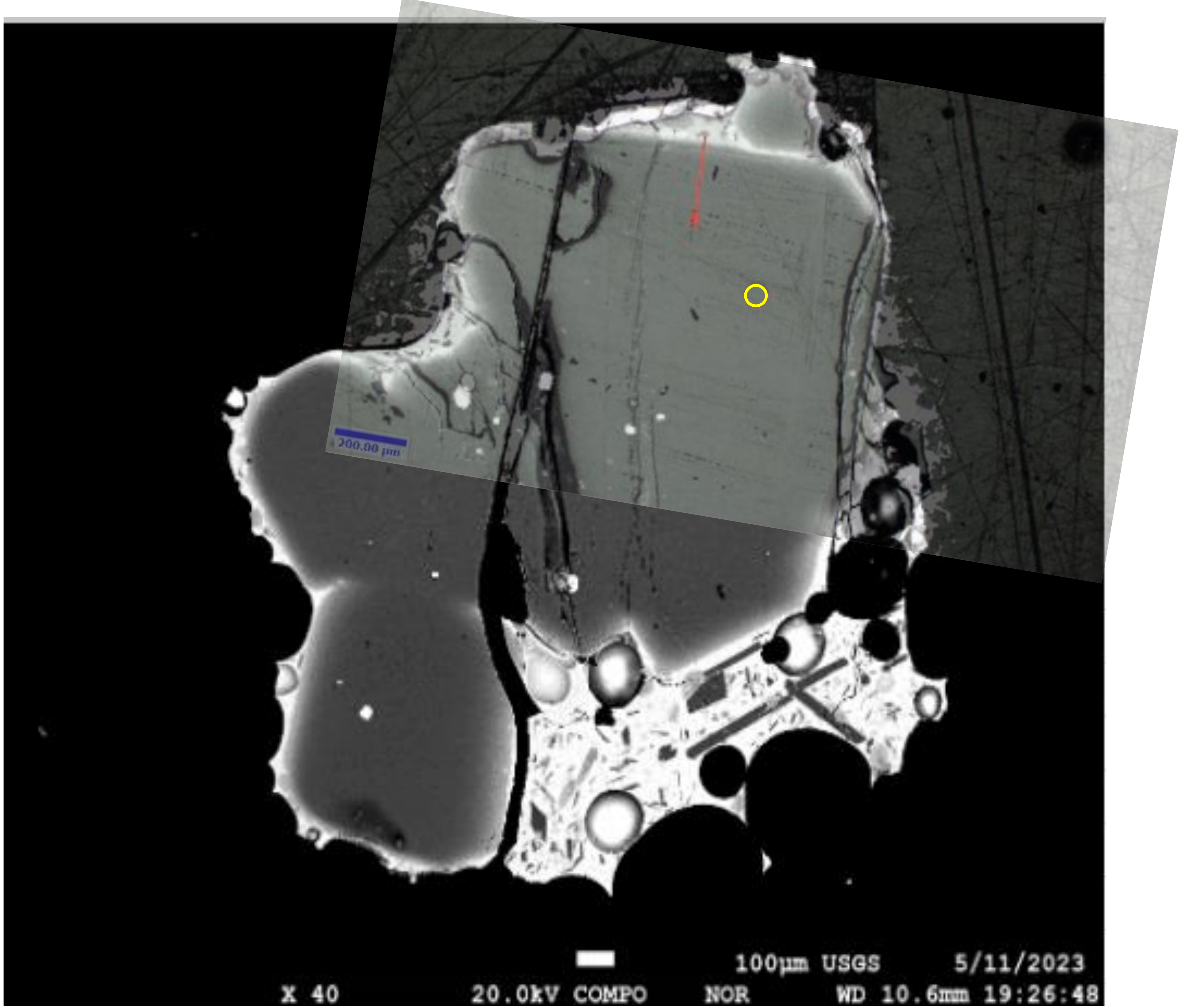

ML_ORI_1_ol5_C

Density: 0.229099469

*Lowest in crystal

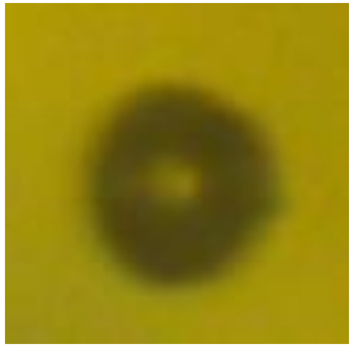

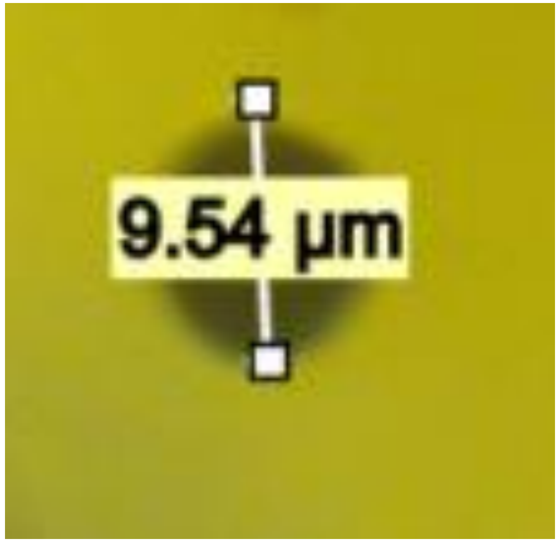

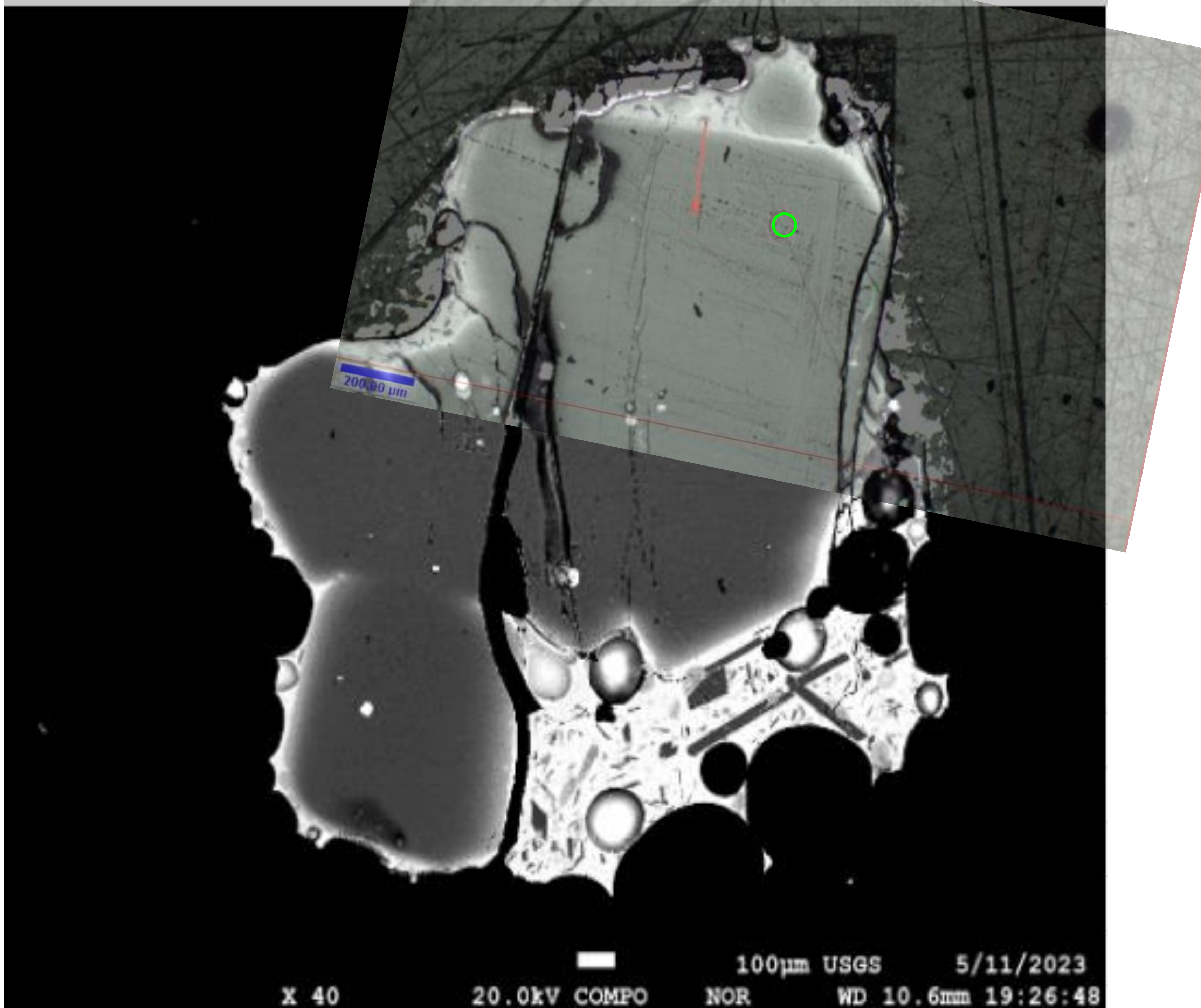

ML_ORI_1_ol5_D

Density: 0.338542272

*Highest in crystal

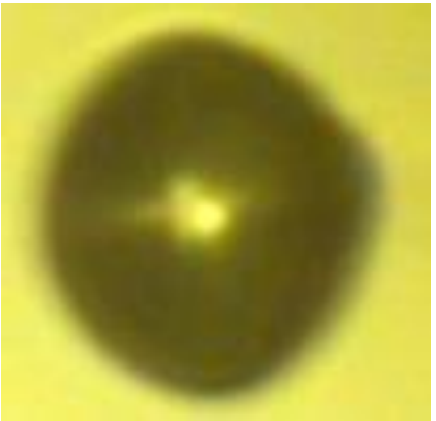

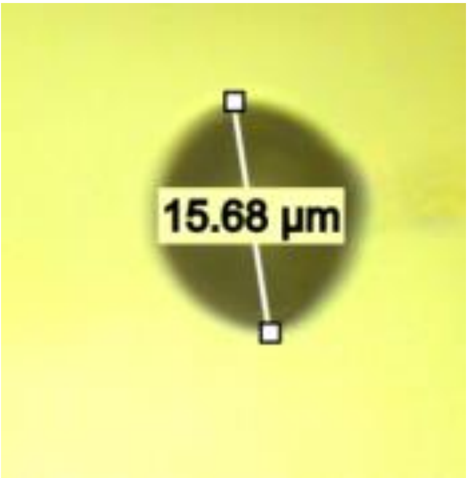

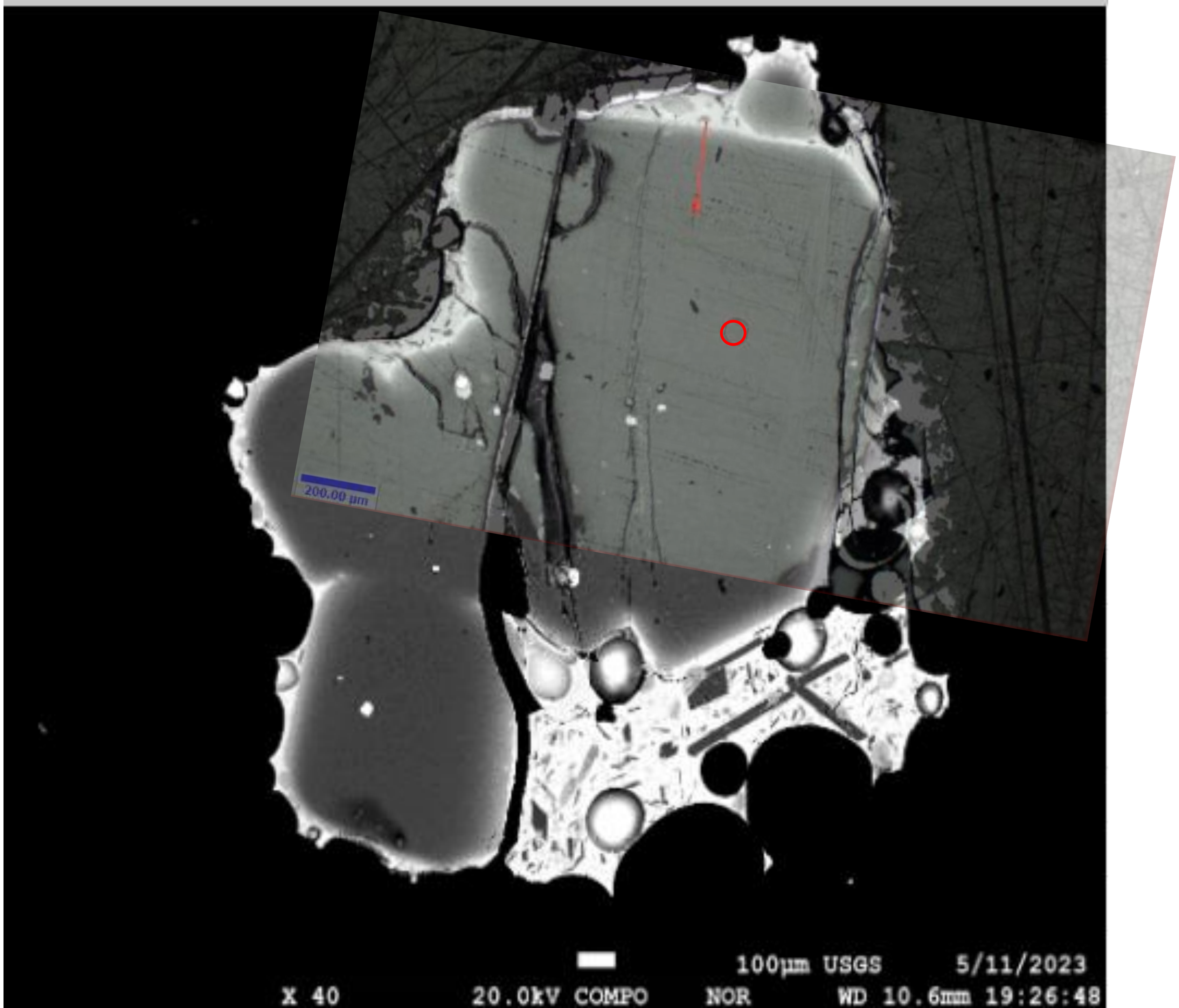

ML_ORI_1_ol5_E

Density: 0.257518406

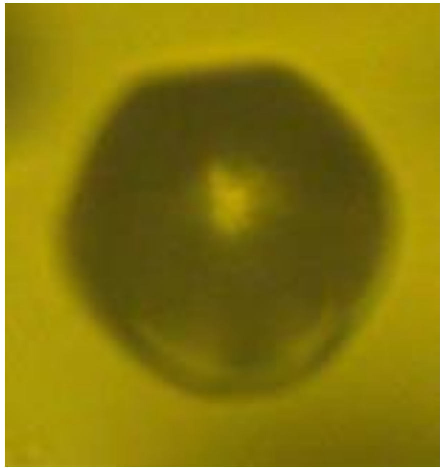

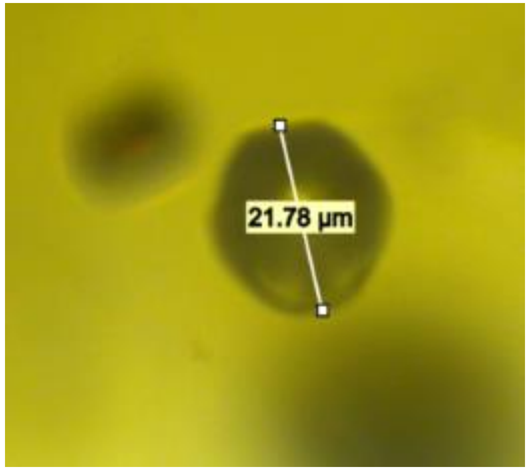

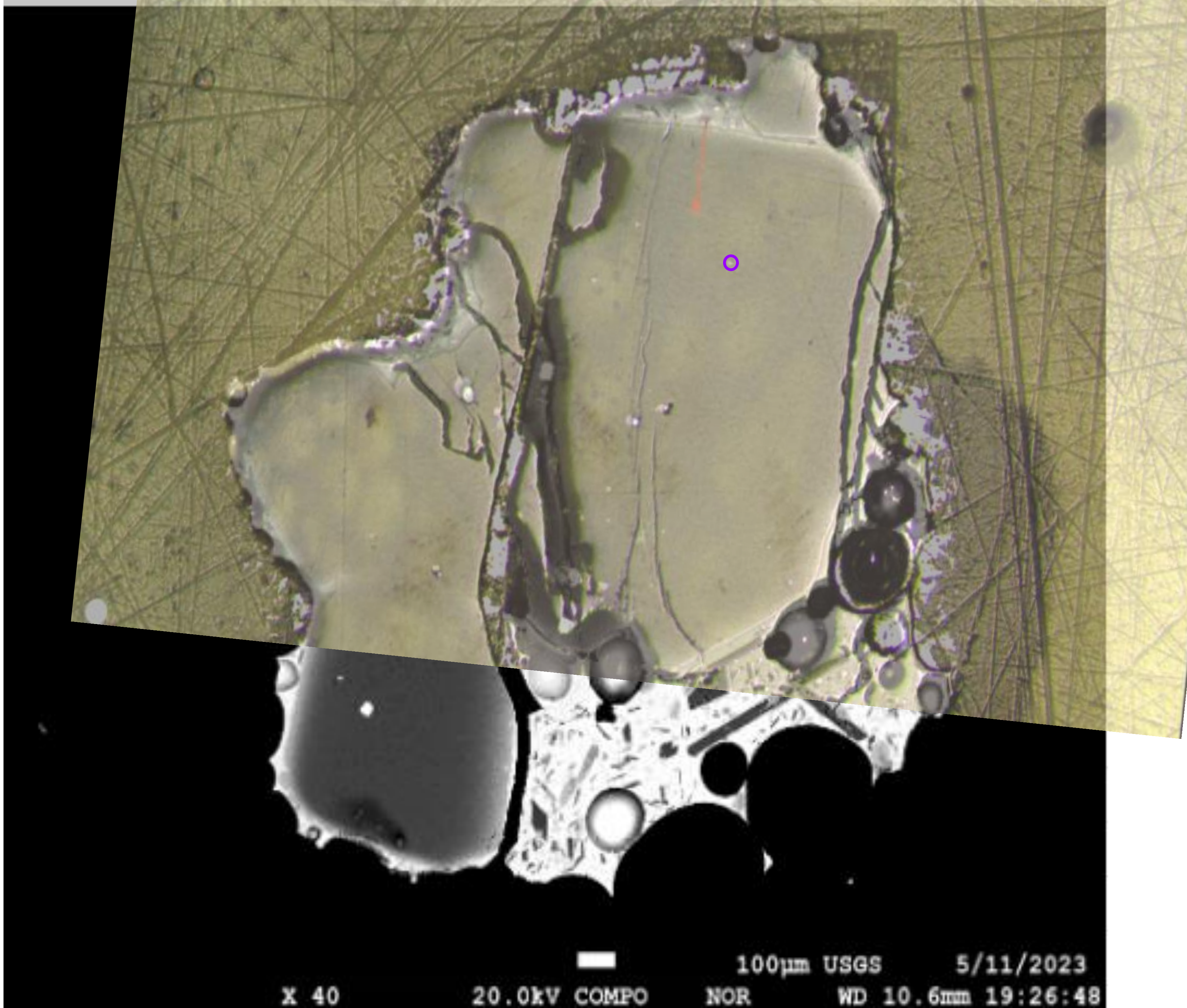

ML_ORI_1_ol5_F

Density: 0.268740334

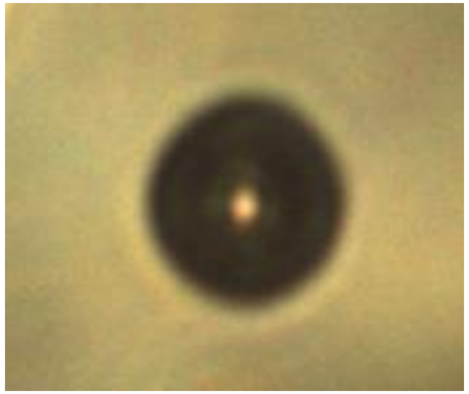

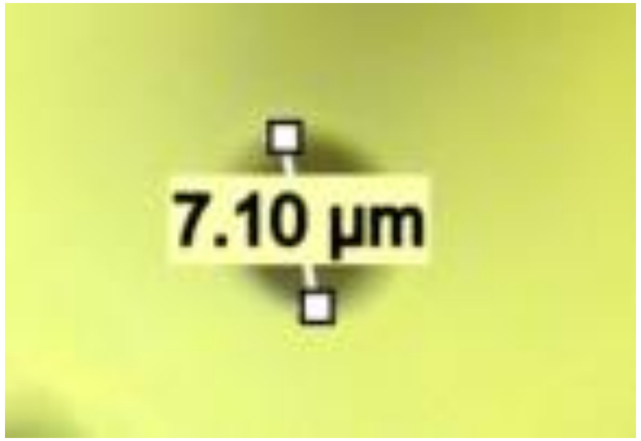

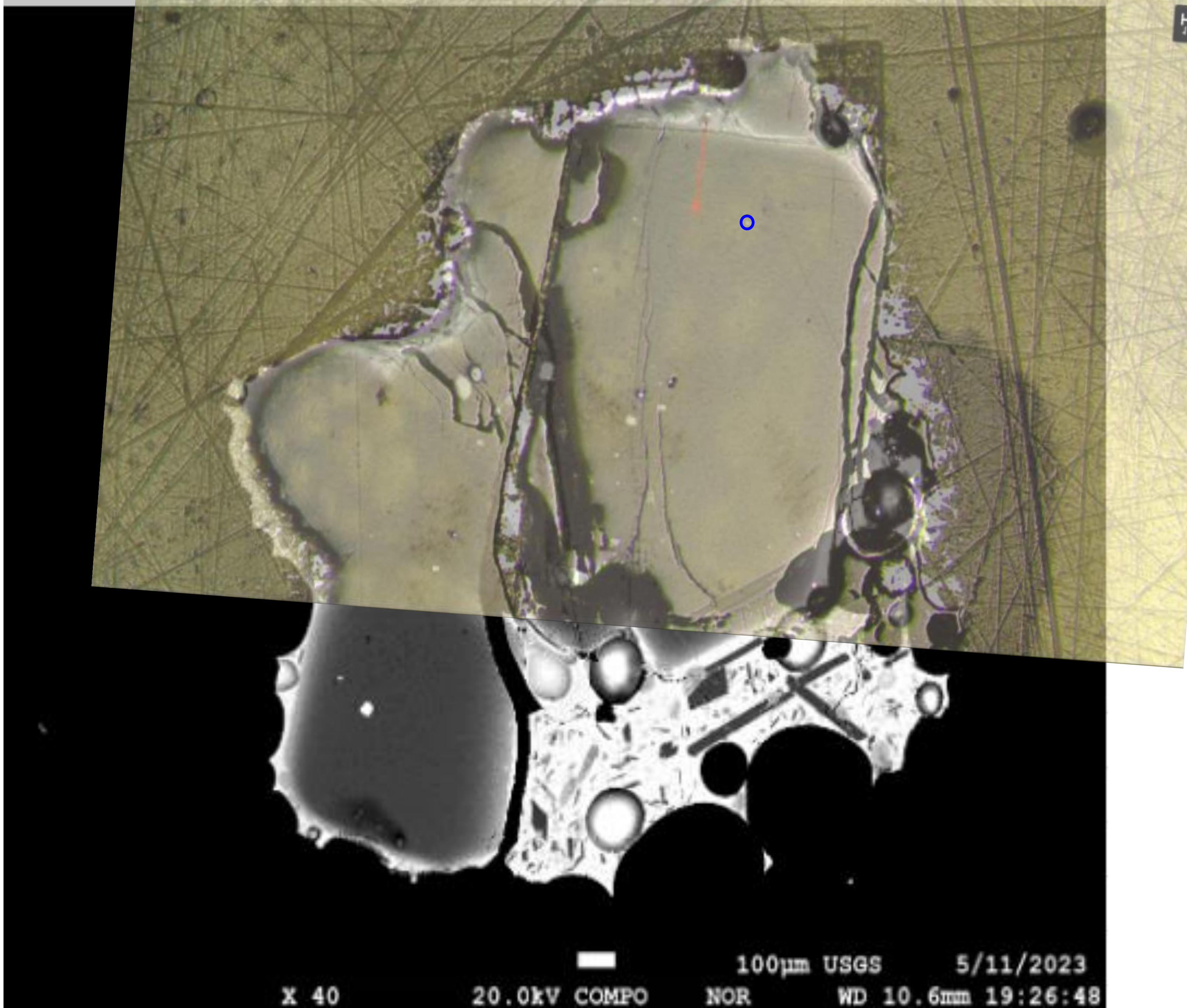

ML_ORI_1_ol5_G

Density: 0.277198361

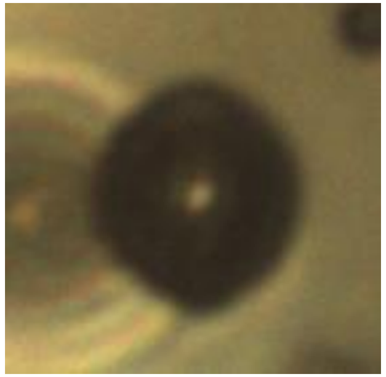

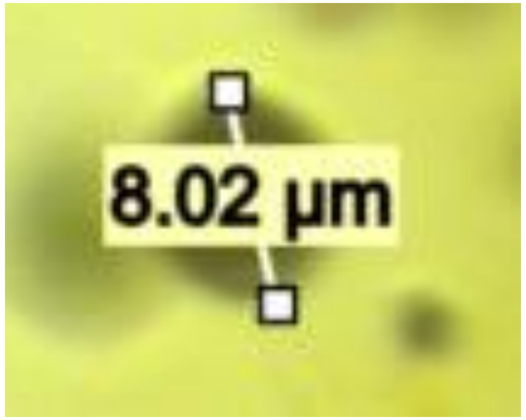

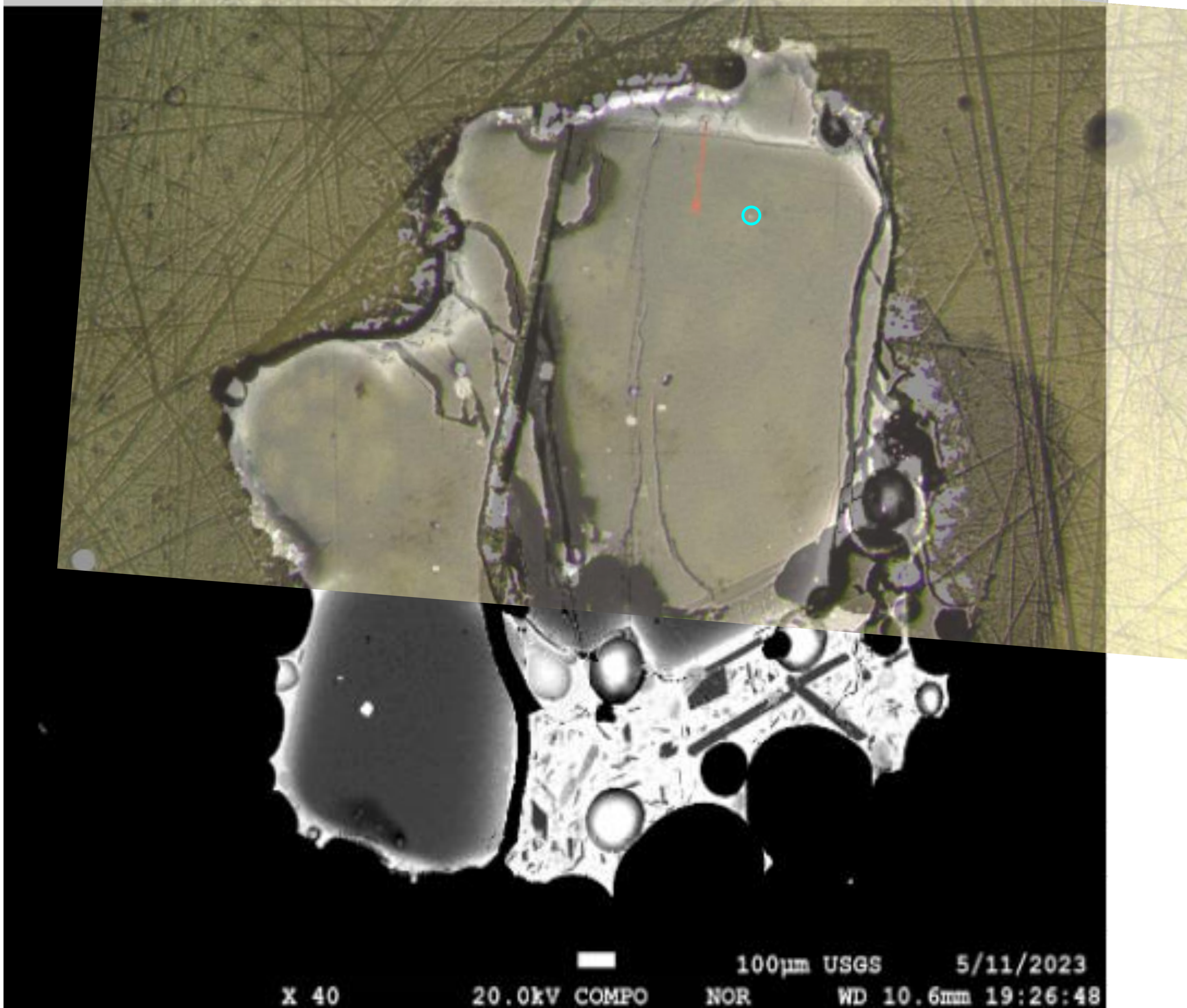

ML_ORI_1_ol5_D

Density: 0.338542272
*Highest in crystal

ML_ORI_1_ol5_G

Density: 0.277198361

ML_ORI_1_ol5_F

Density: 0.268740334

ML_ORI_1_ol5_E

Density: 0.257518406

ML_ORI_1_ol5_B

Density: 0.244401115

ML_ORI_1_ol5_C

Density: 0.229099469
*Lowest in crystal

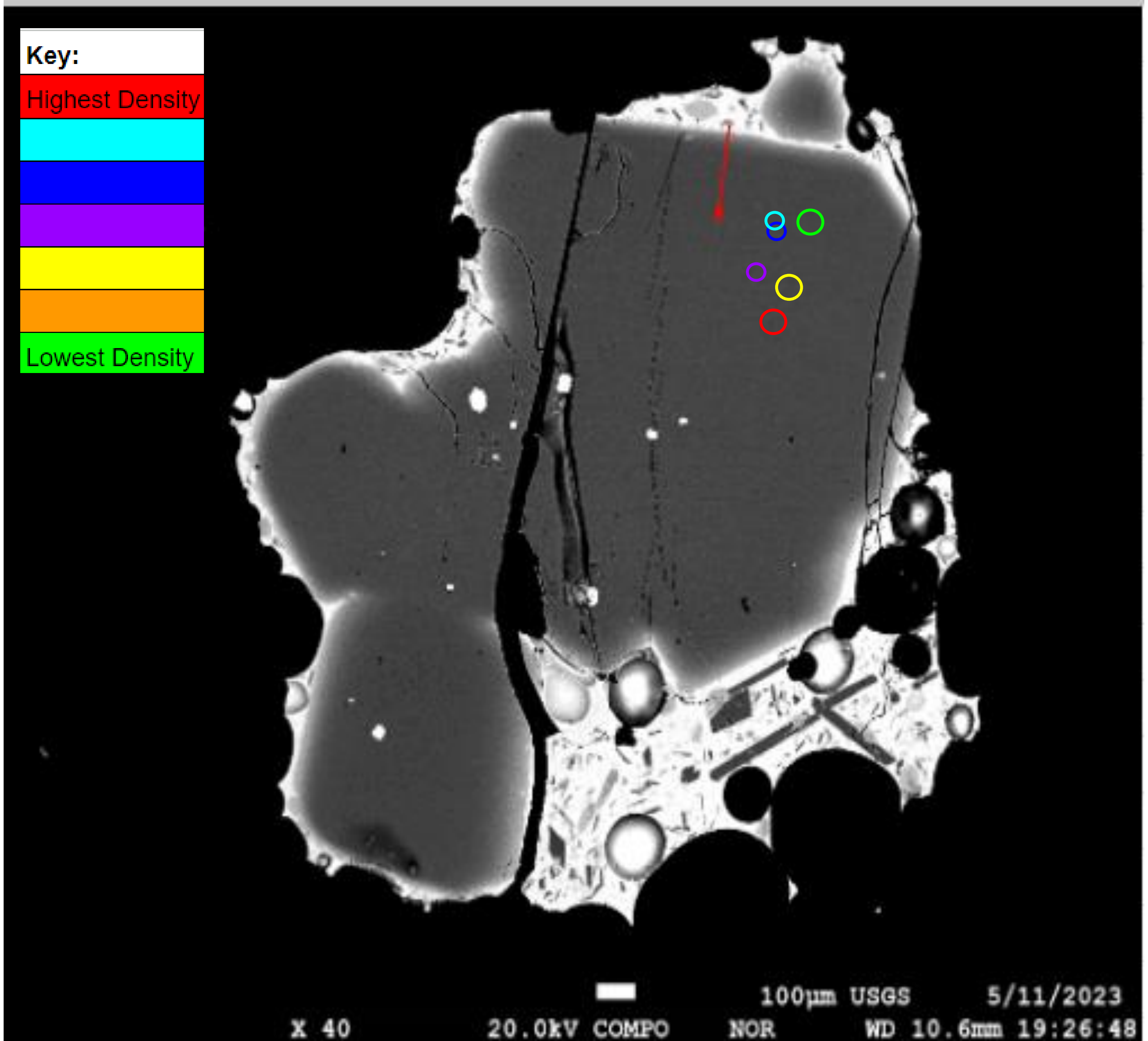

ML_ORI_1_ol7

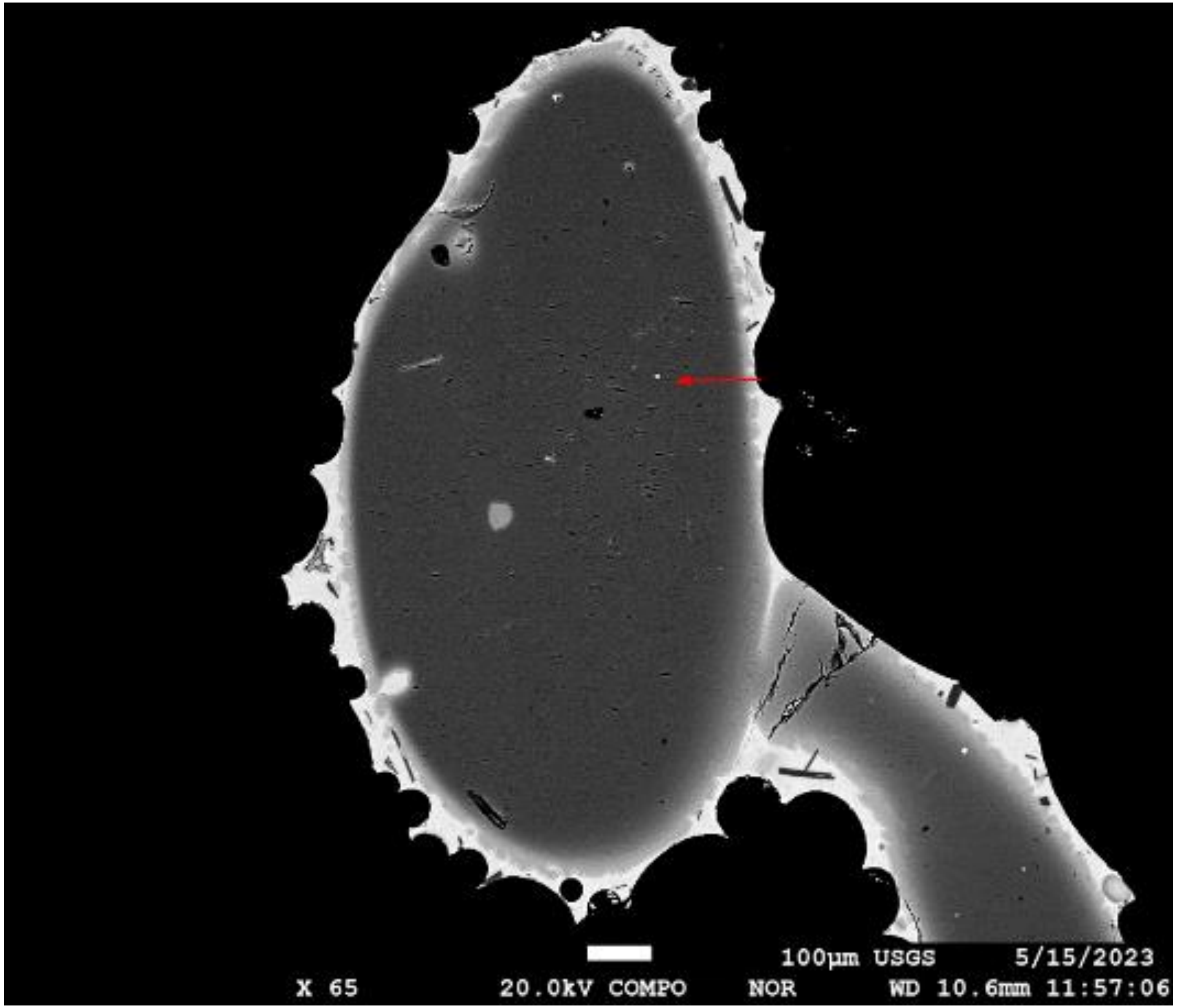

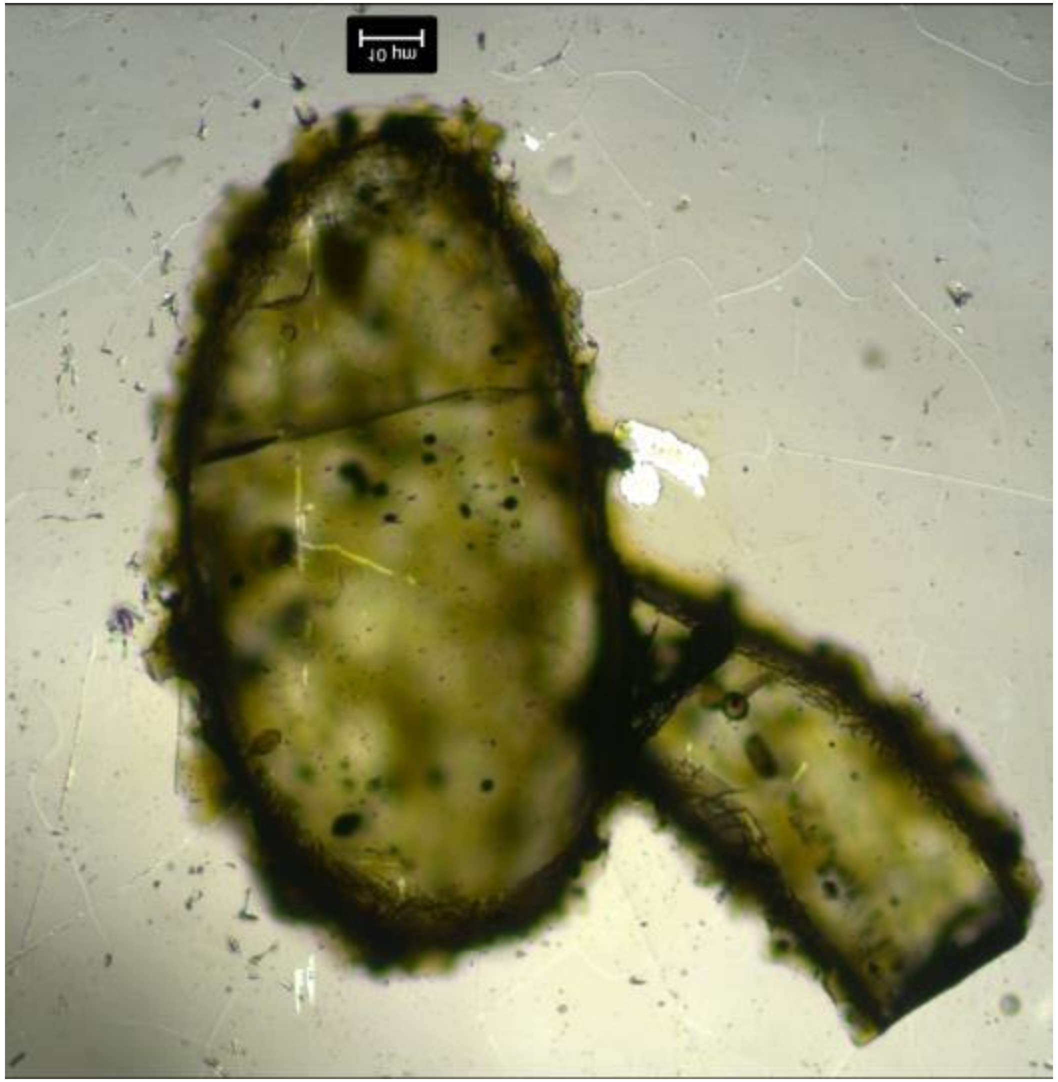

ML_ORI_1_ol7_A

Density: 0.031426483

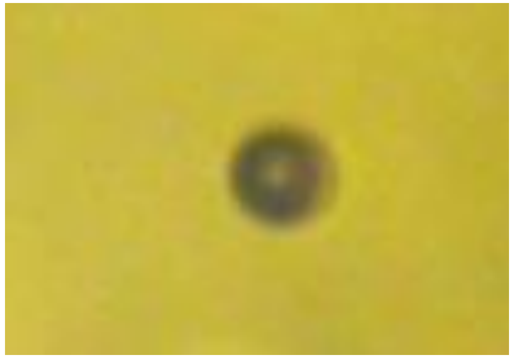

4.20µm

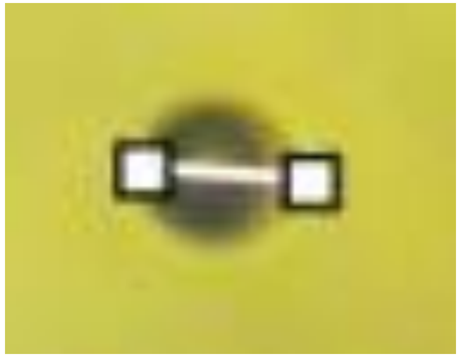

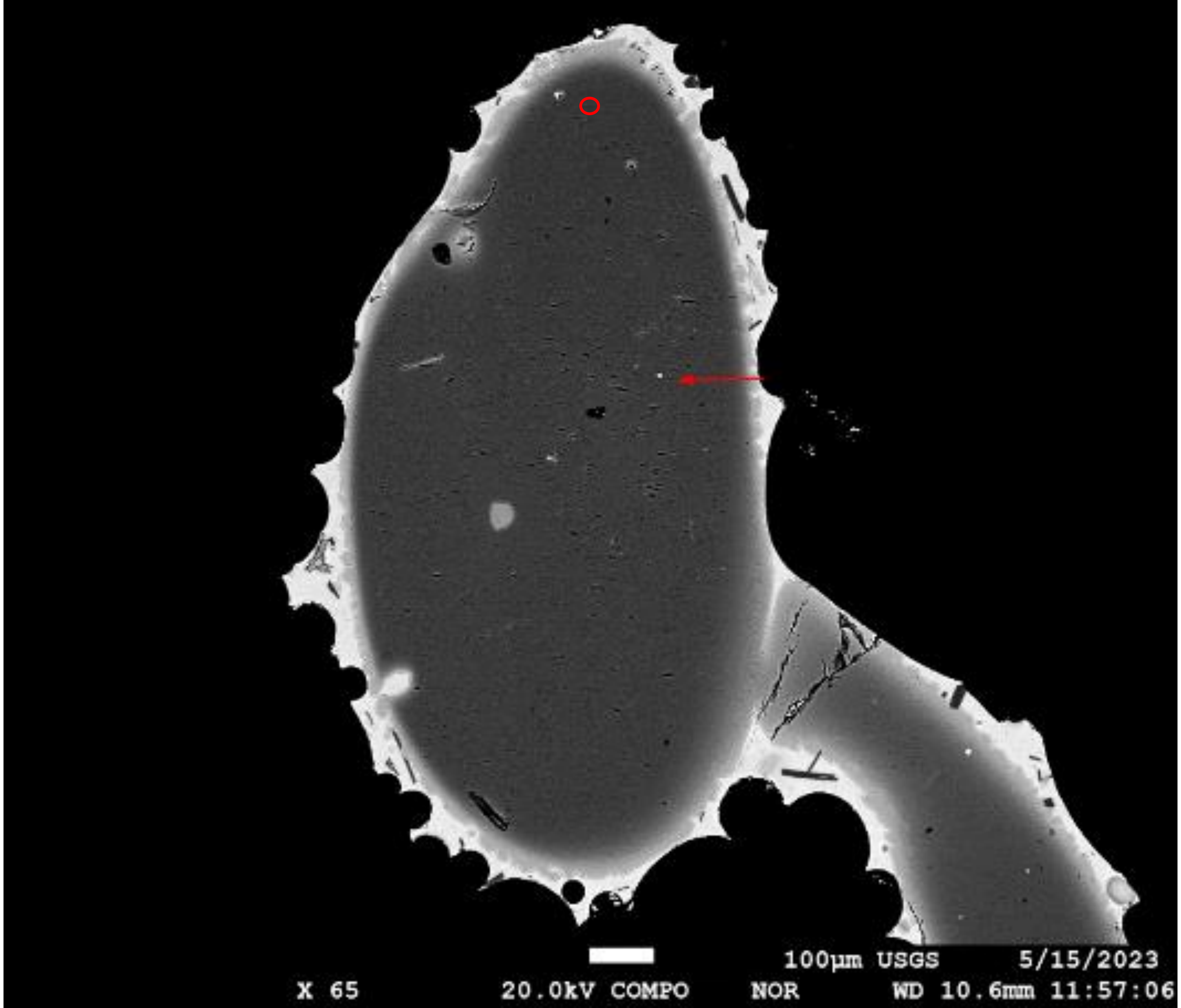

ML_ORI_1_ol9

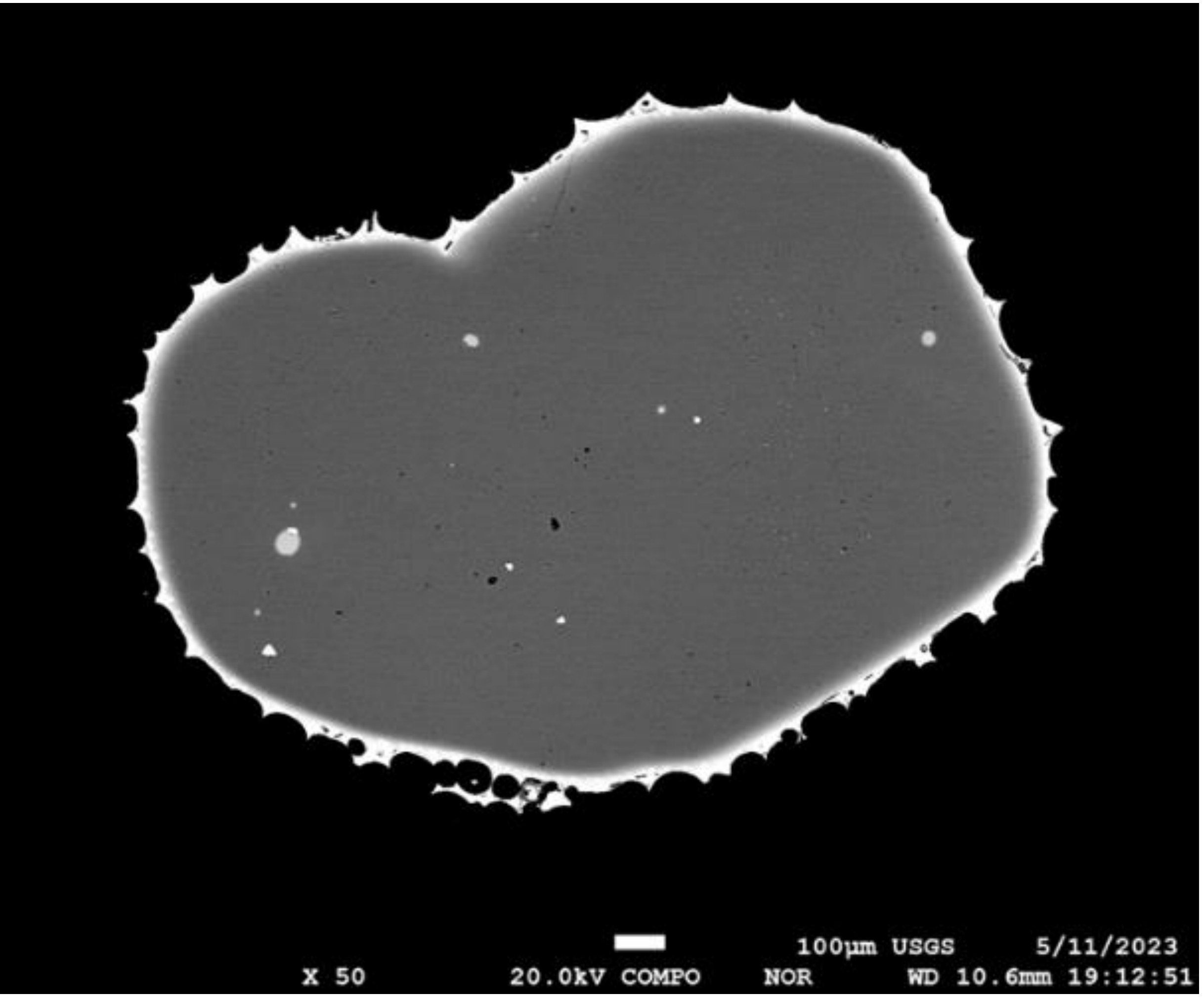

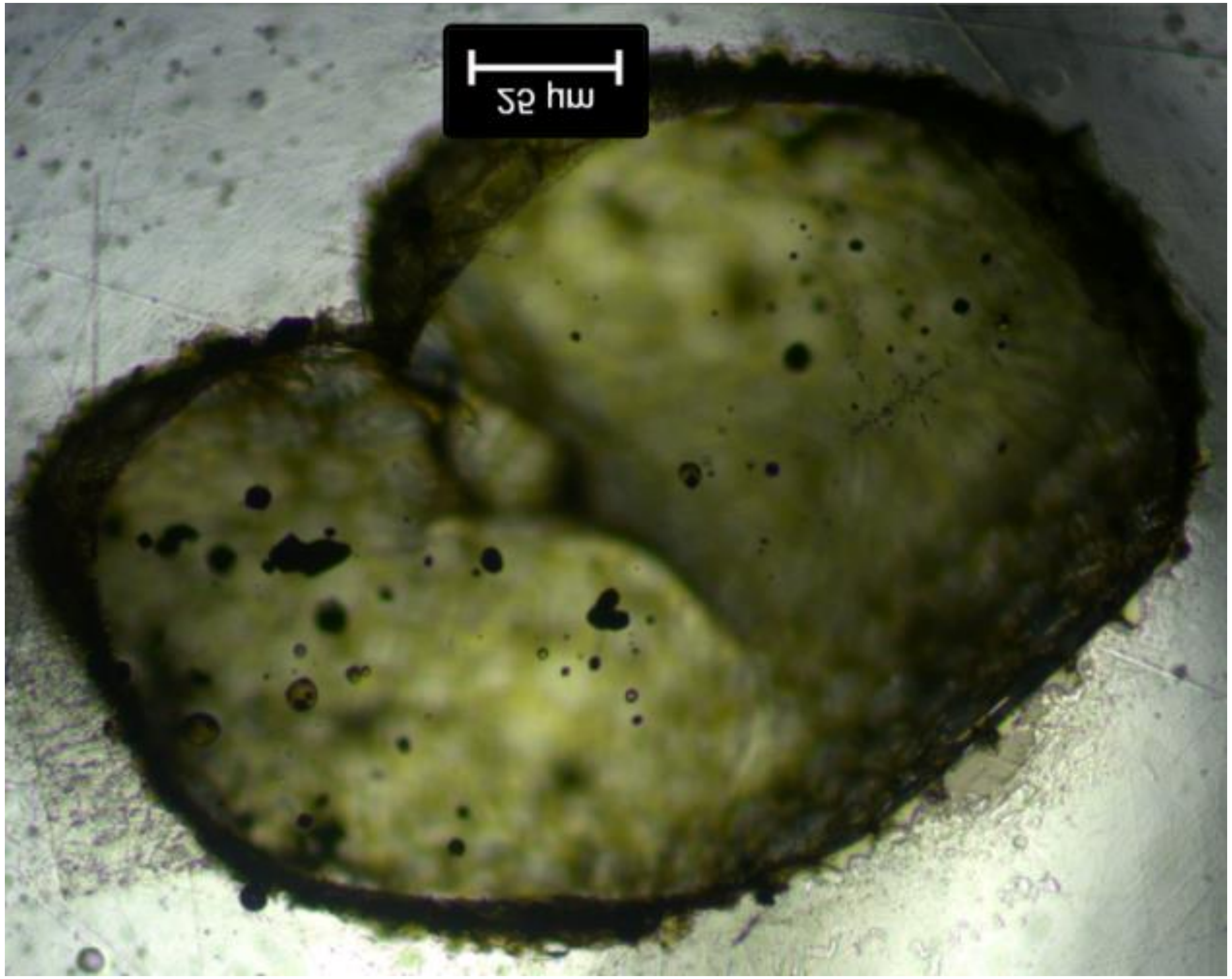

ML_ORI_1_ol9_A

Density: 0.250875966

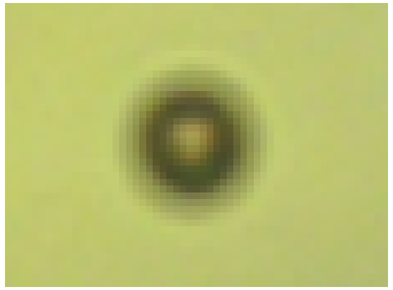

3.65µm

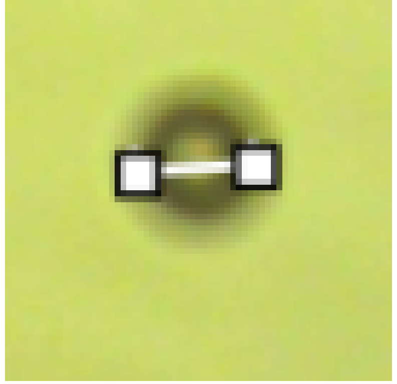

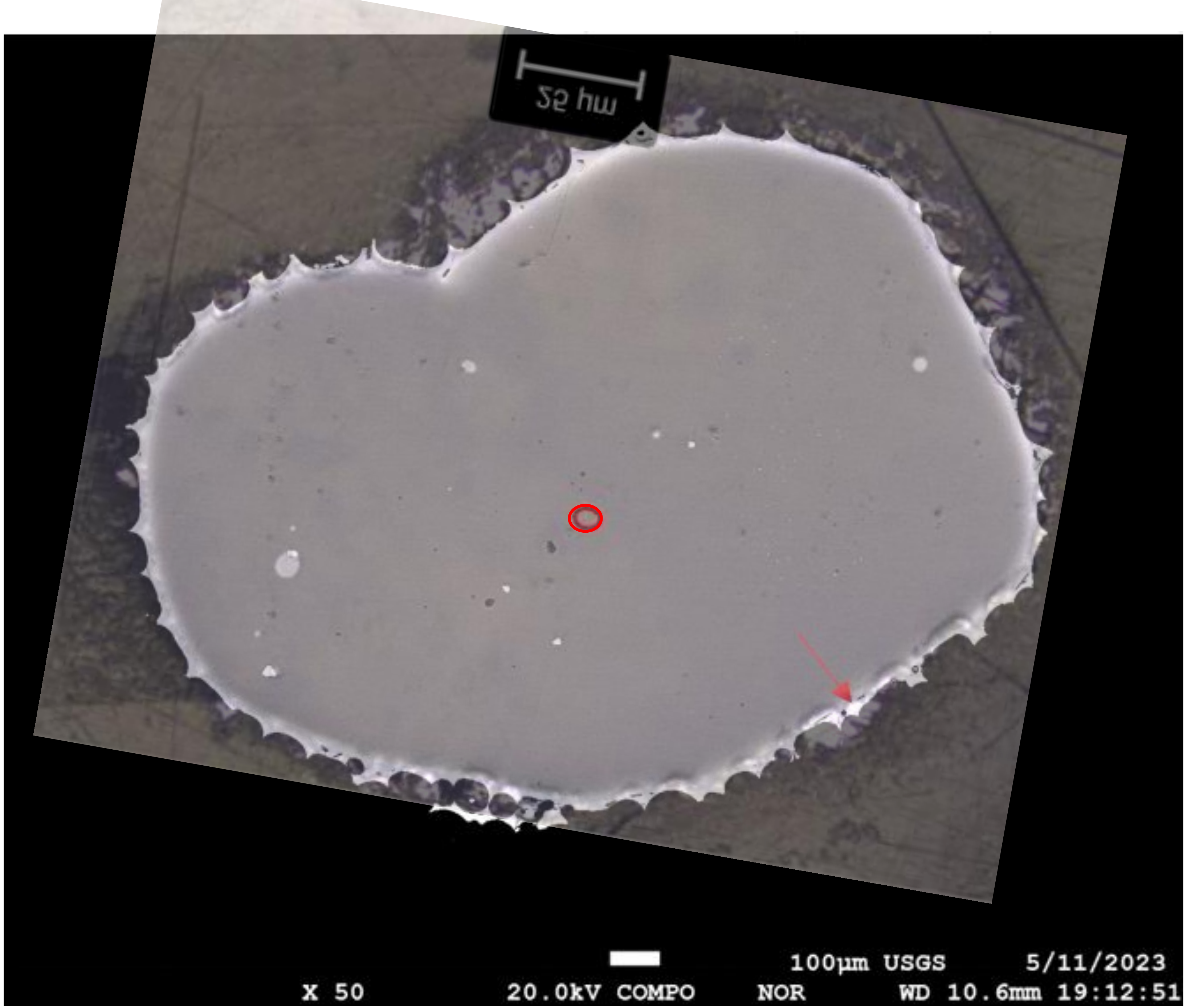

ML_ORI_1_ol15

NO EPMA

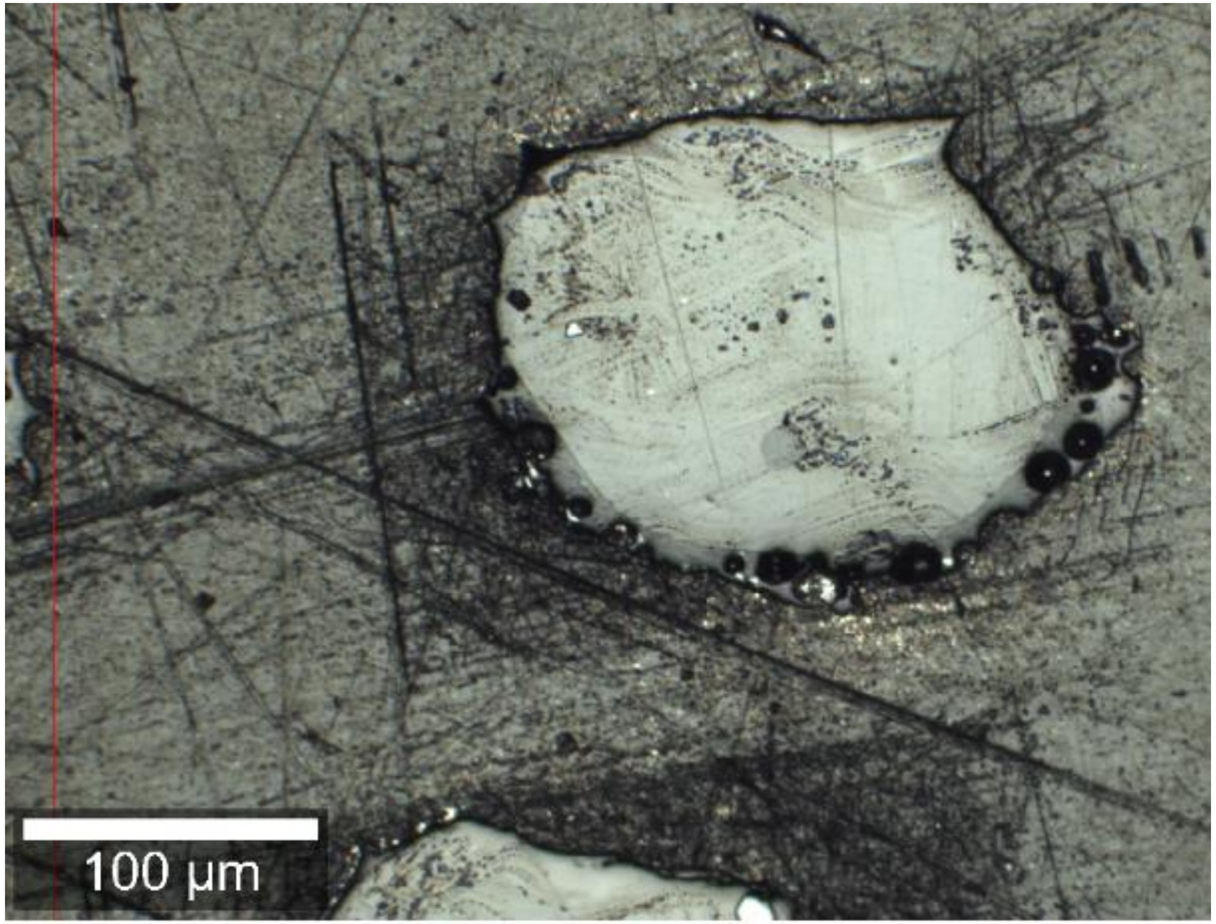

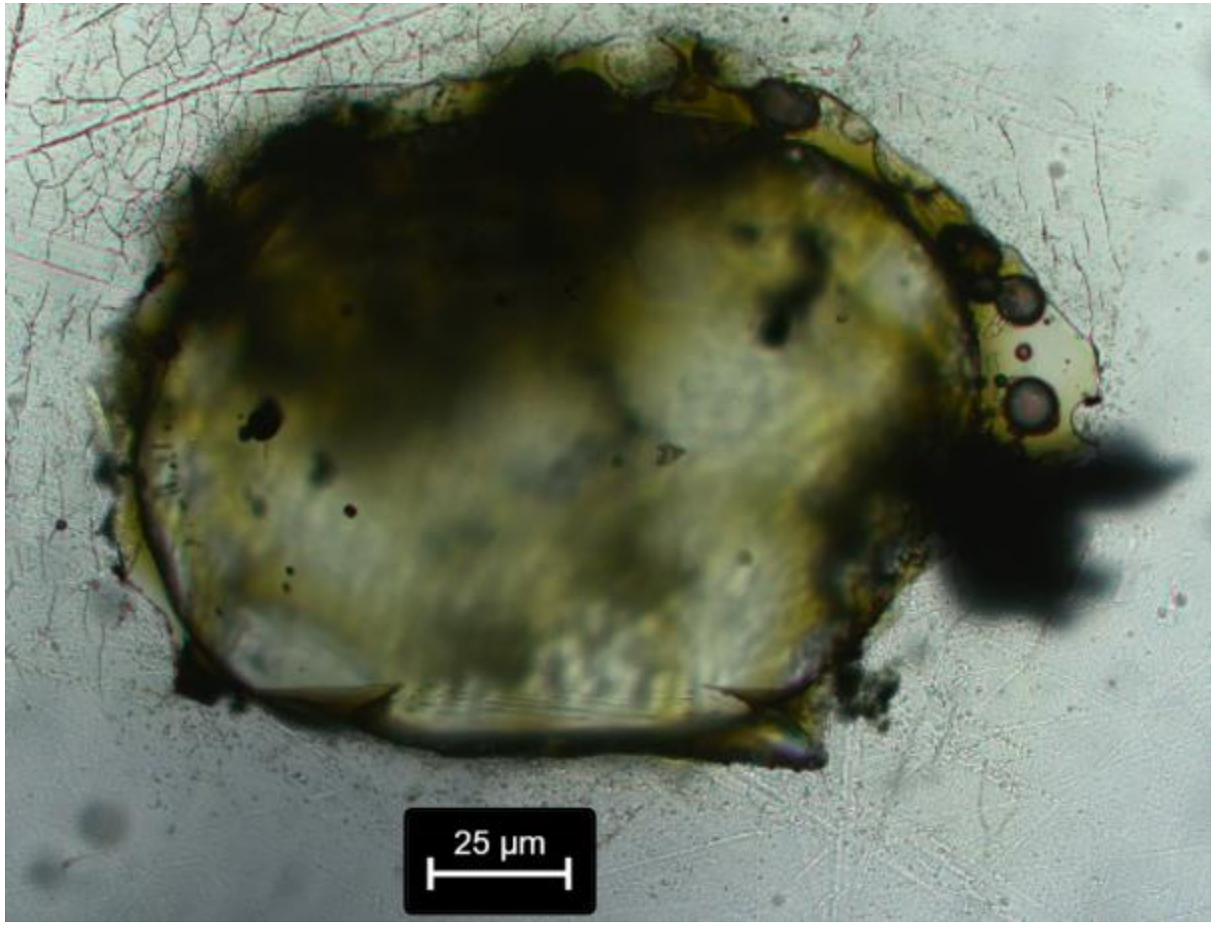

ML_ORI_1_ol15_B

Density: 0.094450675

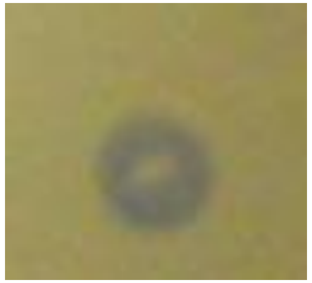

5.47µm

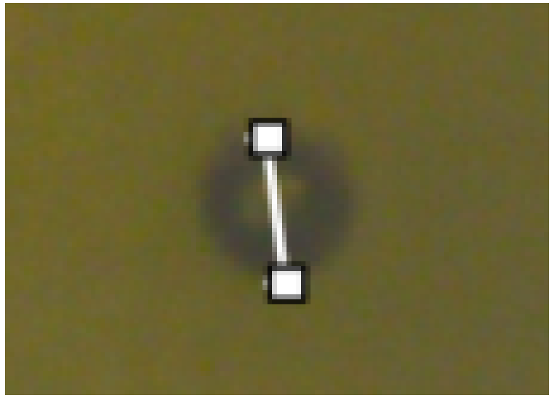

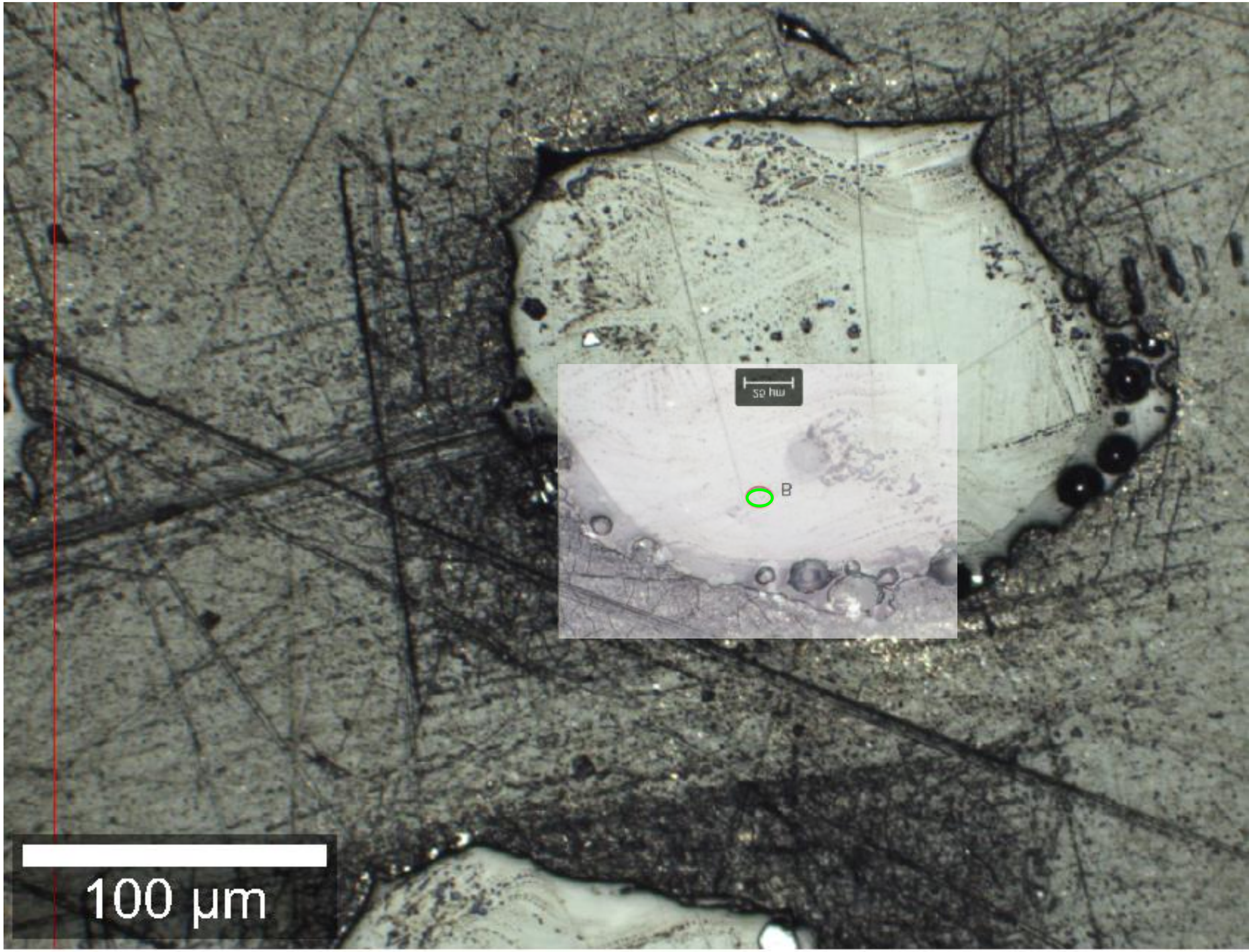

ML_ORI_1_ol15_C

Density: 0.110375196

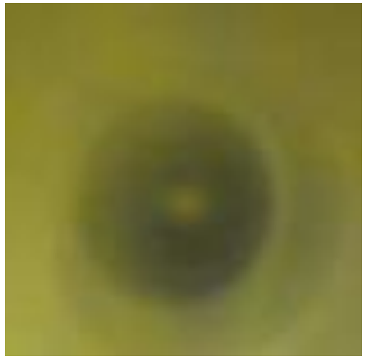

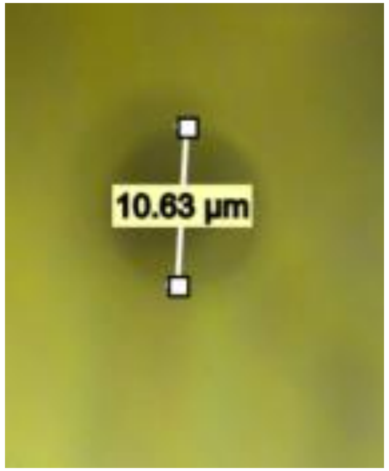

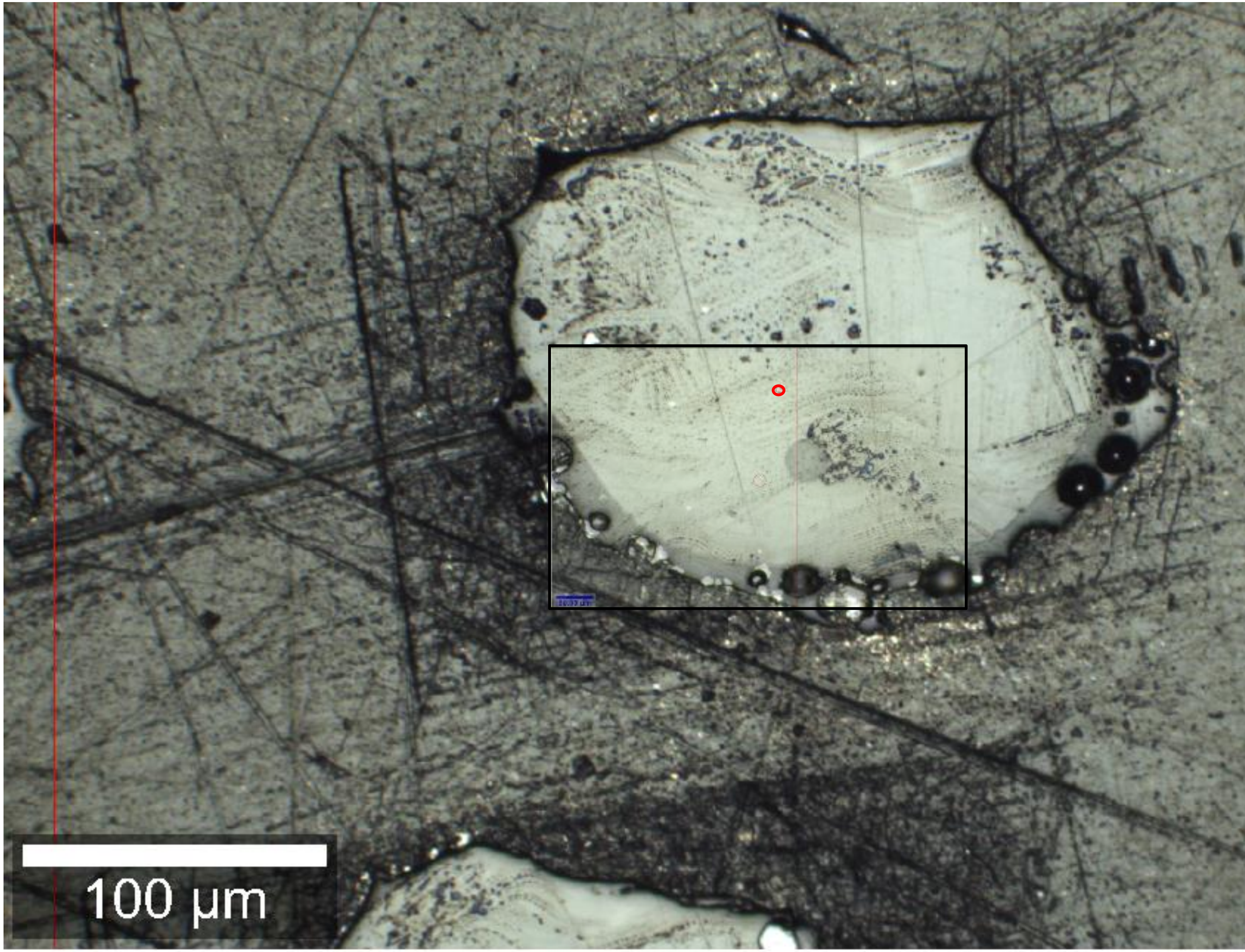

ML_ORI_1_ol15_C

Density: 0.110375196

ML_ORI_1_ol15_B

Density: 0.094450675

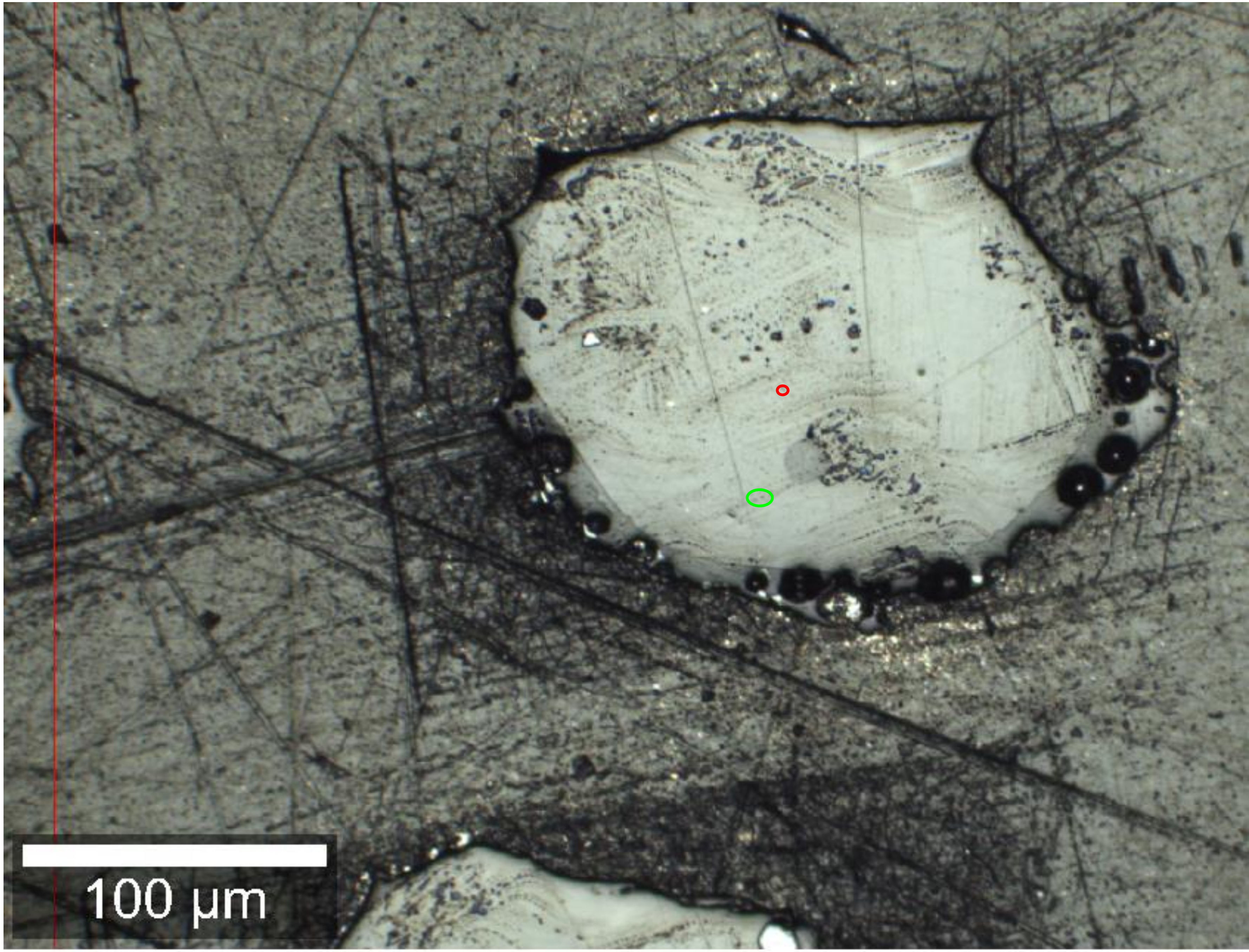

ML_ORI_2

ML_ORI_2_OL2_A

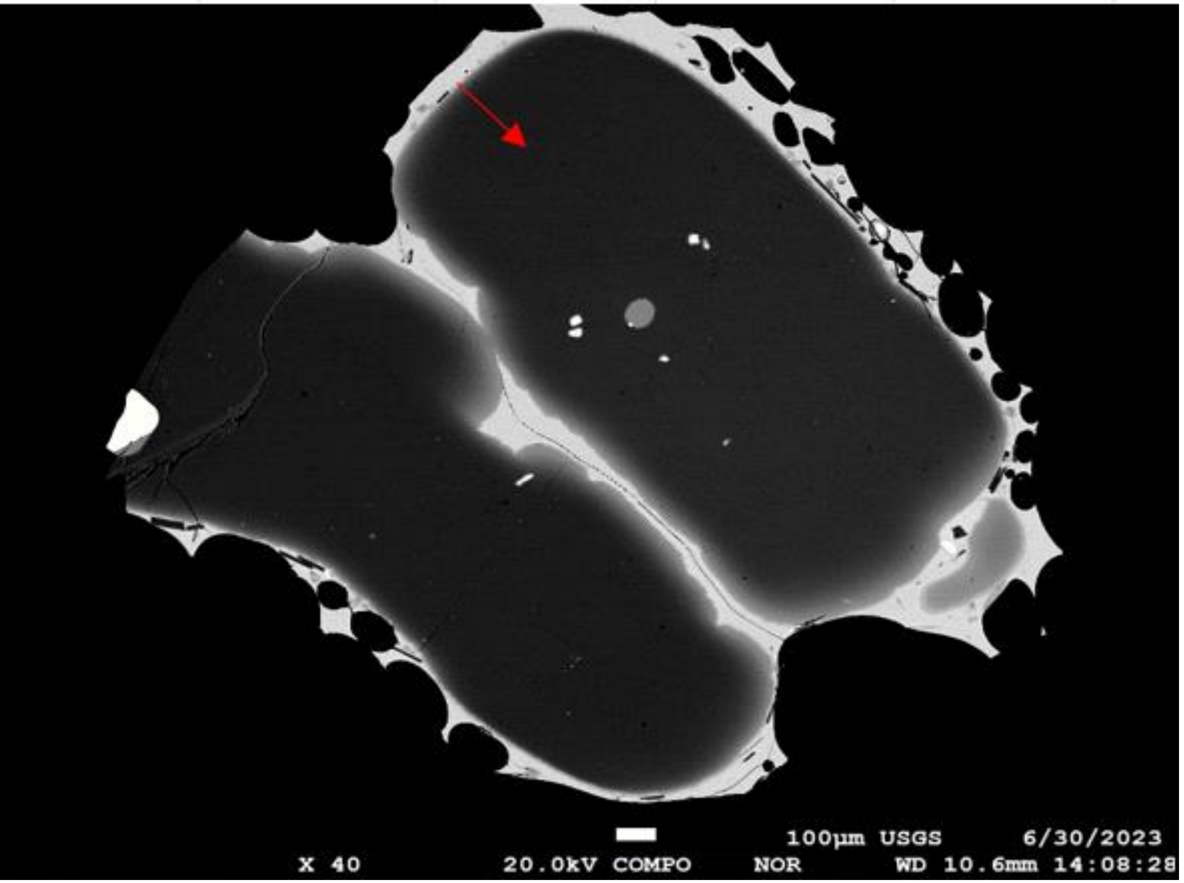

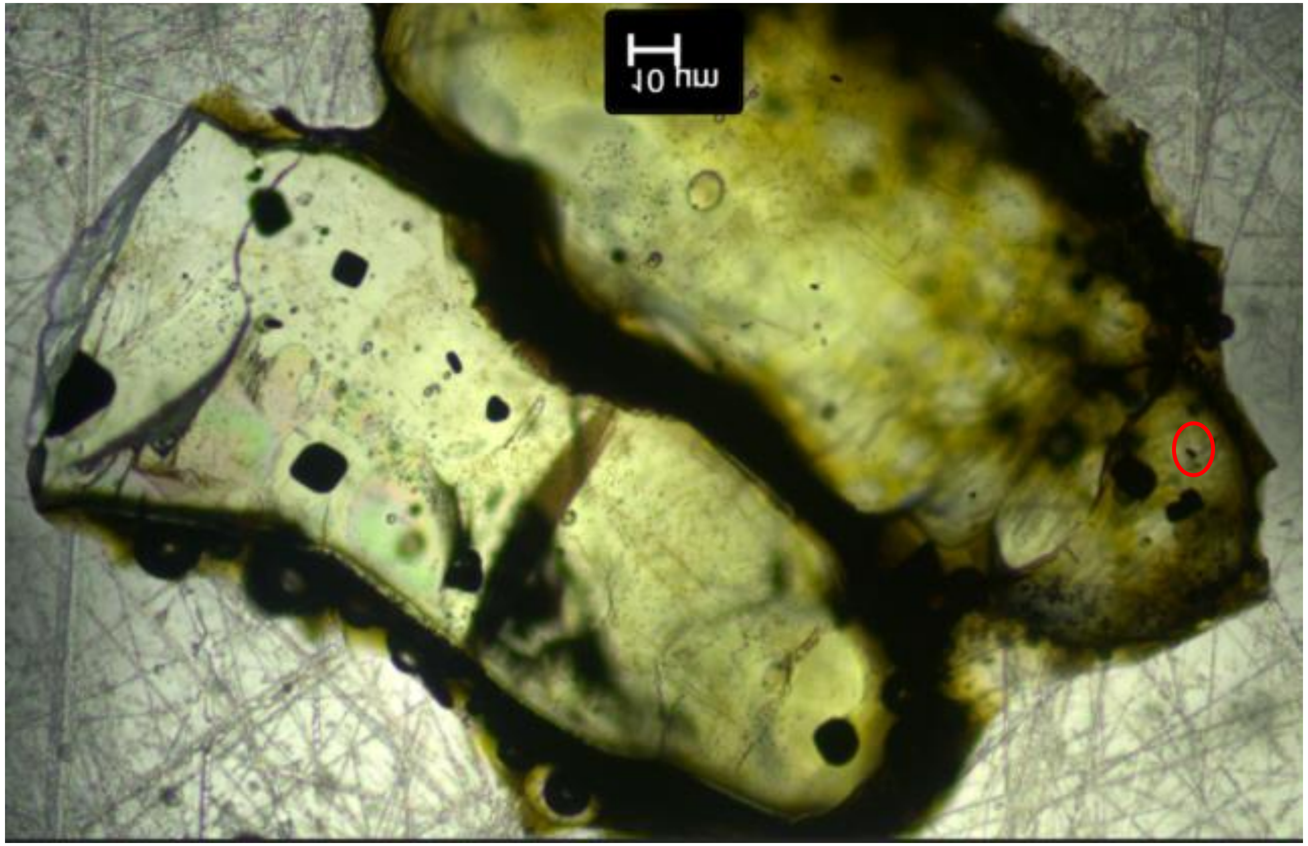

ML_ORI_2_OL2_A

Density:0.211159321

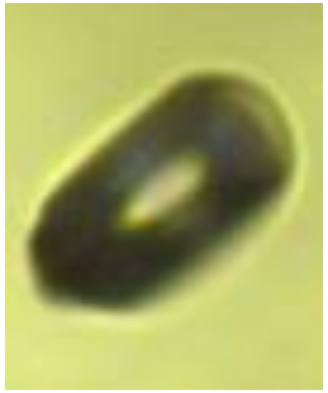

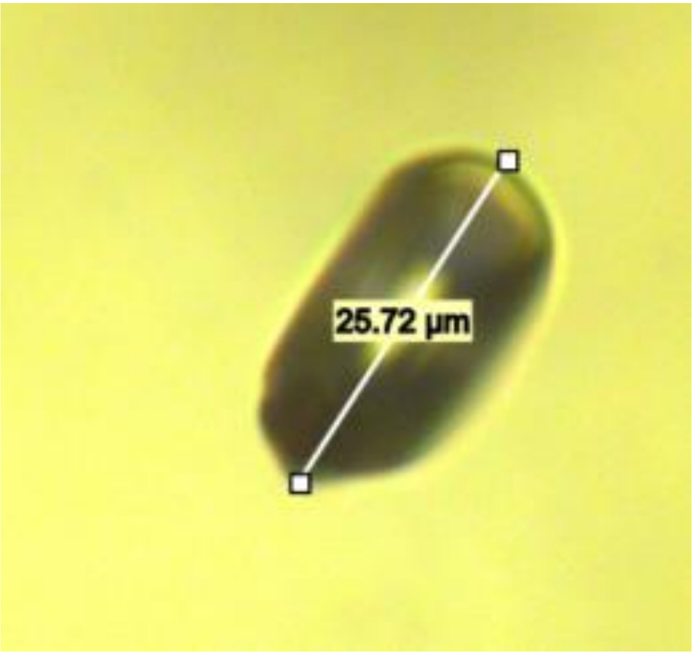

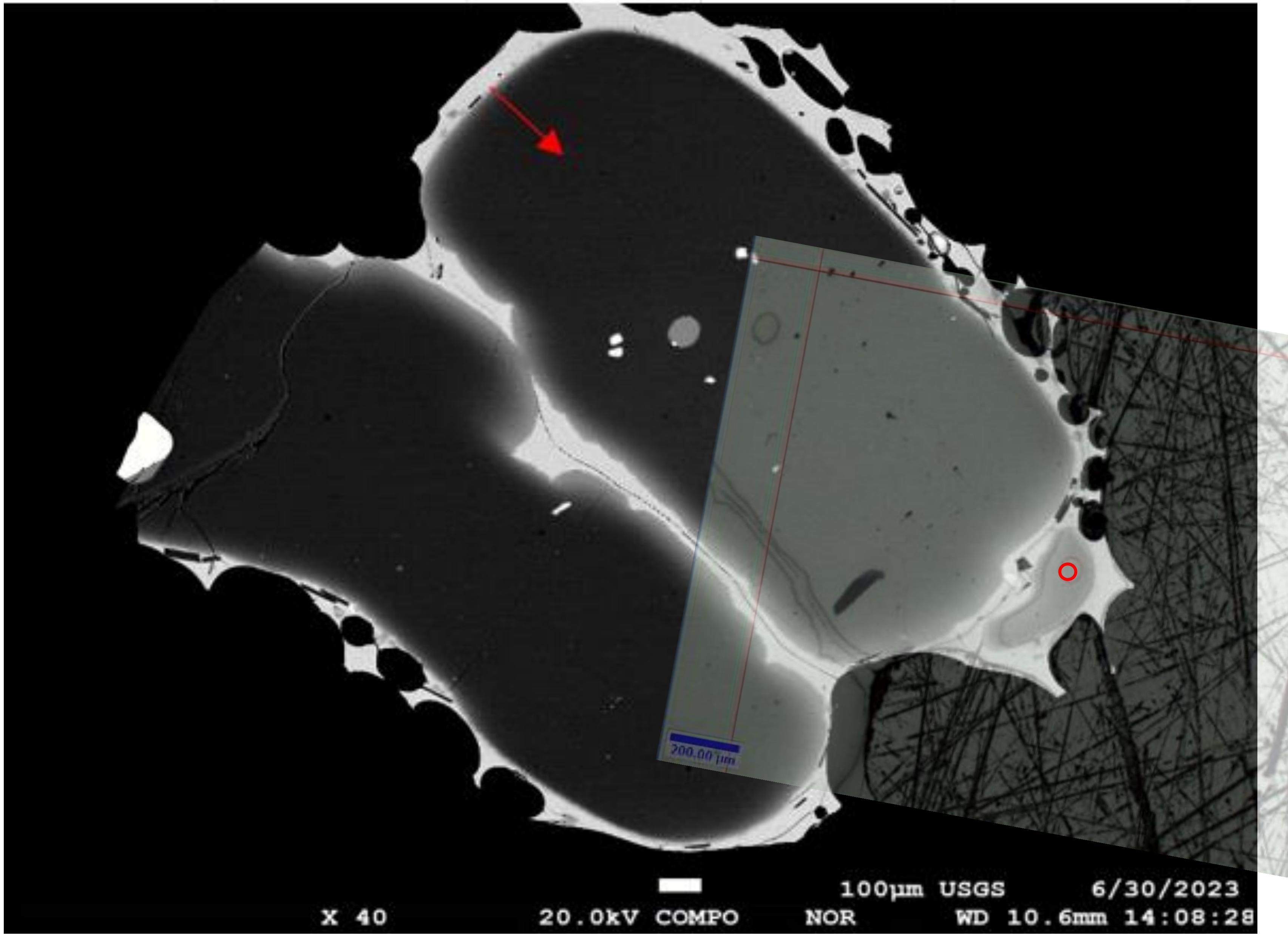

ML_ORI_2_OL11_A

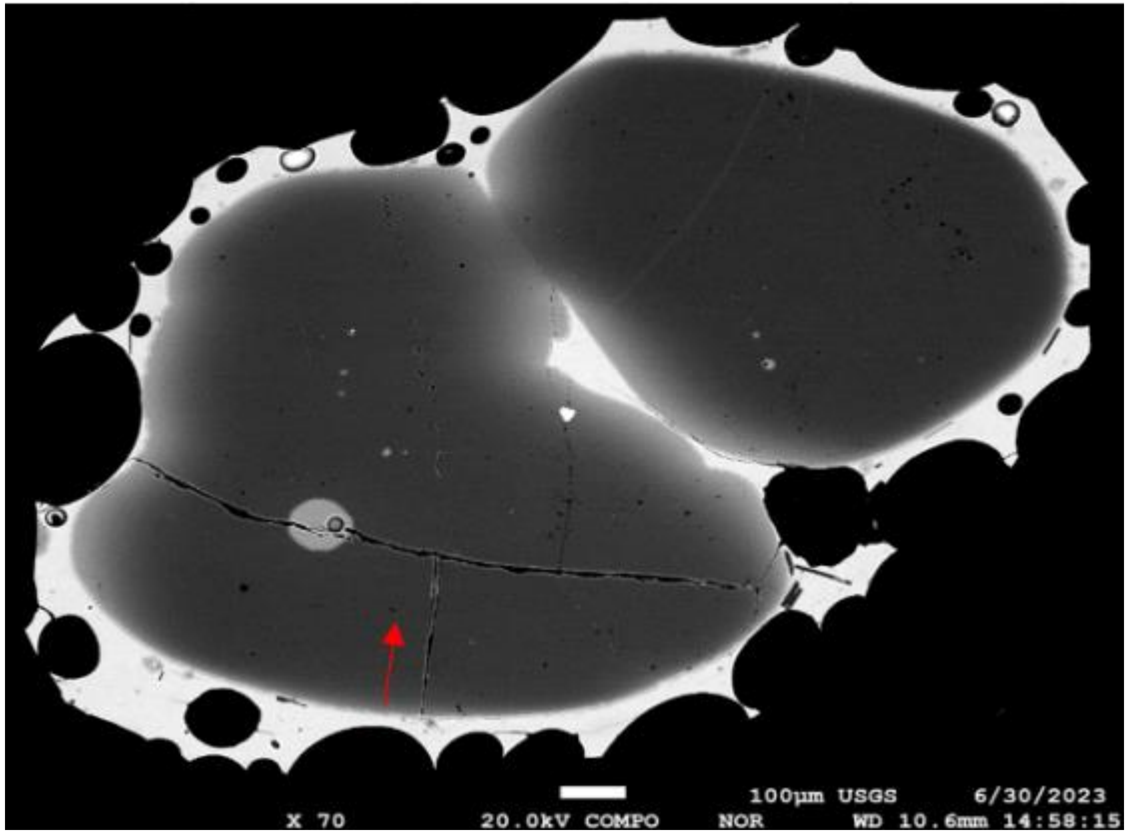

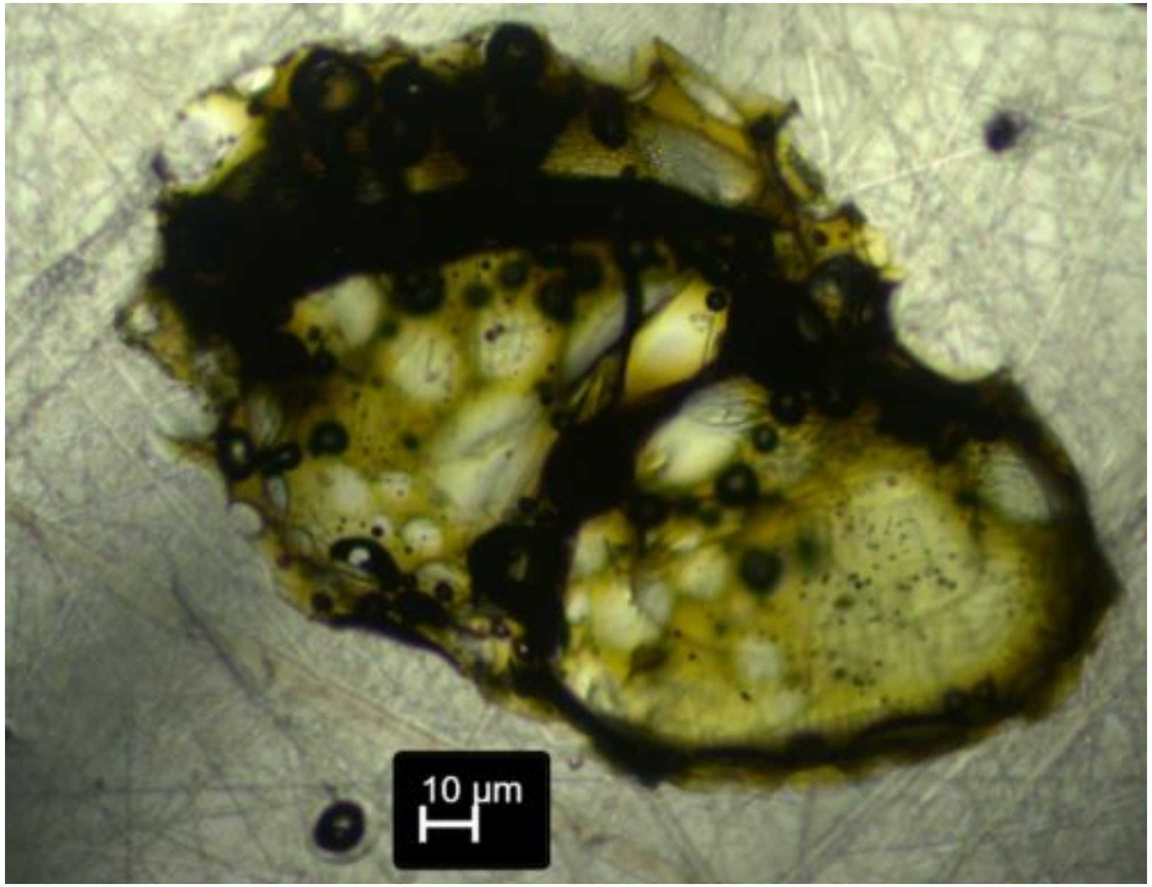

ML_ORI_2_OL11_A

Density:0.106189899

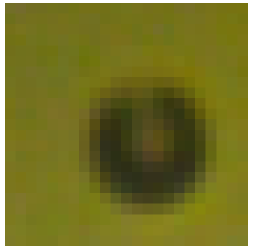

5.32µm

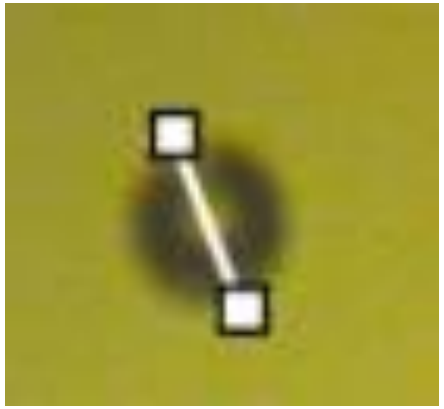

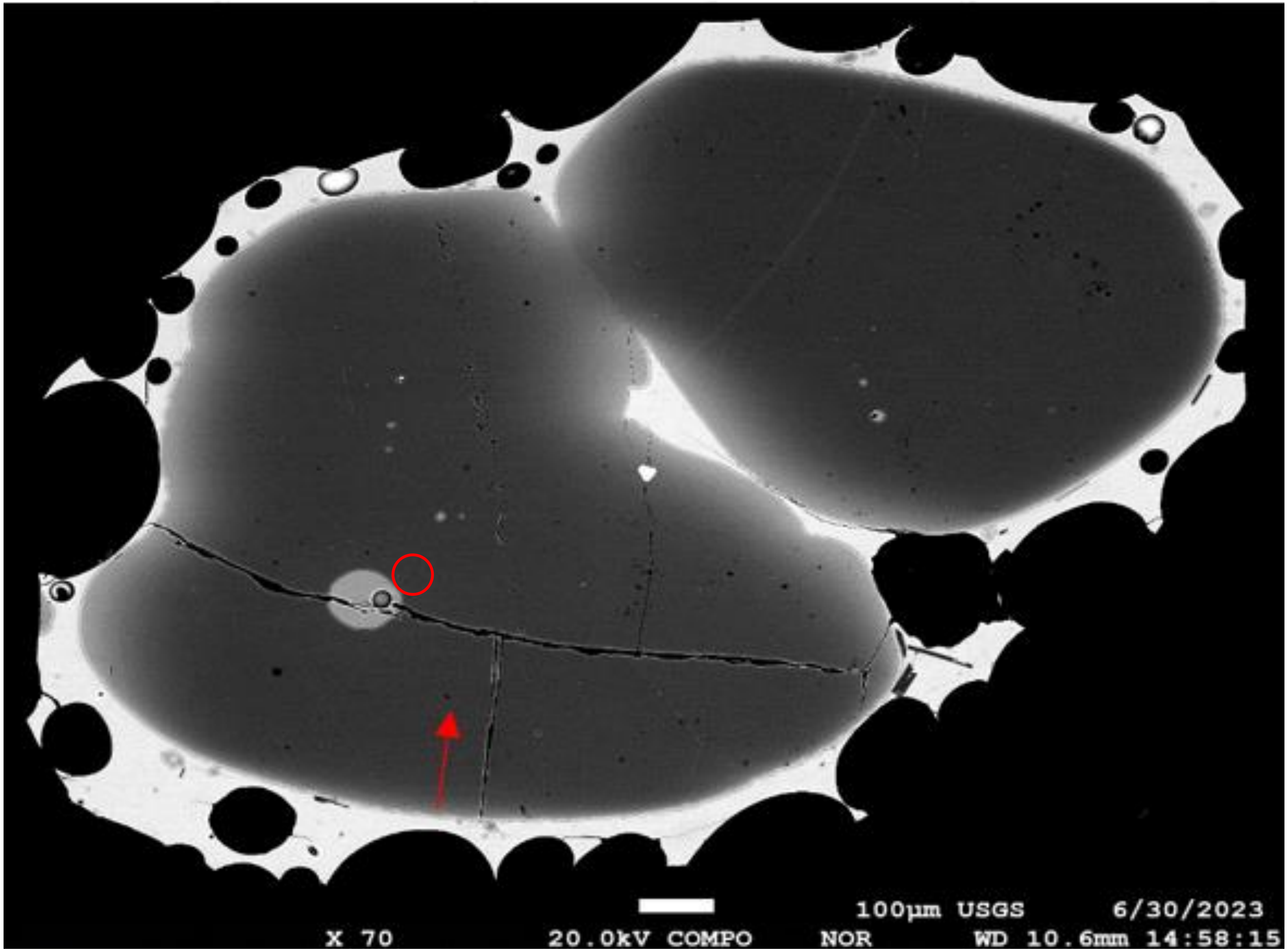

ML_ORI_3

ML_ORI_3_OL19_A

NO EPMA

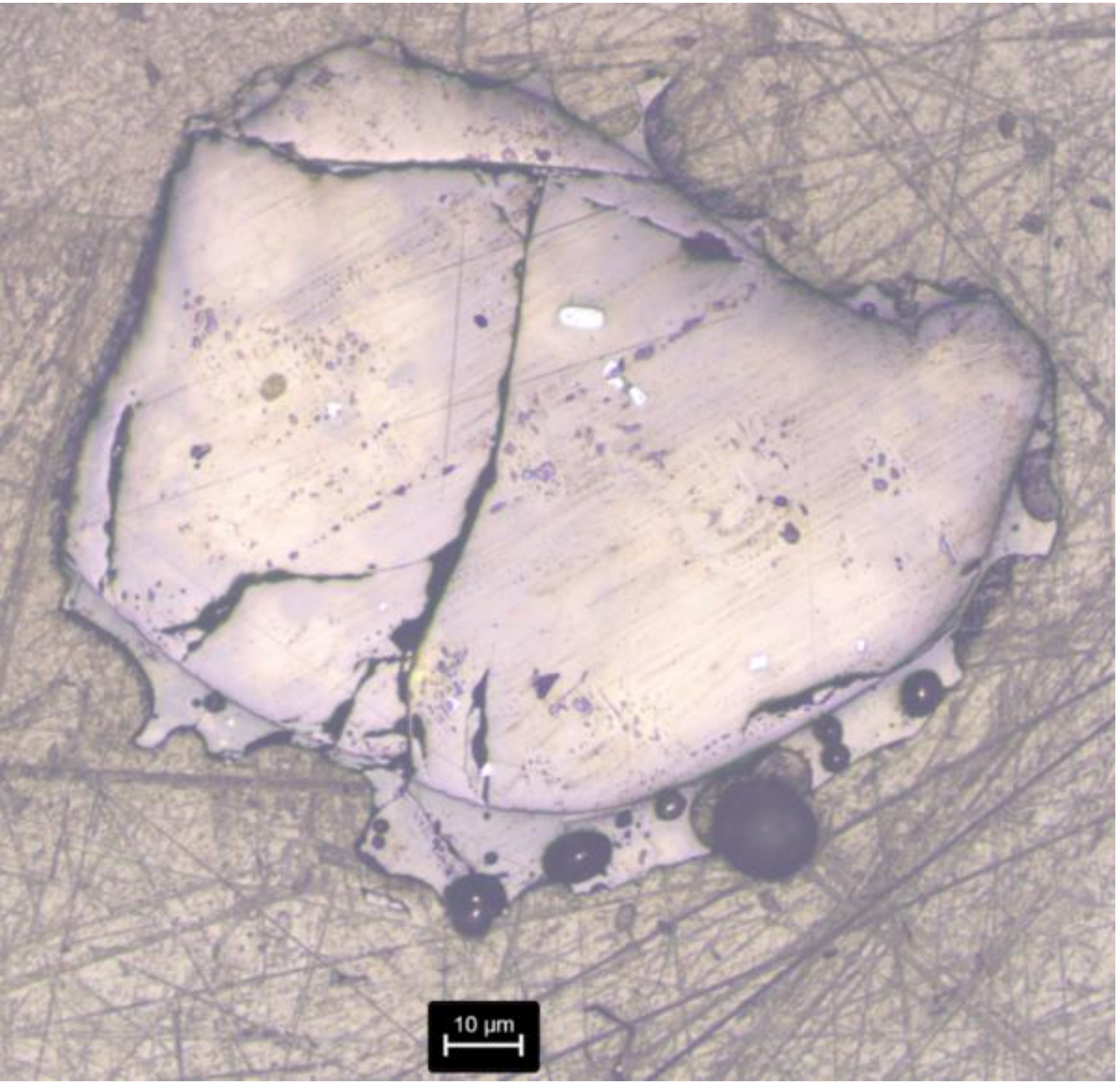

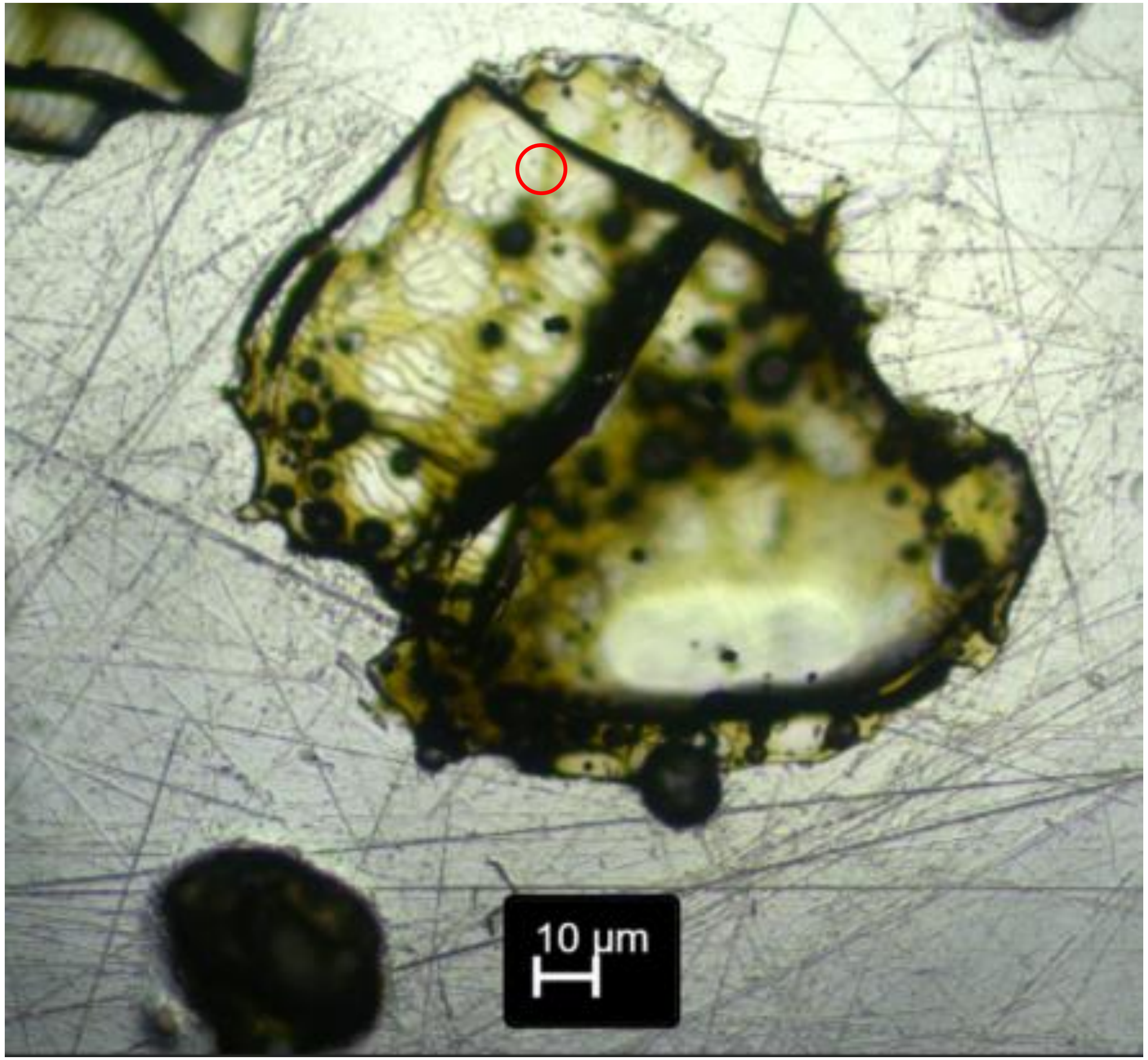

ML_ORI_3_OL19_A

Density:0.162273193

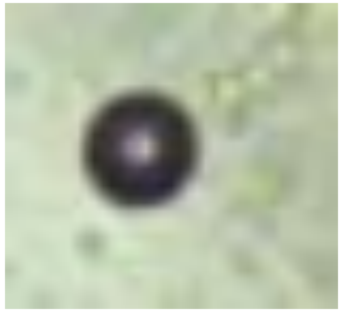

5.32µm

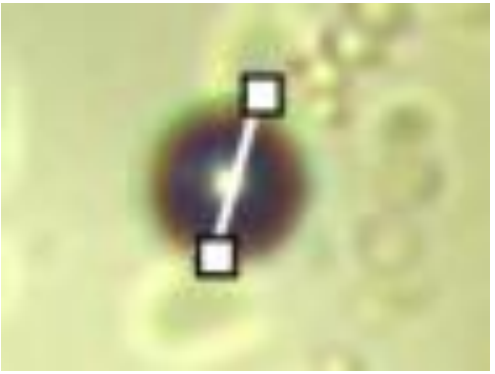

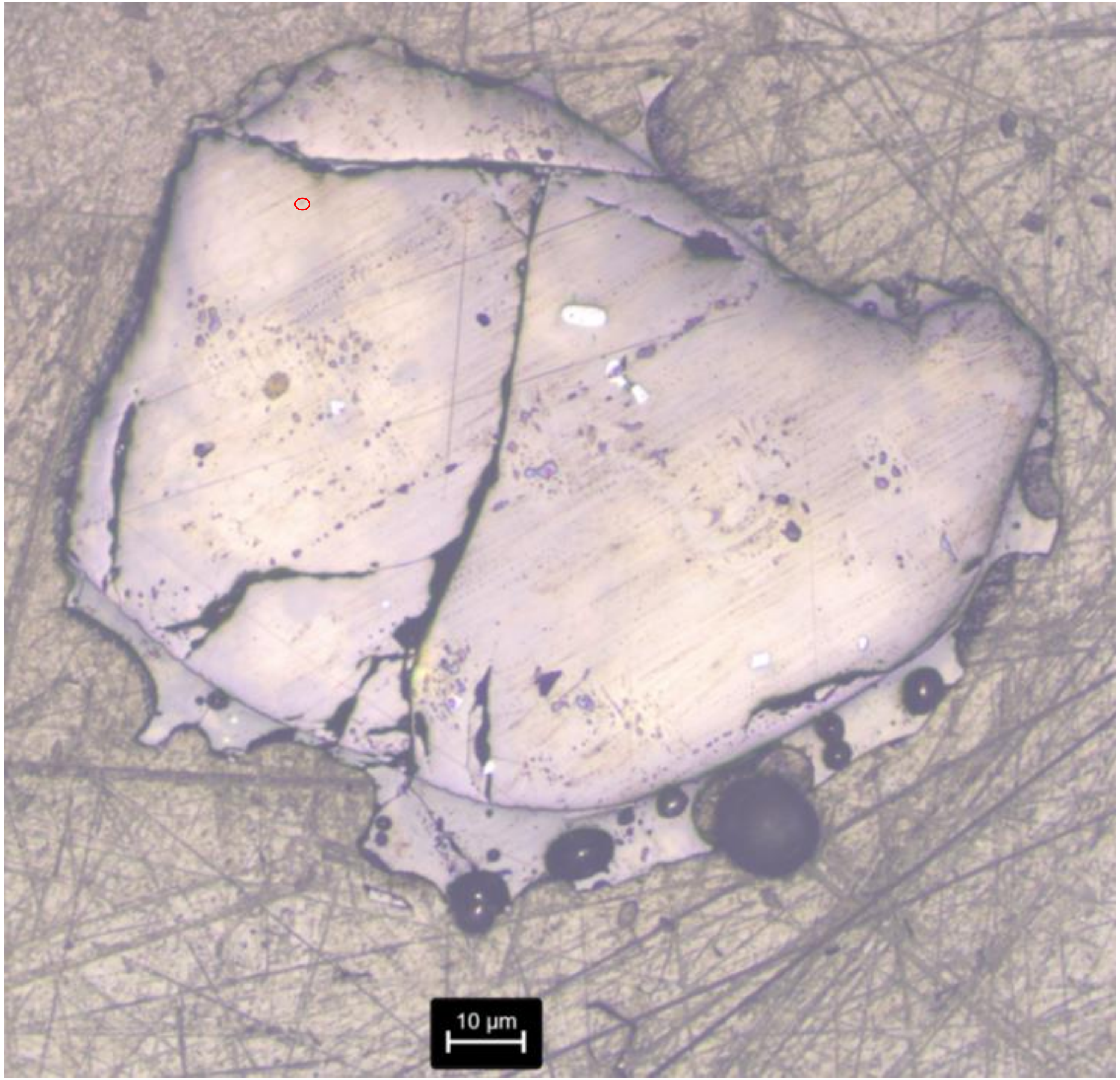

Supplement: Supplementary file 1 — Supplementary Information [file 41467_2024_52881_MOESM1_ESM.pdf]
