# Supplementary material for: Triggering the 2022 eruption of Mauna Loa
Source: Nat Commun. 2024 Nov 12;15:9451. doi: 10.1038/s41467-024-52881-7 (PMC11557896; doi:10.1038/s41467-024-52881-7)
Supplement: Supplementary file 3 — Description of Additional Supplementary Files [file 41467_2024_52881_MOESM3_ESM.pdf]

Description of Additional Supplementary Files

File Name: Supplementary Video 1
Description: Video of micro-CT scan of a Mauna Loa 2022 olivine crystal. Adhering glass and olivine crystal have been made transparent to see the distribution of fluid inclusions (blue), melt inclusions (yellow) and cr-spinels (red).

File Name: Supplementary Video 2
Description: Video of micro-CT scan of a Mauna Loa 2022 olivine crystal. Adhering glass and olivine crystal have been made transparent to see the distribution of fluid inclusions (blue), melt inclusions (yellow) and cr-spinels (red).

File Name: Supplementary Data 1
Description: Ten tabs of methods metadata, secondary standards from electron probe microanalysis of glass and olivine, olivine core and rim compositions, electron backscatter diffraction data, diffusion modelling timescales, raman spectroscopy data for fluid inlusions, fluid inclusion modelling simulations, multi-GAS volatile data, historically active volcanoes and those that have diffusion studies (used to make Fig. 1 in main text), and 61 tabs for each olivine and enstatite core-to-rim transect data with diffusion model input parameters.
